# Accessing Medium-Sized Rings via Vinyl Carbocation Intermediates

**DOI:** 10.1021/acs.orglett.3c04014

**Published:** 2024-01-31

**Authors:** Zhenqi Zhao, Stasik Popov, Woojin Lee, Jessica E. Burch, David A. Delgadillo, Lee Joon Kim, Mona Shahgholi, Naiara Lebrón-Acosta, K. N. Houk, Hosea M. Nelson

**Affiliations:** † Division of Chemistry and Chemical Engineering, California Institute of Technology, Pasadena, California 91125, United States; ‡ Department of Chemistry and Biochemistry, University of California, Los Angeles, Los Angeles, California 90095, United States

## Abstract

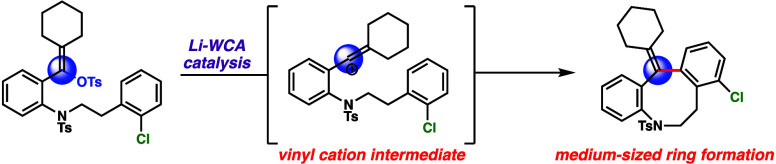

Medium-sized rings (8–11-membered cycles) are often more challenging to synthesize than smaller rings (5–7-membered cycles) due to ring strain. Herein, we report a catalytic method for forming 8- and 9-membered rings that proceeds via the intramolecular Friedel–Crafts reactions of vinyl carbocation intermediates. These reactive species are generated catalytically through the ionization of vinyl toluenesulfonates by a Lewis acidic lithium cation–weakly coordinating anion salt.

Cyclic structural motifs are ubiquitous in natural products, pharmaceuticals, and other industrially relevant compositions of matter.^1,2^ Among them, 5- and 6-membered rings are the most common cyclic structures due to their ease of preparation.^3,4^ In contrast, medium-sized rings (8–11-membered rings) are often more difficult to access, where methods commonly utilized to forge 6- or 5-membered rings fail. Unlike macrocycles (≥12-membered rings), medium-sized rings suffer from torsional and transannular strain; therefore, their annulation reactions can be less favorable and sluggish.^3−7^ As a result, medium-sized rings appear less frequently in synthetic molecules, hindering their utility across a broad range of applications.

Despite their challenging formation, compounds with medium-sized rings are abundant in natural products.^8,9^ For some bioactive compounds bearing medium-sized cyclic motifs, it has been proposed that the unique balance of structural rigidity and broad conformational space enables higher binding affinities for biological targets relative to small ring analogues.^10^ Despite these facts, the number of methods for medium-sized ring formation remains limited in organic synthesis. Ring expansion from smaller rings is widely used to generate medium-sized rings; however, these reactions need to be carefully designed depending on the structure of the medium-sized ring desired and usually require several synthetic steps toward well-poised, smaller ring precursors.^11^ For direct annulation methods, catalytic ring-closing metatheses and cross-coupling reactions are the most common, but precious noble metals such as palladium and ruthenium are required as catalysts.^12,13^ Medium-sized ring formation through radical intermediates has also been reported, although stoichiometric radical sources are commonly used.^12,13^ As a result, developing catalytic annulation reactions to access medium-sized rings is still of great interest.

In recent years, our group has developed various platforms for generating vinyl carbocation intermediates.^14−18^ The most prominent method is Lewis acid–weakly coordinating anion (WCA) catalysis, in which vinyl trifluoromethanesulfonates (vinyl triflates) are ionized to form kinetically persistent vinyl cation intermediates.^14,15^ These reactive species can then engage in C–H insertion (Figure 1A) and intermolecular Friedel–Crafts reactions. In this paper, we report that vinyl carbocations can also be used to forge challenging medium-sized ring systems (Figure 1B).

**Figure 1:**
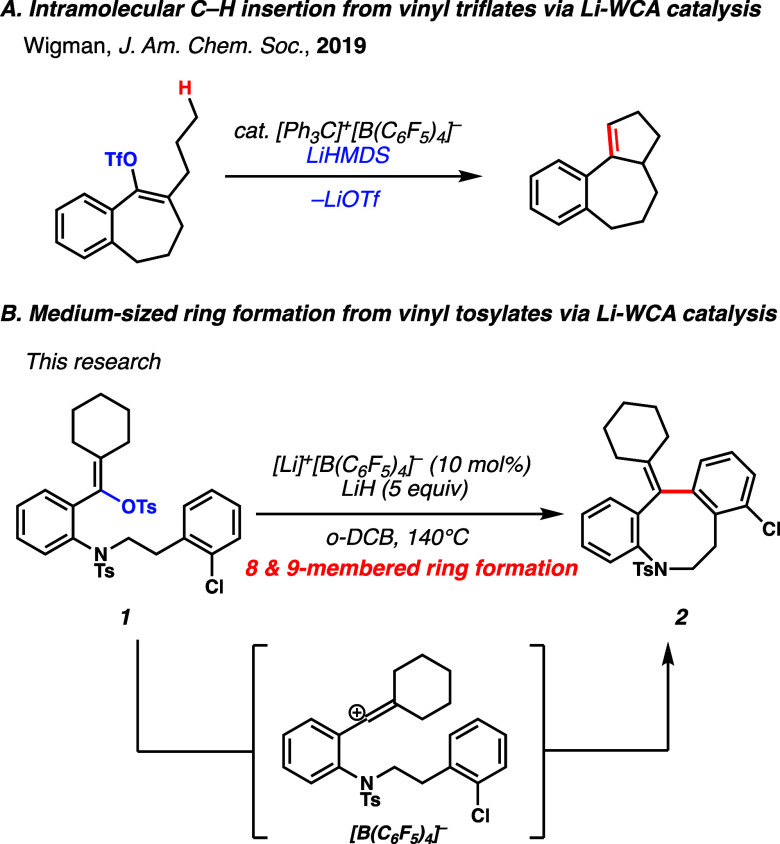
C–C bond formation via Lewis acid–WCA catalysis. (A) Intramolecular C–H insertion reactions from vinyl triflates via Li–WCA catalysis. (B) Medium-sized ring formation via Li–WCA catalysis (this work).

Vinyl triflates have served as vinyl carbocation precursors in previous studies.^14−16^ However, due to the difficulty in preparing pure samples of electron-rich vinyl triflates, we investigated vinyl toluenesulfonates (vinyl tosylates).^19^ As such, vinyl tosylate **1** was selected as our model substrate. A sulfonamide was introduced into the aniline-derived scaffold to protect the amine moiety, a common functional group in many bioactive molecules.^20,21^ We proposed that vinyl tosylate **1** would transform into tetrahydroazocine **2** under Li–WCA catalysis. Medium-sized ring **2** features an *exo*-alkene on the 8-membered ring, which is reminiscent of commercial drugs pizotifen,^22^ amitriptyline,^23^ and cyproheptadine,^24^ but these are comprised of more readily prepared 7-membered rings instead of 8-membered rings. The established route to these drugs features a key intramolecular Friedel–Crafts acylation of a carboxylic acid to forge their core 7-membered ring. As there are few reports about building larger medium-sized rings via Friedel–Crafts acylation,^25,26^ our complementary method provides access to underexplored chemical space via vinyl carbocation intermediates.

Recognizing that electron-deficient arenes are sluggish nucleophiles, we questioned whether electrophilic vinyl cation species could engage them in Friedel–Crafts reactions. Therefore, we began optimization with vinyl tosylate **1** to study the Friedel–Crafts reactions with electrophilic vinyl cation species (Table 1). When vinyl tosylate **1** was subjected to 10 mol % lithium tetrakis(pentafluorophenyl)borate {[Li]^+^[B(C_6_F_5_)_4_]^−^} (**3**) in 1,2-dichlorobenzene (*o*-DCB) at 140 °C, tetrahydroazocine **2** was formed in 40% yield (entry 1). The structure of product **2** was confirmed using microcrystal electron diffraction (microED).^27^ Because a significant amount of starting material remained after long reaction times (entry 1), we hypothesized that adding a lithium base could help regenerate the lithium catalyst and improve the reaction yield. Indeed, adding an excess of LiH increased the yield to 73% (entry 2). In contrast, the presence of lithium bis(trimethylsilyl)amide (LiHMDS), which was used in previous reports,^15,16^ was detrimental to the reaction, forming the product in 21% yield (entry 3). Performing the reaction without [Li]^+^[B(C_6_F_5_)_4_]^−^ did not provide tetrahydroazocine **2** (entry 4). Smaller loadings of [Li]^+^[B(C_6_F_5_)_4_]^−^ gave lower yields of the product (entries 5 and 6), highlighting the essential role of [Li]^+^[B(C_6_F_5_)_4_]^−^ in this catalytic cyclization. Solvents other than *o*-DCB were also examined but were found to be inferior (entries 7–9). Hydrogen bonding catalyst **4**, which our group had previously applied in the ionization of vinyl triflates, gave diminished yields (entries 10 and 11).^16^

**Table 1:**
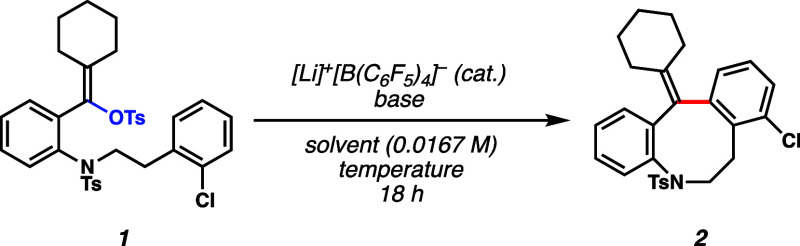
Optimization of the Reaction Conditions to Build Medium-Sized Rings^a^

entry	catalyst (mol %)	base (equiv)	solvent	temperature (°C)	yield (%)
1	3 (10)	none	*o*-DCB	140	40
2	3 (10)	LiH (5)	*o*-DCB	140	73
3	3 (10)	LiHMDS (1.5)	*o*-DCB	140	21
4	none	LiH (5)	*o*-DCB	140	nd
5	3 (5)	LiH (5)	*o*-DCB	140	49
6	3 (1)	LiH (5)	*o*-DCB	140	24
7	3 (10)	LiH (5)	*o*-DFB	92	nd
8	3 (10)	LiH (5)	mesitylene	140	50
9	3 (10)	LiH (5)	DMF	140	nd
10	4 (10)	LiH (5)	*o*-DCB	140	19
11	4 (10)	LiHMDS (1.5)	*o*-DCB	140	nd

a Yields determined by ^1^H NMR using 1,4-dioxane as an internal standard. Abbreviations: Ts, *p*-toluenesulfonyl; *o*-DCB, 1,2-dichlorobenzene; *o*-DFB, 1,2-difluorobenzene; DMF, dimethylformamide; LiHMDS, lithium bis(trimethylsilyl)amide.

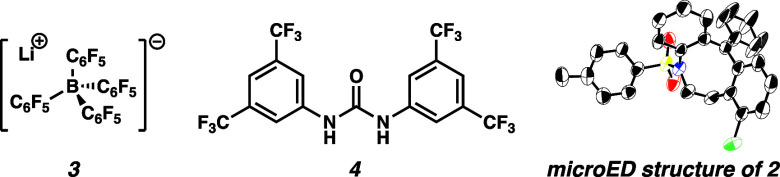

With the optimized conditions, we explored the substrate scope. First, we tested various ring sizes. Similar to vinyl tosylate **1**, the substrate with a nonsubstituted aryl nucleophile also gave the 8-membered ring product in moderate yield [**5** (Figure 2)]. A 9-membered ring was also formed under this system, giving tetrahydroazonine **6** in 82% yield. However, the formation of a 10-membered ring proved to be difficult, as hexahydroazecine **7** was not observed under the reaction conditions. We also found that the sulfonamide could be replaced with other functional groups. For example, thioether **8** was obtained with a moderate yield of 46%, and medium-sized carbocycle **9** could be synthesized in 81% yield. Nine-membered ring ether **10** could be produced in 65% yield with an electron-rich arene as the nucleophile. Substitution effects on the aryl nucleophiles were also studied. Phenyl groups with the dimethylamino and methoxy groups could give 8-membered ring products with good yields (**11** and **12**, respectively). Notably, *tert*-butyldimethylsilyl (TBS)-protected phenol was also tolerated under the reaction conditions as a 79% yield of **13** was obtained. Unfortunately, when the strong electron-withdrawing group trifluoromethyl was present on the aryl group, product **14** was not formed. With a weak electron-withdrawing group, such as bromine, medium-sized ring product **15** could be obtained smoothly in 79% yield. The electronic effect of the aryl ring vicinal to the vinyl tosylate in the starting material was also examined. With an electron-donating methoxy group, product **16** was formed in 78% yield. Conversely, product **17** was not obtained because the respective vinyl tosylate with an electron-withdrawing trifluoromethyl group had no reactivity, which could be due to the challenging ionization to the vinyl cation intermediate. Furthermore, heterocycles could also be used in the reaction. Thiophene was tolerated, yielding 8-membered ring products **18** and **19** in 73% and 85% yields, respectively. The two aryl groups fused with the medium-sized ring in the product were important to this cyclization. Product **20** could not be formed when only one fused aryl ring was on the 8-membered ring. Reducing the sp^2^ carbon in the medium-sized ring in **20** (four sp^2^ carbon atoms instead of five) might introduce more transannular strain and make the cyclization more challenging.^7^ To show that the reaction is scalable, tetrahydroazocine **5** was synthesized with a 66% yield on a 1 mmol scale (0.4 g).

**Figure 2:**
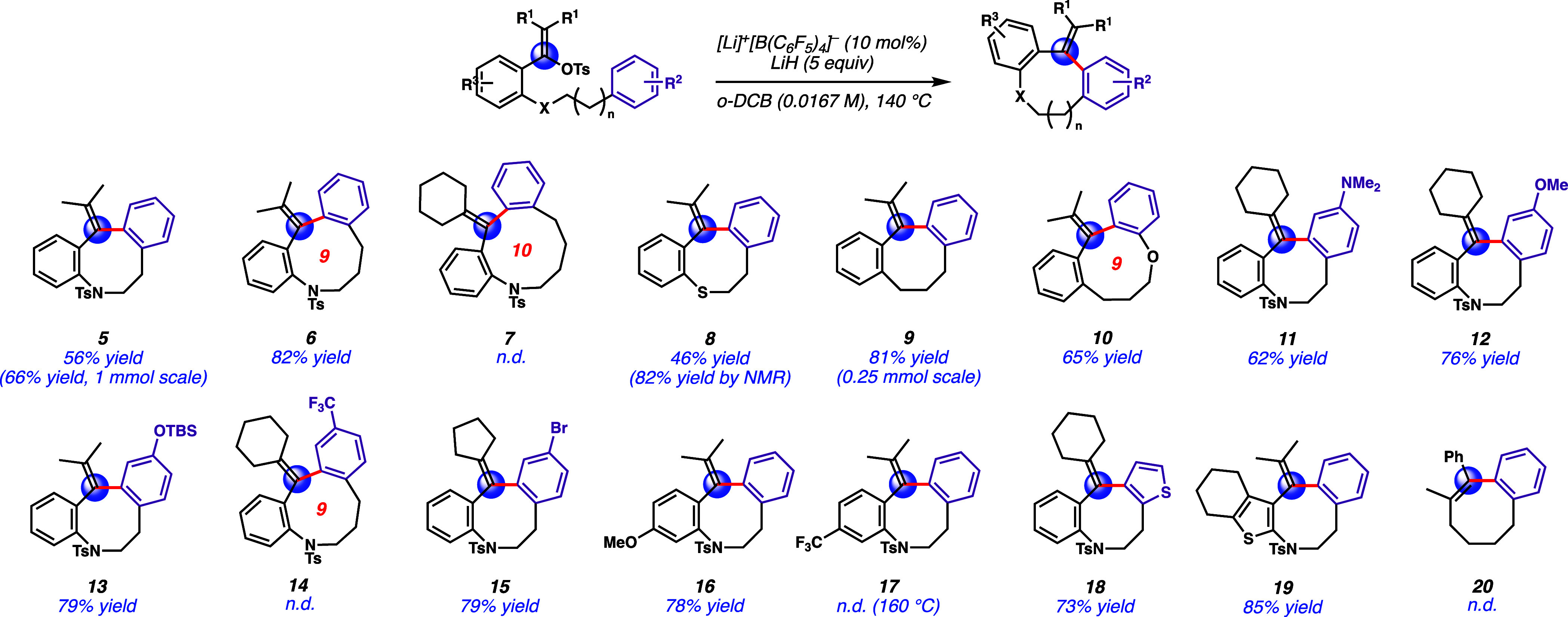
Scope of Li–WCA-catalyzed medium-sized ring formation. The reactions were performed on a 0.05 mmol scale unless otherwise specified. All yields were isolated unless specified. All structures were characterized by NMR. The structures of **5**, **9**, **12**, **13**, **16**, and **17** were also characterized by MicroED.

Because the formation of medium-sized rings through direct cyclization is challenging, we decided to study the reaction mechanism further. Lithium–WCA catalysis systems employing [Li]^+^[B(C_6_F_5_)_4_]^−^ have been demonstrated to ionize vinyl sulfonates to vinyl cations.^15^ Here, we proposed three possible pathways in forming 8-membered ring **27** from vinyl cation **22** (Figure 3A). Path 1 is a conventional Friedel–Crafts reaction of the vinyl cation in which medium-sized ring intermediate **23** is formed in one step. In path 2, the vinyl cation reacts with the aromatic π-system at the *ipso* carbon to form a 7-membered ring in **24**, which often harbors less ring strain than the corresponding 8-membered ring. A 1,2-shift of the alkyl group then occurs to expand the ring to give intermediate **23**. Alternatively, in path 3, a concerted insertion of the vinyl cation into an aryl C–H bond is operative, mechanistically analogous to the insertion of vinyl cations into alkyl C–H bonds.^14−16^

**Figure 3:**
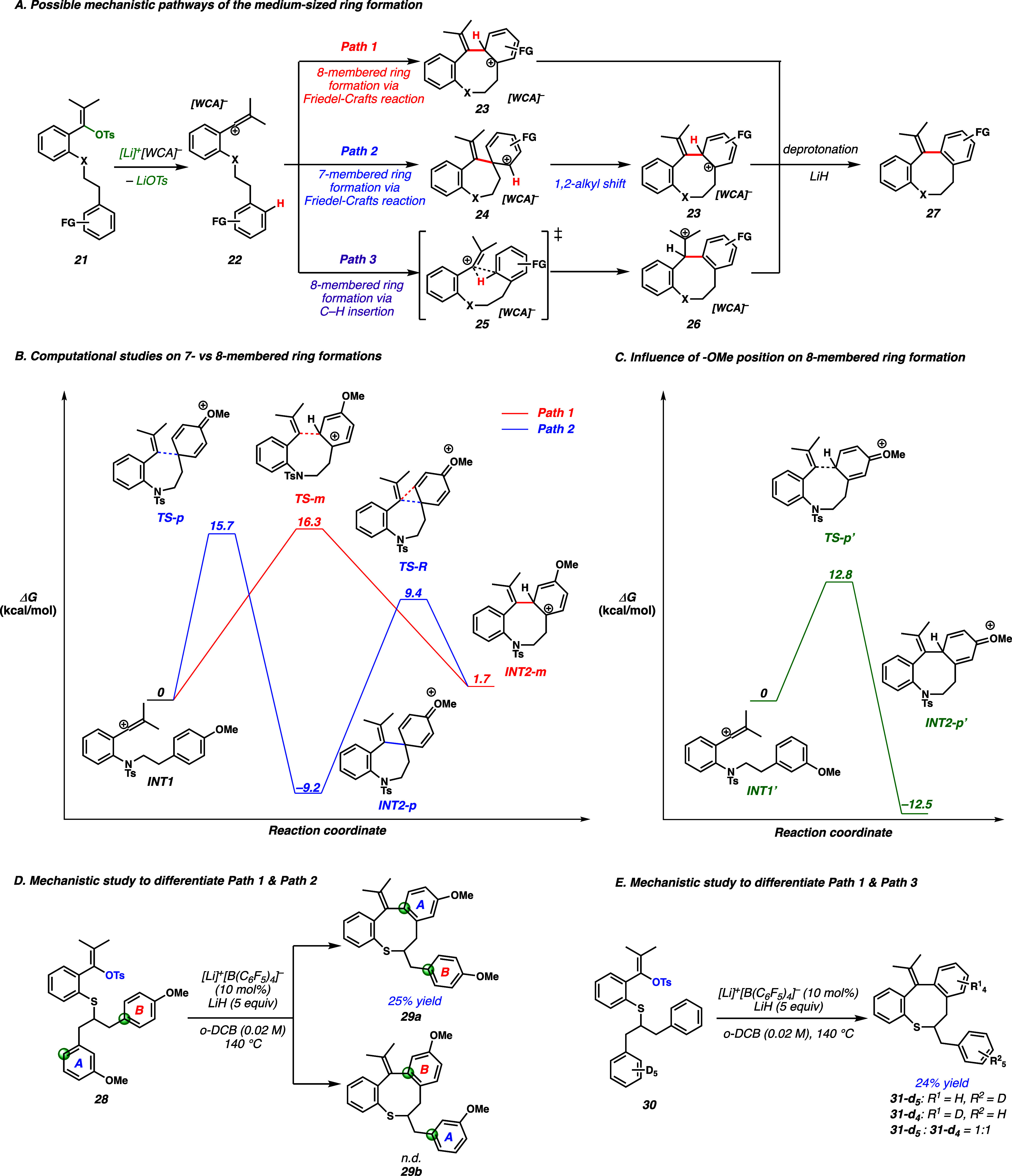
(A) Possible mechanistic pathways. (B) Computational investigation of the medium-sized ring formation of vinyl cations. (C) Influence of the -OMe position on medium-sized ring formation. (D) Mechanistic study for paths 1 and 2. The yield was determined by NMR with an internal standard. (E) Mechanistic study for path 3. The ratio was determined by FD-MS.

To differentiate the potential mechanisms of paths 1 and 2, these proposed pathways were evaluated by density functional theory (DFT) calculations (Figure 3B).^28^**INT1** can undergo the hypothetical Friedel–Crafts reaction via **TS-m** (16.3 kcal/mol) to form 8-membered ring **INT2-m** (path 1). For the other putative mechanism shown in path 2, **INT1** goes through 7-membered ring formation via **TS-p** (15.7 kcal/mol) and subsequent 1,2-alkyl shift **TS-R** (9.4 kcal/mol). Potentially because of ring strain and stabilization from oxonium resonance, arenium **INT2-p** is thermodynamically more stable than **INT2-m**. The alkyl shift of **INT2-p** is energetically feasible, given that the deprotonation step cannot be attained from **INT2-p**. These calculations support the anisyl substituent [**12** (Figure 2)] proceeding through either path 1 or path 2 because ΔΔ*G*^⧧^ is only 0.6 kcal/mol. Because of the small energy difference between paths 1 and 2, we carried out further computations to probe the influence of electronic effects (Figure 3C). Here, we found that the formation of 8-membered ring **INT2-p′** originating from the electron-rich carbon *para* to methoxy group was considerably favorable relative to both paths 1 and 2 from **INT1**, suggesting a strong electronic bias in **INT1**.

Therefore, vinyl tosylate **28** was designed to experimentally probe the influence of electronic effects on the mechanism (Figure 3D). Tosylate **28** has two aromatic nucleophiles (green spheres highlight the most nucleophilic positions). If path 1 were operative, then ring A would be incorporated into the product [**29a** (Figure 3D)]. Conversely, if 7-membered ring formation occurred first, as in path 2, ring B would be incorporated into the cyclic scaffold (**29b**). Interestingly, tosylate **28** favored the formation of **29a** in 25% yield, although the reaction led to a complex mixture. Various analytical techniques, including NMR and LC-MS, suggested this was the major cyclization product (Supporting Information).

From these calculations and experiments, direct C–H insertion (path 3) could not be excluded. Thus, we prepared vinyl tosylate **30** to probe the feasibility of path 3 (Figure 3E). Under the standard reaction conditions, a mixture of **31-*d***_**5**_ and **31-*d***_**4**_ was obtained with a distribution of roughly 1:1. This result was inconsistent with that of path 3, where a primary kinetic isotope effect in the putative product-determining step would provide a larger ratio of **31-*d***_**5**_ to **31-*d***_**4**_. Overall, the reactions of vinyl tosylates **28** (Figure 3D) and **30** (Figure 3E) both support path 1 as a potential reaction mechanism, consistent with the canonical Friedel–Crafts reactivity.

In conclusion, we have discovered a method for accessing medium-sized rings via vinyl carbocation intermediates. Vinyl tosylates are used as the precursors and ionized into vinyl carbocations under the Li–WCA catalysis system. It is followed by an intramolecular Friedel–Crafts reaction with aryl nucleophiles to form medium-sized rings. These discoveries further demonstrate the application of vinyl cations in chemical synthesis.

## Data Availability

The data underlying this study are available in the published article and its Supporting Information.

## Supporting Information Available

Experimental procedures, characterization data, crystal data, computational data, and NMR spectra (PDF)
