# Supplementary material for: Accessing Medium-Sized Rings via Vinyl Carbocation Intermediates
Source: Org Lett. 2024 Jan 31;26(5):1000–5. doi: 10.1021/acs.orglett.3c04014 (PMC10863392; doi:10.1021/acs.orglett.3c04014)
Supplement: Supplementary file 1 — ol3c04014_si_001.pdf [file ol3c04014_si_001.pdf]

Accessing Medium-sized Rings *via* Vinyl Carbocation Intermediates

Zhenqi Zhao, Stasik Popov, Woojin Lee, Jessica E. Burch, David A. Delgadillo, Lee Joon Kim, Mona Shahgholi, Naiara Lebrón-Acosta, Kendall N. Houk, Hosea M. Nelson

Table of Contents

1. Materials and methods……………………………………………………………..…...2
2. Preparation of vinyl tosylate substrates……………………………………………..….3
3. Friedel-Crafts reactions………………………………………………….….………35
4. Reaction condition screen……………………………………………………………...42
5. Mechanism studies…………………………………………………………………….44
6. Computational studies…………………………………………………………………51
7. MicroED data ………...………………………………………………………………71
8. References……………………………………………………………………………..91
9. ^{1}H NMR, ^{13}C NMR spectral data…………………………………………………...93

1. Materials and methods

Unless otherwise stated, all reactions were performed in a VAC glovebox under nitrogen atmosphere with ≤ 3.0 ppm O_2 levels. All glassware and stir-bars were dried in a 160 °C oven for at least 12 hours and dried *in vacuo* before use. All liquid substrates were rigorously dried (over CaH_2 or filtered through dry neutral aluminum oxide) before use. Ethyl ether, tetrahydrofuran, dichloromethane, dimethylformamide, toluene and hexanes were degassed and dried in a JC Meyer solvent system. Acetonitrile, triethylamine, and pyridine were distilled over CaH_2. 1,2-dichlorobenzene was degassed and dried in a JC Meyer solvent system and stored inside the glovebox for benchtop Friedel-Crafts reactions. Solid substrates were dried over P_2O_5. $[Li]^+[B(C_6F_5)_4]^-$ salts were synthesized according to literature procedure.[1] Thin layer chromatography (TLC) was performed using Millipore silica gel 60 F_{254} pre-coated plates (0.25 mm) and visualized by UV fluorescence quenching. SiliaFlash P60 silica gel (230-400 mesh) was used for flash chromatography. NMR spectra were recorded on a Bruker AV-400 (^1H, ^{13}C), Bruker DRX-500 (^1H, ^{13}C), and Bruker AV-500 (^1H, ^{13}C). ^1H NMR spectra are reported relative to $CDCl_3$ (7.26 ppm) unless noted otherwise. Data for ^1H NMR spectra are as follows: chemical shift (ppm), multiplicity, coupling constant (Hz), integration. Multiplicities are as follows: s = singlet, d = doublet, t = triplet, dd = doublet of doublet, dt = doublet of triplet, ddd = doublet of doublet of doublet, td = triplet of doublet, tt = triplet of triplet, quint = quintet, sept = septet, m = multiplet. ^{13}C NMR spectra are reported relative to $CDCl_3$ (77.0 ppm) unless noted otherwise. IR Spectra were record on a Perkin Elmer 100 spectrometer and are reported in terms of frequency absorption (cm^{-1}). High resolution mass spectra (HR-MS) were recorded on a Waters (Micromass) GCT Premier spectrometer, a Waters (Micromass) LCT Premier, an Agilent GC EI-MS, and are reported as follows: m/z (% relative intensity). Purification by preparative HPLC was done on an Agilent 1200 series instrument with a reverse phase Alltima C_{18} (5m, 25 cm length, 1 cm internal diameter) column. Unless noted otherwise, aluminum dry bath heating blocks were used as the heating source for the reactions that require heating.

2. Preparation of vinyl tosylate substrates

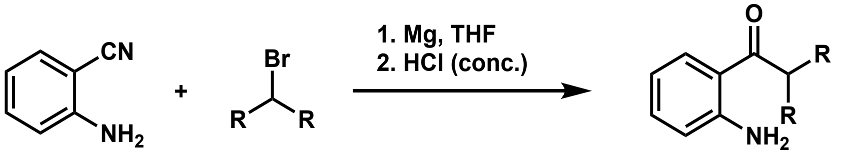

Representative scheme for the reaction between Grignard reagent and aryl nitrile.

General procedure 1: Magnesium (3.0 equiv) was put into a flame-dried three-neck flask equipped with a condenser. THF (12.3 equiv) was then added into the flask. Alkyl bromide (1.0 equiv) was added slowly into the flask to keep the solution under gentle reflux. After the formation of the Grignard reagent, cooled the solution down to 0 °C and 2-aminobenzonitrile (3.0 equiv) in THF (36.9 equiv) was added dropwise. The reaction was run overnight. After this the reaction was quenched with water and concentrated hydrochloric acid to make the pH down to 1. Then it was extracted with ethyl ether three times. The combined organic phase was washed with saturated sodium bicarbonate solution and brine. It was dried with magnesium sulfate, filtered, and concentrated to give the crude product. The crude product was purified *via* flash column chromatography to give the product.

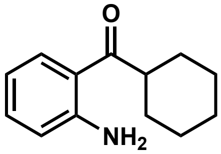

(2-aminophenyl)(cyclohexyl)methanone (SI-1).

Synthesized according to general procedure 1 starting from 2-aminobenzonitrile (3.00 g, 0.0254 mol). Crude product was purified *via* flash column chromatography using 20% ethyl ether in hexanes to give the product as a yellow solid (2.51 g, 48.6% yield).

^{1}H NMR data matches previous report[2].

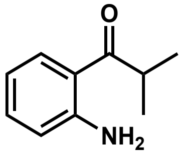

1-(2-aminophenyl)-2-methylpropan-1-one (SI-2).

Synthesized according to general procedure 1 starting from 2-aminobenzonitrile (4.00 g, 0.0423 mol). Crude product was purified *via* flash column chromatography using 10% ethyl ether in hexanes to give the product as a yellow solid (5.45 g, 78.9% yield).

^{1}H NMR data matches previous report[3].

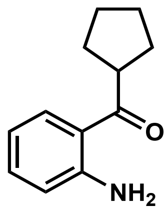

(2-aminophenyl)(cyclopentyl)methanone (SI-3).

Synthesized according to general procedure 1 starting from 2-aminobenzonitrile (9.24 g, 0.0783 mol). Crude product was purified *via* flash column chromatography using 10% ethyl ether in hexanes to give the product as a white solid (13.32 g, 88.8% yield).

^{1}H NMR data matches previous report[4].

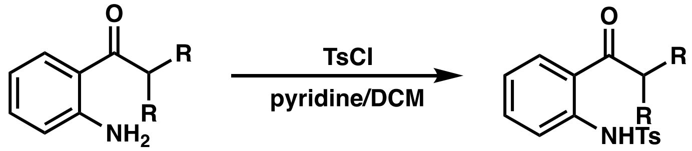

Representative scheme for N-tosylation of anilines.

General Procedure 2: To a round bottom flask was added aniline (1.0 equiv) followed by DCM (13.0 equiv) and pyridine (7.0 equiv). This was cooled to 0 °C and then tosyl chloride (1.42 equiv) was added. The reaction was warmed up to room temperature and stirred for 12 hours. The reaction was diluted with additional DCM (15 equiv) and water. The layers were separated and the aqueous later was extracted twice more with DCM. The combined organics were washed 1M aqueous HCl, water and brine in that order and the dried over magnesium sulfate, filtered and concentrated. The crude product was purified by flash column chromatography to give pure material as a white solid.

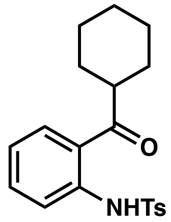

***N*-(2-(cyclohexanecarbonyl)phenyl)-4-methylbenzenesulfonamide (SI-4).**

Synthesized according to general procedure 2 starting from the corresponding aniline **SI-1** (14.9 g, 0.0733 mol). Crude product was purified *via* flash column chromatography using 20% ethyl acetate in hexanes to give sulfonamide **SI-4** as a white solid (26.2 g, 80% yield).

^{1}H NMR data matches previous report[5].

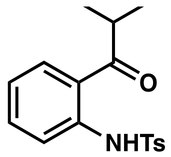

***N*-(2-isobutyrylphenyl)-4-methylbenzenesulfonamide (SI-5).**

Synthesized according to general procedure 2 starting from the corresponding aniline **SI-2** (4.00 g, 0.0245 mol). Crude product was purified *via* flash column chromatography using 30% ethyl acetate in hexanes to give sulfonamide **SI-5** as a white solid (5.15 g, 66% yield).
^{1}H NMR data matches previous report[5].

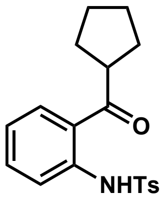

***N*-(2-(cyclopentanecarbonyl)phenyl)-4-methylbenzenesulfonamide (SI-6).**
Synthesized according to general procedure 2 starting from the corresponding aniline **SI-3** (11.8 g, 0.0625 mol). Crude product was purified *via* flash column chromatography using 20% ethyl acetate in hexanes to give sulfonamide **SI-6** as a white solid (10.43 g, 48.59% yield).
^{1}H NMR data matches previous report[6].

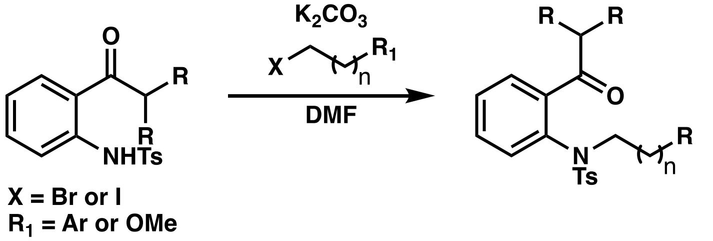

Representative scheme for N-alkylation of sulfonamides.

General Procedure 3: To an oven dried 20 mL scintillation vial was added sulfonamide (1.0 equiv) followed by DMF (13.0 equiv). To the solution was added and potassium carbonate (2.0 equiv) and alkyl iodide/bromide (2.0 equiv unless noted) under a stream of N_2. The vial was sealed and heated to 100 °C for 24 h. The reaction mixture was cooled to rt, diluted with water and ether. The layers were separated, and the aqueous layer was extracted with ether (3x). The combined organics were washed with water (3x) and brine (1x) then dried over $MgSO_4$, filtered, and concentrated to give crude product. The crude product was purified by flash column chromatography.

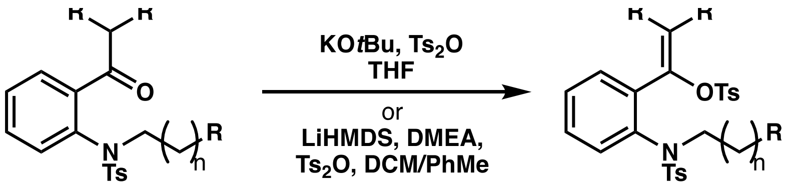

Representative scheme for N-alkylation of sulfonamides.

General Procedure 4 (KO*t*Bu): The corresponding ketone (1 equiv) was dissolved in THF (36.9 equiv) and cooled to 0 °C. To this was added a solution of potassium *tert*-butoxide (1.5 equiv) in THF (18.4 equiv). This was stirred 1.5 hours and then tosic anhydride (1.5 equiv) was added and the reaction was warmed up to rt. After 4 hours, the reaction mixture (generally a thick slurry) was diluted with ethyl acetate. This was washed with water (x1) and brine (x1) then dried over $MgSO_4$,

filtered and concentrated to give crude vinyl tosylate. This was purified by flash column chromatography to give pure vinyl tosylate.

General Procedure 5 (LiHMDS/DMEA): Followed established literature procedure[16]. Inside a glovebox, LiHMDS (2.0 equiv) was dissolved in dry toluene (20.8 equiv) inside a round bottom flask which was then removed from the glovebox. To this was added distilled *N,N*-dimethylethylamine (DMEA, 2.0 equiv) and ketone (1.0 equiv) in dry toluene (9.4 equiv). After stirring for 20 minutes, tosic anhydride (2.0 equiv) in DCM (39.0 equiv) was added and this was stirred for one hour at room temperature. The reaction was then diluted with diethyl ether and 0.25 M aqueous NaOH. The layers were separated and the aqueous was extracted with diethyl ether (x3). The combined organics were washed with brine, dried over $MgSO_4$, filtered and concentrated to give crude vinyl tosylate. This was purified by flash column chromatography to give pure vinyl tosylate.

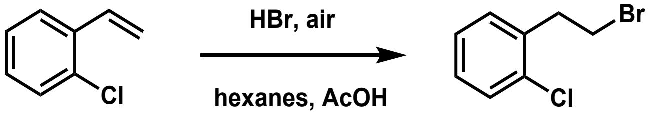

1-(2-bromoethyl)-2-chlorobenzene (SI-7).

1-chloro-2-vinylbenzene (3.45 g, 3.15 mL, 25.0 mmol) in hexanes (112.5 mL) was stirred at 0 °C and the air was bubbled through the solution for 1h. Hydrobromic acid in acetic acid (9.19 mL, 52.5 mmol, 33% w/V) was then added, and the reaction went in the closed flask for 20 min. The reaction solution was washed with saturated sodium bicarbonate solution and brine. Then it was dried with sodium sulfate and concentrated to give the crude product. The product was purified via flash column chromatography using hexanes to give the product as a colorless liquid (3.85 g, 70.2% yield).

^{1}H NMR data matches previous report[7].

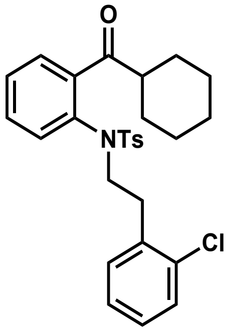

***N*-(2-chlorophenethyl)-*N*-(2-(cyclohexanecarbonyl)phenyl)-4-methylbenzenesulfonamide (SI-8).**

Synthesized according to general procedure 3 starting from the corresponding sulfonamide **SI-4** (0.54 g, 0.0015 mol) and 1-(2-bromoethyl)-2-chlorobenzene **SI-16** (1.31 g, 0.00600 mol). Crude product was purified via flash column chromatography using 25% ethyl ether in hexanes to give sulfonamide **SI-8** as a white powder (0.50 g, 67% yield).

^{1}H NMR (400 MHz, $CDCl_3$) δ 7.59 (dd, *J* = 7.6, 1.8 Hz, 1H), 7.48 (d, *J* = 8.3 Hz, 2H), 7.39 (ddd, *J* = 7.5, 7.5, 1.3 Hz, 1H), 7.33 (ddd, *J* = 7.8, 7.8, 1.8 Hz, 1H), 7.29 (m, 1H), 7.23 (d, *J* = 8.0 Hz,

2H), 7.17 (m, 3H), 6.78 (dd, J = 7.9, 1.2 Hz, 1H), 3.95 (m, 1H), 3.49 (m, 1H), 3.42 (tt, J = 10.8, 3.4 Hz, 1H), 3.10 (m, 1H), 2.94 (m, 1H), 2.41 (s, 3H), 2.11 (m, 1H), 1.94-1.56 (m, 5H), 1.46-1.22 (m, 4H).

^{13}C NMR (101 MHz, $CDCl_3$) δ 206.8, 143.6, 141.8, 136.8, 136.0, 135.5, 134.2, 131.0, 130.6, 129.5, 129.4, 129.3, 128.2, 128.1, 128.0, 127.0, 51.3, 49.3, 32.8, 29.2, 26.0, 25.9, 21.4.

FTIR (Neat film NaCl): 3065, 2928, 2853, 1690, 1596, 1444, 1351, 1159, 1092, 580

HR-MS (ESI-MS) m/z: [M+Na]+ Calc'd for $C_{28}H_{30}ClNO_3SNa$ 518.1533; Found 518.1528.

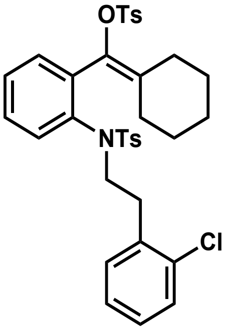

(2-((*N*-(2-chlorophenethyl)-4-methylphenyl)sulfonamido)phenyl)(cyclohexylidene)methyl-4-methylbenzenesulfonate (1).

Synthesized according to general procedure 4 starting from the corresponding ketone **SI-8** (1.47 g, 2.96 mmol). Crude product was purified via flash column chromatography using benzene to give vinyl tosylate **1** as a white solid (0.66 g, 34% yield).

*^{1}H NMR had poor resolution at room temperature, so ^{1}H NMRs are reported below at 70 °C.

^{1}H NMR (500 MHz, $CDCl_3$, 70 °C) δ 7.71 (d, J = 7.8 Hz, 2H), 7.57 (d, J = 7.8 Hz, 2H), 7.44 (br s, 1H), 7.31 (m, 2H), 7.25 (m, 3H), 7.09 (m, 2H), 7.04 (d, J = 7.8 Hz, 3H), 6.97 (m, 1H), 3.60 (m, 1H), 3.44 (m, 1H), 2.79 (m, 1H), 2.59 (m, 1H), 2.49 (m, 1H), 2.43 (s, 3H), 2.32 (m, 1H), 2.26 (s, 3H), 2.10 (m, 2H), 1.68 (br s, 2H), 1.59 (br s, 2H), 1.51 (br s, 2H).

^{13}C NMR (126 MHz, $CDCl_3$) δ 144.4, 143.4, 139.0, 136.8, 136.39, 136.36, 135.6, 134.5, 134.1, 133.6, 130.9, 129.4, 129.3, 128.4, 128.0, 127.79, 127.76, 126.8, 50.8, 32.3, 30.6, 28.7, 27.0, 26.7, 26.2, 21.4, 21.3.

FTIR (Neat film NaCl): 3066, 2973, 2928, 2855, 1597, 1475, 1444, 1356, 1176, 1160, 656, 571, 552.

HR-MS (ESI-MS) m/z: [M+Na]+ Calc'd for $C_{35}H_{36}ClNO_5S_2Na$ 672.1621; Found 672.1607.

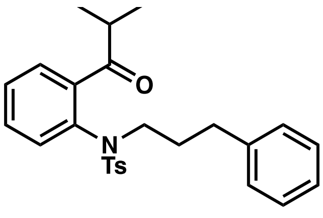

***N*-(2-isobutyrylphenyl)-4-methyl-*N*-(3-phenylpropyl)benzenesulfonamide (SI-10).**

Synthesized according to general procedure 3 starting from the corresponding sulfonamide **SI-5** (2.00 g, 0.00630 mol) and (3-iodopropyl)benzene (2.32 g ,0.00945 mol). Crude product was purified *via* flash column chromatography using 30% ether in hexanes to give sulfonamide **SI-10** as a white solid (2.60 g, 95% yield).

*NMR had poor resolution at room temperature, so NMRs are reported below at 70 °C.

^{1}H NMR (500 MHz, $CDCl_3$, 70 °C) δ 7.55 (d, *J* = 7.5 Hz, 1H), 7.49 (s, 2H), 7.35 (t, *J* = 7.5 Hz, 1H), 7.30 (t, *J* = 7.6 Hz, 1H), 7.23 (d, *J* = 7.6 Hz, 4H), 7.15 (t, *J* = 7.4 Hz, 1H), 7.09 (d, *J* = 7.3 Hz, 2H), 6.79 (s, 1H), 3.64 – 3.55 (m, 3H), 2.59 (t, *J* = 7.5 Hz, 2H), 2.41 (s, 3H), 1.98 – 1.85 (m, 2H), 1.24 (s, 3H), 1.23 (s, 3H).
^{13}C NMR (126 MHz, $CDCl_3$, 70 °C) δ 207.4, 143.5, 141.7, 141.0, 137.2, 135.8, 130.5, 129.3, 129.2, 128.3, 128.2, 128.0, 127.9, 125.9, 51.6, 39.3, 33.1, 29.5, 21.3, 18.8.
FTIR (Neat film NaCl): 3063, 3027, 2971, 2932, 2871, 1694, 1596, 1348, 1161, 980, 700, 658, 576.

HR-MS (CI-MS) m/z: [M+H]+ Calc'd for $C_{26}H_{30}NO_3S$ 436.1946; Found 436.1946.

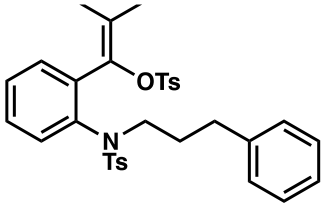

2-methyl-1-(2-((4-methyl-*N*-(3-phenylpropyl)phenyl)sulfonamido)phenyl)prop-1-en-1-yl-4-methylbenzenesulfonate (SI-11).

Synthesized according to general procedure 4 starting from the corresponding ketone **SI-10** (2.60 g, 0.00597 mol). Crude product was purified *via* flash column chromatography using 25% diethyl ether in hexanes to give vinyl tosylate **SI-11** as a yellow solid (1.10 g, 31% yield).

^{1}H NMR (400 MHz, $CDCl_3$) δ 7.53 (app d, *J* = 8.2 Hz, 4H), 7.40 (s, 1H), 7.31 – 7.21 (m, 6H), 7.20 – 7.15 (m, 1H), 7.12 (d, *J* = 8.1 Hz, 2H), 7.03 (d, *J* = 6.9 Hz, 2H), 6.92 (s, 1H), 3.41 – 3.18 (m, 2H), 2.42 (s, 3H), 2.36 (t, *J* = 7.6 Hz, 2H), 2.33 (s, 3H), 1.80 (s, 3H), 1.76 – 1.65 (m, 2H), 1.60 (s, 3H).
^{13}C NMR (101 MHz, $CDCl_3$) δ 144. 5, 143.3, 140.9, 138.9, 138.0, 136.4, 134.1, 133.6, 129.9, 129.6, 129.3, 129.28, 129.26, 128.23, 128.21, 127.96, 127.91, 127.7, 125.8, 51.0, 32.9, 29.1, 21.5, 21.4, 20.3, 18.6.
FTIR (Neat film NaCl): 3063, 3027, 2971, 2932, 2871, 1694, 1596, 1495, 1348, 1161, 980, 700, 658, 576.

HR-MS (ESI-MS) m/z: [M+H]+ Calc'd for $C_{33}H_{36}NO_5S_2$ 590.2035; Found 590.2061.

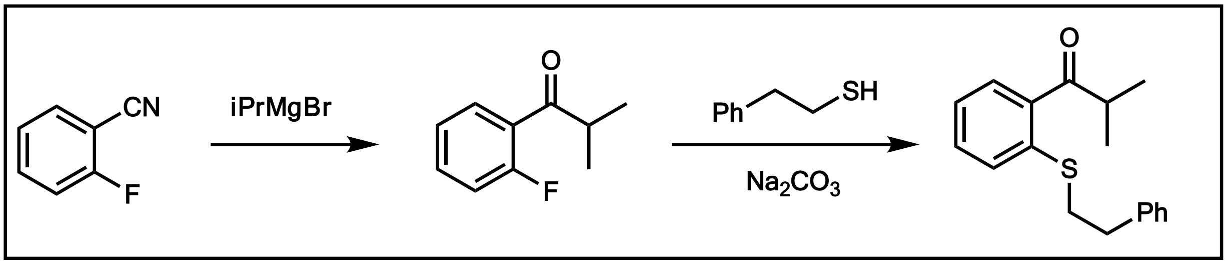

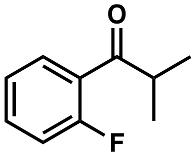

1-(2-fluorophenyl)-2-methylpropan-1-one (SI-12).

Magnesium (0.602 g, 24.8 mmol) was put into a flame-dried three-neck flask equipped with a condenser. THF (24 mL) was then added into the flask. 2-bromopropane (3.02 g, 2.33 mL, 24.8 mmol) was added slowly into the flask to keep the solution under gentle reflux. After the formation of the Grignard reagent, cooled the solution down to 0 °C and 2-fluorobenzonitrile (2.21 g, 20.6 mmol) in THF (20 mL) was added dropwise. The reaction was run overnight. After this the reaction was quenched with water and concentrated hydrochloric acid to make the pH down to 1. Then it was extracted with ethyl ether three times. The combined organic phase was washed with saturated sodium bicarbonate solution and brine. It was dried with magnesium sulfate, filtered, and concentrated to give the crude product. The crude product was purified via flash column chromatography using 2% ethyl acetate in hexanes to give the product as an oil (1.35 g, 39.3% yield).

^{1}H NMR data matches previous report[8].

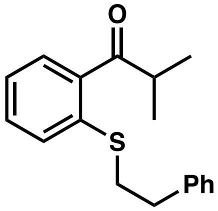

2-methyl-1-(2-(phenethylthio)phenyl)propan-1-one (SI-13).

2-phenylethane-1-thiol (1.96 g, 1.91 mL, 14.2 mmol), 1-(2-fluorophenyl)-2-methylpropan-1-one (**SI-12**) (1.18 g, 7.10 mmol), sodium carbonate (3.01 g, 28.4 mmol) and DMF (7 mL) was added to a flask. The solution was heated at 100 °C in the silicone oil bath overnight. After this, ethyl acetate and water was added into the solution. After the separation, the organic phase was washed with water three times and then washed with brine. The organic phase was dried with sodium sulfate and concentrated to give the crude product. The crude product was purified with flash column chromatography with 2% ethyl acetate in hexanes to give the product **SI-13** as a pale-yellow oil (0.53 g, 26% yield).

^{1}H NMR (400 MHz, $CDCl_3$) δ 7.61 (d, J = 7.7 Hz, 1H), 7.41 (m, 2H), 7.30 (m, 2H), 7.22 (m, 4H), 3.44 (sept, J = 6.8 Hz, 1H), 3.15 (m, 2H), 2.94 (m, 2H), 1.20 (d, J = 6.9 Hz, 6H).

^{13}C NMR (101 MHz, $CDCl_3$) δ 207.3, 140.3, 138.3, 137.9, 131.2, 128.9, 128.6, 128.5, 128.1, 126.5, 124.8, 38.2, 35.0, 34.9, 18.8.

FTIR (Neat film NaCl): 3061, 3027, 2969, 2930, 2870, 1691, 1585, 1454, 1431, 1214, 1075, 974, 738, 697.

HR-MS (CI-MS) m/z: [M+H]+ Calc'd for C18H21OS 285.1313; Found 285.1323.

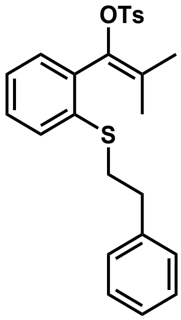

2-methyl-1-(2-(phenethylthio)phenyl)prop-1-en-1-yl 4-methylbenzenesulfonate (SI-14).

Synthesized according to general procedure 5 starting from the corresponding ketone **SI-13** (0.34 g, 0.0012 mol). Crude product was purified via flash column chromatography using 40% ether in hexanes to give vinyl tosylate **SI-14** as a pale yellow oil (0.29 g, 55% yield).

^{1}H NMR (400 MHz, $CDCl_3$) δ 7.46 (d, *J* = 8.4 Hz, 2H), 7.29 (m, 2H), 7.23 (m, 2H), 7.16 (m, 3H), 7.07 (m, 2H), 7.02 (d, *J* = 7.7 Hz, 2H), 2.97 (m, 2H), 2.79 (m, 2H), 2.29 (s, 3H), 1.92 (s, 3H), 1.60 (s, 3H).

^{13}C NMR (101 MHz, $CDCl_3$) δ 143.9, 140.3, 139.6, 137.7, 134.4, 133.5, 132.6, 129.1, 129.0, 128.6, 128.4, 128.2, 127.8, 127.3, 126.5, 124.8, 35.4, 34.2, 21.5, 19.9, 18.4.

FTIR (Neat film NaCl): 3062, 3032, 2918, 2856, 1598, 1496, 1454, 1364, 1176, 1086, 1071, 990, 823, 809, 792.

HR-MS (ESI-MS) m/z: [M+Na]+ Calc'd for C25H26O3S2Na 461.1221; Found 461.1209.

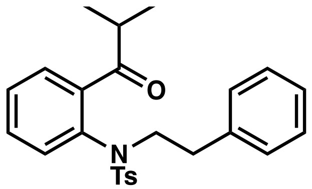

***N*-(2-isobutyrylphenyl)-4-methyl-*N*-phenethylbenzenesulfonamide (SI-15).**

Synthesized according to general procedure 3 starting from the corresponding sulfonamide **SI-5** (2.00 g, 0.0063 mol) and (2-iodoethyl)benzene (2.19 g. 0.00945 mol). Crude product was purified *via* flash column chromatography using 30% ether in hexanes to give sulfonamide **SI-15** as a white solid (1.70 g, 64% yield).

*NMR had poor resolution at room temperature, so NMRs are reported below at 70 °C.

^{1}H NMR (500 MHz, $CDCl_3$, 70 °C) δ 7.58 (d, *J* = 7.6 Hz, 1H), 7.53 (d, *J* = 7.8 Hz, 2H), 7.37 (t, *J* = 7.5 Hz, 1H), 7.33 (t, *J* = 7.7 Hz, 1H), 7.23 (d, *J* = 7.3 Hz, 4H), 7.17 (d, *J* = 7.3 Hz, 1H), 7.09 (d,

J = 7.4 Hz, 2H), 6.85 (d, J = 7.7 Hz, 1H), 3.82 (s, 2H), 3.58 (sept, J = 6.9 Hz, 1H), 2.90 (s, 2H), 2.40 (s, 3H), 1.24 (s, 3H), 1.23 (s, 3H).
^{13}C NMR (126 MHz, $CDCl_3$, 70 °C) δ 207.2, 143.6, 141.6, 138.2, 137.0, 135.7, 130.6, 129.3, 129.2, 128.5, 128.4, 128.0, 127.9, 126.4, 53.3, 39.2, 34.8, 31.4, 18.8.
FTIR (Neat film NaCl): 2924, 1693, 1596, 1455, 1349, 1163, 1093, 1056, 1033, 1017, 815, 688, 579.

HR-MS (CI-MS) m/z: [M+H]+ Calc'd for $C_{25}H_{28}NO_3S$ 422.1790; Found 422.1790.

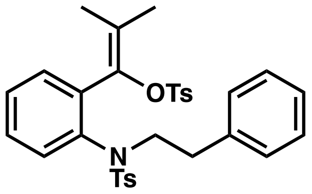

2-methyl-1-(2-((4-methyl-*N*-phenethylphenyl)sulfonamido)phenyl)prop-1-en-1-yl-4-methylbenzenesulfonate (SI-16).

Synthesized according to general procedure 4 starting from the corresponding ketone **SI-15** (1.70 g, 0.00403 mol). Crude product was purified *via* flash column chromatography using 25% diethyl ether in hexanes to give vinyl tosylate **SI-16** as a yellow solid (0.76 g, 33% yield).

^{1}H NMR (400 MHz, $CDCl_3$) δ 7.65 – 7.59 (m, 2H), 7.56 (d, J = 8.0 Hz, 2H), 7.51 – 7.44 (m, 1H), 7.38 – 7.29 (m, 2H), 7.29 – 7.23 (m, 2H), 7.23 – 7.14 (m, 3H), 7.07 (d, J = 8.1 Hz, 2H), 6.93 (d, J = 6.5 Hz, 3H), 3.60 (d, J = 10.9 Hz, 1H), 3.36 (td, J = 13.0, 4.9 Hz, 1H), 2.73 (td, J = 12.7, 5.3 Hz, 1H), 2.48 (td, J = 12.7, 4.8 Hz, 1H), 2.42 (s, 3H), 2.29 (s, 3H), 1.81 (s, 3H), 1.65 (s, 3H).

^{13}C NMR (101 MHz, $CDCl_3$) δ 144.5, 143.5, 139.0, 138.5, 137.9, 136.1, 134.1, 133.8, 130.2, 129.5, 129.4, 129.1, 128.6, 128.3, 128.2, 127.9, 126.3, 53.1, 34.5, 21.6, 21.5, 20.4, 18.69.

FTIR (Neat film NaCl): 3064, 3028, 2921, 1598, 1487, 1446, 1352, 1305, 1190, 1177, 1161, 1093, 1083, 814.

HR-MS (ESI-MS) m/z: [M+Na]+ Calc'd for $C_{32}H_{33}NO_5S_2Na$ 598.1698; Found 598.1689.

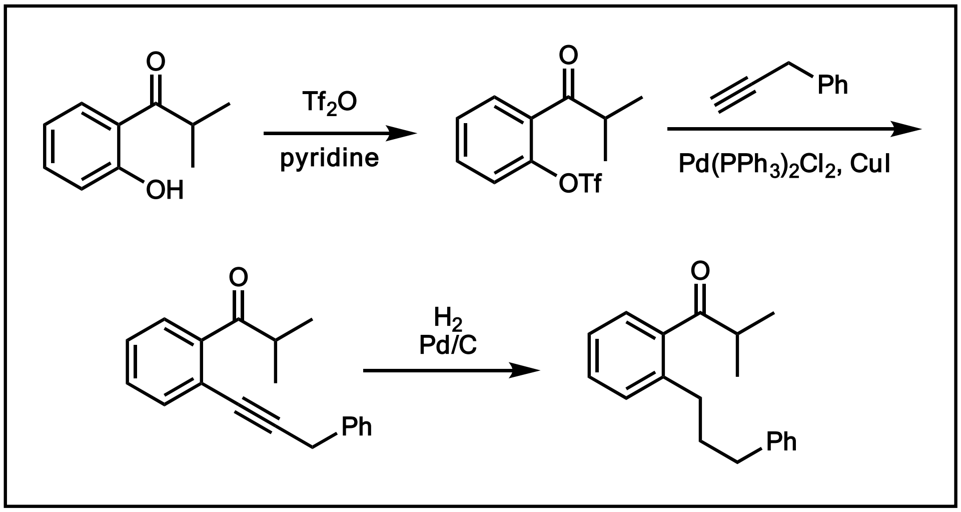

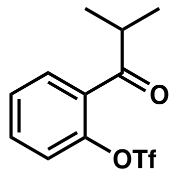

2-isobutyrylphenyl trifluoromethanesulfonate (SI-17).

1-(2-Hydroxyphenyl)-2-methylpropan-1-one (9.60 g, 58.5 mmol, 1.0 equiv) was dissolved in pyridine (56.9 g, 58.0 mL, 720 mmol, 12.0 equiv) and cooled to 0 °C. Triflic anhydride (19.8 g, 70.2 mmol, 1.2 equiv) was added dropwise. The reaction was warmed up to rt and stirred for 12h. Ethyl acetate (150 mL) was added to the reaction it was washed with aqueous 1M $CuSO_4$ (50 mL x 4) and brine (100 mL x 1). The organic layer was dried over $MgSO_4$, filtered and concentrated to give crude aryl triflate. Crude material was purified by silica flash column chromatography using 5% ethyl acetate in hexanes to give pure aryl triflate as yellow oil **SI-17** (12.9 g, 75% yield). Spectral data match those reported in the literature.[14]

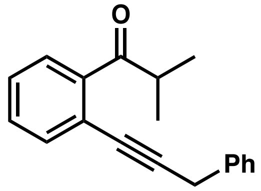

2-methyl-1-(2-(3-phenylprop-1-yn-1-yl)phenyl)propan-1-one (SI-18).

$Ph(PPh_3)_2Cl_2$ (237 mg, 0.337 mmol) and CuI (64.2 mg, 0.337 mmol) were added into a flame-dried Schlenk flask. Triethylamine (46 mL) and 2-isobutyrylphenyl trifluoromethanesulfonates (**SI-17**) (2.00 g, 6.74 mmol) were then added under nitrogen atmosphere. And at last prop-2-yn-1-ylbenzene (2.34 g, 2.52 mL, 20.2 mmol) was added and the reaction was heated at 70 °C in the silicone oil bath overnight. The reaction was cooled down to room temperature and quenched with saturated ammonium chloride solution. The mixture was extracted with ethyl acetate three times, and the combined organic phase was washed with water two times and brine. It was dried with magnesium sulfate, filtered and concentrated to give the crude product. The crude product was purified via flash column chromatography using 10% ethyl ether in hexanes to give the product **SI-18** as an oil (0.62 g, 35% yield).

^1H NMR (400 MHz, $CDCl_3$) δ 7.51 (dd, J = 7.5, 1.4 Hz, 1H), 7.46 (dd, J = 7.5, 1.4 Hz, 1H), 7.37 (m, 6H), 7.26 (tt, J = 7.8, 1.6 Hz, 1H), 3.85 (s, 2H), 3.62 (sept, J = 6.9 Hz, 1H), 1.12 (d, J = 6.8 Hz, 6H).

^{13}C NMR (101 MHz, $CDCl_3$) δ 208.9, 142.2, 136.3, 133.5, 130.2, 128.6, 128.0, 127.8, 127.6, 126.8, 121.2, 92.7, 81.0, 39.1, 26.0, 18.5.

FTIR (Neat film NaCl): 3386, 3063, 3030, 2972, 2932, 2873, 2199, 1768, 1690, 1593, 1454, 1214, 980, 757, 698.

HR-MS (ESI-MS) m/z: [M+Na]+ Calc'd for $C_{19}H_{18}ONa$ 285.1255; Found 285.1243.

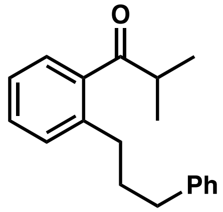

2-methyl-1-(2-(3-phenylpropyl)phenyl)propan-1-one (SI-19).

Alkyne **SI-18** (0.750 g, 2.86 mmol) was dissolved in ethanol (50 mL). Pd/C (0.103 g, 0.0972 mmol, 10% Pd) was then added. Hydrogen gas was blown into the solution for a while and the reaction was run under hydrogen atmosphere (1 atm) overnight. The reaction solution was filtered through celite and concentrated to give the crude product. The crude product was purified by flash chromatography with 4% ether in hexanes to give the product **SI-19** (0.63 g, 83% yield).

^{1}H NMR (400 MHz, $CDCl_3$) δ 7.48 (d, *J* = 7.8 Hz, 1H), 7.36 (t, *J* = 7.0 Hz, 1H), 7.31-7.21 (m, 4H), 7.21-7.14 (m, 3H), 3.30 (sept, *J* = 6.9 Hz, 1H), 2.76 (m, 2H), 2.68 (t, *J* = 7.8 Hz, 2H), 1.93 (m, 2H), 1.16 (d, *J* = 6.9 Hz, 6H).

^{13}C NMR (101 MHz, $CDCl_3$) δ 209.6, 142.3, 141.7, 138.8, 130.7, 130.5, 128.5, 128.3, 127.4, 125.8, 125.7, 39.1, 35.9, 33.5, 33.4, 18.7.

FTIR (Neat film NaCl): 3062, 3026, 2969, 2931, 2869, 1686, 1454, 1221, 976, 745, 633.

HR-MS (ESI-MS) m/z: [M+H]+ Calc'd for $C_{19}H_{23}O$ 267.1749; Found 267.1750.

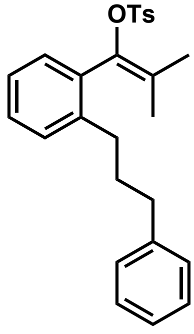

2-methyl-1-(2-(3-phenylpropyl)phenyl)prop-1-en-1-yl 4-methylbenzenesulfonate (SI-20).

Synthesized according to general procedure 5 starting from the corresponding ketone **SI-19** (0.64 g, 0.0024 mol). Crude product was purified via flash column chromatography using 20% ethyl acetate in hexanes to give vinyl tosylate **SI-20** as a white solid (0.32 g, 32% yield).

^{1}H NMR (400 MHz, $CDCl_3$) δ 7.33 (d, *J* = 8.4 Hz, 2H), 7.27 (t, *J* = 7.4 Hz, 2H), 7.19 (m, 2H), 7.15 (m, 1H), 7.11 (d, *J* = 6.8 Hz, 2H), 7.07 (t, *J* = 7.4 Hz, 1H), 6.98 (d, *J* = 8.2 Hz, 2H), 6.97 (t, *J* = 6.2 Hz, 1H), 2.57 (m, 1H), 2.53 (t, *J* = 7.7 Hz, 2H), 2.34 (m, 1H), 2.29 (s, 3H), 1.93 (s, 3H), 1.68 (quint, *J* = 8.0 Hz, 2H), 1.57 (s, 3H).

^{13}C NMR (101 MHz, $CDCl_3$) δ 143.9, 142.3, 142.0, 140.9, 134.5, 132.6, 132.3, 129.1, 128.8, 128.4, 128.3, 127.6, 127.0, 125.7, 125.2, 35.7, 32.6, 32.0, 21.5, 19.9, 18.4.

FTIR (Neat film NaCl): 3065, 3026, 2922, 2859, 1599, 1496, 1453, 1367, 1081, 990, 823, 810.

HR-MS (ESI-MS) m/z: [M+Na]+ Calc'd for $C_{26}H_{28}O_3SNa$ 443.1657; Found 443.1649.

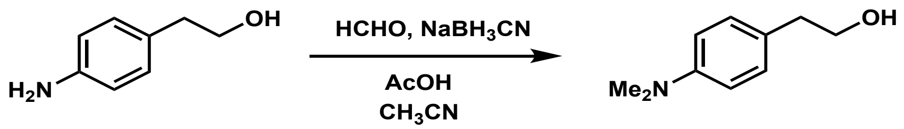

2-(4-(dimethylamino)phenyl)ethan-1-ol (SI-21).

2-(4-aminophenyl)ethan-1-ol (2.06 g, 15.0 mmol) in acetonitrile (150 mL) was added formaldehyde (37% in water, 11.2 mL, 150 mmol), sodium cyanoborohydride (2.83 g, 45.0 mmol) and acetic acid (0.987 mL, 17.2 mmol). After 3 h, the reaction was basified to pH 7-8 with saturated sodium bicarbonate solution. The mixture was extracted with ethyl acetate three times. The combined organic phase was washed with brine, dried with magnesium sulfate and concentrated to give the crude product. The crude product was purified *via* flash column chromatography using 20% methanol in ethyl ether with 5% triethylamine to give the product **SI-21** as yellow oil (2.43 g, 98.0% yield).

^{1}H NMR data matches previous report[9].

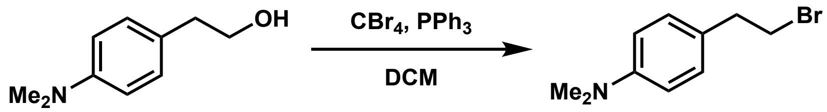

4-(2-bromoethyl)-*N*,*N*-dimethylaniline (SI-22).

2-(4-(dimethylamino)phenyl)ethan-1-ol **SI-21** (1.65 g, 10.0 mmol) and carbon tetrabromide (4.97 g, 15.0 mmmol) were dissolved in DCM (30 mL). It was cooled to 0 °C, and a solution of triphenylphosphine (2.62 g, 10.0 mmol) in DCM (20 mL) was added into it. The reaction was warmed to room temperature and run overnight. It was washed with water, and after separation the aqueous phase was extracted with DCM two times. The combined organic phase was dried with magnesium sulfate and then filtered. It was concentrated to give the crude product. The crude product was purified *via* flash column chromatography using 11% ethyl ether in hexanes with 5% triethylamine to give bromide **SI-22** as white solid (1.10 g, 48.2% yield).

^{1}H NMR data matches previous report[10].

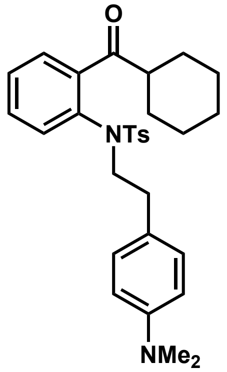

N-(2-(cyclohexanecarbonyl)phenyl)-N-(4-(dimethylamino)phenethyl)-4-methylbenzene sulfonamide (SI-23).

Synthesized according to general procedure 3 starting from the corresponding sulfonamide **SI-4** (0.86 g, 0.0024 mol) and 4-(2-bromoethyl)-N,N-dimethylaniline **SI-22** (1.09 g, 0.00482 mol).

Crude product was purified via flash column chromatography using 20% ethyl acetate in hexanes to give sulfonamide **SI-23** as a white powder (0.50 g, 41% yield).

*NMR had poor resolution at room temperature, so ^{1}H NMR is reported below at 60 °C.

^{1}H NMR (500 MHz, $CDCl_3$, 60 °C) δ 7.56 (d, *J* = 7.6 Hz, 1H), 7.52 (d, *J* = 8.0 Hz, 2H), 7.36 (dd, *J* = 7.4, 7.4 Hz, 1H), 7.32 (dd, *J* = 7.8, 7.8 Hz, 1H), 7.23 (d, *J* = 8.0 Hz, 2H), 6.96 (d, *J* = 8.2 Hz, 2H), 6.80 (d, *J* = 7.9 Hz, 1H), 6.66 (d, *J* = 8.1 Hz, 2H), 3.73 (br s, 2H), 3.40 (tt, *J* = 11.2, 3.4 Hz, 1H), 2.90 (s, 6H), 2.77 (m, 2H), 2.41 (s, 3H), 2.01 (br s, 2H), 1.83 (d, *J* = 12.6 Hz, 2H), 1.70 (d, *J* = 12.5 Hz, 1H), 1.46 (br s, 2H), 1.37 (m, 2H), 1.28 (m, 1H).

^{13}C NMR (126 MHz, $CDCl_3$) δ 207.4, 149.4, 143.7, 141.9, 136.8, 134.7, 130.7, 129.7, 129.4, 129.4, 128.5, 128.1, 128.1, 127.1, 113.0, 53.4, 49.2, 40.8, 33.8, 29.7, 28.6, 26.0, 21.6.

FTIR (Neat film NaCl): 2925, 2854, 1691, 1522, 1350, 1163, 1033, 577

HR-MS (ESI-MS) m/z: [M+Na]+ Calc'd for $C_{30}H_{36}N_2O_3SNa$ 527.2344; Found 527.2333.

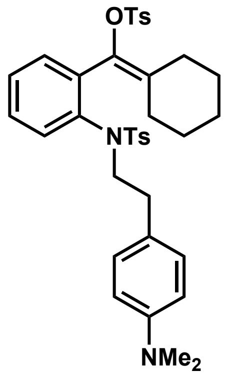

cyclohexylidene(2-((*N*-(4-(dimethylamino)phenethyl)-4-methylphenyl)sulfonamido)phenyl) methyl 4-methylbenzenesulfonate (SI-24).

Synthesized according to general procedure 5 starting from the corresponding ketone **SI-23** (0.34 g, 0.00067 mol). Crude product was purified via flash column chromatography using 50% ether in hexanes with 5% triethylamine to give vinyl tosylate **SI-24** as a white solid (0.18 g, 41% yield).

*NMR had poor resolution at room temperature, so ^{1}H NMR is reported below at 70 °C.

^{1}H NMR (500 MHz, $CDCl_3$, 70 °C) δ 7.69 (d, *J* = 7.7 Hz, 2H), 7.57 (d, *J* = 7.8 Hz, 2H), 7.43 (br s, 1H), 7.29 (m, 2H), 7.26 (d, *J* = 7.6 Hz, 2H), 7.08 (d, *J* = 7.9 Hz, 2H), 6.98 (d, *J* = 6.6 Hz, 1H), 6.79 (d, *J* = 8.0 Hz, 2H), 6.62 (d, *J* = 8.0 Hz, 2H), 3.56 (m, 1H), 3.39 (m, 1H), 2.90 (s, 6H), 2.61 (m, 1H), 2.49 (m, 1H), 2.43 (s, 3H), 2.40 (m, 1H), 2.31 (s, 3H), 2.28 (m, 1H), 2.11 (m, 1H), 2.04 (m, 1H), 1.69 (br s, 2H), 1.59 (br s, 2H), 1.50 (m, 2H).

^{13}C NMR (126 MHz, $CDCl_3$) δ 149.3, 144.2, 143.4, 139.2, 136.7, 136.2, 135.5, 134.2, 133.6, 129.4, 129.4, 129.4, 129.3, 128.7, 128.3, 128.1, 127.8, 126.4, 125.5, 112.8, 53.2, 40.8, 33.4, 30.6, 28.7, 27.0, 26.7, 26.2, 21.6, 21.6.

FTIR (Neat film NaCl): 3032, 2929, 2857, 1616, 1597, 1522, 1445, 1352, 1188, 1176, 1161, 1093, 807, 789, 572, 555.

HR-MS (ESI-MS) m/z: [M+H]+ Calc'd for $C_{37}H_{43}N_2O_5S_2$ 659.2614; Found 659.2619.

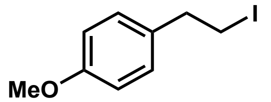

1-(2-iodoethyl)-4-methoxybenzene (SI-25).

Iodine (3.96 g, 15.6 mmol) and triphenylphosphine (4.09g, 15.6 mmol) was added to DCM (24 mL). To the solution was added imidazole (1.15 g, 16.9 mmol) in DCM (10 mL) and stirred together for 15 min. Then the alcohol (1.98 g, 13.0 mmol) in DCM (5 mL) was added and it was stirred overnight. The reaction was washed with sodium thiosulfate solution. And the organic phase was dried with magnesium sulfate and filtered. The solution was then concentrated to give the crude product. The crude product was purified by flash column chromatography using 5% ethyl ether in hexanes to give the product as a white solid (3.15 g, 92.5% yield).

^{1}H NMR data matches previous report[11].

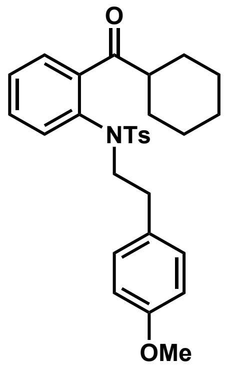

***N*-(2-(cyclohexanecarbonyl)phenyl)-*N*-(4-methoxyphenethyl)-4-methylbenzenesulfonamide (SI-26).**

Synthesized according to general procedure 3 starting from the corresponding sulfonamide **SI-4** (1.02 g, 0.00286 mol) and 1-(2-iodoethyl)-4-methoxybenzene **SI-25** (1.50 g, 0.00572 mol). Crude product was purified via flash column chromatography using 40% ether in hexanes to give sulfonamide **SI-26** as a white powder (1.22 g, 86.7% yield).

*NMR had poor resolution at room temperature, so NMRs are reported below at 70 °C.

^{1}H NMR (500 MHz, $CDCl_3$, 70 °C) δ 7.54 (m, 3H), 7.37 (t, *J* = 7.4 Hz, 1H), 7.32 (t, *J* = 7.8 Hz, 1H), 7.24 (d, *J* = 7.8 Hz, 2H), 6.99 (d, *J* = 7.4 Hz, 2H), 6.82 (d, *J* = 8.1 Hz, 1H), 6.78 (d, *J* = 6.9 Hz, 2H), 3.76 (s, 3H), 3.75 (br s, 2H), 3.36 (m, 1H), 2.81 (br s, 2H), 2.41 (s, 3H), 2.01 (br s, 2H), 1.83 (m, 2H), 1.69 (m, 1H), 1.36 (m, 5H).

^{13}C NMR (126 MHz, $CDCl_3$, 70 °C) δ 206.6, 158.4, 143.4, 141.9, 136.9, 136.0, 130.3, 129.4, 129.2, 129.1, 128.3, 127.9, 127.8, 114.1, 55.1, 53.6, 49.2, 33.9, 29.0, 25.9, 25.7, 21.2.

FTIR (Neat film NaCl): 3032, 2930, 2853, 1691, 1513, 1350, 1248, 1162, 578

HR-MS (ESI-MS) m/z: [M+H]+ Calc'd for $C_{29}H_{34}NO_4S$ 492.2209; Found 492.2229.

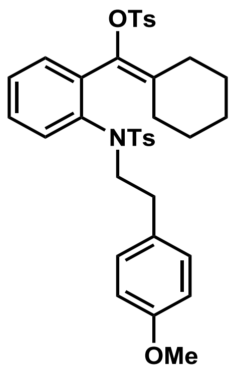

cyclohexylidene(2-((*N*-(4-methoxyphenethyl)-4-methylphenyl)sulfonamido)phenyl)methyl 4-methylbenzenesulfonate (SI-27).

Synthesized according to general procedure 5 starting from the corresponding ketone **SI-26** (0.58 g, 0.0012 mol). Crude product was purified via flash column chromatography using 40% ether in hexanes to give vinyl tosylate **SI-27** as a white solid (0.43 g, 56% yield).

*NMR had poor resolution at room temperature, so NMRs are reported below at 50 °C.

^{1}H NMR (500 MHz, $CDCl_3$, 50 °C) δ 7.67 (d, *J* = 7.9 Hz, 2H), 7.57 (d, *J* = 8.2 Hz, 2H), 7.43 (br s, 1H), 7.31 (m, 2H), 7.26 (d, *J* = 6.6 Hz, 2H), 7.09 (d, *J* = 8.0 Hz, 2H), 6.96 (br s, 1H), 6.83 (d, *J* = 8.6 Hz, 2H), 6.74 (d, *J* = 8.6 Hz, 2H), 3.76 (s, 3H), 3.59 (ddd, *J* = 13.2, 5.5, 5.5 Hz, 1H), 3.38 (ddd, *J* = 12.2, 5.0, 5.0 Hz, 1H), 2.66 (ddd, *J* = 12.8, 5.4, 5.4 Hz, 1H), 2.44 (m, 2H), 2.43 (s, 3H), 2.31 (s, 3H), 2.29 (m, 1H), 2.12 (m, 1H), 2.03 (m, 1H), 1.67 (m, 2H), 1.54 (m, 4H).

^{13}C NMR (126 MHz, $CDCl_3$, 50 °C) δ 158.1, 144.2, 143.2, 139.2, 136.6, 136.5, 135.5, 134.4, 133.5, 130.5, 129.4, 129.3, 129.2, 129.1, 128.1, 127.9, 127.6, 113.8, 55.1, 53.0, 33.5, 30.4, 28.5, 26.8, 26.6, 26.1, 21.3, 21.2.

FTIR (Neat film NaCl): 3029, 2971, 2928, 2855, 1611, 1597, 1512, 1364, 1175, 1157, 788, 656, 570, 552.

HR-MS (ESI-MS) m/z: [M+Na]+ Calc'd for $C_{36}H_{39}NO_6S_2Na$ 668.2117; Found 668.2093.

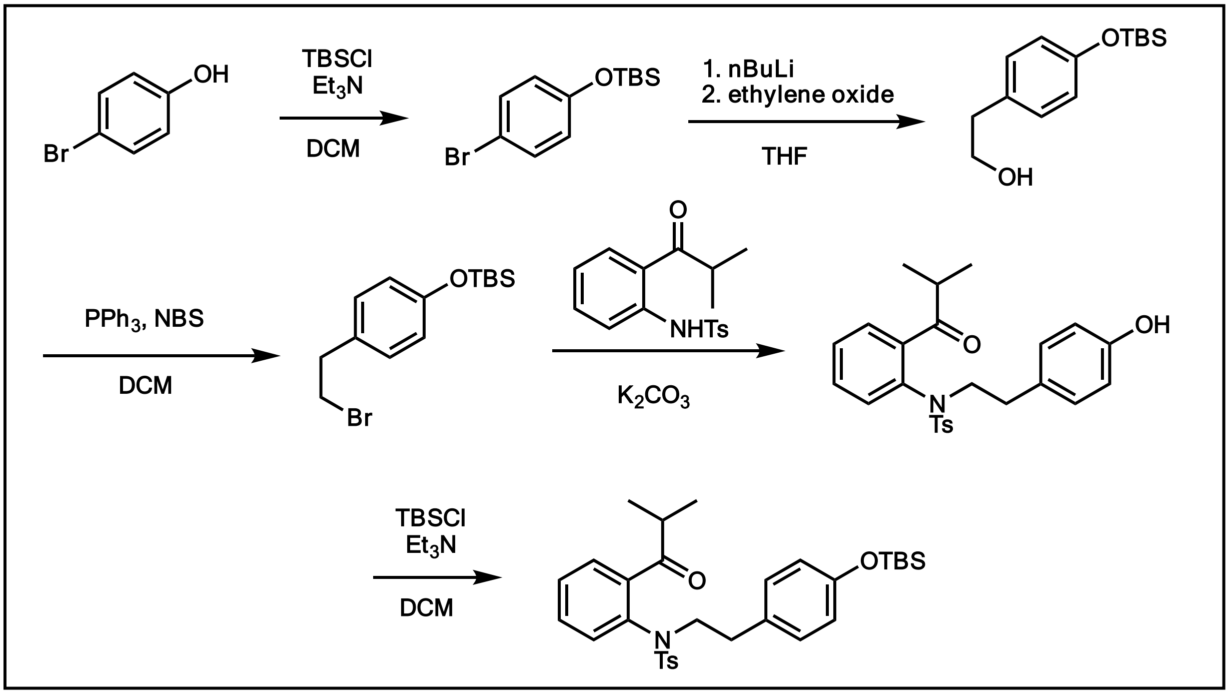

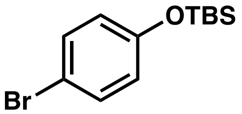

(4-bromophenoxy)(*tert*-butyl)dimethylsilane (SI-28).

In the DCM (50mL) solution of the 4-bromophenol (9.94 g, 57.8 mmol) and triethylamine (7.26 g, 71.7 mmol) was added the DCM (50mL) solution of *tert*-butyldimethylsilyl chloride (13.0 g, 86.7 mmol). After a day, the reaction was done. It was then diluted and washed with water 2 times until the aqueous phase was strongly acidic. The organic phase was then washed with sat. $NaHCO_3$, dried with $MgSO_4$, filtered and concentrated in vacuo. The crude product was purified by column chromatography with 2.4% diethyl ether in hexanes as a colorless liquid (15.95g, 96.1% yield).

^{1}H NMR matches previous report[12].

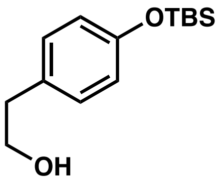

2-(4-((tert-butyldimethylsilyl)oxy)phenyl)ethan-1-ol (SI-29).

In a 3-neck flask equipped with a condenser was added **SI-28** (5.40 g, 18.8 mmol) and THF (38 mL). The solution was cooled down to –78°C. n-BuLi (2.06 M in hexanes, 10.0 mL, 20.7 mmol) was added dropwise, and the reaction was left at –78°C for 30 min after the addition. Then ethylene oxide (2.5 M in THF, 15.0 mL, 37.6 mmol) was added dropwise at –78°C. The reaction was warmed to 0°C for 30 min, and then it was heated at 40°C for 30 min, which was followed by

reflux for 1 h. After the reaction was complete, the solution was concentrated in vacuo and then diluted with DCM and washed with 1M HCl (40 mL). The aqueous phase was extracted with DCM 2 times. The combined organic phase was washed with sat. $NaHCO_3$, brine, dried with $MgSO_4$, filtered, and concentrated in vacuo. The crude product was purified by column chromatography with 50% hexanes and 50% diethyl ether to give the product **SI-29** as a pale-yellow oil (1.72 g, 36.2% yield).

^{1}H NMR matches previous report[12].

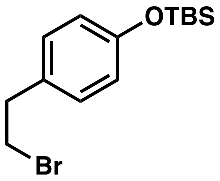

(4-(2-bromoethyl)phenoxy)(tert-butyl)dimethylsilane (SI-30).

SI-29 (1.72 g, 6.81 mmol) was dissolved in DCM (17 mL) and the solution was cooled down to 0°C. After 10 min, triphenylphosphine (2.14 g, 8.18 mmol) and *N*-bromosuccinimide (1.46 g, 8.18 mmol) was added into the solution. After 4 hours, the reaction was complete and quenched by sat. $NaHCO_3$. The solution was extracted with DCM 3 times. The combined organic phase was washed with brine, dried with $MgSO_4$, filtered, and concentrated in vacuo. The crude product was purified by column chromatography with 2.4% diethyl ether in hexanes to give the product **SI-30** as a colorless liquid (1.67 g, 77.7% yield).

^{1}H NMR (400 MHz, $CDCl_3$) δ 7.06 (d, *J* = 8.6 Hz, 2H), 6.78 (d, *J* = 8.5 Hz, 2H), 3.52 (t, *J* = 7.8 Hz, 2H), 3.09 (t, *J* = 7.8 Hz, 2H), 0.98 (s, 9H), 0.19 (s, 6H).

^{13}C NMR (101 MHz, $CDCl_3$) δ 153.5, 130.6, 128.6, 119.1, 37.7, 32.3, 24.6, 17.2, –5.5.

FTIR (Neat film NaCl): 2956, 2929, 2857, 1608, 1508, 1471, 1463, 1252, 911, 837, 779.

HR-MS (CI-MS) m/z: [M]+ Calc'd for $C_{14}H_{23}OSiBr$ 314.0701; Found 314.0708.

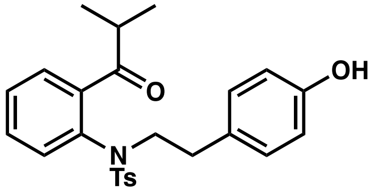

N-(4-hydroxyphenethyl)-N-(2-isobutyrylphenyl)-4-methylbenzenesulfonamide (SI-31).

Synthesized according to general procedure 3 starting from the corresponding sulfonamide **SI-5** (1.26 g, 0.00396 mol) and (4-(2-bromoethyl)phenoxy)(tert-butyl)dimethylsilane **SI-30** (1.49 g, 0.00476 mol). Crude product was purified via flash column chromatography using 25% acetone in hexanes to give sulfonamide **SI-31** as a white powder (0.63 g, 36% yield).

^{1}H NMR (400 MHz, $CDCl_3$) δ 7.59 (dd, *J* = 7.6, 1.8 Hz, 1H), 7.46 (d, *J* = 8.1 Hz, 2H), 7.40 (td, *J* = 7.5, 1.3 Hz, 1H), 7.34 (td, *J* = 7.6, 1.8 Hz, 1H), 7.23 (d, *J* = 8.0 Hz, 2H), 6.96 (d, *J* = 8.1Hz, 2H), 6.77 – 6.70 (m, 3H), 3.95 (br, 1H), 3.66 (hepta, *J* = 6.9 Hz, 1H), 3.46 (br, 1H), 2.88 (br, 1H), 2.62 (br, 1H), 2.41 (s, 3H), 1.36 – 1.06 (m, 6H).

^{13}C NMR (101 MHz, $CDCl_3$) δ 207.3, 153.3, 142.8, 140.5, 135.7, 133.7, 129.8, 129.0, 128.8, 128.7, 128.5, 127.2, 127.0, 126.4, 114.4, 52.2, 38.2, 32.8, 20.5, 18.2 (br), 17.6 (br)

FTIR (Neat film NaCl): 3377, 2973, 2931, 2871, 1690, 1614, 1595, 1516, 1444, 1347, 1214, 1159, 1090, 981, 815, 657, 577.

HR-MS (ESI-MS) m/z: [M+Na]+ Calc'd for $C_{25}H_{27}NO_4SNa$ 460.1535; Found 460.1558.

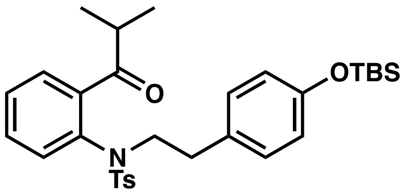

N-(4-((tert-butyldimethylsilyl)oxy)phenethyl)-N-(2-isobutyrylphenyl)-4-methylbenzenesulfonamide (SI-32).

SI-31 (0.51 g, 1.2 mmol) and triethylamine (0.16 mL, 1.2 mmol) were added in DCM (6 mL). *Tert*-butyldimethylsilyl chloride (0.18 g, 1.2 mmol) was also dissolved in DCM (6 mL) and the solution was added into the first solution slowly. Reaction was monitored by TLC and additional *tert*-butyldimethylsilyl chloride (0.09 g, 0.6 mmol) and triethylamine (0.08 mL, 0.6 mmol) were added each time until **SI-31** was completely consumed. The reaction was then diluted with DCM, washed with 1M HCl, saturated $NaHCO_3$ solution and brine in order. The organic phase was dried with $MgSO_4$, filtered, and concentrated in vacuo. Crude product was purified via column chromatography using 20% diethyl ether in hexanes to give sulfonamide **SI-32** as a yellow oil (0.53 g, 82% yield).

^{1}H NMR (400 MHz, $CDCl_3$) δ 7.59 (dd, J = 7.6, 1.8 Hz, 1H), 7.46 (m, 2H), 7.39 (td, J = 7.5, 1.3 Hz, 1H), 7.33 (td, J = 7.6, 1.8 Hz, 1H), 7.23 (d, J = 8.4 Hz, 2H), 6.94 (d, J = 8.6 Hz, 2H), 6.75 (dd, J = 7.9, 1.3 Hz, 1H), 6.71 (d, J = 8.6 Hz, 2H), 3.99 (br, 1H), 3.67 (hepta, J = 6.9 Hz, 1H), 3.48 (br, 1H), 2.88 (br, 1H), 2.62 (br, 1H), 2.41 (s, 3H), 1.33 – 1.09 (m, 6H), 0.96 (s, 9H), 0.17 (s, 6H).

^{13}C NMR (101 MHz, $CDCl_3$) δ 207.1, 153.3, 142.7, 140.5, 135.7, 133.8, 129.8, 129.6, 128.7, 128.6, 128.4, 127.2, 127.0, 126.4, 119.1, 52.1, 38.2, 32.9, 24.7, 20.5, 17.2, –5.5.

FTIR (Neat film NaCl): 2957, 2929, 2858, 1694, 1596, 1510, 1471, 1350, 1260, 1163, 914.

HR-MS (ESI-MS) m/z: [M+Na]+ Calc'd for $C_{31}H_{41}NO_4SSiNa$ 574.2423; Found 574.2432.

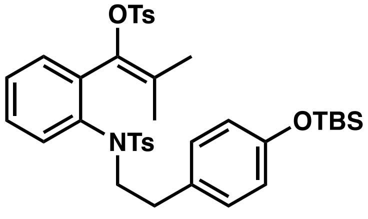

1-(2-((N-(4-((tert-butyldimethylsilyl)oxy)phenethyl)-4-methylphenyl)sulfonamido)phenyl)-2-methylprop-1-en-1-yl 4-methylbenzenesulfonate (SI-33).

Synthesized according to general procedure 6 starting from the corresponding ketone **SI-32** (0.43 g, 0.00078 mol). Crude product was purified via flash column chromatography using 14% acetone in hexanes to give vinyl tosylate **SI-33** as a white solid (0.49 g, 89% yield).

^{1}H NMR (400 MHz, $CDCl_3$) δ 7.62 (s, 2H), 7.55 (d, *J* = 8.0 Hz, 2H), 7.47 (s, 1H), 7.39 – 7.29 (m, 2H), 7.27 (s, 2H), 7.08 (d, *J* = 8.1 Hz, 2H), 6.91 (s, 1H), 6.78 – 6.73 (m, 2H), 6.69 – 6.65 (m, 2H), 3.56 (br s, 1H), 3.34 (td, *J* = 12.9, 4.8 Hz, 1H), 2.62 (td, *J* = 12.8, 5.4 Hz, 1H), 2.45–2.33 (m, 1H), 2.42 (s, 3H), 2.31 (s, 3H), 1.81 (s, 3H), 1.64 (s, 3H), 0.97 (s, 9H), 0.17 (d, *J* = 0.7 Hz, 6H).

^{13}C NMR (101 MHz, $CDCl_3$) δ 153.0, 143.5, 142.4, 138.0, 136.9, 135.2, 133.1, 132.8, 130.1, 129.2, 128.5, 128.43. 128.40, 127.2, 127.0, 118.8, 52.2, 32.6, 24.7, 20.53, 20.52 19.4, 17.7, 17.2, –5.4.

FTIR (Neat film NaCl): 2955, 2927, 2856, 1598, 1509, 1353, 1258, 1189, 1177, 1159, 1083, 912, 804, 573.

HR-MS (ESI-MS) m/z: [M+Na]+ Calc'd for $C_{38}H_{47}NO_6S_2SiNa$ 728.2512; Found 728.2511.

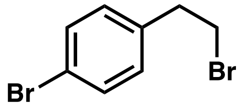

1-bromo-4-(2-bromoethyl)benzene (SI-34)

2-(4-bromophenyl)ethan-1-ol (2.01 g, 10.0 mmol) in DCM (25 mL) was cooled down to 0 °C. Triphenylphosphine (3.15 g, 12.0 mmol) and *N*-bromosuccinimide (2.14 g, 12.0 mmol) was then added into the solution and it was stirred overnight to room temperature. The reaction was quenched by saturated sodium bicarbonate solution. It was then separated, and the aqueous phase was extracted with DCM three times. The combined organic phase was dried with magnesium sulfate and concentrated to give the crude product. The crude product was purified via flash chromatography to give the product **SI-34** as a cloudy oil (2.45 g, 92.8% yield).

^{1}H NMR data matches previous report[13].

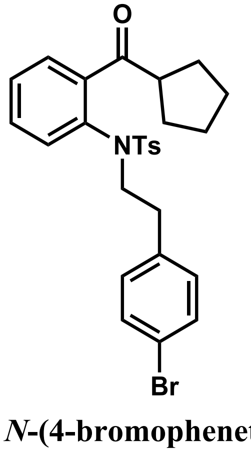

***N*-(4-bromophenethyl)-*N*-(2-(cyclopentanecarbonyl)phenyl)-4-methylbenzenesulfonamide (SI-35).**

Synthesized according to general procedure 3 starting from 2.91 mmol of the corresponding sulfonamide **SI-6** (1.00 g, 0.00291 mol) and 1-bromo-4-(2-bromoethyl)benzene **SI-34** (1.52 g, 0.00582 mol). Crude product was purified via flash column chromatography using 40% ether in hexanes to give sulfonamide **SI-35** as a white powder (1.06g, 69% yield).

*NMR had poor resolution at room temperature, so NMRs are reported below at 70 °C.

^{1}H NMR (500 MHz, $CDCl_3$, 70 °C) δ 7.59 (d, *J* = 7.6 Hz, 1H), 7.51 (d, *J* = 8.4 Hz, 2H), 7.38 (t, *J* = 7.5 Hz, 1H), 7.35 (d, *J* = 6.4 Hz, 2H), 7.33 (t, *J* = 7.6 Hz, 1H), 7.23 (d, *J* = 7.9 Hz, 2H), 6.96 (d, *J* = 7.8 Hz, 2H), 6.84 (d, *J* = 7.9 Hz, 1H), 3.77 (br s, 2H), 3.63 (quint, *J* = 8.0 Hz, 1H), 2.86 (br s, 2H), 2.42 (s, 3H), 1.93 (br s, 4H), 1.76 (m, 2H), 1.62 (m, 2H).

^{13}C NMR (126 MHz, $CDCl_3$, 70 °C) δ 206.2, 143.3, 142.3, 137.4, 136.7, 136.1, 131.4, 130.4, 130.2, 129.2, 129.0, 128.7, 127.9, 127.8, 120.2, 53.1, 50.3, 34.3, 30.3, 26.1, 21.2.

FTIR (Neat film NaCl): 3064, 3028, 2952, 2867, 1690, 1595, 1488, 1440, 1348, 1159, 572

HR-MS (EI-MS) m/z: [M+Na]+ Calc'd for $C_{27}H_{28}BrNO_3SNa$ 548.0871; Found 548.0877.

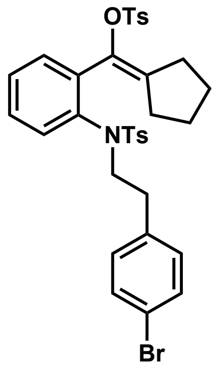

(2-((*N*-(4-bromophenethyl)-4-methylphenyl)sulfonamido)phenyl)(cyclopentylidene)methyl 4-methylbenzenesulfonate (SI-36).

Synthesized according to general procedure 5 starting from the corresponding ketone **SI-35** (0.92 g, 0.00175 mol). Crude product was purified via flash column chromatography using 40% ether in hexanes to give vinyl tosylate **SI-36** as a white solid (0.72 g, 61% yield).

*NMR had poor resolution at room temperature, so NMRs are reported below at 70 °C.

^{1}H NMR (500 MHz, $CDCl_3$, 70 °C) δ 7.63 (m, 4H), 7.48 (d, *J* = 7.4 Hz, 1H), 7.31 (m, 4H), 7.26 (d, *J* = 8.0 Hz, 2H), 7.13 (d, *J* = 7.8 Hz, 2H), 6.94 (d, *J* = 7.6 Hz, 1H), 6.83 (d, *J* = 7.8 Hz, 2H), 3.54 (m, 2H), 2.63 (m, 3H), 2.43 (s, 3H), 2.35 (s, 3H), 2.26 (m, 3H), 1.65 (br s, 4H).

^{13}C NMR (126 MHz, $CDCl_3$, 70 °C) δ 144.3, 143.3, 141.9, 138.5, 137.6, 136.6, 135.2, 133.2, 131.3, 130.3, 130.2, 130.1, 129.3, 129.2, 129.1, 129.0, 128.0, 128.0, 127.9, 120.0, 52.7, 33.9, 30.8, 30.1, 26.2, 25.8, 21.2, 21.2.

FTIR (Neat film NaCl): 3070, 3032, 2957, 2869, 1597, 1488, 1352, 1189, 1176, 1160, 806, 788, 659, 572, 553.

HR-MS (EI-MS) m/z: [M+Na]+ Calc'd for $C_{34}H_{34}BrNO_5S_2Na$ 702.0959; Found 702.0975.

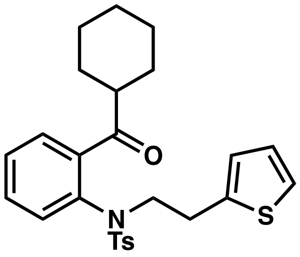

N-(2-(cyclohexanecarbonyl)phenyl)-4-methyl-N-(2-(thiophen-2-yl)ethyl)benzenesulfonamide (SI-37).

Synthesized according to general procedure 3 starting from the corresponding sulfonamide **SI-4** (1.50 g, 0.00420 mol) and 2-(2-iodoethyl)thiophene (1.50 g, 0.00630 mol). Crude product was purified *via* flash column chromatography using 15% ether in hexanes to give sulfonamide **SI-37** as a white solid (0.80 g, 40% yield).

^{1}H NMR (500 MHz, $CDCl_3$) δ 7.59 (dd, J = 7.7, 1.7 Hz, 1H), 7.48 (d, J = 8.3 Hz, 2H), 7.39 (td, J = 7.5, 1.2 Hz, 1H), 7.34 (td, J = 7.7, 1.7 Hz, 1H), 7.24 (d, J = 8.3 Hz, 2H), 7.10 (dd, J = 5.1, 1.2 Hz, 1H), 6.88 (dd, J = 5.1, 3.4 Hz, 1H), 6.76 – 6.73 (m, 2H), 4.04 (br s, 1H), 3.60 (br s, 1H), 3.35 (tt, J = 11.4, 3.4 Hz 1H), 3.25 (br s, 1H), 3.00 (br s, 1H), 2.41 (s, 3H), 2.10 (br s, 1H), 1.92 (br s, 1H), 1.87 – 1.77 (m, 2H), 1.69 (d, J = 12.5 Hz, 1H), 1.63 – 1.53 (m, 1H), 1.41 – 1.18 (m, 4H).
^{13}C NMR (126 MHz, $CDCl_3$) δ 206.8, 143.7, 141.4, 140.0, 136.5, 134.6, 130.8, 129.4, 129.3, 128.2, 127.8, 127.7, 126.8, 125.2, 123.7, 53.1, 49.0, 28.8, 25.8, 21.4.
FTIR (Neat film NaCl): 3068, 2929, 1854, 1690, 1596, 1444, 1350, 1162, 1092, 907, 728.

HR-MS (ESI-MS) m/z: [M+Na]+ Calc'd for $C_{26}H_{29}NO_3S_2Na$ 490.1487; Found 490.1496.

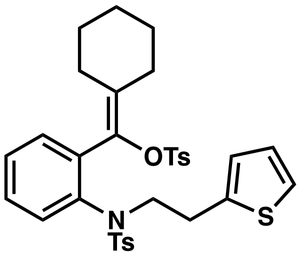

cyclohexylidene(2-((4-methyl-N-(2-(thiophen-2-yl)ethyl)phenyl)sulfonamido)phenyl)methyl 4-methylbenzenesulfonate (SI-38).

Synthesized according to general procedure 3 starting from the corresponding ketone **SI-37** (0.55 g, 0.0012 mol). Crude product was purified *via* flash column chromatography using 30% diethyl ether in hexanes to give vinyl tosylate **SI-38** as a yellow solid (0.49 g, 67% yield).

^{1}H NMR (500 MHz, $CDCl_3$, 55 °C) δ 7.67 (d, J = 7.9 Hz, 2H), 7.56 (d, J = 8.4 Hz, 2H), 7.55 (br s, 1H), 7.44 – 7.26 (m, 5H), 7.15 – 7.04 (m, 2H), 6.93 (br s, 1H), 6.87 – 6.85 (m, 1H), 6.60 (s, 1H), 3.65 (ddd, J = 13.7, 12.0, 5.3 Hz, 1H), 3.46 (ddd, J = 13.8, 12.0, 5.0 Hz, 1H), 2.95 (ddd, J = 14.5, 12.2, 5.3 Hz, 1H), 2.75 (ddd, J = 14.5, 11.9, 4.9 Hz, 1H), 2.51 – 2.40 (m, 1H), 2.43 (s, 3H), 2.32 – 2.25 (m, 1H), 2.31 (s, 3H), 2.20 – 2.00 (m, 2H), 1.80 – 1.66 (m, 2H), 1.66 – 1.50 (m, 4H).

^{13}C NMR (126 MHz, $CDCl_3$, 55 °C) δ 144.4, 143.6, 140.8, 139.3, 136.8, 136.5, 135.6, 134.5, 133.7, 129.5, 129.4, 129.2, 128.3, 128.0, 127.9, 126.8, 125.0, 123.5, 52.9, 30.6, 28.7, 27.0, 26.8, 26.3, 21.5.

FTIR (Neat film NaCl): 2930, 2925, 2856, 1492, 1356, 1175, 1093, 802, 573.

HR-MS (ESI-MS) m/z: $[M+NH_4]^+$ Calc'd for $C_{33}H_{39}N_2O_5S_3$ 639.2021; Found 639.2044.

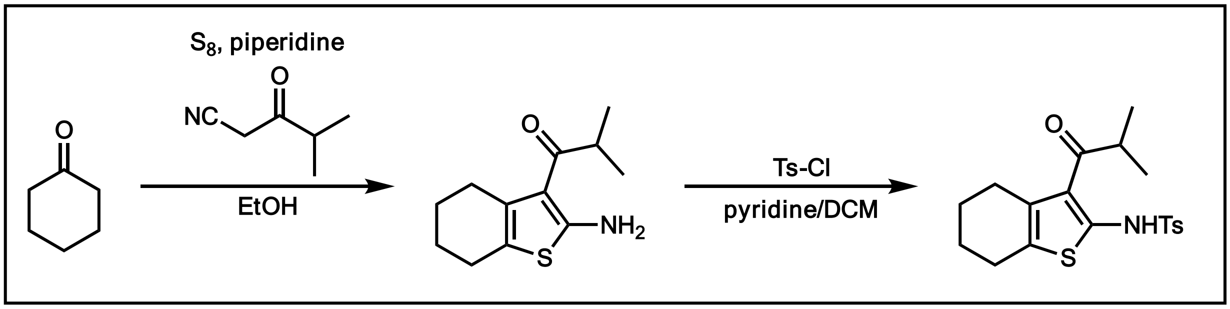

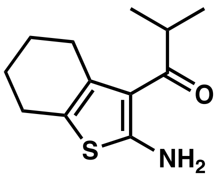

1-(2-amino-4,5,6,7-tetrahydrobenzo[*b*]thiophen-3-yl)-2-methylpropan-1-one (SI-39).

Cyclohexanone (1.25 g, 12.7 mmol, 1.0 equiv) was added to a 100 mL schlenk followed by *α*-cyano isopropyl ketone (1.70 g, 15.3 mmol, 1.2 equiv) and this was dissolved in ethanol (20 mL). To this solution was added S_8 (3.92 g, 15.3 mmol, 1.2 equiv) and piperidine (1.30 g, 15.3 mmol, 1.2 equiv). The reaction vessel was sealed and heated to 65 °C in the silicone oil bath for 48 hours. Upon completion, the reaction was cooled to r.t. and poured onto ice. After the ice melted, the resultant suspension was filtered and washed with water followed by pentane. The light yellow solid (2.54 g, ca. 89%) was dried under vacuum and carried forward to the next step without further purification.

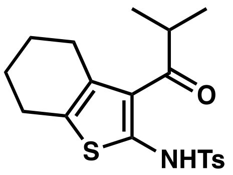

***N*-(3-isobutyryl-4,5,6,7-tetrahydrobenzo[*b*]thiophen-2-yl)-4-methylbenzenesulfonamide (SI-40).**

Synthesized from the crude material (2.54 g, ca. 11.3 mmol) from the previous step according to a slightly modified general procedure 2 using 26 equiv of DCM (18 mL) instead of 13 equiv. The crude product was purified by flash column chromatography using 30% ether/hexanes to give roughly a 7:1 mixture of desired sulfonamide **SI-40** to ditosylated sulfonamide (2.80 g, ca. 60% yield desired). This was carried forward without additional purification. Representative ^1H NMR shifts of desired product shown below.

^{1}H NMR (300 MHz, $CDCl_3$) δ 11.60 (s, 1H), 7.75 (d, *J* = 8.3 Hz, 2H), 7.24 (d, *J* = 8.3 Hz, 2H), 3.12 (sept, *J* = 6.7 Hz, 1H), 2.74 – 2.59 (m, 4H), 2.38 (s, 3H), 1.82 – 1.72 (m, 4H), 0.99 (d, *J* = 6.7 Hz, 6H).
HR-MS (ESI-MS) m/z: [M+Na]+ Calc'd for $C_{19}H_{23}NO_3S_2Na$ 400.1017; Found 400.1015.

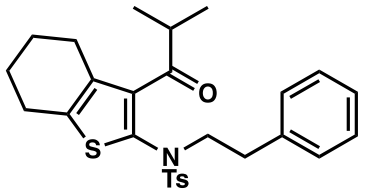

***N*-(3-isobutyryl-4,5,6,7-tetrahydrobenzo[*b*]thiophen-2-yl)-4-methyl-*N*-phenethylbenzenesulfonamide (SI-41).**

Synthesized according to general procedure 3 starting from the corresponding sulfonamide **SI-40** (2.80 g, 0.00742 mol) (7:1 mixture) and (2-iodoethyl)benzene (2.57 g, 0.0111 mol). Crude product was purified *via* flash column chromatography using 20% ether in hexanes and then recrystallization from boiling DCM/hexanes (1:1) to give sulfonamide **SI-41** as yellow crystalline solid (1.35 g, 38% yield).

*NMR had poor resolution at room temperature, so NMRs are reported below at 70 °C.

^{1}H NMR (500 MHz, $CDCl_3$, 70 °C) δ 7.55 (d, *J* = 8.3 Hz, 2H), 7.30 – 7.21 (m, 5H), 7.20 – 7.15 (d, *J* = 8.3 Hz, 2H), 4.15 – 3.21 (br s, 2H), 3.55 (quint, *J* = 6.9 Hz, 1H), 2.92 (t, *J* = 8.5 Hz, 2H), 2.71 – 2.51 (br s, 2H), 2.64 (t, *J* = 6.1 Hz, 2H), 2.42 (s, 3H), 1.94 – 1.68 (m, 4H), 1.13 (s, 6H).
^{13}C NMR (126 MHz, $CDCl_3$, 70 °C) δ 204.4, 144.3, 140.0, 137.9, 137.5, 134.4 (d, *J* = 2.9 Hz), 133.1, 129.5, 128.7, 128.6, 128.4, 126.7, 54.6, 39.4, 34.9, 25.2, 25.1, 22.9, 22.4, 21.6.
FTIR (Neat film NaCl): 3206, 3029, 2931, 2868, 1685, 1597, 1560, 1454, 1356, 1167, 1091, 1059, 662, 575.

HR-MS (ESI-MS) m/z: [M+Na]+ Calc'd for $C_{27}H_{31}NO_3S_2Na$ 504.1643; Found 504.1660.

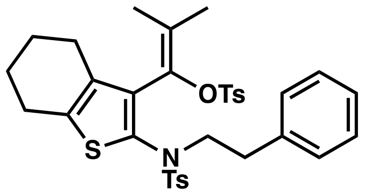

2-methyl-1-(2-((4-methyl-*N*-phenethylphenyl)sulfonamido)-4,5,6,7-tetrahydrobenzo[*b*]thiophen-3-yl)prop-1-en-1-yl 4-methylbenzenesulfonate (SI-42).

Synthesized according to a slightly modified general procedure 5 starting from the corresponding ketone **SI-41** (1.35 g, 0.00280 mol). Followed procedure with exception that 10 mL of PhMe was used for ketone solution and 14 mL DCM used for tosic anhydride due to poor solubility of ketone. Crude product was purified *via* flash column chromatography using 20% diethyl ether in hexanes to give vinyl tosylate **SI-42** as a white solid (0.39 g, 22% yield).

^{1}H NMR (500 MHz, $CDCl_3$, 70 °C) δ 7.69 (d, *J* = 7.9 Hz, 2H), 7.63 (d, *J* = 8.0 Hz, 2H), 7.26 – 7.19 (m, 7.7 Hz, 4H), 7.17 (app q, *J* = 7.0 Hz, 1H), 7.12 (d, *J* = 8.0 Hz, 2H), 7.00 (d, *J* = 7.4 Hz, 2H), 3.65 (td, *J* = 12.8, 12.4, 5.4 Hz, 1H), 3.50 (td, *J* = 12.7, 5.0 Hz, 1H), 2.86 (dd, *J* = 16.4, 5.5

Hz, 1H), 2.80 – 2.61 (m, 4H), 2.45 – 2.35 (m, 1H), 2.41 (s, 3H), 2.33 (s, 3H), 1.98 – 1.81 (m, 4H), 1.79 (s, 3H), 1.61 (s, 3H).

^{13}C NMR (126 MHz, $CDCl_3$, 70 °C) δ 144.3, 143.5, 138.6, 136.8, 136.7, 135.2, 134.7, 134.6, 133.1, 131.1, 129.5, 129.5, 129.4, 128.7, 128.4, 128.3, 127.8, 127.4, 126.3, 54.2, 34.8, 25.4, 25.0, 23.5, 22.8, 21.4, 21.3, 20.0, 18.5.

FTIR (Neat film NaCl): 3199, 3063, 3028, 2932, 2858, 1598, 1453, 1351, 1176, 1162, 1092, 1059, 813, 661, 580, 547.

HR-MS (ESI-MS) m/z: [M+Na]+ Calc'd for $C_{34}H_{37}NO_5S_3Na$ 658.1732; Found 658.1712.

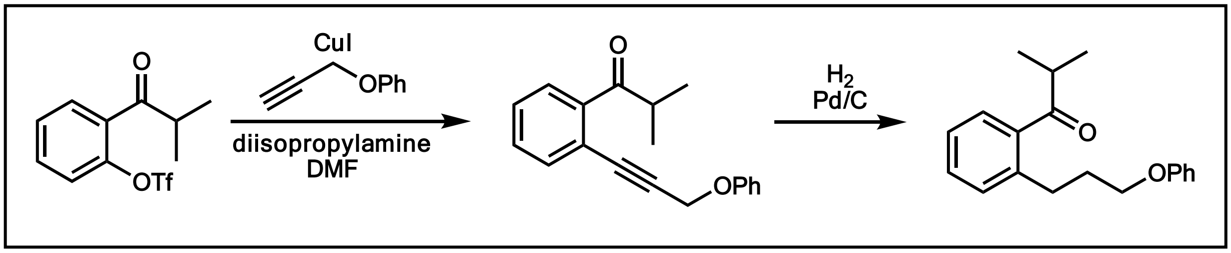

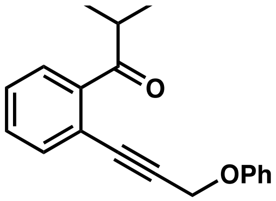

2-methyl-1-(2-(3-phenoxyprop-1-yn-1-yl)phenyl)propan-1-one (SI-43).

Copper iodide (6.43 mg, 0.034 mmol, 0.01 equiv) and $Pd(PPh_3)_4$ (39.0 mg, 0.034 mmol, 0.01 equiv) were added to a Schlenk flask and vacuum/backfilled three times. This was dissolved in DMF (12 mL) and added diisopropylamine (1.02 g, 10.1 mmol, 3 equiv) aryl triflate **SI-17** (1.00 g, 3.38 mmol, 1.0 equiv) and (prop-2-yn-1-yloxy)benzene (1.34 g, 10.1 mmol, 3.0 equiv). The resulting solution was heated to 80 °C in the silicone oil bath for 16 hours. The reaction was cooled to r.t and diluted with 30 mL of H_2O. This was then extracted with diethyl ether (3 x 40 mL). The combined organics were washed with 1M aqueous HCl (50 mL), water (50 mL) and brine (50 mL). Afterwards, the organic layer was dried over $MgSO_4$, filtered and concentrated to give crude alkyne. This was purified by silica flash column chromatography using 3% ether in hexanes to give desired product **SI-43** as an orange oil (540 mg, 58% yield).

^{1}H NMR (600 MHz, $CDCl_3$) δ 7.53 – 7.46 (m, 2H), 7.42 – 7.35 (m, 2H), 7.34 – 7.30 (m, 2H), 7.01 (ddd, J = 9.1, 7.0, 0.9 Hz, 3H), 4.94 (s, 2H), 3.53 (sept, J = 6.9 Hz, 1H), 1.09 (d, J = 6.9, 6H).

^{13}C NMR (151 MHz, $CDCl_3$) δ 208.0, 157.6, 141.9, 133.8, 130.3, 129.5, 128.6, 127.7, 121.5, 119.9, 114.9, 88.7, 85.4, 56.4, 38.9, 18.4.

FTIR (Neat film NaCl): 3063, 2971, 2932, 2871, 1691, 1598, 1589, 1494, 1211, 1033, 752, 590.

HR-MS (CI-MS) m/z: [M]+ Calc'd for $C_{19}H_{18}O_2$ 278.1307; Found 278.1307.

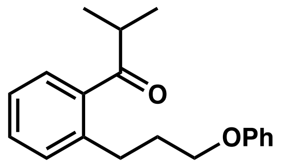

2-methyl-1-(2-(3-phenoxypropyl)phenyl)propan-1-one (SI-44).

To a 25 mL roundbottom flask was added 10% Pd/C (70.4 mg, 0.066 mmol, 0.034 equiv) and suspended in 8 mL of ethanol. To this was added alkyne **SI-43** (0.540 g, 1.94 mmol, 1.0 equiv) and the reaction was sparged with hydrogen gas for 10 minutes. After the sparging, a new hydrogen balloon was attached and reaction stirred for 18 hours. At this point, the reaction was filtered through celite and concentrated. The crude material was purified by a short silica plug with 5% ether in hexanes to give pure ketone **SI-44** as a light yellow oil (0.230 g, 1.94 mmol, 42% yield).

^{1}H NMR (600 MHz, $CDCl_3$) δ 7.53 (dd, *J* = 7.7, 1.3 Hz, 1H), 7.38 (td, *J* = 7.5, 1.3 Hz, 1H), 7.33 – 7.19 (m, 4H), 6.94 (td, *J* = 7.3, 1.1 Hz, 1H), 6.91 (d, *J* = 8.8 Hz, 2H), 4.00 (t, *J* = 6.2 Hz, 2H), 3.36 (sept, *J* = 6.9 Hz, 1H), 2.96 – 2.79 (m, 2H), 2.26 – 1.97 (m, 2H), 1.18 (d, *J* = 6.9 Hz, 6H).

^{13}C NMR (151 MHz, $CDCl_3$) δ 209.3, 158.9, 141.1, 138.6, 130.9, 130.7, 129.4, 127.6, 125.8, 120.5, 114.5, 67.0, 38.9, 31.3, 30.2, 18.7.

FTIR (Neat film NaCl): 3067, 2930, 2869, 1686, 1599, 1497, 1469, 1243, 1037, 976, 751, 591.

HR-MS (CI-MS) m/z: [M+H]+ Calc'd for $C_{19}H_{23}O_2$ 283.1698; Found 283.1700.

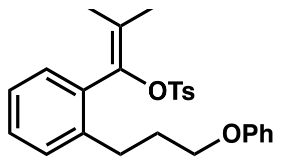

2-methyl-1-(2-(3-phenoxypropyl)phenyl)prop-1-en-1-yl 4-methylbenzenesulfonate (SI-45).

Synthesized according to general procedure 5 starting from the corresponding ketone **SI-44** (0.23 g, 0.00082 mol). Crude product was purified *via* flash column chromatography using 30% ether in hexanes to give vinyl tosylate **SI-45** as a white solid (90 mg, 25% yield).

^{1}H NMR (500 MHz, $CDCl_3$) δ 7.36 (d, *J* = 8.4 Hz, 2H), 7.31 – 7.24 (m, 1H), 7.26 – 7.25 (m, 1H), 7.23 – 7.19 (m, 1H), 7.16 (td, *J* = 7.5, 1.6 Hz, 1H), 7.09 (td, *J* = 7.5, 1.4 Hz, 1H), 7.01 (t, *J* = 8.0 Hz, 3H), 6.93 (t, *J* = 7.4 Hz, 1H), 6.84 (d, *J* = 7.7 Hz, 2H), 3.87 – 3.79 (m, 2H), 2.77 – 2.65 (m, 1H), 2.51 – 2.40 (m, 1H), 2.31 (s, 3H), 1.91 (s, 3H), 1.90 – 1.83 (m, 2H), 1.55 (s, 3H).

^{13}C NMR (126 MHz, $CDCl_3$) δ 158.9, 143.9, 141.1, 140.7, 134.5, 132.7, 132.3, 129.4, 129.1, 128.9, 128.8, 127.5, 127.2, 125.4, 120.5, 114.4, 66.8, 29.7, 29.1, 21.5, 19.8, 18.3.

FTIR (Neat film NaCl): 3065, 2923, 2870, 1600, 1497, 1367, 1245, 1177, 1080, 1037, 990.

HR-MS (ESI-MS) m/z: [M+Na]+ Calc'd for $C_{26}H_{28}O_4SNa$ 459.1606; Found 459.1619.

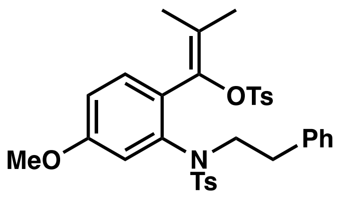

1-(4-methoxy-2-((4-methyl-N-phenethylphenyl)sulfonamido)phenyl)-2-methylprop-1-en-1-yl 4-methylbenzenesulfonate (SI-46).

Synthesized according to general procedure 5 starting from the cooresponding ketone N-(2-isobutyryl-5-methoxyphenyl)-4-methyl-N-phenethylbenzenesulfonamide (0.548 g, 0.00158 mol). Crude product was purified *via* flash column chromatography using 1:1 hexanes:diethyl ether to give vinyl tosylate **SI-46** as a yellow solid (0.31 g, 32% yield).

^{1}H NMR (400 MHz, $CDCl_3$) δ 7.58 (d, *J* = 7.9 Hz, 2H), 7.51 (d, *J* = 8.2 Hz, 2H), 7.31 (br s, 1H), 7.21–7.18 (m, 2H), 7.17 – 7.07 (m, 3H), 7.03 (d, *J* = 8.1 Hz, 2H), 6.86 (d, *J* = 7.6 Hz, 2H,), 6.85 – 6.77 (m, 1H), 6.39 (br s, 1H), 3.66 (s, 3H), 3.53 (ddd, *J* = 12.2, 12.2, 6.0 Hz, 1H), 3.29 (ddd, *J* = 13.6, 12.2, 4.9 Hz, 1H), 2.66 (ddd, *J* = 12.6, 12.6, 5.3 Hz, 1H), 2.50 – 2.38 (m, 1H), 2.35 (s, 3H), 2.24 (s, 3H), 1.71 (s, 3H), 1.56 (s, 3H).
^{13}C NMR (101 MHz, $CDCl_3$) δ 159.0, 143.5, 142.5, 139.1, 137.5, 136.8, 135.12 133.4, 133.2, 128.4, 128.4, 127.7, 127.3, 127.2, 126.9, 125.3, 114.0, 112.2, 54.4, 52.1, 33.5, 20.5, 20.5, 19.5, 17.7.
FTIR (Neat film NaCl): 3028, 2924, 1686, 1603, 1569, 1496, 1456, 1352, 1305, 1291, 1189, 1176, 1162, 1093, 1034, 985, 948, 830, 814, 786, 756, 695, 658, 567.

HR-MS (ESI-MS) m/z: $[M+Na]^+$ Calc'd for $C_{33}H_{35}NO_6S_2Na$ 628.1804; Found 628.1814.

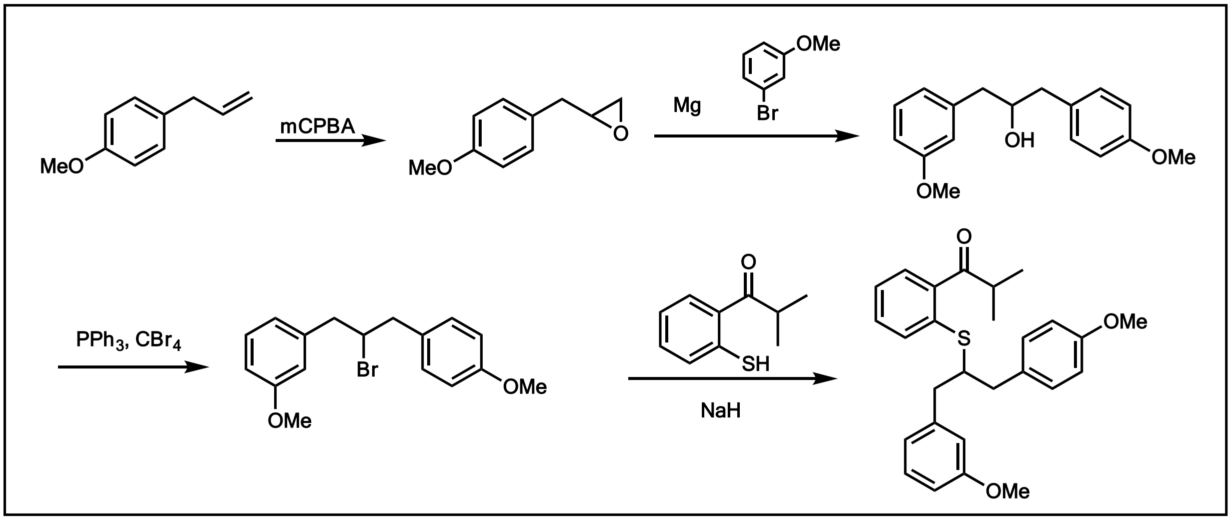

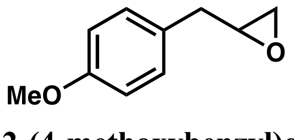

2-(4-methoxybenzyl)oxirane (SI-47).

1-allyl-4-methoxybenzene (5.00 g, 33.7 mmol) was dissolved in DCM (68 mL) and the solution was cooled down to 0 °C. m-chloroperoxybenzoic acid (9.98 g, 40.5 mmol, 70%) was added portion wise over 10 min. The reaction was stirred under room temperature overnight. It was then quenched with saturated $NaHSO_3$ solution. After the separation, the organic phase was washed with saturated $NaHCO_3$ solution and brine. It was then dried with $MgSO_4$, filtered and

concentrated in vacuo to get the product as an orange liquid **SI-47** (4.42 g, 79.8% yield). The product **SI-47** was used in the next step without further purification. Spectral data match those reported in the literature.[15]

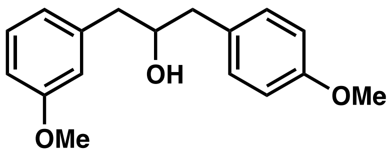

1-(3-methoxyphenyl)-3-(4-methoxyphenyl)propan-2-ol (SI-48).

In a flamed-dried 3-neck flask was added magnesium (0.51 g, 21.1 mmol) and THF (22 mL). A small amount of iodine was added to assist the initiation of Grignard reagent formation. 1-bromo-3-methoxybenzene (3.78 g, 20.2 mmol) was added into the reaction slowly to keep a gentle reflux of the reaction. Upon completion of the addition, the reaction was left stirred for 1 hour. Then copper iodide (0.38 g, 2.02 mmol) was added into the reaction and it was cooled down to 0 °C. **SI-47** (2.21 g, 13.5 mmol) in THF (22 mL) was added slowly into the reaction, and it was then warmed up to room temperature overnight. The reaction was quenched with saturated NH_4Cl solution at 0 °C and then diluted with diethyl ether. After the separation, the aqueous phase was extracted with diethyl ether two more times. The combined organic phase was washed with brine, dried with $MgSO_4$, filtered and concentrated in vacuo to give the crude product. The crude product was purified *via* flash column chromatography using 1:1 diethyl ether and hexanes to give the product **SI-48** as a yellowish oil (2.64 g, 72% yield).

^{1}H NMR (400 MHz, $CDCl_3$) δ 7.25 – 7.20 (m, 1H), 7.15 (d, *J* = 8.7 Hz, 2H), 6.86 (d, *J* = 8.6 Hz, 2H), 6.84 – 6.80 (m, 1H), 6.80 – 6.76 (m, 2H), 4.02 (tt, *J* = 8.1, 4.7 Hz, 1H), 3.80 (s, 3H), 3.80 (s, 3H), 2.82 (ddd, *J* = 12.7, 7.8, 4.6 Hz, 2H), 2.71 (ddd, *J* = 13.6, 8.1, 5.0 Hz, 2H).

^{13}C NMR (101 MHz, $CDCl_3$) δ 160.1, 158.6, 140.4, 130.70, 130.67, 129.9, 122.1, 115.4, 114.3, 112.2, 73.9, 55.6, 55.5, 43.7, 42.8.

FTIR (Neat film NaCl): 3436, 2995, 2936, 2835, 1610, 1583, 1511, 1488, 1245, 1035, 806, 781, 696.

HR-MS (FD-MS) m/z: [M]+ Calc'd for $C_{17}H_{20}O_3$ 272.1412; Found 272.1410.

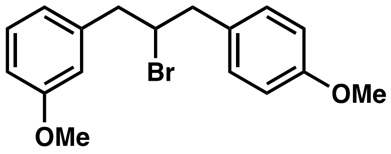

1-(2-bromo-3-(4-methoxyphenyl)propyl)-3-methoxybenzene (SI-49).

In a flask was added **SI-48** (1.32 g, 4.85 mmol), DCM (15 mL) and carbon tetrabromide (2.41 g, 7.27 mmol). The solution was cooled down to 0 °C. Triphenylphosphine (1.27 g, 4.85 mmol) was dissolved in DCM (10 mL) in another flask and slowly added to the reaction. The reaction was warmed up to room temperature overnight. It was then quenched with DCM and water. After the separation, the aqueous phase was extracted with DCM two more times. The combined organic phase was washed with brine, dried with $MgSO_4$, filtered and concentrated in vacuo to give the

crude product. The crude product was purified *via* flash column chromatography using 2% diethyl ether in hexanes to give the product **SI-49** as a colorless liquid (0.56 g, 34% yield).

^{1}H NMR (400 MHz, $CDCl_3$) δ 7.23 (t, *J* = 8.1 Hz, 1H), 7.12 (d, *J* = 8.2 Hz, 2H), 6.85 (d, *J* = 8.7 Hz, 2H), 6.82 – 6.77 (m, 2H), 6.76 – 6.74 (m, 1H), 4.33 (tt, *J* = 8.1, 5.8 Hz, 1H), 3.80 (s, 3H), 3.80 (s, 3H), 3.22 – 3.04 (m, 4H).

^{13}C NMR (101 MHz, $CDCl_3$) δ 159.9, 158.8, 140.4, 130.8, 130.6, 129.8, 121.9, 115.4, 114.2, 112.3, 57.8, 55.6, 55.5, 45.2, 44.4.

FTIR (Neat film NaCl): 2989, 2870, 1393, 1143.

HR-MS (FD-MS) m/z: [M]+ Calc'd for $C_{17}H_{19}O_2Br$ 334.0568; Found 334.0563.

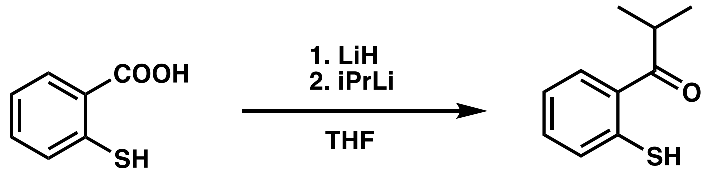

1-(2-mercaptophenyl)-2-methylpropan-1-one (SI-50).

In a flamed dried 3-neck flask was added 2-mercaptobenzoic acid (2.00 g, 13.0 mmol) and THF (26 mL). Lithium hydride (0.31 g, 39 mmol) was added slowly, and the reaction was refluxed for 45 min. Then the solution was cooled down to 0 °C. Isopropyllithium (0.7 M in pentane, 18.5 mL, 13.0 mmol) was added slowly to the solution. After 12 hours, the reaction was quenched with saturated NH_4Cl solution. The solution was then acidified with 3 M HCl until the aqueous phase did not crash out solid anymore. It was then diluted with water and diethyl ether. After the separation, the aqueous phase was extracted with diethyl ether two more times. The combined organic phase was washed with brine, dried with $MgSO_4$, filtered and concentrated in vacuo to give the crude product. The crude product was purified via flash column chromatography using 12.5% diethyl ether in hexanes to give the product **SI-50** as a yellow liquid (1.73 g, 74% yield).

^{1}H NMR (300 MHz, $CDCl_3$) δ 7.78 (d, *J* = 7.9 Hz, 1H), 7.32 – 7.09 (m, 3H), 4.17 (s, 1H), 3.47 (hept, *J* = 6.8 Hz, 1H), 1.16 (d, J = 6.9 Hz, 6H).

^{13}C NMR (101 MHz, $CDCl_3$) δ 205.4, 137.3, 132.7, 132.0, 131.9, 130.3, 124.7, 36.3, 19.1.

FTIR (Neat film NaCl): 2971, 2931, 2871, 2537, 1665, 1587, 1558, 1468, 1220, 1083, 988, 739.

HR-MS (FD-MS) m/z: [M]+ Calc'd for $C_{10}H_{12}OS$ 180.0609; Found 180.0603.

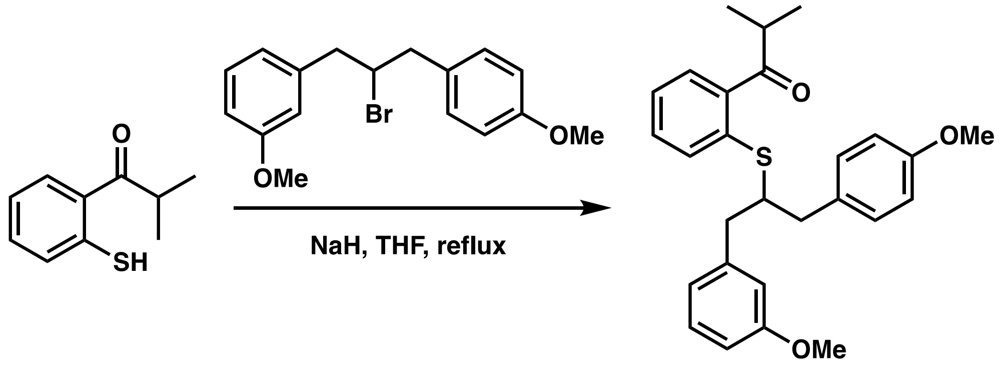

1-(2-((1-(3-methoxyphenyl)-3-(4-methoxyphenyl)propan-2-yl)thio)phenyl)-2-methylpropan-1-one (SI-51).

In a flamed Schlenk flask was charged with NaH (89 mg, 2.2 mmol, 60% dispersion in mineral oil) and THF (11 mL). The reaction was cooled down to 0 degree and **SI-50** (0.40 g, 2.2 mmol) was added dropwise. The reaction was then warmed to room temperature and left for 15 minutes. After, **SI-49** (1.1 g, 3.3 mmol) was added and the reaction was refluxed at 66 °C in the silicone oil bath overnight. After the reaction was complete, the reaction was diluted with diethyl ether and water. After the separation of the two phases, the aqueous phase was extracted with diethyl ether two more times. The combined organic phase was washed with 1M NaOH, saturated NH_4Cl and brine. It was then dried with $MgSO_4$, filtered and concentrated in vacuo. The crude product was purified by column chromatography to get the product **SI-51** as a pale yellow oil (0.57 g, 59% yield).

^{1}H NMR (400 MHz, $CDCl_3$) δ 7.30 (td, *J* = 7.6, 1.4 Hz, 2H), 7.24 (td, *J* = 7.6, 1.6 Hz, 1H), 7.15 (td, *J* = 7.4, 1.3 Hz, 1H), 7.11 (t, *J* = 7.9 Hz, 1H), 6.99 (d, *J* = 8.5 Hz, 2H), 6.73 (d, *J* = 8.0 Hz, 2H), 6.66 (dd, J = 7.9, 2.1 Hz, 2H), 6.60 (t, J = 2.0 Hz, 1H), 3.71 (s, 6H), 3.49 (pent, J = 7.0 Hz, 1H), 3.21 (hept, J = 6.8 Hz, 1H), 2.79 – 2.63 (m, 4H), 1.04 (d, J = 6.9 Hz, 6H).

^{13}C NMR (101 MHz, $CDCl_3$) δ 209.1, 159.6, 158.2, 142.6, 140.7, 134.5, 132.0, 131.0, 130.24, 130.21, 129.3, 127.9, 126.2, 121.6, 115.0, 113.7, 111.7, 55.3, 55.2, 52.1, 40.6, 39.7, 39.6, 18.45, 18.43.

FTIR (Neat film NaCl): 2962, 2929, 1691, 1611, 1602, 1584, 1512, 1465, 1438, 1261, 1248, 1037, 978.

HR-MS (ESI-MS) m/z: [M+Na]+ Calc'd for $C_{27}H_{30}O_3SNa$ 457.1813; Found 457.1820.

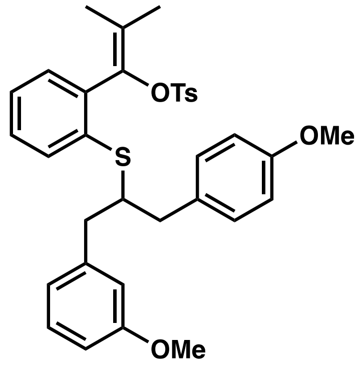

1-(2-((1-(3-methoxyphenyl)-3-(4-methoxyphenyl)propan-2-yl)thio)phenyl)-2-methylprop-1-en-1-yl 4-methylbenzenesulfonate (28).

Synthesized according to a slightly modified general procedure 5 starting from the corresponding ketone **SI-51** (0.56 g, 0.0013 mol). Crude product was purified *via* flash column chromatography using 33% diethyl ether in hexanes to give vinyl tosylate **28** as a yellowish oil (0.42 g, 54% yield).

^{1}H NMR (400 MHz, $CDCl_3$) δ 7.37 (dd, *J* = 8.4, 2.8 Hz, 2H), 7.25 – 7.09 (m, 3H), 7.07 (d, *J* = 7.2 Hz, 2H), 7.01 (dd, *J* = 8.5, 1.9 Hz, 2H), 6.91 (dd, *J* = 8.3, 3.5 Hz, 2H), 6.83 – 6.75 (m, 2H), 6.75 – 6.70 (m, 1H), 6.68 (d, *J* = 7.6 Hz, 1H), 6.64 (s, 1H), 3.79 (m, 3H), 3.77 (m, 3H), 3.44 (tt, *J* = 7.4, 3.7 Hz, 1H), 2.80 – 2.51 (m, 4H), 2.20 (m, 3H), 1.93 (m, 3H), 1.57 (s, 3H). (Some singlet peaks split likely due to the existence of rotamers)

^{13}C NMR (101 MHz, $CDCl_3$) δ 158.5, 157.1, 142.8, 140.0, 139.7, 138.8, 136.1, 133.3, 131.8, 130.3, 130.0, 129.2, 128.9, 128.2, 127.9, 127.0, 126.6, 124.4, 120.6, 114.1, 112.6, 110.4, 54.2, 54.1, 50.1, 39.5, 38.6, 20.4, 18.9, 17.5. (Some peaks split likely due to the existence of rotamers)

FTIR (Neat film NaCl): 2915, 1600, 1584, 1512, 1463, 1455, 1436, 1364, 1248, 1189, 1176, 1085, 1036, 990, 823, 809, 793, 740.

HR-MS (ESI-MS) m/z: $[M+NH_4]+$ Calc'd for $C_{34}H_{40}NO_5S_2$ 606.2348; Found 606.2336.

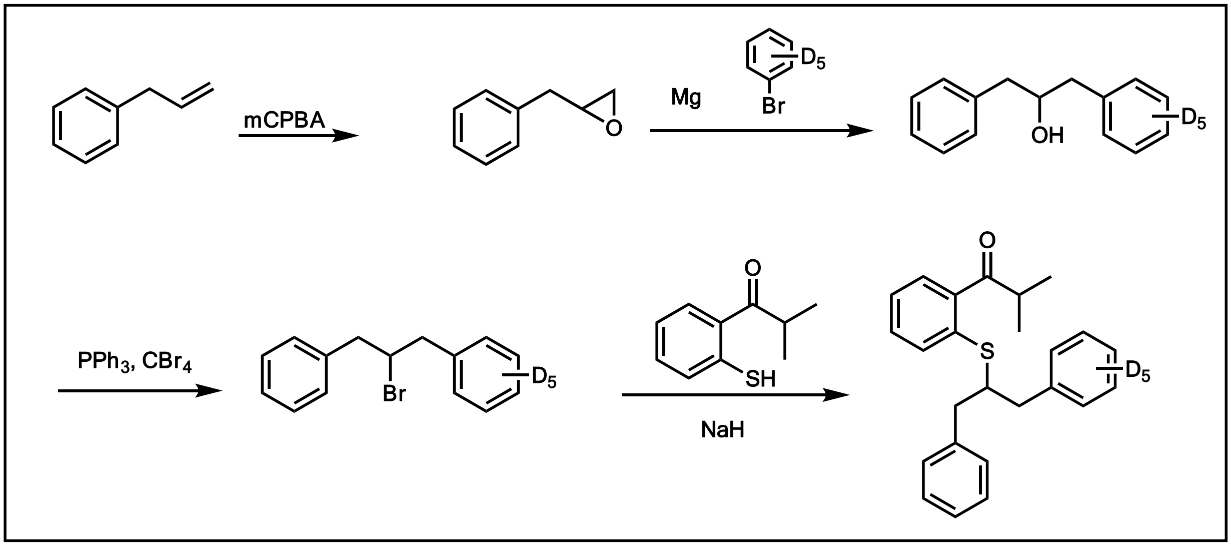

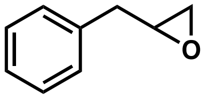

2-benzyloxirane (SI-53).

Allylbenzene (5.00 g, 42.3 mmol) was dissolved in DCM (84 mL) and the solution was cooled down to 0 °C. m-chloroperoxybenzoic acid (12.5 g, 50.8 mmol, 70%) was added portion wise over 10 min. The reaction was stirred under room temperature overnight. It was then quenched with saturated $NaHSO_3$ solution. After the separation, the organic phase was washed with saturated $NaHCO_3$ solution and brine. It was then dried with $MgSO_4$, filtered and concentrated in vacuo to get the product as a pale yellow liquid **SI-53** (3.05 g, 53.7% yield). The product **SI-53** was used in the next step without further purification. Spectral data match those reported in the literature.[15]

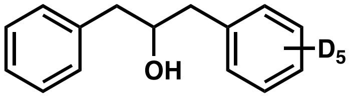

1-phenyl-3-(phenyl-*d*$_5$)propan-2-ol (SI-54).

In a flamed-dried 3-neck flask was added magnesium (0.87 g, 35.7 mmol) and THF (36 mL). A small amount of iodine was added to assist the initiation of Grignard reagent formation. 1-bromobenzene-2,3,4,5,6-d$_5$ (5.52 g, 34.1 mmol) was added into the reaction slowly to keep a gentle reflux of the reaction. Upon completion of the addition, the reaction was left stirred for 1 hour.

Then copper iodide (0.65 g, 3.4 mmol) was added into the reaction and it was cooled down to 0 °C. **SI-53** (3.05 g, 22.7 mmol) in THF (36 mL) was added slowly into the reaction, and it was then warmed up to room temperature overnight. The reaction was quenched with saturated NH_4Cl solution at 0 °C and then diluted with diethyl ether. After the separation, the aqueous phase was extracted with diethyl ether two more times. The combined organic phase was washed with brine, dried with $MgSO_4$, filtered, and concentrated in vacuo to give the crude product. The crude product was purified *via* flash column chromatography using 33% diethyl ether in hexanes to give the product **SI-54** as a colorless liquid (4.37 g, 88.5% yield).

^{1}H NMR (400 MHz, $CDCl_3$) δ 7.36 – 7.28 (m, 2H), 7.25–7.21 (ddd, *J* = 7.0, 3.4, 1.6 Hz, 3H), 4.08 (tt, *J* = 8.2, 4.7 Hz, 1H), 2.88 (ddd, *J* = 13.7, 4.8, 1.1 Hz, 2H), 2.77 (ddd, *J* = 13.7, 8.2, 0.8 Hz, 2H).

^{13}C NMR (101 MHz, $CDCl_3$) δ 138.5, 138.3, 129.4, 129.0 (t, *J* = 24 Hz), 128.6, 128.1 (t, *J* = 24 Hz), 126.5, 126.0 (t, *J* = 24 Hz), 73.6, 43.4, 43.3.

FTIR (Neat film NaCl): 3407, 3027, 2916, 2273, 1600, 1569, 1496, 1454, 1384, 1355, 1189, 1176, 1068, 1030, 988, 821, 747, 699, 545.

HR-MS (FD-MS) m/z: [M]+ Calc'd for $C_{15}H_{11}OD_5$ 217.1515; Found 217.1523.

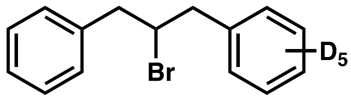

1-(2-bromo-3-phenylpropyl)benzene-2,3,4,5,6-*d*$_5$ (SI-55).

In a flask was added **SI-54** (3.36 g, 15.5 mmol), DCM (20 mL) and carbon tetrabromide (7.69 g, 23.2 mmol). The solution was cooled down to 0 °C. Triphenylphosphine (6.08 g, 23.2 mmol) was dissolved in DCM (13 mL) in another flask and slowly added to the reaction. The reaction was warmed up to room temperature overnight. It was then quenched with DCM and water. After the separation, the aqueous phase was extracted with DCM two more times. The combined organic phase was washed with brine, dried with $MgSO_4$, filtered and concentrated in vacuo to give the crude product. The crude product was purified *via* flash column chromatography using 2% diethyl ether in hexanes to give the product **SI-55** as a colorless liquid (3.00 g, 63.0% yield, 91% purity).

*The product had 9% of alkene side product from elimination, but was used directly in the next synthetic step.

^{1}H NMR (500 MHz, $CDCl_3$) δ 7.36 – 7.16 (m, 5H), 4.37 (tt, *J* = 8.2, 5.7 Hz, 1H), 3.22 (ddd, *J* = 14.3, 5.8, 1.3 Hz, 2H), 3.14 (ddd, *J* = 14.3, 8.2, 1.8 Hz, 2H).

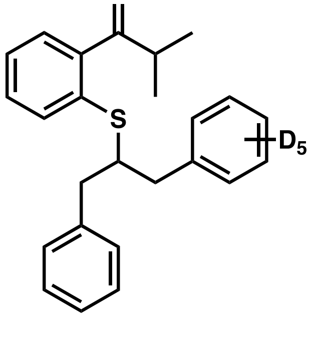

2-methyl-1-(2-((1-phenyl-3-(phenyl-*d*$_5$)propan-2-yl)thio)phenyl)propan-1-one (SI-56).

In a flamed Schlenk flask was charged with NaH (0.27 g, 6.7 mmol, 60% dispersion in mineral oil) and THF (22 mL). **SI-50** (0.80 g, 4.4 mmol) was added dropwise. The reaction was stirred for 15 minutes under room temperature. After, **SI-55** (1.9 g, 6.7 mmol) was added and the reaction was refluxed at 66 °C in the silicone oil bath overnight. After the reaction was complete, the reaction was diluted with diethyl ether and water. After the separation of the two phases, the aqueous phase was extracted with diethyl ether two more times. The combined organic phase was washed with 1M NaOH, saturated NH_4Cl and brine. It was then dried with $MgSO_4$, filtered and concentrated in vacuo. The crude product was purified by column chromatography to get the product **SI-56** as a pale yellow oil (0.75 g, 45% yield).

^{1}H NMR (400 MHz, $CDCl_3$) δ 7.39–7.35 (m, 2H), 7.31 (t, *J* = 7.6 Hz, 1H), 7.29–7.27 (m, 1H), 7.25 – 7.16 (m, 3H), 7.14 (d, *J* = 6.9 Hz, 2H), 3.60 (penta, *J* = 7.0 Hz, 1H), 3.27 (hepta, *J* = 6.9 Hz, 1H), 2.90 – 2.76 (m, 4H), 1.10 (d, *J* = 6.9 Hz, 6H).
^{13}C NMR (101 MHz, $CDCl_3$) δ 208.0, 141.6, 138.0, 137.8, 133.3, 131.0, 129.2, 128.2, 127.3, 126.9, 125.4, 125.2, 50.9, 39.6, 39.5, 38.6, 17.4.
FTIR (Neat film NaCl): 2967, 2925, 2850, 2273, 1692, 1585, 1496, 1454, 1435, 1382, 1342, 1261, 1215, 1077, 1029, 977, 747, 700, 544.

HR-MS (FD-MS) m/z: [M]+ Calc'd for $C_{25}H_{21}OSD_5$ 379.2018; Found 379.2011.

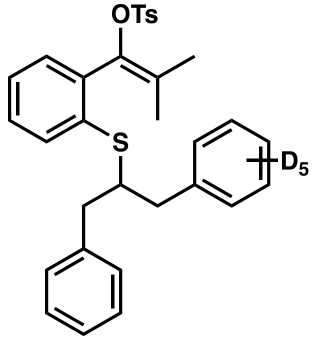

2-methyl-1-(2-((1-phenyl-3-(phenyl-*d*$_5$)propan-2-yl)thio)phenyl)prop-1-en-1-yl-4-methylbenzenesulfonate (30).

Synthesized according to a slightly modified general procedure 5 starting from the corresponding ketone **SI-56** (0.76 g, 0.0020 mol). Crude product was purified *via* flash column chromatography using 33% diethyl ether in hexanes to give vinyl tosylate **30** as a yellowish oil (0.50 g, 47% yield).

^{1}H NMR (400 MHz, $CDCl_3$) δ 7.30 (d, *J* = 8.8 Hz, 2H), 7.23–7.20 (m, 1H), 7.19 – 6.95 (m, 9H), 6.82 (d, *J* = 8.0 Hz, 2H), 3.42 (p, *J* = 7.0 Hz, 1H), 2.77 – 2.49 (m, 4H), 2.12 (s, 3H), 1.86 (s, 3H).

^{13}C NMR (101 MHz, $CDCl_3$) δ 142.8, 138.7, 138.3, 138.0, 136.1, 134.0, 133.3, 131.8, 128.9, 128.2, 128.1, 127.93, 127.91, 127.3, 127.2, 127.0, 126.6, 125.4, 125.3, 124.4, 49.9, (39.47, 39.42, 39.37, 39.32), 20.4, 18.9, 17.5. (The secondary alkyl carbon split to 4 peaks likely due to the existence of rotomers)

FTIR (Neat film NaCl): 2920, 2853, 2362, 2273, 1634, 1598, 1496, 1454, 1436, 1364, 1189, 1176, 1085, 1069, 990, 821, 807, 792, 740, 701, 669, 590, 561, 546.

HR-MS (FD-MS) m/z: [M]+ Calc'd for $C_{32}H_{27}O_3S_2D_5$ 533.2107; Found 533.2099.

3. Friedel-Crafts reactions

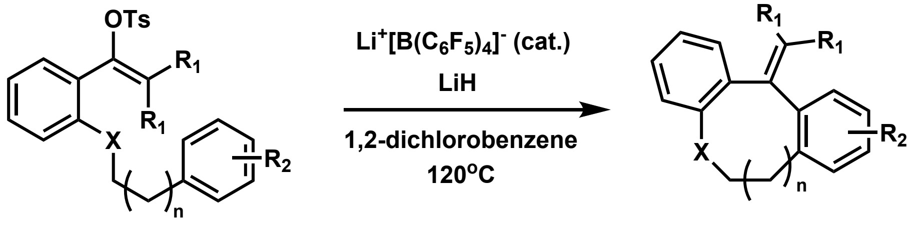

General Procedure 6: In the glovebox, lithium tetrakis(pentafluorophenyl)borate (0.1 equiv), lithium hydride (5.0 equiv), and vinyl tosylate (1.0 equiv) were dissolved into 1,2-dichlorobenzene (618 equiv) to generate a 0.0143 M solution for the vinyl tosylate. The reaction was heated under 140 °C overnight. The reaction solution was directly purified *via* flash column chromatography using hexanes and then ethyl acetate to get rid of 1,2-dichlorobenzene. Then the crude product was purified *via* flash column chromatography again to get the pure product.

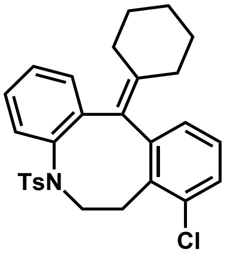

8-chloro-12-cyclohexylidene-5-tosyl-5,6,7,12-tetrahydrodibenzo[*b,e*]azocine (2).

Synthesized according to general procedure 6 starting from the corresponding vinyl tosylate **1** (0.150 g, 0.000231 mol). Crude product was purified via flash column chromatography using 10% ethyl ether in hexanes to give the product **2** as a white powder (0.080 g, 73% yield).

^{1}H NMR (400 MHz, $CDCl_3$) δ 7.85 (d, *J* = 7.8 Hz, 2H), 7.36 (d, *J* = 7.9 Hz, 2H), 7.25 (m, 2H), 7.18 (m, 1H), 7.08 (m, 3H), 6.90 (d, *J* = 8.0 Hz, 1H), 4.31 (dd, *J* = 15.3, 6.9 Hz, 1H), 3.56 (dd, *J* = 14.9, 6.9 Hz, 1H), 3.36 (dd, *J* = 15.1, 9.4 Hz, 1H), 2.80 (dd, *J* = 15.3, 9.4 Hz, 1H), 2.46 (s, 3H), 2.21 (m, 2H), 2.06 (m, 2H), 1.87 (m, 1H), 1.57 (m, 5H).

^{13}C NMR (101 MHz, $CDCl_3$) δ 146.1, 145.4, 143.2, 140.5, 139.4, 139.2, 136.7, 133.9, 130.1, 130.0, 129.8, 128.7, 128.1, 127.8, 127.7, 127.6, 127.4, 127.3, 50.3, 33.7, 31.8, 31.5, 28.1, 27.8, 26.5, 21.6.

FTIR (Neat film NaCl): 3062, 2925, 2852, 1560, 1482, 1446, 1349, 1158, 1092, 569.

HR-MS (ESI-MS) m/z: [M+Na]+ Calc'd for $C_{28}H_{28}ClNO_2SNa$ 500.1427; Found 500.1436.

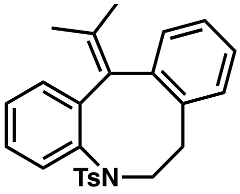

12-(propan-2-ylidene)-5-tosyl-5,6,7,12-tetrahydrodibenzo[*b,e*]azocine (5).

Synthesized according to general procedure 6 at 140 °C for 36 hours starting from the corresponding vinyl tosylate **SI-16** (28.6 mg, 0.0500 mmol). Crude product was purified *via* flash column chromatography using a gradient of 1-15% diethyl ether in hexanes to give arene **5** as white solid (11.3 mg, 56% yield).

Performing the reaction with **SI-16** (572 mg, 1.00 mmol) in a Schlenk heating at 140 °C in the silicone oil bath outside the glovebox gave the product **5** as a white solid (265 mg, 66% yield).

*NMR had poor resolution at room temperature, so NMR spectra are reported below at 75 °C.

^{1}H NMR (500 MHz, DMSO-d_6, 75 °C) δ 7.70 (d, J = 7.8 Hz, 2H), 7.38 (d, J = 7.9 Hz, 2H), 7.30 – 7.16 (m, 2H), 7.15 – 7.00 (m, 6H), 3.60 (br s, 2H), 2.97 (br s, 2H), 2.39 (s, 3H), 1.68 (s, 3H), 1.56 (s, 3H).

^{13}C NMR (126 MHz, DMSO-d_6, 75 °C) δ 143.7, 143.5, 139.7, 138.9, 138.7, 134.5, 131.6, 130.5, 130.3, 128.9, 128.4, 127.8, 127.7, 127.3, 127.2, 51.5 (br s), 37.5 (br s), 21.9, 21.4, 21.1.

FTIR (Neat film NaCl): 3065, 2955, 2923, 2854, 1738, 1599, 1484, 1447, 1348, 1325, 1159, 1092, 1020, 813, 717, 568, 549.

HR-MS (CI-MS) m/z: [M]+ Calc'd for $C_{25}H_{25}NO_2S$ 403.1606; Found 403.1620.

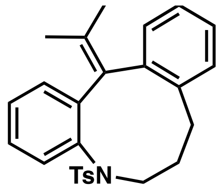

13-(propan-2-ylidene)-5-tosyl-6,7,8,13-tetrahydro-5*H*-dibenzo[*b*,*e*]azonine (6).

Synthesized according to general procedure 6 at 140 °C for 36 hours starting from the corresponding vinyl tosylate **SI-11** (29.5 mg, 0.0500 mmol). Crude product was purified *via* flash column chromatography using a gradient of 1-15% diethyl ether in hexanes to give the product **6** as white solid (17.1 mg, 82% yield).

^{1}H NMR (500 MHz, $CDCl_3$) δ 7.80 – 7.72 (m, 2H), 7.40 (d, *J* = 7.6 Hz, 1H), 7.34 (d, *J*= 8.1 Hz, 2H), 7.28 (t, *J* = 7.5 Hz, 1H), 7.17 (d, *J* = 7.2 Hz, 1H), 7.15 – 6.96 (m, 4H), 6.72 (d, *J* = 8.0 Hz, 1H), 3.64 – 3.43 (m, 2H), 2.71 – 2.48 (m, 2H), 2.46 (s, 3H), 1.79 (s, 3H), 1.60 (s, 3H), 1.57 – 1.47 (m, 2H).

^{13}C NMR (126 MHz, $CDCl_3$) δ 158.0, 142.4, 139.6, 137.5, 132.8, 131.7, 131.0, 129.1, 128.8, 127.9, 126.8, 125.9, 123.9, 122.3, 73.7, 31.6, 29.6, 21.4, 21.1.

FTIR (Neat film NaCl): 3062, 2973, 2920, 2859, 1597, 1483, 1445, 1350, 1161, 1083, 814, 754, 697.

HR-MS (CI-MS) m/z: [M+H]+ Calc'd for $C_{26}H_{28}NO_2S$ 418.1841; Found 418.1840.

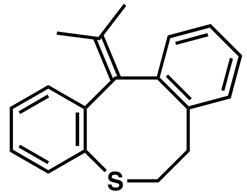

12-(propan-2-ylidene)-7,12-dihydro-6H-dibenzo[b,e]thiocine (8).

Synthesized according to general procedure 6 starting from the corresponding vinyl tosylate **SI-14** (21.9 mg, 0.0500 mmol). Crude product was purified via flash column chromatography using 5% ethyl ether in hexanes to give the product **8** as an oil (6.1 mg, 46% yield).

^{1}H NMR (400 MHz, CDCl3) δ 7.35 (d, *J* = 7.5 Hz, 1H), 7.23 (d, *J* = 7.6 Hz, 1H), 7.17 (t, *J* = 7.5 Hz, 2H), 7.12 (t, *J* = 7.2 Hz, 1H), 7.10 – 7.03 (m, 3H), 3.50 – 3.25 (m, 2H), 3.09 – 2.85 (m, 2H), 1.71 (s, 3H), 1.61 (s, 3H).
^{13}C NMR (101 MHz, $CDCl_3$) δ 140.1, 136.1, 136.0, 132.0, 131.8, 130.9, 130.2, 129.7, 127.1, 127.0, 126.9, 126.8, 37.8, 33.1, 21.6, 21.1.
FTIR (Neat film NaCl): 2917, 2850, 1664, 1484, 1463, 1444, 1260, 1018, 798, 749.

HR-MS (FD-MS) m/z: [2M]+ Calc'd for $C_{36}H_{36}S_2$ 532.2258; Found 532.2257.

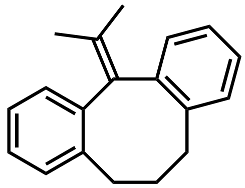

12-(propan-2-ylidene)-5,6,7,12-tetrahydrodibenzo[*a,d*][8]annulene (9).

Synthesized according to general procedure 6 starting from the corresponding vinyl tosylate **SI-20** (105 mg, 0.250 mmol). Crude product was purified via flash column chromatography using 5% ethyl ether in hexanes to give the product **9** as a white powder (50 mg, 81% yield).

^{1}H NMR (400 MHz, $CDCl_3$) δ 7.20 – 7.01 (m, 8H), 2.87 (app d, *J* = 55.2 Hz, 4H), 2.26 (br s, 1H), 1.65 (s, 6H), 1.55 – 1.39 (m, 1H).
^{13}C NMR (101 MHz, $CDCl_3$) δ 144.3, 140.7, 136.9, 129.7, 128.8, 127.9, 126.5, 126.4, 37.9 (br), 29.1 (br), 20.7.
FTIR (Neat film NaCl): 3058, 3012, 2978, 2922, 2847, 1483, 1444, 1371, 1062, 1041, 769, 747.

HR-MS (CI-MS) m/z: [M]+ Calc'd for $C_{19}H_{20}$ 248.1565; Found 248.1562.

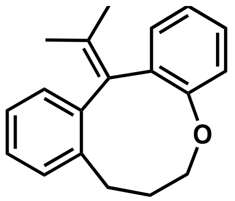

13-(propan-2-ylidene)-6,7,8,13-tetrahydrodibenzo[*b,e*]oxonine (10).

Synthesized according to general procedure 6 at 140 °C for 48 hours starting from the corresponding vinyl tosylate **SI-45** (21.8 mg, 0.0500 mmol). Crude product was purified *via* flash column chromatography using a gradient of 0-4% diethyl ether in hexanes to give the product **10** as colorless oil (8.6 mg, 65% yield).

^{1}H NMR (500 MHz, $CDCl_3$) δ 7.32 (d, *J* = 5.7 Hz, 1H), 7.20 – 7.05 (m, 5H), 6.98 (dd, *J* = 14.7, 7.6 Hz, 2H), 4.05 – 3.98 (m, 1H), 3.45 – 3.31 (m, 2H), 2.66 (d, *J* = 12.9 Hz, 1H), 2.14 (tt, *J* = 9.8, 4.1 Hz, 1H), 1.73 (s, 3H), 1.72 – 1.65 (m, 1H), 1.60 (s, 3H).

^{13}C NMR (126 MHz, $CDCl_3$) δ 157.9, 142.3, 139.6, 137.5, 132.7, 131.7, 131.0, 129.1, 128.8, 127.9, 126.7, 125.9, 123.9, 122.3, 73.6, 31.6, 29.5, 21.4, 21.0.

FTIR (Neat film NaCl): 3062, 3017, 2923, 2857, 1598, 1570, 1483, 1445, 1380, 1238, 1061, 754, 741, 630.

HR-MS (CI-MS) m/z: [M]+ Calc'd for $C_{19}H_{20}O$ 264.1514; Found 264.1512.

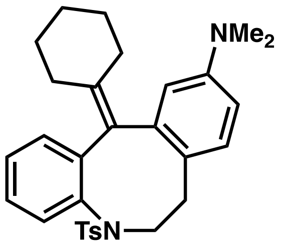

12-cyclohexylidene-N,N-dimethyl-5-tosyl-5,6,7,12-tetrahydrodibenzo[*b,e*]azocin-10-amine (11).

Synthesized according to general procedure 6 starting from the corresponding vinyl tosylate **SI-24** (32.9 mg, 0.0500 mmol). Crude product was purified via flash column chromatography using 10% ethyl ether in hexanes to give the product **11** as a white powder (0.015 g, 62% yield).

*NMR had poor resolution at room temperature, so NMRs are reported below at 75 °C in DMSO-*d*6.

^{1}H NMR (500 MHz, DMSO-d_6, 75 °C) δ 7.75 (d, J = 7.8 Hz, 2H), 7.43 (d, J = 7.8 Hz, 2H), 7.33 (d, J = 7.6 Hz, 1H), 7.24 (t, J = 7.6 Hz, 1H), 7.13 (t, J = 7.9 Hz, 1H), 6.95 (d, J = 8.0 Hz, 1H), 6.88 (dd, J = 8.3, 2.5 Hz, 1H), 6.56 – 6.35 (m, 2H), 4.19 – 4.09 (m, 1H), 3.20 (d, J = 12.0 Hz, 1H), 2.83 (d, J = 2.7 Hz, 7H), 2.74 – 2.64 (m, 1H), 2.42 (d, J = 2.8 Hz, 3H), 2.18 – 1.92 (m, 4H), 1.59 – 1.45 (m, 6H).

^{13}C NMR (126 MHz, DMSO-d_6, 75 °C) δ 149.2, 142.7, 139.0, 138.3, 137.5, 131.13, 130.1, 129.5, 129.4, 127.5, 126.7, 125.9, 112.2, 110.5, 51.5 (br), 36.0 (br), 30.9, 30.5, 27.2, 26.8, 25.7, 20.5.

FTIR (Neat film NaCl): 2922, 2850, 1604, 1505, 1483, 1445, 1341, 1156, 1091, 869, 716, 651, 569, 548.

HR-MS (ESI-MS) m/z: [M+H]+ Calc'd for $C_{30}H_{35}N_2O_2S$ 487.2419; Found 487.2413.

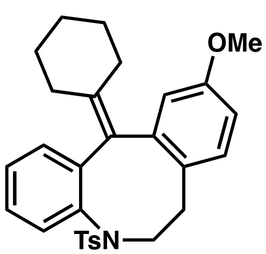

12-cyclohexylidene-10-methoxy-5-tosyl-5,6,7,12-tetrahydrodibenzo[*b,e*]azocine (12).

Synthesized according to general procedure 6 starting from the corresponding vinyl tosylate **SI-27** (32.3 mg, 0.0500 mmol). Crude product was purified via flash column chromatography using 10% ethyl ether in hexanes to give the product **12** as a white powder (0.018 g, 76% yield).

*NMR had poor resolution at room temperature, so NMRs are reported below at 75 °C in DMSO-*d*6.

^{1}H NMR (500 MHz, DMSO-*d*6, 75 °C) δ 7.73 (d, *J* = 7.6 Hz, 2H), 7.41 (d, *J* = 7.8 Hz, 2H), 7.32 (d, *J* = 7.6 Hz, 1H), 7.23 (dd, *J* = 7.5, 7.5 Hz, 1H), 7.12 (ddd, *J* = 7.6, 7.6, 1.6 Hz, 1H), 6.98 (d, *J* = 8.2 Hz, 1H), 6.93 (d, *J* = 8.0 Hz, 1H), 6.68 (s, 1H), 6.65 (d, *J* = 8.2 Hz, 1H), 4.41 (br s, 1H), 3.68 (s, 3H), 3.21 (br s, 1H), 2.75 (br s, 1H), 2.48 (m, 1H), 2.41 (s, 3H), 2.16-2.02 (m, 2H), 2.02-1.90 (m, 2H), 1.74 (br s, 1H), 1.58-1.44 (m, 5H).

^{13}C NMR (126 MHz, DMSO-*d*6, 75°C) δ 158.6, 143.6, 139.9, 139.0, 138.9, 131.4, 131.2, 131.1, 130.4, 130.2, 128.4, 127.8, 127.6, 114.8, 112.3, 55.6, 52.0, 36.9, 31.7, 31.3, 27.9, 27.6, 26.5, 21.4.

FTIR (Neat film NaCl): 2922, 2851, 1602, 1572, 1484, 1446, 1343, 1157, 1093, 1039, 874, 813, 737, 717, 651, 569, 549.

HR-MS (ESI-MS) m/z: [M+Na]+ Calc'd for $C_{29}H_{31}NO_3SNa$ 496.1922; Found 496.1930.

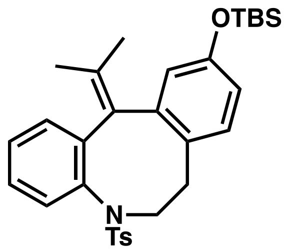

10-((*tert*-butyldimethylsilyl)oxy)-12-(propan-2-ylidene)-5-tosyl-5,6,7,12-tetrahydrodibenzo[b,e]azocine (13).

Synthesized according to general procedure 6 starting from the corresponding vinyl tosylate **SI-33** (35.3 mg, 0.0500 mmol). Crude product was purified via flash column chromatography using 10% ethyl ether in hexanes to give the product **13** as a white powder (0.021 g, 79% yield).

^{1}H NMR (400 MHz, $CDCl_3$) δ 7.83 (br s, 2H), 7.39 – 7.17 (m, 4H), 7.09 (t, *J* = 7.5 Hz, 1H), 6.99 – 6.81 (m, 2H), 6.64 – 6.48 (m, 2H), 4.21 (br s, 1H), 3.33 (br s, 1H), 2.83 (br s, 1H), 2.68 (br s, 1H), 2.44 (s, 3H), 1.77 (s, 3H), 1.65 (s, 3H), 0.96 (s, 9H), 0.16 (s, 6H).

^{13}C NMR (101 MHz, $CDCl_3$) δ 154.7, 143.5, 139.9, 139.2, 134.0, 132.8, 132.1, 131.2, 130.4, 130.0, 129.0, 128.0, 127.7, 125.9, 120.8, 118.4, 52.4, 38.1, 26.1, 22.1, 21.9, 21.3, 18.6, –4.1.

FTIR (Neat film NaCl): 2955, 2928, 2856, 1600, 1489, 1347, 1258, 1159, 1093, 877.

HR-MS (ESI-MS) m/z: [M+NH$_4$]+ Calc'd for $C_{31}H_{43}N_2O_3SSi$ 551.2764; Found 551.2759.

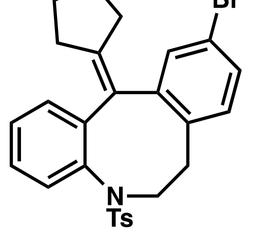

10-bromo-12-cyclopentylidene-5-tosyl-5,6,7,12-tetrahydrodibenzo[b,e]azocine (15).

Synthesized according to general procedure 6 starting from the corresponding vinyl tosylate **SI-36** (34.0 mg, 0.0500 mmol). Crude product was purified via flash column chromatography using 10% ethyl ether in hexanes to give the product **15** as a white powder (0.020 g, 79% yield).

*NMR had poor resolution at room temperature, so NMRs are reported below at 75 °C in DMSO-*d*6.

^{1}H NMR (500 MHz, DMSO-*d*6, 75 °C) δ 7.73 – 7.59 (m, 2H), 7.38 (dd, *J* = 7.6, 1.7 Hz, 3H), 7.33 (s, 1H), 7.28 (dd, *J* = 8.1, 2.2 Hz, 1H), 7.25 (t, *J* = 7.5 Hz, 1H), 7.15 (td, *J* = 7.7, 1.6 Hz, 1H), 7.06 (d, *J* = 8.1 Hz, 2H), 3.82 (br s, 2H), 2.99 (br s, 2H), 2.39 (s, 3H), 2.23 (br s, 2H), 2.00 (br s, 2H), 1.64 (app s, 4H). **CH2 next to nitrogen is very broad and hard to see/integrate

^{13}C NMR (126 MHz, DMSO-*d*6, 75 °C) δ 142.9, 138.9, 132.1, 130.5, 129.68, 129.4, 129.2, 128.9, 127.2, 126.8, 119.3, 50.5 (br), 35.9 (br), 30.8, 30.6, 29.6, 25.6, 25.5, 20.6.

FTIR (Neat film NaCl): 2952, 2924, 2857, 1454, 1347, 1158, 1091, 1077, 717, 568.

HR-MS (ESI-MS) m/z: [M+Na]$^+$ Calc'd for $C_{27}H_{26}BrNO_2SNa$ 530.0765; Found 530.0781.

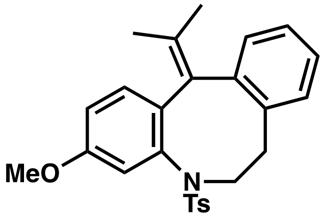

3-methoxy-12-(propan-2-ylidene)-5-tosyl-5,6,7,12-tetrahydrodibenzo[*b*,*e*]azocine (16)

Synthesized according to general procedure 6 at 140 °C for 24 hours starting from the corresponding vinyl tosylate **SI-46** (30.3 mg, 0.0500 mmol). Crude product was purified *via* flash column chromatography using 3:1 hexanes:diethyl ether to give the product **16** as white solid (0.017 g, 78% yield).

^{1}H NMR (500 MHz, $CDCl_3$) δ 7.85 (br s, 2H), 7.35 (br s, 2H), 7.21–6.92 (m, 5H), 6.79 (br s, 1H), 6.44 (br s, 1H), 4.27 (br s, 1H), 3.64 (s, 3H), 3.46 (br s, 1H), 2.89–2.69 (m, 2H), 2.50–2.22 (m, 3H), 1.80 (s, 3H), 1.62 (s, 3H).

^{13}C NMR (101 MHz, $CDCl_3$) δ 157.4, 142.2, 131.9, 129.5, 128.6, 127.7, 126.7, 126.6, 126.0, 125.6, 113.4, 112.3, 54.2, 50.7, 37.7, 20.7, 20.5, 20.0.

FTIR (Neat film NaCl): 2989, 2870, 1605, 1495, 1443, 1381, 1347, 1287, 1157, 1143, 1091, 813, 688, 584.

HR-MS (ESI-MS) m/z: [M]$^+$ Calc'd for $C_{26}H_{27}NO_3SNa$ 456.1609; Found 456.1596.

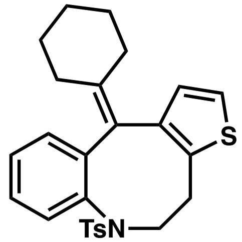

11-cyclohexylidene-6-tosyl-4,5,6,11-tetrahydrobenzo[*b*]thieno[3,2-*e*]azocine (18).

Synthesized according to general procedure 6 at 140 °C for 20 hours starting from the corresponding vinyl tosylate **SI-38** (31.1 mg, 0.0500 mmol). Crude product was purified *via* flash column chromatography using a gradient of 0-30% diethyl ether in hexanes to give the product **18** as white solid (16.5 mg, 73% yield).

^{1}H NMR (500 MHz, $CDCl_3$) δ 7.79 (d, *J* = 8.3 Hz, 2H), 7.34 (d, *J* = 8.0 Hz, 2H), 7.26 – 7.20 (m, 2H), 7.14 – 7.04 (m, 1H), 6.92 (d, *J* = 5.1 Hz, 1H), 6.88 – 6.82 (m, 1H), 6.77 (d, *J* = 5.1 Hz, 1H), 4.15 (ddd, *J* = 14.5, 5.5, 2.1 Hz, 1H), 3.48 (ddd, *J* = 15.8, 10.5, 2.1 Hz, 1H), 2.98 (dd, *J* = 14.4, 10.3 Hz, 1H), 2.81 (dd, *J* = 15.7, 5.3 Hz, 1H), 2.46 (s, 3H), 2.27 – 2.14 (m, 3H), 2.12 (dd, *J* = 8.4, 4.4 Hz, 1H), 1.87 – 1.79 (m, 1H), 1.73 – 1.65 (m, 1H), 1.63 – 1.48 (m, 4H).
^{13}C NMR (126 MHz, Chloroform-*d*) δ 145.9, 143.2, 142.0, 141.0, 139.3, 138.4, 135.0, 130.3, 130.2, 129.6, 128.6, 128.1, 127.6, 127.4, 125.5, 120.8, 51.4, 32.0, 31.6, 31.1, 28.4, 27.9, 26.6, 21.5.
FTIR (Neat film NaCl): 3062, 2921, 2851, 1598, 1483, 1343, 1157, 1094, 864, 737, 726, 661.

HR-MS (ESI-MS) m/z: [M+H]+ Calc'd for $C_{26}H_{28}NO_2S_2$ 450.1561; Found 450.1562.

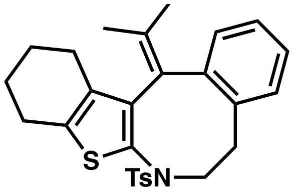

13-(propan-2-ylidene)-7-tosyl-5,6,7,9,10,11,12,13-octahydrobenzo[*e*]benzo[4,5]thieno[2,3-*b*]azocine (19).

Synthesized according to general procedure 6 at 120 °C for 20 hours starting from the corresponding vinyl tosylate **SI-42** (31.8 mg, 0.0500 mmol). Crude product was purified *via* flash column chromatography using a gradient of 1-20% diethyl ether in hexanes to give the product **19** as white solid (19.6 mg, 85% yield).

^{1}H NMR (500 MHz, $CDCl_3$) δ 7.84 (d, *J* = 7.8 Hz, 2H), 7.32 (d, *J* = 7.9 Hz, 2H), 7.21 – 7.04 (m, 4H), 4.26 (dd, *J* = 15.1, 7.1 Hz, 1H), 3.41 (dd, *J* = 15.0, 9.7 Hz, 1H), 3.00 – 2.71 (m, 2H), 2.71 – 2.47 (m, 4H), 2.44 (s, 3H), 1.86 – 1.79 (m, 2H), 1.75 (s, 3H), 1.75 – 1.67 (m, 2H), 1.60 (s, 3H).

^{13}C NMR (126 MHz, $CDCl_3$) δ 143.6, 143.4, 143.1, 139.1, 137.8, 134.8, 133.7, 132.9, 132.7, 130.4, 129.6, 128.9, 128.39, 127.6, 126.8, 126.7, 51.7, 38.9, 25.1, 25.0, 23.3, 22.7, 21.6, 21.3, 21.0.

FTIR (Neat film NaCl): 3059, 2986, 2929, 2857, 2843, 1484, 1441, 1341, 1159, 1091, 731, 659, 545.

HR-MS (ESI-MS) m/z: [M+Na]+ Calc'd for $C_{27}H_{29}NO_2S_2Na$ 486.1537; Found 486.1538.

4. Reaction condition screen

Catalyst and catalyst loading screen

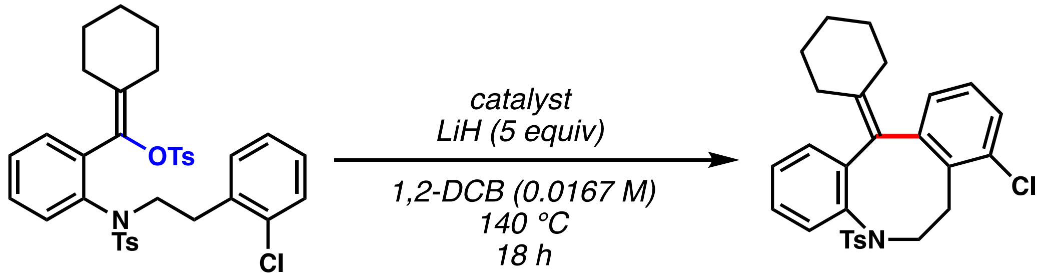

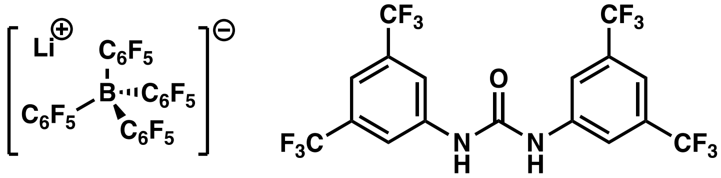

$[Li]^+ [B(C_6F_5)_4]^-$ Schreiner's catalyst

entry	catalyst	catalyst loading (mol%)	yield
1	$[Li]^+[B(C_6F_5)_4]^-$	1	24
2	$[Li]^+[B(C_6F_5)_4]^-$	5	49
3	$[Li]^+[B(C_6F_5)_4]^-$	10	73
4	$[Li]^+[B(C_6F_5)_4]^-$	20	71
5	Schreiner's catalyst	10	19
6	none	0	0

10 mol% of $[Li]^+[B(C_6F_5)_4]^-$ was found to efficiently catalyze the reaction. A lower loading would give a decreased yield, while a higher loading of 20 mol% would give a similar yield.

Base and base loading screen

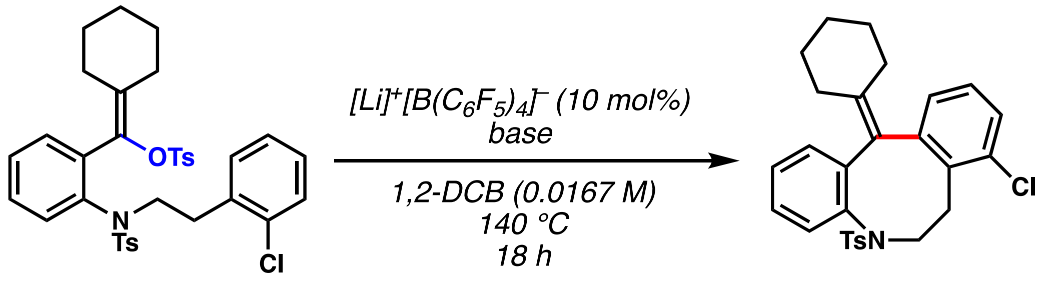

entry	base	base loading (eq)	yield
1	LiH	0.5	54
2	LiH	1	58
3	LiH	5	73

4	LiH	10	67
5	LiHMDS	1.5	21
6	none	0	40

5 equiv of LiH was found to give the highest yield of the product. The reaction had higher yield compared to when LiHMDS or no base was used.

Solvent and concentration screen

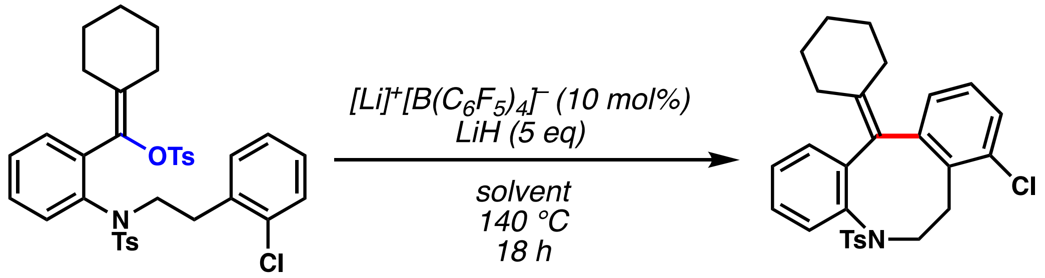

entry	solvent	Concentration (M)	Temperature (°C)	yield
1	1,2-difluorobenzene	0.0167	92	0
2	mesitylene	0.0167	140	50
3	dimethylformamide	0.0167	140	0
4	1,2-dichlorobenzene	0.0167	140	73
5	1,2-dichlorobenzene	0.025	140	71
6	1,2-dichlorobenzene	0.05	140	75
7	1,2-dichlorobenzene	0.1	140	58
8	1,2-dichlorobenzene	0.0167	120	0

Under lower temperature the reaction could not happen (entry 1 and 8), so solvents with lower boiling points were not ideal. Although the reaction could happen in mesitylene (b.p. = 165 °C), the yield was lower and the transformation was not clean, presumably because the electron-rich mesitylene could react with the vinyl cation intermediates through intermolecular Friedel–Crafts reactions. The reaction would not happen in polar solvent such as N,N-dimethylformamide, presumably because lithium cations were not acidic enough to initiate the reaction due to coordination from the polar solvent.

The yields of the reaction were similar in the 0.0167–0.05 M concentration range. But when the concentration was higher (0.1 M, entry 7), the yield started to decrease.

5. Mechanism studies

5.1 8-membered ring formation vs 7-membered ring formation

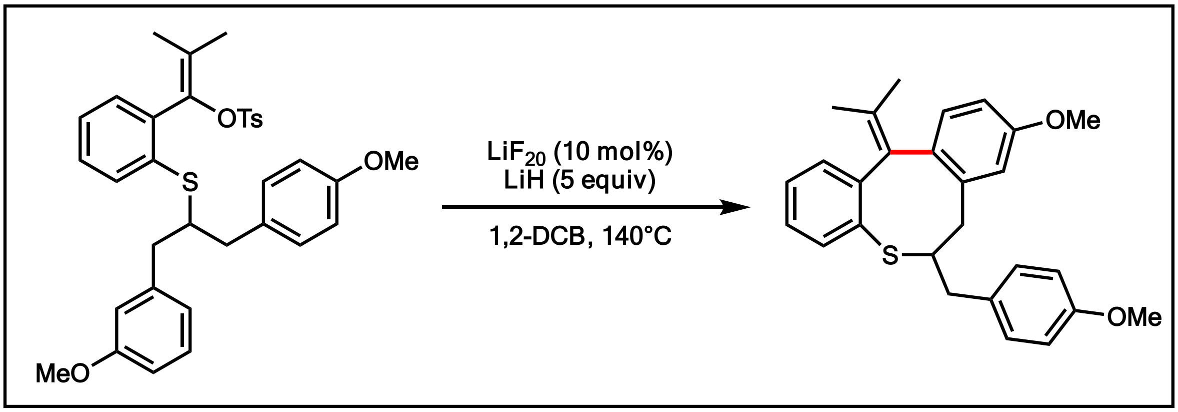

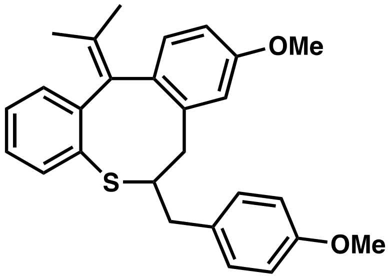

9-methoxy-6-(4-methoxybenzyl)-12-(propan-2-ylidene)-7,12-dihydro-6*H*-dibenzo[*b*,*e*]thiocine (29a)

In the glovebox, lithium tetrakis(pentafluorophenyl)borate (3.4 mg, 0.0050 mmol, 0.1 equiv), lithium hydride (2.0 mg, 0.25 mmol, 5.0 equiv), and vinyl tosylate **28** (29.4 mg, 0.0500 mmol, 1.0 equiv) were dissolved into 1,2-dichlorobenzene (2.5 mL) to generate a 0.02 M solution for the vinyl tosylate. The reaction was heated under 140 °C overnight. The reaction solution was directly purified *via* flash column chromatography using hexanes and then ethyl acetate to get rid of 1,2-dichlorobenzene. Then the crude product was purified *via* preparative TLC to get the product. The yield was determined by NMR with nitromethane as the internal standard from the crude reaction.

^{1}H NMR (400 MHz, $CDCl_3$) δ 7.24 – 7.13 (m, 2H), 7.09 – 7.01 (m, 3H), 6.99 – 6.89 (m, 1H), 6.83 (d, J = 8.4 Hz, 2H), 6.78 – 6.58 (m, 2H), 6.56 – 6.37 (m, 1H), 4.05–3.90 (m, 1H), 3.79 (s, 3H), 3.76 – 3.66 (m, 3H), 3.36 – 3.17 (m, 1H), 2.91 – 2.64 (m, 3H), 1.70 (br, 3H), 1.60 – 1.56 (m, 3H).

IR (Neat film NaCl): 2907, 2851, 2834, 1603, 1583, 1511, 1496, 1463, 1440, 1245, 1177, 1155, 1114, 1035, 807, 752.

HR-MS (FD-MS) m/z: $[M]^+$ Calc'd for $C_{27}H_{28}O_2S$ 416.1810; Found 416.1804.

* ^{13}C NMR was complicated due to the existence of rotamers.

Despite the reaction forming **29a/b** is complicated with multiple products, LC-MS shows the isolated **29a** is the major Friedel-Crafts product (MW = 417) based on the UV (second row, **Figure S1**) and EIE chromatogram (third row, **Figure S1**).

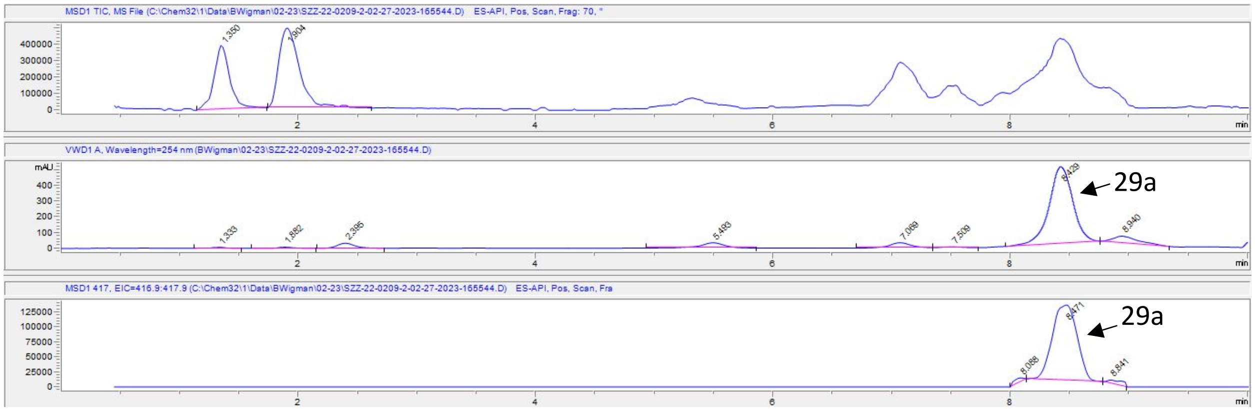

Figure S1. The LC-MS trace of the reaction forming **29a**. (first row: TIC chromatogram; second row: UV (254 nm) chromatogram; third row: EIC (MW = 417) chromatogram; method: 75%:25% water:ACN (0.01 min) to 26%:74% water:ACN (9.80 min) to 100% ACN (9.81 min))

The ^{1}H NMR of **29a** is also complicated. A zoom-in into the aromatic region of the ^{1}H NMR shows there are two characteristic doublets each with 2 protons (one is overlapped with one other proton signal) which is corresponding to a *para*-substituted aromatic ring. This pattern is only seen if **29a** is formed, instead of **29b** which has no *para*-substituted aromatic rings.

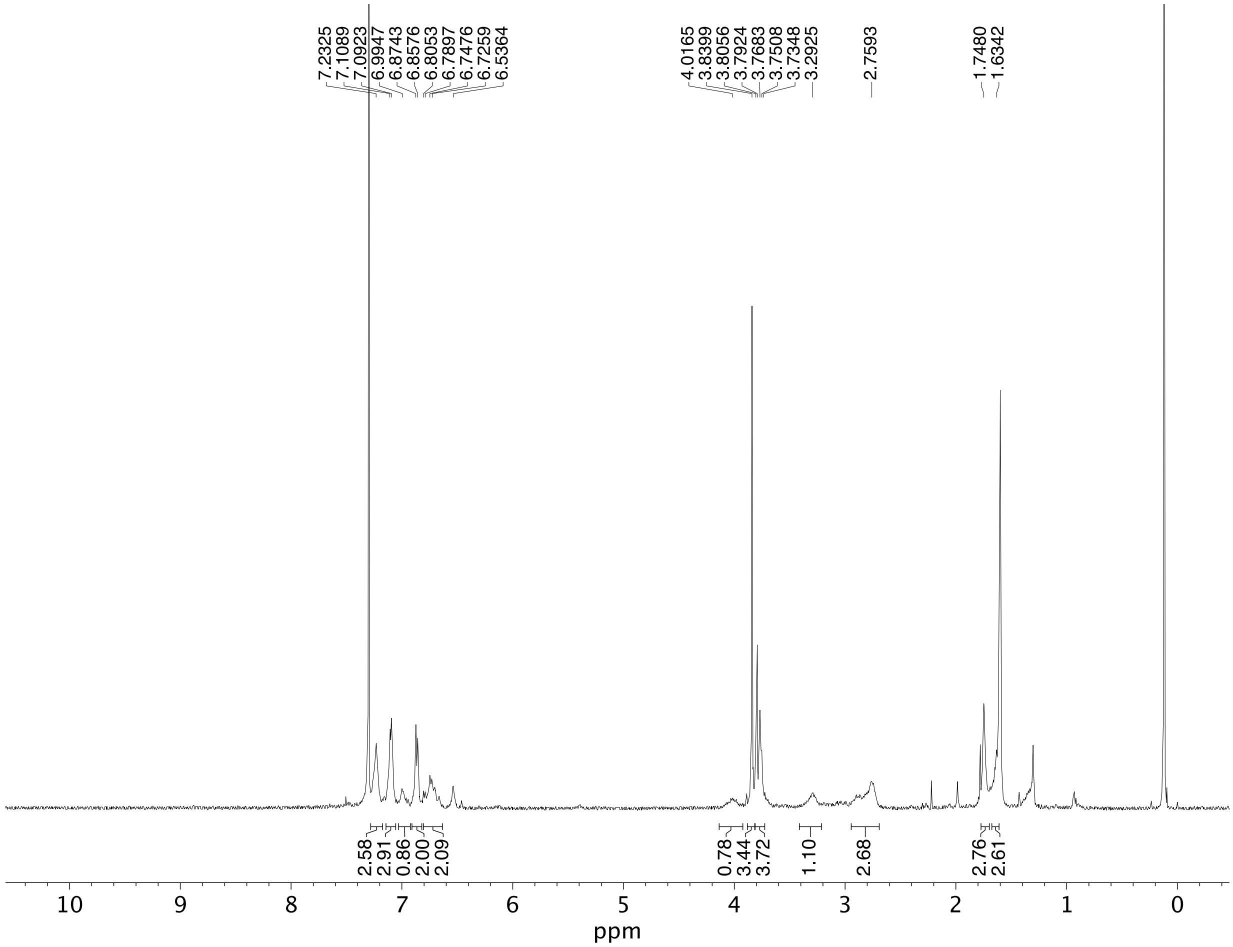

^{1}H NMR (400 MHz, $CDCl_3$) of compound **29a**.

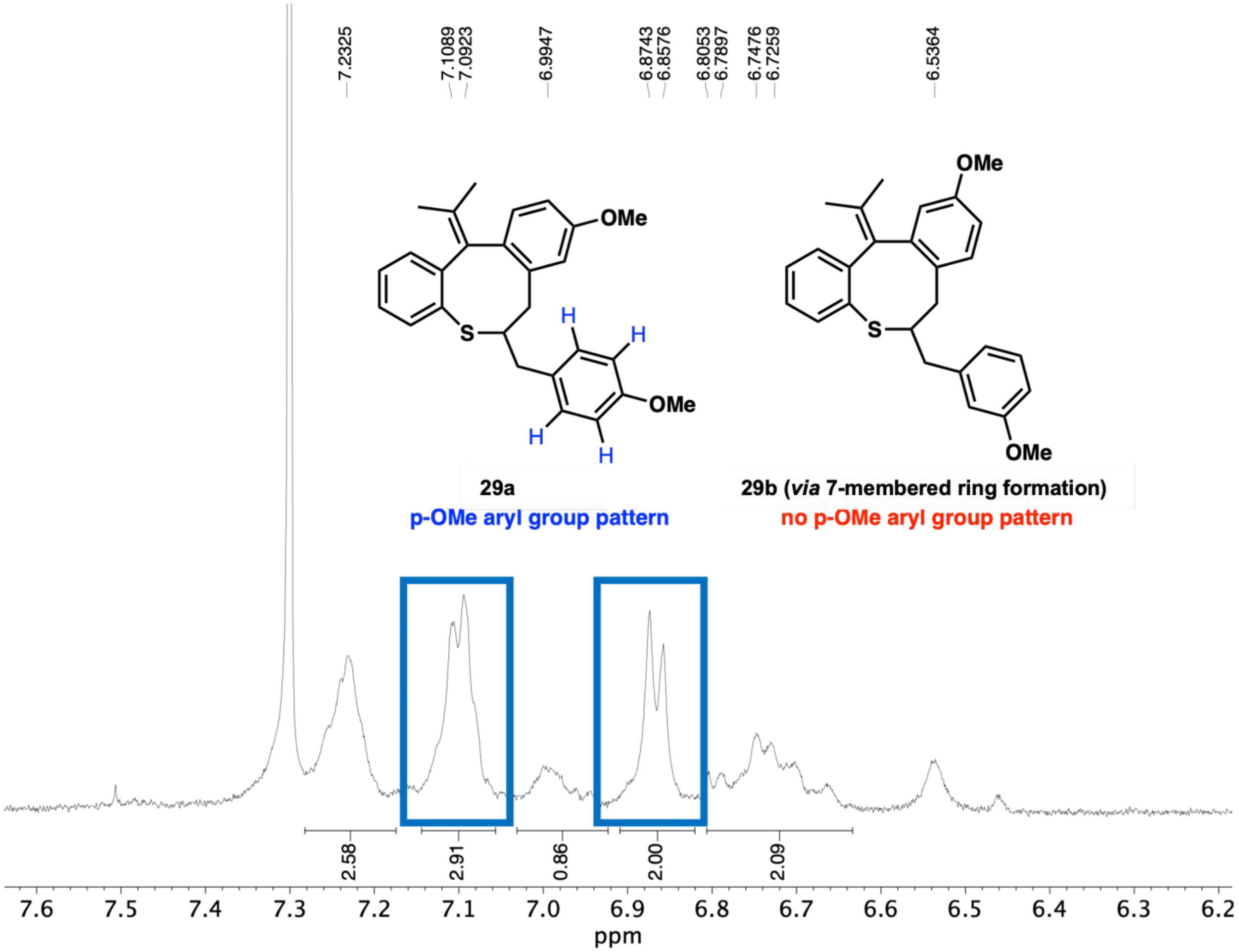

^{1}H NMR (400 MHz, $CDCl_3$) of compound **29a** (zoom-in of 7.7–6.1 ppm).

5.2 Friedel-Crafts reactions vs C–H insertions

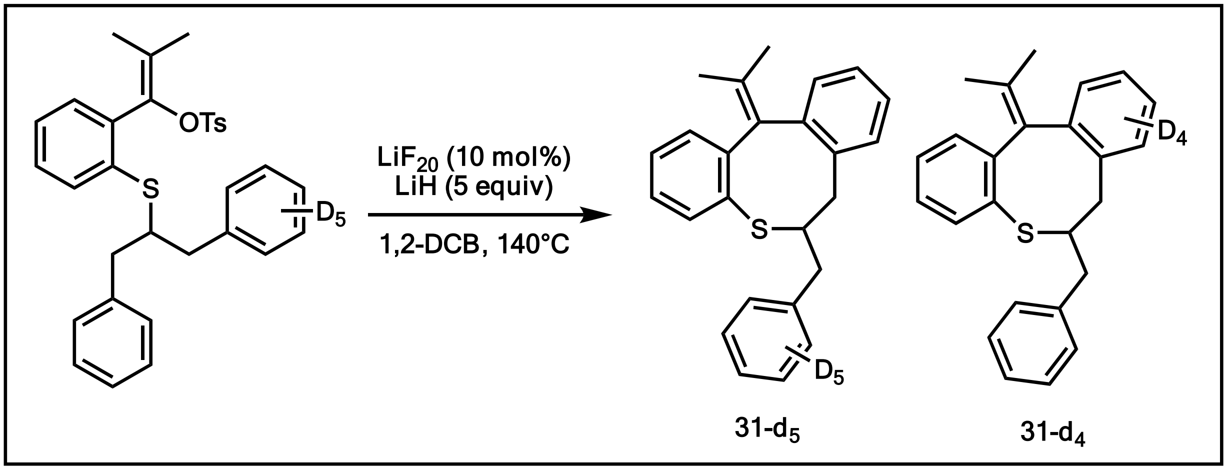

In the glovebox, lithium tetrakis(pentafluorophenyl)borate (3.4 mg, 0.0050 mmol, 0.1 equiv), lithium hydride (2.0 mg, 0.25 mmol, 5.0 equiv), and vinyl tosylate **30** (26.7 mg, 0.0500 mmol, 1.0 equiv) were dissolved into 1,2-dichlorobenzene (2.5 mL) to generate a 0.02 M solution for the vinyl tosylate. The reaction was heated under 140 °C overnight. The reaction solution was directly purified *via* flash column chromatography using hexanes and then ethyl acetate to get rid of 1,2-dichlorobenzene. Then the crude product was purified *via* preparative TLC to get the product.

^{1}H NMR shows the product is the desired 8-membered ring product from the Friedel-Crafts reaction.

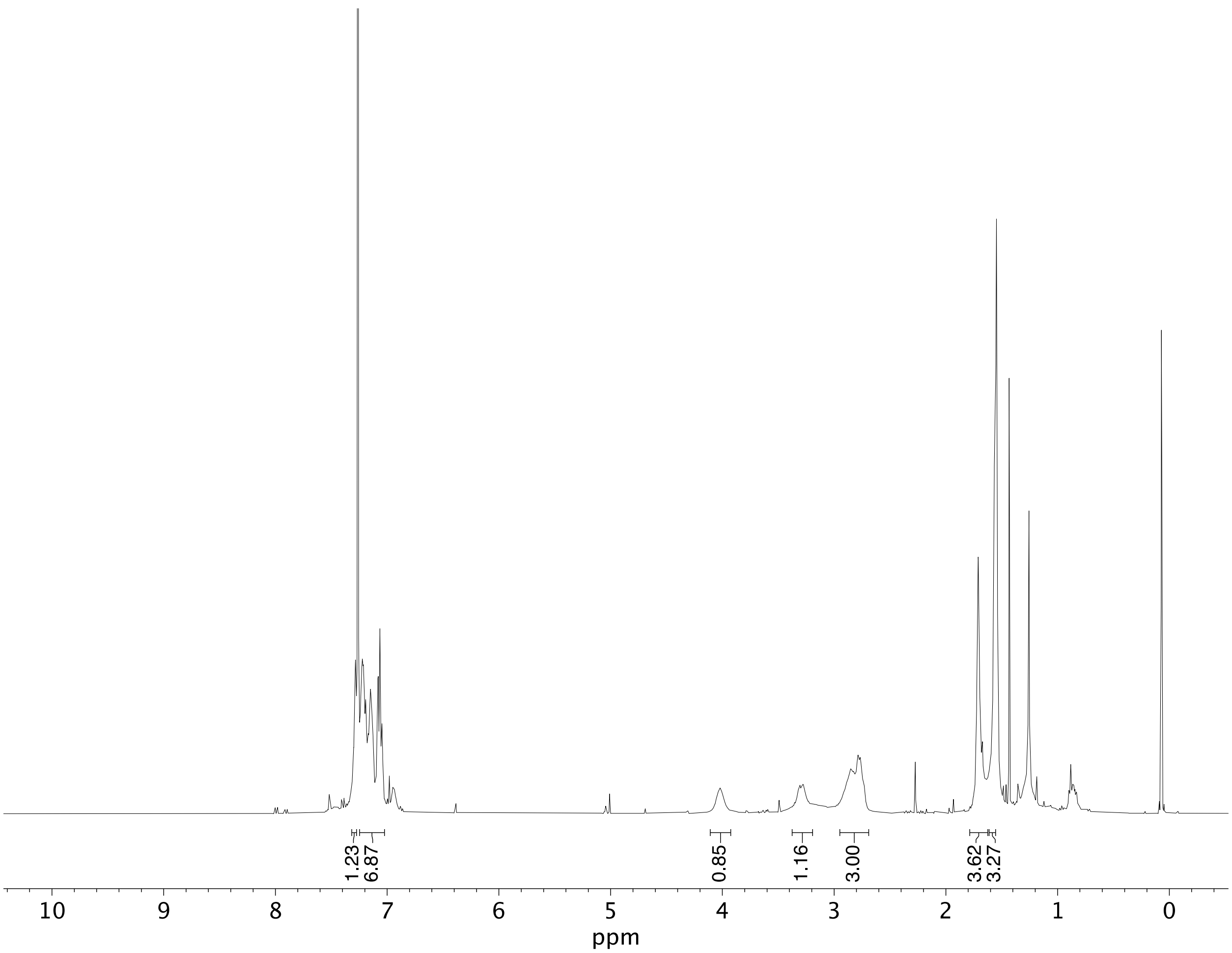

^{1}H NMR (400 MHz, $CDCl_3$) of the mixture product **31-d$_5$** and **31-d$_4$**.

The HR-MS of this **31-d₅** and **31-d₄** mixture showed their ratio is around 1:1.

Spectrum

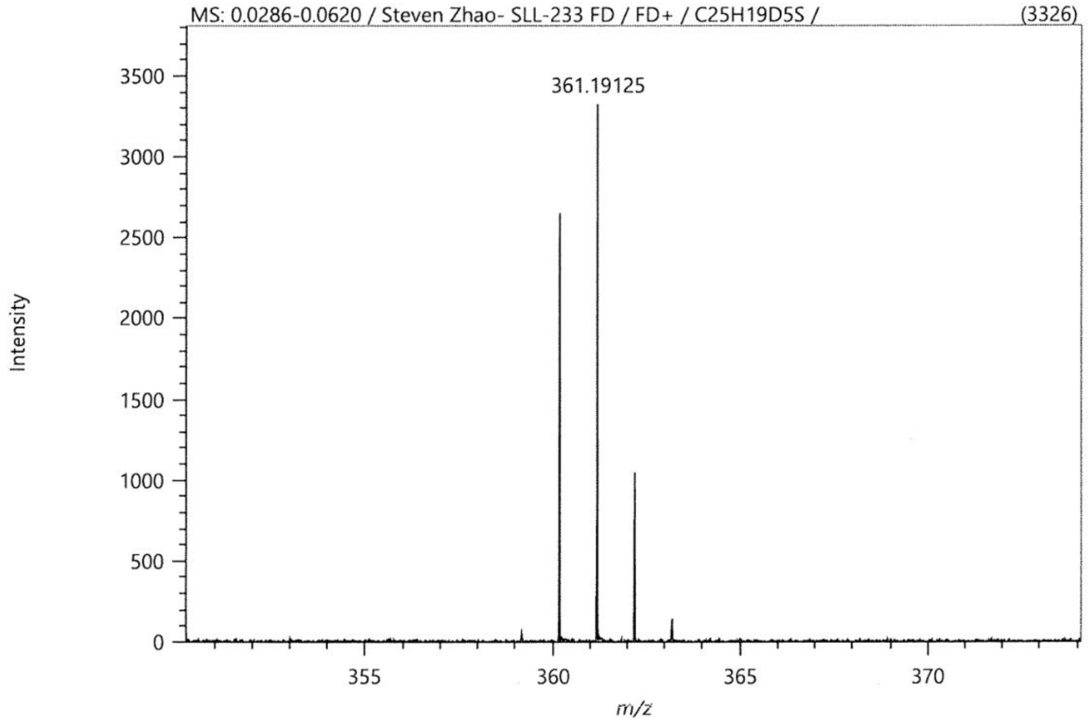

Elemental Composition

Parameters		Elements Set 1:					
Tolerance:	±3.00 ppm	Symbol	C	H	O	Hx	S
Electron:	Odd/Even	Min	0	0	0	0	1
Charge:	0	Max	100	50	2	5	1
DBE:	-1.5 - 100.0						

Results

Mass	Intensity	Formula	Calculated Mass	Mass Difference [mDa]	Mass Difference [ppm]	DBE
360.18528	2654.29	C25 H20 S Hx4	360.18498	0.30	0.83	14.0
361.19125	3325.59	C25 H19 S Hx5	361.19126	0.00	-0.01	14.0

6. Computational Studies

6.1 Computational Methods

Density functional theory (DFT) computations were performed with Gaussian 16.[16] The ωB97X-D functional was used to optimize molecular geometries.[17] Geometry optimizations were completed with the def2-SVP basis set[18] and the SMD solvation model accounting for the effect of *o*-dichlorobenzene.[19] Frequency calculations were conducted at the same level of theory used for the geometry optimizations in order to obtain thermal Gibbs free energies and characterize the stationary points on the potential energy surface. Single point energies were obtained using the def2-TZVPP basis set.[18] Intrinsic reaction coordinate (IRC) calculations were performed to verify that a transition state (TS) connects the reactant and the product on the potential energy surface.

Conformation searches were executed by Grimme's Conformer-Rotamer Ensemble Sampling Tool (CREST) to find the lowest energy conformers.[20] CYLview was employed to visualize molecular structures.[21]

6.2 Energies and Cartesian coordinates of calculated structures

Table S1. Energies of the optimized structures (□B97X-D/def2-SVP/SMD=o-dichlorobenzene)

Structure	E	ZPE	H	T.S	G(T)
INT1	-1684.511784	0.488122	-1683.967990	0.159247	-1684.127237
INT2-m	-1684.517623	0.488675	-1683.974546	0.154411	-1684.128957
INT2-p	-1684.540119	0.491438	-1683.994980	0.151622	-1684.146602
TS-R	-1684.510634	0.489840	-1683.967969	0.148111	-1684.116080
TS-m	-1684.487656	0.487587	-1683.945750	0.154876	-1684.100626
TS-p	-1684.487267	0.487612	-1683.945149	0.155956	-1684.101105
INT1'	-1684.514887	0.488449	-1683.971117	0.157303	-1684.128420
INT2-p'	-1684.543812	0.491621	-1683.998548	0.151343	-1684.149891
TS-p'	-1684.493320	0.488175	-1683.950954	0.15397	-1684.104924

Table S2. Single point energies (□B97X-D/def2-TZVPP/SMD=o-dichlorobenzene)

Structure	E
INT1	-1686.093476
INT2-m	-1686.094830
INT2-p	-1686.117135
TS-R	-1686.088536
TS-m	-1686.069921
TS-p	-1686.070019
INT1'	-1686.094658

INT2-p’	-1686.122058
TS-p’	-1686.076146

6.3 Cartesian coordinates for structures of Table S1, & Table S2

INT1

Charge: 1

C	1.010925	2.218217	-1.610785
C	0.888011	3.600868	-1.684825
C	0.210187	4.350815	-0.706262
C	-0.374103	3.697303	0.351702
C	-0.316478	2.271450	0.429751
C	0.444571	1.523471	-0.537879
N	0.574344	0.151369	-0.403093
S	1.240534	-0.519113	1.029589
O	1.194203	0.518883	2.049986
O	0.585558	-1.794229	1.246058
C	2.929708	-0.785623	0.579620
C	3.371549	-2.078045	0.312049
C	4.701369	-2.267857	-0.057593
C	5.584951	-1.187605	-0.163886
C	5.105490	0.103562	0.114899
C	3.786097	0.315549	0.489808
H	3.430847	1.322746	0.716836
H	5.784797	0.956458	0.043497
C	7.021660	-1.390179	-0.553025
H	7.223482	-2.434283	-0.827385
H	7.687923	-1.119712	0.281271
H	7.291539	-0.746886	-1.404319
H	5.057500	-3.279536	-0.265035
H	2.688551	-2.924943	0.395747
C	0.712378	-0.702418	-1.594856
H	0.790888	-1.735254	-1.232421
C	-0.477762	-0.578117	-2.540632

C	-1.098495	1.623801	1.343579
C	-1.973173	1.084349	2.132742
C	-3.414738	1.027430	1.680255
C	-1.629881	0.505254	3.478936
H	-0.583323	0.672749	3.751446
H	-1.824503	-0.577544	3.443986
H	-4.016044	1.643889	2.365127
H	-3.552030	1.384320	0.652382
H	-0.462577	0.401803	-3.040463
H	-0.312790	-1.321454	-3.338441
H	1.652051	-0.484128	-2.127471
H	-0.917341	4.238263	1.128153
H	0.152969	5.436495	-0.790447
H	1.361960	4.121703	-2.519950
H	1.580810	1.686461	-2.372265
C	-1.834140	-0.787041	-1.905689
C	-2.066767	-1.776631	-0.947512
C	-2.922183	0.008100	-2.293961
C	-3.332292	-1.981330	-0.394548
H	-1.246763	-2.406969	-0.596763
C	-4.189605	-0.182453	-1.757917
H	-2.772852	0.796478	-3.037410
C	-4.406662	-1.179930	-0.797314
H	-3.461738	-2.762167	0.355452
H	-5.030543	0.443607	-2.063332
H	-2.294354	0.948427	4.235009
H	-3.764044	-0.012269	1.751470
O	-5.656905	-1.278375	-0.304044
C	-5.934805	-2.265893	0.658738
H	-5.336706	-2.126185	1.575709
H	-6.997576	-2.166593	0.912768
H	-5.756794	-3.281764	0.266712

There are no imaginary frequencies

INT2-m

Charge: 1

C	1.321834	2.259574	-1.336273
C	0.919234	3.493721	-1.840293
C	-0.383603	3.942277	-1.622329
C	-1.280294	3.157272	-0.899534
C	-0.882914	1.927444	-0.365420
C	0.431861	1.482840	-0.592325
N	0.807780	0.204790	-0.085104
S	1.832928	0.088172	1.220559
O	2.066516	1.442537	1.698495
O	1.293378	-0.926627	2.118241
C	3.361183	-0.547587	0.569836
C	4.306478	0.345254	0.061170
C	5.490189	-0.153149	-0.469991
C	5.749505	-1.532447	-0.499073
C	4.786237	-2.403191	0.024924
C	3.595103	-1.920984	0.563489
H	2.857753	-2.606563	0.984278
H	4.972415	-3.479923	0.018009
C	7.046987	-2.051496	-1.052754
H	7.319974	-1.530139	-1.982038
H	6.996131	-3.129771	-1.257334
H	7.864903	-1.884023	-0.333277
H	6.234095	0.542863	-0.865829
H	4.119562	1.420377	0.091749
C	0.461962	-1.046179	-0.765712
H	0.258235	-1.812196	-0.003403
C	-0.721099	-0.961125	-1.735958
C	-1.830311	1.042812	0.375746
C	-1.842634	0.938545	1.718293
C	-2.649723	-0.076187	2.479912
C	-1.016081	1.837009	2.592490
H	-1.660407	2.284902	3.366766
H	-0.515464	2.640218	2.039340

H	-2.028512	-0.496153	3.285925
H	-3.523601	0.391197	2.962269
H	-0.620805	-0.067107	-2.370902
H	-0.618921	-1.824746	-2.405347
H	1.323971	-1.391586	-1.362799
H	-2.302953	3.505553	-0.733977
H	-0.704492	4.907628	-2.019762
H	1.622093	4.102388	-2.413040
H	2.332351	1.889121	-1.517057
C	-2.112916	-0.999418	-1.170088
C	-2.913650	-2.102460	-1.391330
C	-2.721524	0.173879	-0.507001
C	-4.256432	-2.138932	-0.995420
H	-2.503070	-2.974017	-1.904754
C	-4.113275	0.055796	-0.075242
H	-2.915441	0.786787	-1.440008
C	-4.887211	-1.051922	-0.356033
H	-4.823826	-3.045681	-1.214977
H	-4.561144	0.907250	0.441873
H	-0.245481	1.249584	3.113838
H	-2.997748	-0.917891	1.865022
O	-6.165977	-1.031629	0.024720
C	-6.985995	-2.159648	-0.220877
H	-7.977400	-1.908388	0.172891
H	-6.608341	-3.053719	0.301288
H	-7.070168	-2.367452	-1.299929

There are no imaginary frequencies

INT2-p

Charge: 1

C	1.740215	2.398813	-0.817479
C	1.593455	3.784488	-0.865930
C	0.378507	4.365953	-0.503788

C	-0.685113	3.569459	-0.079027
C	-0.546053	2.180434	0.001746
C	0.677889	1.610361	-0.381259
N	0.753531	0.188630	-0.385799
S	1.433502	-0.621936	0.890869
O	1.468681	0.302999	2.012076
O	0.735735	-1.901098	1.006589
C	3.104931	-0.969233	0.403403
C	3.384893	-2.126565	-0.322042
C	4.689759	-2.356906	-0.748160
C	5.719354	-1.451584	-0.456651
C	5.407118	-0.299892	0.280229
C	4.108813	-0.051901	0.714306
H	3.875767	0.842935	1.293905
H	6.197442	0.415094	0.522067
C	7.132451	-1.723699	-0.891432
H	7.663793	-0.791986	-1.133023
H	7.163564	-2.384512	-1.769085
H	7.692144	-2.220259	-0.081856
H	4.914382	-3.262922	-1.316423
H	2.593815	-2.844668	-0.545002
C	0.084916	-0.509261	-1.475693
H	0.014097	-1.571624	-1.211463
C	-1.297917	0.074414	-1.765617
C	-1.637562	1.296460	0.503406
C	-2.080542	1.405312	1.774162
C	-3.235943	0.621862	2.338892
C	-1.416779	2.300540	2.785746
H	-0.438003	2.673189	2.464629
H	-1.278981	1.738794	3.723645
H	-4.017446	0.397899	1.602782
H	-2.888091	-0.326268	2.781781
H	-1.204684	1.052630	-2.256887
H	-1.809549	-0.591931	-2.475189
H	0.680574	-0.436284	-2.400872
H	-1.633376	4.029406	0.208412

H	0.256122	5.450195	-0.552600
H	2.424969	4.409126	-1.198974
H	2.675512	1.920050	-1.115198
C	-2.233637	0.271849	-0.501148
C	-2.370673	-1.094165	0.071677
C	-3.505870	0.791349	-1.075730
C	-3.461960	-1.877319	-0.108491
H	-1.503390	-1.492271	0.607945
C	-4.615706	0.033352	-1.219405
H	-3.496216	1.822576	-1.438801
C	-4.601709	-1.330210	-0.776110
H	-3.482680	-2.894660	0.279577
H	-5.529355	0.416239	-1.675409
H	-2.063233	3.161076	3.025829
H	-3.709372	1.193410	3.151022
O	-5.680055	-2.001275	-1.007329
C	-5.815838	-3.378799	-0.625683
H	-5.744299	-3.476844	0.465711
H	-6.811983	-3.678933	-0.964784
H	-5.049550	-3.987394	-1.124410

There are no imaginary frequencies

TS-R

Charge: 1

C	1.507565	2.369705	-0.722466
C	1.198449	3.692647	-1.028379
C	-0.126734	4.125529	-0.990087
C	-1.138759	3.235715	-0.637921
C	-0.839778	1.914270	-0.291680
C	0.498517	1.484863	-0.340837
N	0.763106	0.111704	-0.073120
S	1.708963	-0.359213	1.215005
O	1.827685	0.795488	2.091786

O	1.148583	-1.609977	1.707728
C	3.314130	-0.722075	0.543230
C	3.565241	-1.981026	-0.000195
C	4.812952	-2.238935	-0.563152
C	5.816355	-1.261980	-0.581046
C	5.539653	-0.009518	-0.011797
C	4.298843	0.267605	0.550338
H	4.096575	1.240789	1.001089
H	6.314336	0.761462	-0.004783
C	7.170002	-1.547003	-1.169002
H	7.168743	-2.477669	-1.752745
H	7.922488	-1.649200	-0.370530
H	7.499394	-0.724729	-1.821549
H	5.012093	-3.223855	-0.992348
H	2.798626	-2.757557	0.024630
C	0.498969	-0.867920	-1.125653
H	0.385620	-1.854877	-0.655473
C	-0.755530	-0.524582	-1.923746
C	-1.939316	0.998874	0.150476
C	-2.125759	0.736781	1.479759
C	-3.173098	-0.151384	2.056446
C	-1.267374	1.400463	2.513933
H	-0.541847	2.116320	2.118050
H	-0.717780	0.623719	3.069268
H	-3.139214	-0.124211	3.152408
H	-4.178918	0.157068	1.730701
H	-0.645307	0.438533	-2.437350
H	-0.849586	-1.285128	-2.715382
H	1.346146	-0.919194	-1.831726
H	-2.178095	3.572658	-0.611256
H	-0.374267	5.159738	-1.237842
H	1.994892	4.383055	-1.313416
H	2.537896	2.015621	-0.783890
C	-2.049635	-0.570949	-1.164012
C	-2.552210	-1.832626	-0.751164
C	-2.927117	0.587043	-1.045857

C	-3.889182	-2.006270	-0.511871
H	-1.883990	-2.695844	-0.741924
C	-4.345698	0.349699	-0.772401
H	-2.775762	1.363623	-1.805335
C	-4.821835	-0.908587	-0.550259
H	-4.248424	-3.013243	-0.294170
H	-5.030421	1.199261	-0.773826
H	-1.921553	1.907089	3.241578
H	-3.041428	-1.198778	1.739346
O	-6.133683	-1.067342	-0.340050
C	-6.676337	-2.360667	-0.151888
H	-6.280080	-2.840618	0.757507
H	-7.757788	-2.222889	-0.035791
H	-6.492064	-3.009032	-1.023823

1 imaginary frequency: –140.91 cm^{-1}

TS-m

Charge: 1

C	1.659123	2.141509	-0.929783
C	1.492579	3.515355	-1.118783
C	0.274249	4.136655	-0.834452
C	-0.789750	3.387624	-0.348538
C	-0.622506	2.010765	-0.116431
C	0.607649	1.383636	-0.429280
N	0.672182	-0.030959	-0.328444
S	1.427139	-0.745150	0.973115
O	1.316441	0.204485	2.070364
O	0.872842	-2.084971	1.083774
C	3.140799	-0.887355	0.534458
C	4.030514	0.113010	0.922302
C	5.362779	0.023056	0.526399
C	5.818130	-1.050994	-0.248925
C	4.900139	-2.047088	-0.616535

C	3.567136	-1.976537	-0.228669
H	2.866465	-2.765639	-0.507387
H	5.239516	-2.897643	-1.213092
C	7.259173	-1.157000	-0.663275
H	7.809437	-0.229646	-0.453367
H	7.346213	-1.379646	-1.737335
H	7.756488	-1.976946	-0.120805
H	6.063991	0.803666	0.831028
H	3.685471	0.950362	1.531098
C	0.426572	-0.798207	-1.548687
H	0.300490	-1.850637	-1.261705
C	-0.819894	-0.296388	-2.276190
C	-1.630891	1.246456	0.538851
C	-2.037764	0.835670	1.716367
C	-3.043306	-0.204859	2.091750
C	-1.365796	1.551216	2.878337
H	-2.158806	1.975975	3.513074
H	-0.679272	2.346821	2.569758
H	-2.931361	-0.450106	3.155370
H	-4.065252	0.169694	1.934330
H	-0.685108	0.749396	-2.588288
H	-0.901659	-0.876838	-3.208604
H	1.291282	-0.728880	-2.231564
H	-1.749140	3.856515	-0.122222
H	0.155883	5.209092	-0.998095
H	2.322721	4.106135	-1.511555
H	2.602151	1.654566	-1.185183
C	-2.114563	-0.446282	-1.520394
C	-2.609605	-1.723512	-1.213762
C	-2.957723	0.663060	-1.252688
C	-3.903320	-1.908333	-0.752258
H	-1.984479	-2.600278	-1.398507
C	-4.272120	0.477047	-0.785060
H	-2.694346	1.643975	-1.651176
C	-4.752829	-0.801160	-0.531645
H	-4.253918	-2.922783	-0.559331

H	-4.925845	1.335295	-0.621523
H	-0.804876	0.803959	3.456987
H	-2.906330	-1.118242	1.502651
O	-6.004689	-0.899974	-0.064022
C	-6.548566	-2.174208	0.209694
H	-7.569201	-2.003181	0.572537
H	-5.975693	-2.700547	0.990940
H	-6.592316	-2.800454	-0.696515

1 imaginary frequency: –297.45 cm^{-1}

TS-p

Charge: 1

C	1.678658	2.167778	-0.878696
C	1.460934	3.535673	-1.061209
C	0.212652	4.107971	-0.801815
C	-0.830398	3.317741	-0.337453
C	-0.611780	1.946318	-0.107902
C	0.645416	1.368167	-0.408245
N	0.745471	-0.041377	-0.317029
S	1.520341	-0.756225	0.969275
O	1.419449	0.187070	2.072924
O	0.976448	-2.100556	1.079509
C	3.228201	-0.886726	0.503705
C	3.643360	-1.960507	-0.286954
C	4.969359	-2.020590	-0.699420
C	5.891781	-1.029409	-0.329318
C	5.448231	0.027988	0.474976
C	4.122656	0.107420	0.895547
H	3.786338	0.932439	1.525714
H	6.153184	0.804166	0.782298
C	7.324920	-1.123438	-0.773017
H	7.391738	-1.324599	-1.852759
H	7.834453	-1.952632	-0.256565

H	7.876988	-0.198933	-0.555403
H	5.299718	-2.858545	-1.318482
H	2.939616	-2.745729	-0.568944
C	0.383245	-0.810078	-1.506172
H	0.250590	-1.856335	-1.201834
C	-0.888631	-0.268527	-2.153872
C	-1.578337	1.150325	0.559731
C	-2.062531	0.834814	1.736809
C	-3.144488	-0.117499	2.135096
C	-1.376840	1.564767	2.884272
H	-0.596843	2.262241	2.561736
H	-0.924960	0.807052	3.540867
H	-4.113491	0.191047	1.720204
H	-2.921174	-1.130962	1.778625
H	-0.738911	0.769685	-2.484866
H	-1.045964	-0.845666	-3.079306
H	1.198295	-0.772663	-2.249389
H	-1.809769	3.748392	-0.121447
H	0.057096	5.175854	-0.963992
H	2.275302	4.161449	-1.432389
H	2.644759	1.720123	-1.118262
C	-2.161503	-0.367896	-1.344747
C	-2.595990	-1.635724	-0.890011
C	-3.122003	0.675427	-1.361258
C	-3.911093	-1.871945	-0.545850
H	-1.890667	-2.469212	-0.867780
C	-4.446986	0.452757	-0.990773
H	-2.845305	1.648222	-1.773601
C	-4.858378	-0.822418	-0.599472
H	-4.206883	-2.872270	-0.229468
H	-5.179313	1.260917	-1.019815
H	-2.148948	2.108316	3.450173
H	-3.228315	-0.140695	3.228219
O	-6.143702	-0.965998	-0.271783
C	-6.632534	-2.231315	0.129344
H	-6.132213	-2.584942	1.045078

H	-7.700800	-2.096695	0.336187
H	-6.513348	-2.981363	-0.668965

1 imaginary frequency: –232.18 cm^{-1}

INT1'

Charge: 1

C	0.873986	2.381439	-1.254939
C	0.757846	3.761624	-1.120733
C	0.044298	4.355027	-0.065401
C	-0.578524	3.549501	0.859243
C	-0.526130	2.128935	0.719890
C	0.260601	1.536289	-0.328918
N	0.371573	0.153860	-0.411073
S	1.077271	-0.712051	0.886440
O	0.934737	0.110607	2.078963
O	0.519903	-2.052248	0.843173
C	2.787384	-0.769319	0.446111
C	3.572353	0.368579	0.646703
C	4.908586	0.331638	0.268380
C	5.471401	-0.821162	-0.301821
C	4.657093	-1.947429	-0.479426
C	3.314692	-1.931997	-0.111875
H	2.688568	-2.815396	-0.247060
H	5.081518	-2.857688	-0.909200
C	6.910646	-0.836368	-0.731865
H	7.529618	-0.202188	-0.081625
H	7.004459	-0.445722	-1.758491
H	7.320447	-1.855855	-0.727081
H	5.532906	1.213922	0.427821
H	3.150472	1.266129	1.103230
C	0.494985	-0.501060	-1.726299
H	0.517523	-1.581446	-1.535058
C	-0.658929	-0.167138	-2.663097

C	-1.332124	1.359686	1.513899
C	-2.234481	0.770473	2.232913
C	-3.670646	0.807738	1.758341
C	-1.933058	0.062866	3.527022
H	-2.466323	0.579498	4.339182
H	-0.860680	0.033628	3.744517
H	-4.044473	-0.224083	1.694316
H	-4.265609	1.347959	2.510116
H	-0.590936	0.880844	-2.991217
H	-0.493505	-0.768342	-3.572587
H	1.453018	-0.242832	-2.206586
H	-1.146382	3.970348	1.690404
H	-0.009390	5.440946	0.017845
H	1.264154	4.401325	-1.847225
H	1.472604	1.965139	-2.064654
C	-2.058179	-0.425454	-2.145747
C	-2.303874	-1.300683	-1.079467
C	-3.136656	0.208834	-2.769646
C	-3.612332	-1.534503	-0.643015
H	-1.472865	-1.785391	-0.569574
C	-4.442793	-0.045078	-2.345215
H	-2.954932	0.900597	-3.596174
C	-4.689281	-0.911822	-1.287977
H	-5.281248	0.447815	-2.843265
H	-2.324948	-0.963000	3.463531
H	-3.783176	1.296823	0.783091
H	-5.703359	-1.108679	-0.934688
O	-3.916587	-2.320602	0.411373
C	-2.869123	-2.948140	1.115194
H	-3.331506	-3.478344	1.957095
H	-2.335369	-3.678106	0.483531
H	-2.138826	-2.219041	1.502671

There are no imaginary frequencies

INT2-p'

Charge: 1

C	1.494789	2.042645	-1.376370
C	1.318957	3.290026	-1.970197
C	0.100729	3.956456	-1.837627
C	-0.939426	3.373928	-1.115433
C	-0.771418	2.132158	-0.494081
C	0.463535	1.471708	-0.628093
N	0.603000	0.183063	-0.028017
S	1.610140	-0.020008	1.284452
O	1.868246	1.303543	1.830318
O	1.019284	-1.062140	2.113244
C	3.141514	-0.657429	0.641142
C	4.169829	0.231383	0.323940
C	5.352844	-0.265566	-0.212292
C	5.528852	-1.639380	-0.434977
C	4.482990	-2.508817	-0.098344
C	3.292856	-2.029446	0.442973
H	2.492204	-2.718442	0.717449
H	4.603704	-3.583269	-0.257040
C	6.824602	-2.164067	-0.987612
H	7.241474	-1.483000	-1.743607
H	6.697276	-3.157218	-1.440395
H	7.572937	-2.257453	-0.183597
H	6.161088	0.427582	-0.458652
H	4.045715	1.300534	0.504636
C	0.218884	-1.025106	-0.757630
H	0.031867	-1.819095	-0.021629
C	-1.006386	-0.866756	-1.660970
C	-1.879876	1.452351	0.241985
C	-1.963366	1.453216	1.585964
C	-2.929443	0.606506	2.373305
C	-1.070909	2.310956	2.436484
H	-1.690329	2.888010	3.143220
H	-0.452403	3.006170	1.856917

H	-2.498458	0.384531	3.360634
H	-3.881114	1.133029	2.553000
H	-0.845825	-0.030394	-2.357802
H	-1.039746	-1.781581	-2.268336
H	1.045714	-1.356865	-1.411409
H	-1.896913	3.892113	-1.021154
H	-0.042439	4.933685	-2.303995
H	2.132252	3.738507	-2.544569
H	2.437782	1.504270	-1.489888
C	-2.355673	-0.701595	-1.013508
C	-3.165934	-1.788862	-0.855848
C	-2.846193	0.666005	-0.665089
C	-4.520730	-1.630318	-0.443553
H	-2.793254	-2.780539	-1.112593
C	-4.267224	0.742812	-0.251854
H	-2.834550	1.212291	-1.634172
C	-5.076477	-0.334803	-0.190064
H	-4.654812	1.735960	-0.012479
H	-0.397493	1.679688	3.035387
H	-3.146996	-0.353837	1.888421
H	-6.127921	-0.265832	0.090552
O	-5.337935	-2.622994	-0.303816
C	-4.929175	-3.983059	-0.504773
H	-5.815047	-4.588929	-0.290798
H	-4.614021	-4.134916	-1.546293
H	-4.119596	-4.243264	0.190227

There are no imaginary frequencies

TS-p'

Charge: 1

C	1.564688	2.053575	-1.241333
C	1.494838	3.405227	-1.591358
C	0.376917	4.177533	-1.266903

C	-0.686977	3.603318	-0.584291
C	-0.613430	2.252583	-0.193428
C	0.518274	1.470157	-0.540796
N	0.477171	0.079918	-0.252231
S	1.255552	-0.496894	1.107824
O	1.269861	0.607392	2.055794
O	0.618666	-1.761007	1.440474
C	2.926042	-0.827497	0.611062
C	3.892873	0.165167	0.763143
C	5.189514	-0.081172	0.318310
C	5.532962	-1.303236	-0.273875
C	4.539901	-2.286569	-0.403716
C	3.240750	-2.060385	0.035468
H	2.480093	-2.837671	-0.057148
H	4.792992	-3.250584	-0.852138
C	6.930376	-1.571099	-0.758453
H	7.597650	-0.721787	-0.558669
H	6.935840	-1.766438	-1.842229
H	7.349515	-2.462748	-0.267192
H	5.952077	0.691832	0.440041
H	3.635663	1.116795	1.231211
C	0.192596	-0.835397	-1.358696
H	0.009166	-1.827564	-0.924947
C	-1.021287	-0.382002	-2.167848
C	-1.609482	1.662829	0.617660
C	-2.019148	1.306231	1.804839
C	-3.035049	0.286743	2.207822
C	-1.330220	2.059779	2.933383
H	-2.114438	2.516767	3.556202
H	-0.640930	2.837724	2.586518
H	-2.834496	-0.026839	3.240497
H	-4.046060	0.717458	2.170839
H	-0.830506	0.607273	-2.608825
H	-1.115229	-1.079235	-3.014412
H	1.062843	-0.911593	-2.034256
H	-1.572653	4.186988	-0.327473

H	0.336072	5.228933	-1.555744
H	2.323112	3.858283	-2.140101
H	2.429734	1.450556	-1.523257
C	-2.333152	-0.363926	-1.420822
C	-2.918793	-1.570351	-1.024345
C	-3.029941	0.845445	-1.205440
C	-4.222190	-1.588817	-0.512760
H	-2.369042	-2.500043	-1.175403
C	-4.353268	0.810846	-0.705189
H	-2.671801	1.762183	-1.671990
C	-4.948445	-0.383431	-0.374195
H	-4.907511	1.745397	-0.595873
H	-0.770366	1.328697	3.533053
H	-2.995340	-0.590625	1.554334
H	-5.967390	-0.430116	0.013383
O	-4.860361	-2.695837	-0.135437
C	-4.206094	-3.946445	-0.221148
H	-4.913008	-4.691265	0.162802
H	-3.951024	-4.196544	-1.263633
H	-3.292853	-3.963990	0.395636

1 imaginary frequency: –281.76 cm^{-1}

6.4 3-D Figures of computational Structures

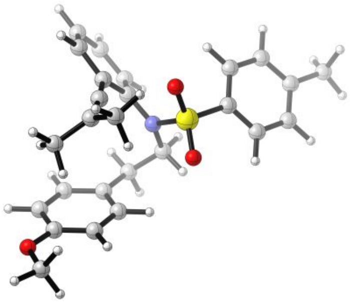

INT1

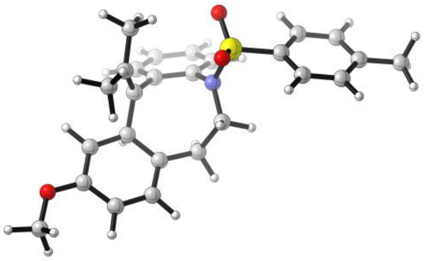

INT2-m

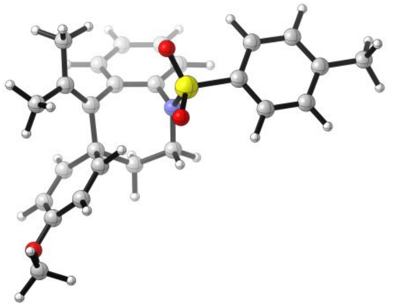

INT2-p

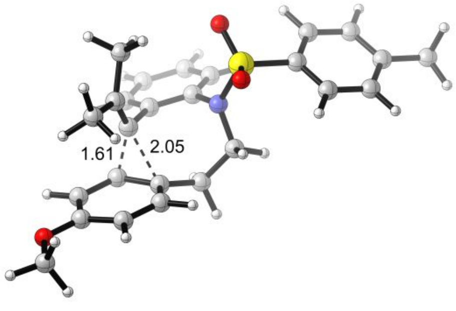

TS-R

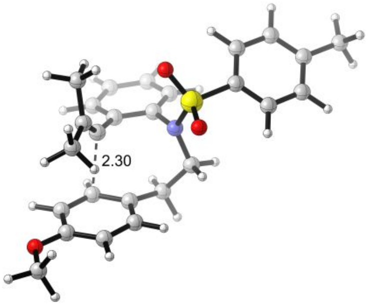

TS-m

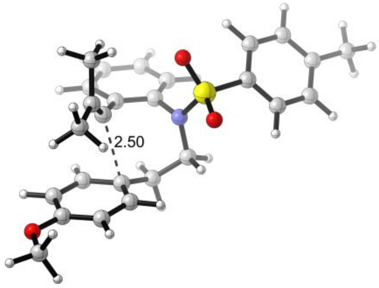

TS-p

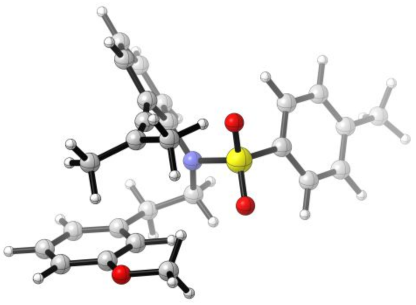

INT1’

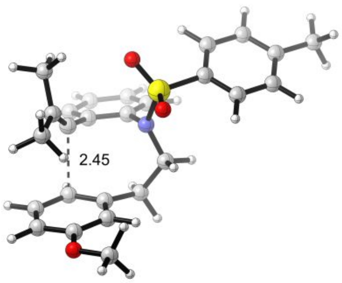

TS-p’

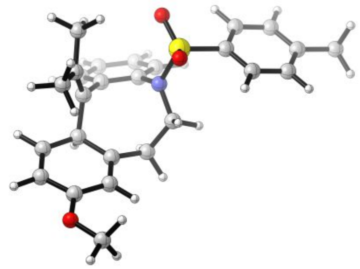

INT2-p’

7. MicroED data

7.1 MicroED Sample Preparation

All samples were crystallized using the following conditions. The samples were solubilized in minimal MeOH or $CHCl_3$ and subsequently transferred to 50 mm culture tubes. The culture tubes were then placed in a 4°C refrigerator to allow for slow solvent evaporation overnight. All crystalline samples were prepared according to previously disclosed procedures outlined in Jones *et al.*[22] Data was collected on a Thermo Fisher Talos F200C or Talos Arctica F200C transmission electron microscope operating with an accelerating voltage of 200keV, corresponding to an electron wavelength of 0.0251 Å. Electron diffraction data was collected using a Thermo Fisher CetaD camera. Screening the TEM grid for microcrystals was performed at 2600x magnification in imaging mode. Particles were visually selected for data collection and isolated by a selected area aperture. Data was collected by taking images of the diffraction patterns generated by a continuously rotating crystal integrated continuously at a rate of 3 seconds per frame. This rotation was performed at a rate of 0.3° per second with a minimum and maximum tilt range of −70° to +70°. Crystals selected for data collection were isolated by a selected area aperture to reduce the background noise contributions and calibrated to eucentric height to stay in the aperture over the entire tilt range. All diffraction data was processed using the XDS suite of programs as controlled by a custom Python automation script.[23-25] Structures were solved ab initio by direct methods in SHELXT or SHELXD and the direct preliminary solution is included for each entry. After this, structures were refined with SHELXL using ShelXle and incorporating electron scattering factors.[26-29] Thermal parameters were refined anisotropically for all non-hydrogen atoms. Hydrogen atoms were assigned using the riding model unless otherwise noted.

7.2 8-chloro-12-cyclohexylidene-5-tosyl-5,6,7,12-tetrahydrodibenzo[b,e]azocine (**SI-58, CCDC 2221222).**

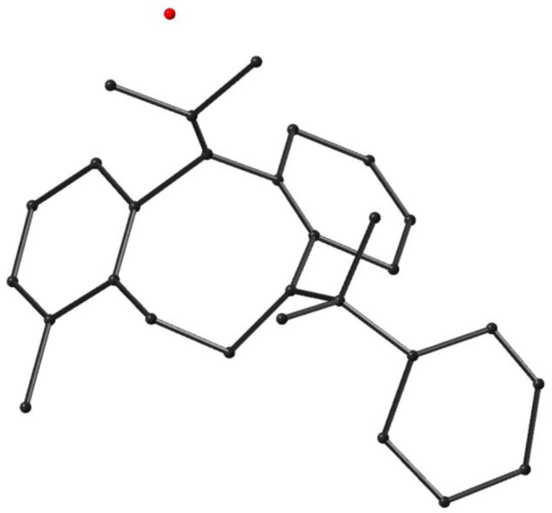

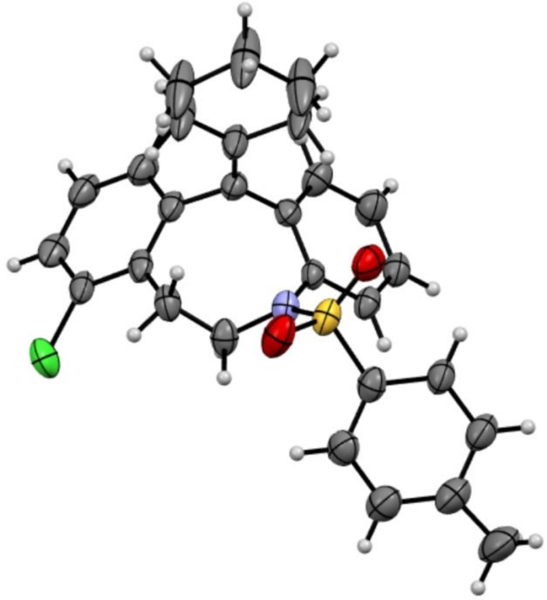

Initial direct methods solution of **SI-58** (left) and ORTEP diagram of refined **SI-58** (right). Thermal ellipsoids shown as shaded octants at 50% probability.

Crystal data and structure refinement for SI-58.

Empirical formula	$C_{28}H_{28}ClNO_2S$
Formula weight	478.05

Data Collection

Type of instrument	Talos Arctica F200C
Wavelength	0.0215 Å
Data collection temperature	100(4) K

Unit cell dimensions	a = 28.02(4)
	b = 8.53(2)
	c = 19.40(2)
Volume	4410(13)
Z	8
Crystal system	Monoclinic
Space group	C2/c
Density (calculated)	1.440 Mg/m^3
F(000)	810
Measured reflections	1903
Reflections with I > 2σ(*I*)	1196
Resolution	1.05 Å
Completeness	84.0%
Index ranges	26 ≤ h ≤ -26, 8 ≤ k ≤ -8, 18 ≤ l ≤ -18

Structure Solution and Refinement

Structure solution program	SHELXD (Uson & Sheldrick, 1999)
Primary solution method	Direct methods
Secondary solution method	Difference Fourier map
Hydrogen placement	Geometric positions
Structure refinement program	SHELXL-2018/3 (Sheldrick, 2018)
Refinement method	Full matrix least-squares on F^2

Data / restraints / parameters	1903 / 586 / 264
Treatment of hydrogen atoms	Riding
Goodness-of-fit on F^2	2.060
Final R indices [I>2s(I)]	R1 = 0.1882, *w*R2 = 0.4440
R indices (all data)	R1 = 0.2230, *w*R2 = 0.4605
Type of weighting scheme used	Sigma
Weighting scheme used	$w=1/s^2(Fo^2)$
Max shift/error	0.001
Average shift/error	0.000
Largest diff. peak and hole	0.14 and -0.15 e.Å^{-3}

Special Refinement Details

Refinement of F^2 against ALL reflections. The weighted R-factor (wR) and goodness of fit (S) are based on F-^{2}, conventional R-factors (R) are based on F, with F set to zero for negative F^2. The threshold expression of $F^2 > 2s(F^2)$ is used only for calculating R-factors(gt) etc. and is not relevant to the choice of reflections for refinement. R-factors based on F^2 are statistically about twice as large as those based on F, and R-factors based on ALL data will be even larger.

All esds (except the esd in the dihedral angle between two l.s. planes) are estimated using the full covariance matrix. The cell esds are taken into account individually in the estimation of esds in distances, angles and torsion angles; correlations between esds in cell parameters are only used when they are defined by crystal symmetry. An approximate (isotropic) treatment of cell esds is used for estimating esds involving l.s. planes.

7.3 12-(propan-2-ylidene)-5,6,7,12-tetrahydrodibenzo[a,d][8]annulene (**SI-62, CCDC 2252688).**

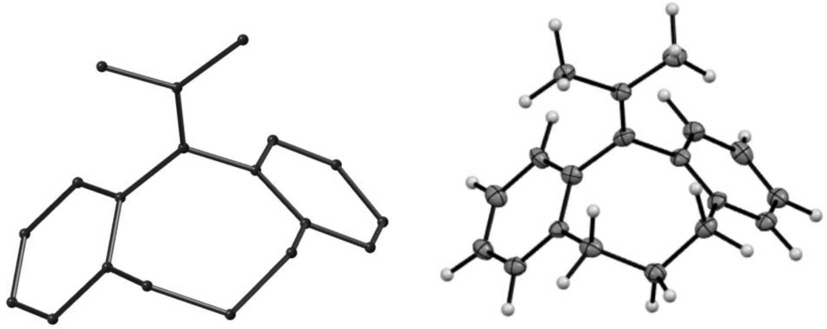

Initial direct methods solution of **SI-62** (left) and ORTEP diagram of refined **SI-62** (right). Thermal ellipsoids shown as shaded octants at 50% probability.

Crystal data and structure refinement for SI-62.

Empirical formula	$C_{19}H_{20}$
Formula weight	248.35

Data Collection

Type of instrument	Talos Arctica F200C
Wavelength	0.0215 Å
Data collection temperature	80(4) K
Unit cell dimensions	a = 8.2200(10)
	b = 13.690(2)
	c = 25.450(4)
Volume	2863.9(7)

Z	8
Crystal system	Orthorhombic
Space group	Pbca
Density (calculated)	1.152 Mg/m^3
F(000)	8
Measured reflections	2343
Reflections with $I > 2\sigma(I)$	1963
Resolution	0.85 Å
Completeness	92.6%
Index ranges	9 ≤ h ≤ -9, 16 ≤ k ≤ -16, 29 ≤ l ≤ -29

Structure Solution and Refinement

Structure solution program	SHELXT (Uson & Sheldrick, 1999)
Primary solution method	Direct methods
Secondary solution method	Difference Fourier map
Hydrogen placement	Refxyz
Structure refinement program	SHELXL-2018/3 (Sheldrick, 2018)
Refinement method	Full matrix least-squares on F^2
Data / restraints / parameters	2343 / 21 / 237
Treatment of hydrogen atoms	Riding
Goodness-of-fit on F^2	1.214
Final R indices [I>2s(I)]	R1 = 0.1503, *w*R2 = 0.3663

R indices (all data)	R1 = 0.1642, *w*R2 = 0.3763
Type of weighting scheme used	Sigma
Weighting scheme used	w=1/s^2(Fo^2)
Max shift/error	0.012
Average shift/error	0.000
Largest diff. peak and hole	0.15 and -0.13 e.Å^{-3}

Special Refinement Details

Refinement of F^2 against ALL reflections. The weighted R-factor (wR) and goodness of fit (S) are based on F-^{2}, conventional R-factors (R) are based on F, with F set to zero for negative F^2. The threshold expression of $F^2 > 2s(F^2)$ is used only for calculating R-factors(gt) etc. and is not relevant to the choice of reflections for refinement. R-factors based on F^2 are statistically about twice as large as those based on F, and R-factors based on ALL data will be even larger.

All esds (except the esd in the dihedral angle between two l.s. planes) are estimated using the full covariance matrix. The cell esds are taken into account individually in the estimation of esds in distances, angles and torsion angles; correlations between esds in cell parameters are only used when they are defined by crystal symmetry. An approximate (isotropic) treatment of cell esds is used for estimating esds involving l.s. planes.

7.4 12-cyclohexylidene-10-methoxy-5-tosyl-5,6,7,12-tetrahydrodibenzo[b,e]azocine (**SI-64, CCDC 2252686).**

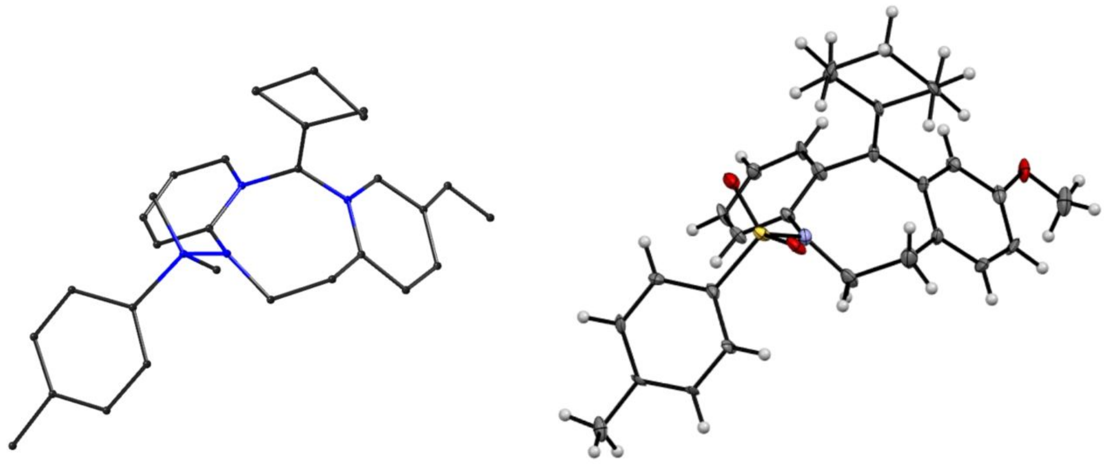

Initial direct methods solution of **SI-64** (left) and ORTEP diagram of refined **SI-64** (right). Thermal ellipsoids shown as shaded octants at 50% probability.

Crystal data and structure refinement for SI-64.

Empirical formula	$C_{29}H_{31}NO_3S$
Formula weight	473.61

Data Collection

Type of instrument	Talos Arctica F200C
Wavelength	0.0215 Å
Data collection temperature	80(4) K
Unit cell dimensions	a = 9.9400(10)
	b = 8.940(2)
	c = 28.230(4)
	β = 96.45
Volume	2492.7(7)
Z	4
Crystal system	Monoclinic
Space group	$P2_1/n$
Density (calculated)	1.262 Mg/m^3
F(000)	24
Measured reflections	3339
Reflections with $I > 2\sigma(I)$	2066
Resolution	0.90 Å

Completeness	91.1%
Index ranges	$11 \leq h \leq -11$, $9 \leq k \leq -9$, $31 \leq l \leq -31$

Structure Solution and Refinement

Structure solution program	SHELXT (Uson & Sheldrick, 1999)
Primary solution method	Direct methods
Secondary solution method	Difference Fourier map
Hydrogen placement	Geometric positions
Structure refinement program	SHELXL-2018/3 (Sheldrick, 2018)
Refinement method	Full matrix least-squares on F^2
Data / restraints / parameters	3339 / 228 / 308
Treatment of hydrogen atoms	Riding
Goodness-of-fit on F^2	1.464
Final R indices [I>2s(I)]	R1 = 0.1654, *w*R2 = 0.4156
R indices (all data)	R1 = 0.2153, *w*R2 = 0.4486
Type of weighting scheme used	Sigma
Weighting scheme used	$w=1/s^2(Fo^2)$
Max shift/error	0.005
Average shift/error	0.000
Largest diff. peak and hole	0.20 and -0.26 e.Å^{-3}

Special Refinement Details

Refinement of F^2 against ALL reflections. The weighted R-factor (wR) and goodness of fit (S) are based on F-^{2}, conventional R-factors (R) are based on F, with F set to zero for negative

F^2. The threshold expression of $F^2 > 2s(F^2)$ is used only for calculating R-factors(gt) etc. and is not relevant to the choice of reflections for refinement. R-factors based on F^2 are statistically about twice as large as those based on F, and R-factors based on ALL data will be even larger.

All esds (except the esd in the dihedral angle between two l.s. planes) are estimated using the full covariance matrix. The cell esds are taken into account individually in the estimation of esds in distances, angles and torsion angles; correlations between esds in cell parameters are only used when they are defined by crystal symmetry. An approximate (isotropic) treatment of cell esds is used for estimating esds involving l.s. planes.

7.5 10-bromo-12-(propan-2-ylidene)-5-tosyl-5,6,7,12-tetrahydrodibenzo[b,e]azocine (SI-66, CCDC 2252689).

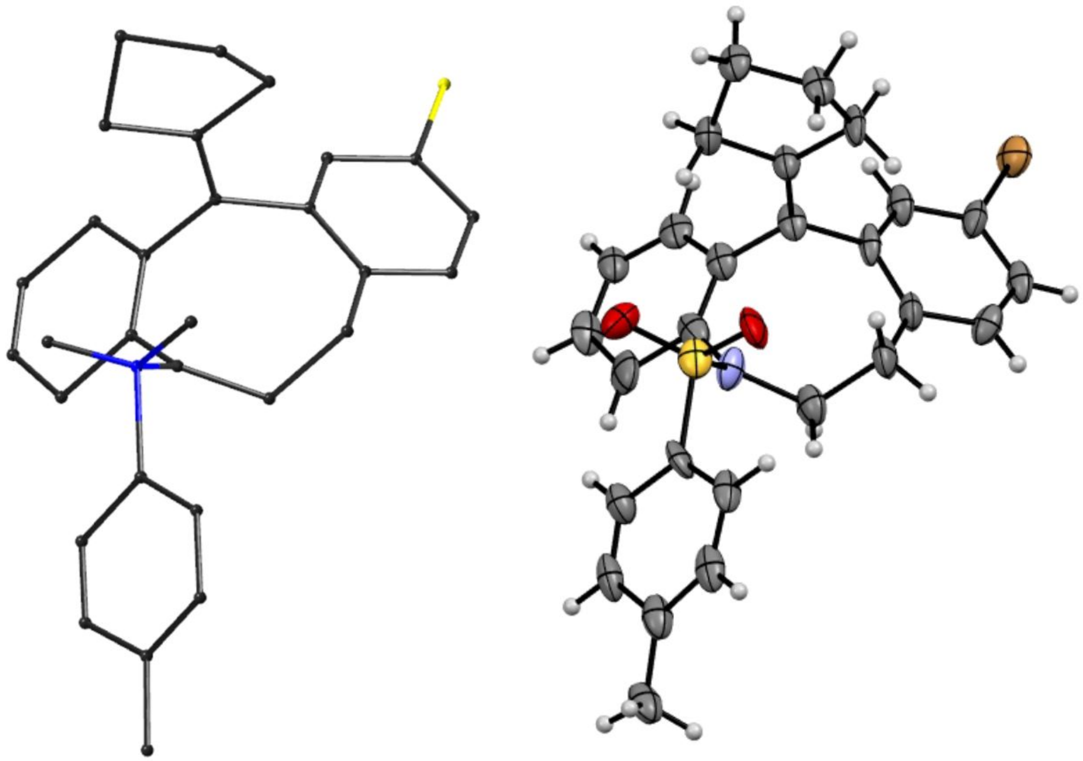

Initial direct methods solution of **SI-66** (left) and ORTEP diagram of refined **SI-66** (right). Thermal ellipsoids shown as shaded octants at 50% probability.

Crystal data and structure refinement for SI-66.

Empirical formula	$C_{27}H_{26}BrNO_2S$
Formula weight	508.45

Data Collection

Type of instrument	Talos Arctica F200C
Wavelength	0.0215 Å
Data collection temperature	80(4) K
Unit cell dimensions	a = 9.6000(10)
	b = 9.640(2)
	c = 25.890(4)
	β = 95.46
Volume	2385.1(7)
Z	4
Crystal system	Monoclinic
Space group	$P2_1/n$
Density (calculated)	1.416 Mg/m^3
F(000)	24
Measured reflections	2299
Reflections with $I > 2\sigma(I)$	1573
Resolution	1.0 Å
Completeness	89.1%
Index ranges	5 ≤ h ≤ -5, 9 ≤ k ≤ -9, 18 ≤ l ≤ -18

Structure Solution and Refinement

Structure solution program	SHELXT (Uson & Sheldrick, 1999)

Primary solution method	Direct methods
Secondary solution method	Difference Fourier map
Hydrogen placement	Geometric positions
Structure refinement program	SHELXL-2018/3 (Sheldrick, 2018)
Refinement method	Full matrix least-squares on F^2
Data / restraints / parameters	2299 / 216 / 290
Treatment of hydrogen atoms	Riding
Goodness-of-fit on F^2	1.531
Final R indices [I>2s(I)]	R1 = 0.1809, *w*R2 = 0.4249
R indices (all data)	R1 = 0.2194, *w*R2 = 0.4511
Type of weighting scheme used	Sigma
Weighting scheme used	$w=1/s^2(Fo^2)$
Max shift/error	0.005
Average shift/error	0.000
Largest diff. peak and hole	0.29 and -0.21 e.Å^{-3}

Special Refinement Details

Refinement of F^2 against ALL reflections. The weighted R-factor (wR) and goodness of fit (S) are based on F-^{2}, conventional R-factors (R) are based on F, with F set to zero for negative F^2. The threshold expression of $F^2 > 2s(F^2)$ is used only for calculating R-factors(gt) etc. and is not relevant to the choice of reflections for refinement. R-factors based on F^2 are statistically about twice as large as those based on F, and R-factors based on ALL data will be even larger.

All esds (except the esd in the dihedral angle between two l.s. planes) are estimated using the full covariance matrix. The cell esds are taken into account individually in the estimation of esds in distances, angles and torsion angles; correlations between esds in cell parameters are only used when they are defined by crystal symmetry. An approximate (isotropic) treatment of cell esds is used for estimating esds involving l.s. planes.

7.6 12-(propan-2-ylidene)-5-tosyl-5,6,7,12-tetrahydrodibenzo[b,e]azocine (SI-61, CCDC 2252690).

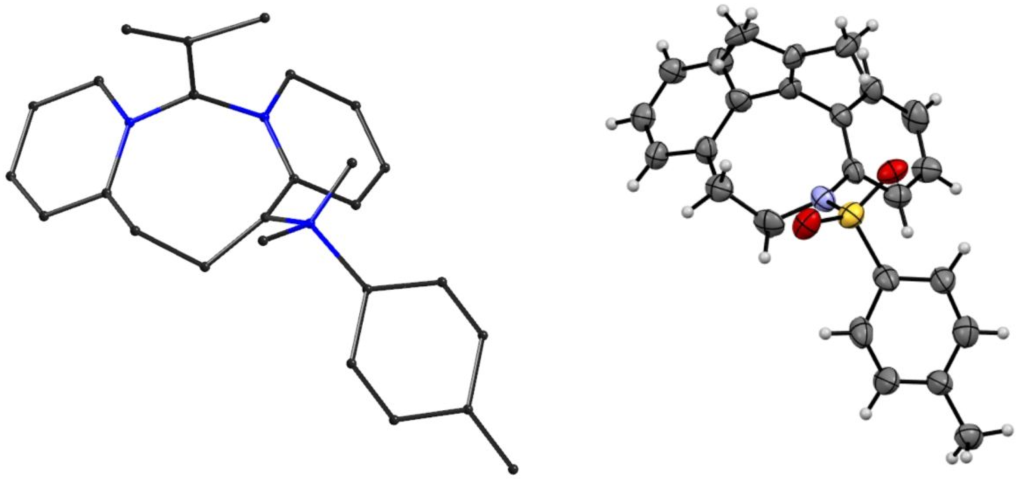

Initial direct methods solution of **SI-61** (left) and ORTEP diagram of refined **SI-61** (right). Thermal ellipsoids shown as shaded octants at 50% probability.

Crystal data and structure refinement for SI-61.

Empirical formula	$C_{25}H_{25}NO_2S$
Formula weight	403.52

Data Collection

Type of instrument	Talos Arctica F200C
Wavelength	0.0215 Å
Data collection temperature	80(4) K
Unit cell dimensions	a = 12.9200(2)
	b = 8.920(10)
	c = 38.240(4)

Volume	4407.0(10)
Z	8
Crystal system	Orthorhombic
Space group	Pbca
Density (calculated)	1.216 Mg/m^3
F(000)	17
Measured reflections	1858
Reflections with I > 2σ(*I*)	1093
Resolution	1.0 Å
Completeness	83.4%
Index ranges	12 ≤ h ≤ -12, 8 ≤ k ≤ -8, 34 ≤ l ≤ -34

Structure Solution and Refinement

Structure solution program	SHELXT (Uson & Sheldrick, 1999)
Primary solution method	Direct methods
Secondary solution method	Difference Fourier map
Hydrogen placement	Geometric positions
Structure refinement program	SHELXL-2018/3 (Sheldrick, 2018)
Refinement method	Full matrix least-squares on F^2
Data / restraints / parameters	1858 / 194 / 263
Treatment of hydrogen atoms	Riding
Goodness-of-fit on F^2	1.471

Final R indices [I>2s(I)]	R1 = 0.1667, *w*R2 = 0.4314
R indices (all data)	R1 = 0.2165, *w*R2 = 0.4314
Type of weighting scheme used	Sigma
Weighting scheme used	$w=1/s^2(Fo^2)$
Max shift/error	0.000
Average shift/error	0.000
Largest diff. peak and hole	0.12 and -0.11 e.Å^{-3}

Special Refinement Details

Refinement of F^2 against ALL reflections. The weighted R-factor (wR) and goodness of fit (S) are based on $F\text{-}^2$, conventional R-factors (R) are based on F, with F set to zero for negative F^2. The threshold expression of $F^2 > 2s(F^2)$ is used only for calculating R-factors(gt) etc. and is not relevant to the choice of reflections for refinement. R-factors based on F^2 are statistically about twice as large as those based on F, and R-factors based on ALL data will be even larger.

All esds (except the esd in the dihedral angle between two l.s. planes) are estimated using the full covariance matrix. The cell esds are taken into account individually in the estimation of esds in distances, angles and torsion angles; correlations between esds in cell parameters are only used when they are defined by crystal symmetry. An approximate (isotropic) treatment of cell esds is used for estimating esds involving l.s. planes.

7.7 12-cyclohexylidene-N,N-dimethyl-5-tosyl-5,6,7,12-tetrahydrodibenzo[b,e]azocin-10-amine (SI-63, CCDC 2252687).

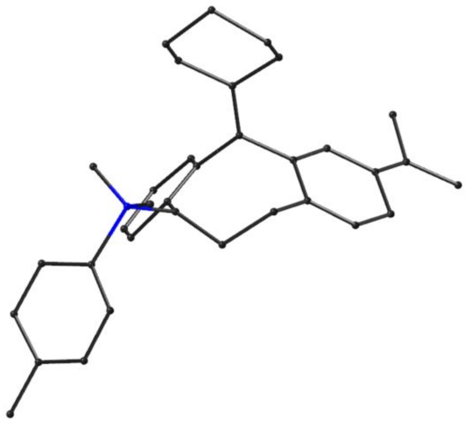

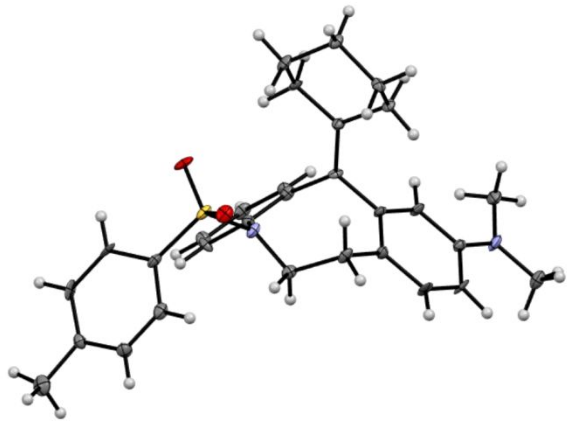

Initial direct methods solution of **SI-63** (left) and ORTEP diagram of refined **SI-63** (right). Thermal ellipsoids shown as shaded octants at 50% probability.

Crystal data and structure refinement for SI-63.

Empirical formula	$C_{30}H_{34}N_2O_2S$
Formula weight	486.65

Data Collection

Type of instrument	Talos Arctica F200C
Wavelength	0.0215 Å
Data collection temperature	80(4) K
Unit cell dimensions	a = 9.9900(10)
	b = 8.920(2)
	c = 28.880(4)
	β = 97.95
Volume	2548.8(7)
Z	4
Crystal system	Monoclinic
Space group	$P2_1/n$
Density (calculated)	1.268 Mg/m^3
F(000)	17
Measured reflections	4147
Reflections with I > 2σ(*I*)	2603
Resolution	0.85 Å

Completeness	94.7%
Index ranges	$11 \le h \le -11$, $10 \le k \le -10$, $33 \le l \le -33$

Structure Solution and Refinement

Structure solution program	SHELXT (Uson & Sheldrick, 1999)
Primary solution method	Direct methods
Secondary solution method	Difference Fourier map
Hydrogen placement	Geometric positions
Structure refinement program	SHELXL-2018/3 (Sheldrick, 2018)
Refinement method	Full matrix least-squares on F^2
Data / restraints / parameters	4147 / 234 / 317
Treatment of hydrogen atoms	Riding
Goodness-of-fit on F^2	1.497
Final R indices [I>2s(I)]	R1 = 0.1834, *w*R2 = 0.4392
R indices (all data)	R1 = 0.2291, *w*R2 = 0.4670
Type of weighting scheme used	Sigma
Weighting scheme used	$w=1/s^2(Fo^2)$
Max shift/error	0.004
Average shift/error	0.000
Largest diff. peak and hole	0.38 and -0.19 e.Å^{-3}

Special Refinement Details

Refinement of F^2 against ALL reflections. The weighted R-factor (wR) and goodness of fit (S) are based on $F\text{-}^2$, conventional R-factors (R) are based on F, with F set to zero for negative

F^2. The threshold expression of $F^2 > 2s(F^2)$ is used only for calculating R-factors(gt) etc. and is not relevant to the choice of reflections for refinement. R-factors based on F^2 are statistically about twice as large as those based on F, and R-factors based on ALL data will be even larger.

All esds (except the esd in the dihedral angle between two l.s. planes) are estimated using the full covariance matrix. The cell esds are taken into account individually in the estimation of esds in distances, angles and torsion angles; correlations between esds in cell parameters are only used when they are defined by crystal symmetry. An approximate (isotropic) treatment of cell esds is used for estimating esds involving l.s. planes.

7.8 11-cyclohexylidene-6-tosyl-4,5,6,11-tetrahydrobenzo[b]thieno[3,2-e]azocine (SI-67, CCDC 2252685).

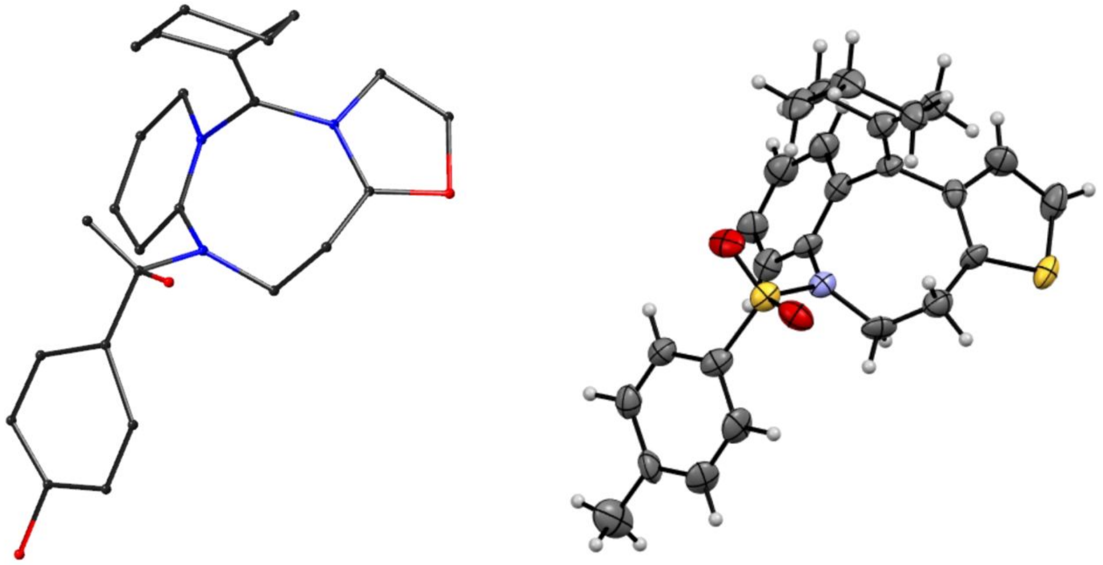

Initial direct methods solution of **SI-67** (left) and ORTEP diagram of refined **SI-67** (right). Thermal ellipsoids shown as shaded octants at 50% probability.

Crystal data and structure refinement for SI-67.

Empirical formula	$C_{26}H_{27}NO_2S_2$
Formula weight	449.61

Data Collection

Type of instrument	Talos Arctica F200C
Wavelength	0.0215 Å

Data collection temperature	80(4) K
Unit cell dimensions	a = 13.890(2)
	b = 9.2000(10)
	c = 37.650(4)
Volume	4811.2(10)
Z	8
Crystal system	Orthorhombic
Space group	Pbca
Density (calculated)	1.241 Mg/m^3
F(000)	17
Measured reflections	1679
Reflections with I > 2σ(*I*)	939
Resolution	1.1 Å
Completeness	85.1%
Index ranges	12 ≤ h ≤ -12, 8 ≤ k ≤ -8, 31 ≤ 1 ≤ -31

Structure Solution and Refinement

Structure solution program	SHELXT (Uson & Sheldrick, 1999)
Primary solution method	Direct methods
Secondary solution method	Difference Fourier map
Hydrogen placement	Geometric positions
Structure refinement program	SHELXL-2018/3 (Sheldrick, 2018)

Refinement method	Full matrix least-squares on F^2
Data / restraints / parameters	1679 / 210 / 281
Treatment of hydrogen atoms	Riding
Goodness-of-fit on F^2	1.447
Final R indices [I>2s(I)]	R1 = 0.1278, *w*R2 = 0.3810
R indices (all data)	R1 = 0.1867, *w*R2 = 0.4140
Type of weighting scheme used	Sigma
Weighting scheme used	$w=1/s^2(Fo^2)$
Max shift/error	0.000
Average shift/error	0.000
Largest diff. peak and hole	0.13 and -0.10 e.$Å^{-3}$

Special Refinement Details

Refinement of F^2 against ALL reflections. The weighted R-factor (wR) and goodness of fit (S) are based on F-^{2}, conventional R-factors (R) are based on F, with F set to zero for negative F^2. The threshold expression of $F^2 > 2s(F^2)$ is used only for calculating R-factors(gt) etc. and is not relevant to the choice of reflections for refinement. R-factors based on F^2 are statistically about twice as large as those based on F, and R-factors based on ALL data will be even larger.

All esds (except the esd in the dihedral angle between two l.s. planes) are estimated using the full covariance matrix. The cell esds are taken into account individually in the estimation of esds in distances, angles and torsion angles; correlations between esds in cell parameters are only used when they are defined by crystal symmetry. An approximate (isotropic) treatment of cell esds is used for estimating esds involving l.s. planes.

8. References

1. Kuprat, M.; Lehmann, M.; Shulz, A.; Villinger, A. Synthesis of Pentafluorophenyl Silver by Means of Lewis Acid Catalysis: Structure of Silver Solvent Complexes. *Organometallics*, **2010**, *29*, 1421–1427.

2. Colas, K.; Dos Santos A.; Mendoza, A. i-Pr2NMgCl·LiCl Enables the Synthesis of Ketones by Direct Addition of Grignard Reagents to Carboxylate Anions. *Org. Lett.* **2019**, *21*, 7908–7913.

3. Bruneau-Voisine, A.; Pallova, L.; Bastin, S.; César, V.; Sortais, J. Manganese catalyzed α-methylation of ketones with methanol as a C1 source. *Chem. Commun.* **2019**, *55*, 314–317.

4. Kruegel, A. C. *U. S. Patent Application 17/510*, 108.

5. Zheng, Q.; Liang, Y.; Qin, C.; Jiao, N. Ru(II)-catalyzed intermolecular C–H amidation of weakly coordinating ketones. *Chem. Commun.* **2013**, *49*, 5654–5656.

6. Yang, F.; Ding, D.; Wang, C. Nickel-Catalyzed Directed Cross-Electrophile Coupling of Phenolic Esters with Alkyl Bromides. *Org. Lett.* **2020**, *22*, 9203–9209.

7. Mihai, M. T.; Williams, B. D.; Phipps, R. J. Para-Selective C–H Borylation of Common Arene Building Blocks Enabled by Ion-Pairing with a Bulky Countercation. *J. Am. Chem. Soc.* **2019**, *141*, 15477–15482.

8. Zhang, B.; Wang, H.; Kang, Y.; Zhang, P.; Xu, H.; Lu, Y.; Sun, W. Rhodium-Catalyzed Direct Ortho C–H Arylation Using Ketone as Directing Group with Boron Reagent. *Org. Lett.* **2017**, *19*, 5940–5943.

9. Li, Z.; Gupta, M. K.; Snowden, T. S. One-Carbon Homologation of Primary Alcohols and the Reductive Homologation of Aldehydes Involving a Jocic-Type Reaction. *Eur. J. Org. Chem.* **2015**, *32*, 7009–7019.

10. Avery, M. A.; Muraleedharan, K. M.; Desai, P. V.; Bandyopadhyaya, A. K.; Furtado, M. M.; Tekwani, B. L. Structure−Activity Relationships of the Antimalarial Agent Artemisinin. 8. Design, Synthesis, and CoMFA Studies toward the Development of Artemisinin-Based Drugs against Leishmaniasis and Malaria. *J. Med. Chem.* **2003**, *46*, 4244–4258.

11. Liu, Q.; Hong, J.; Sun, B.; Bai, G.; Li, F.; Liu, G.; Yang, Y.; Mo, F. Transition-Metal-Free Borylation of Alkyl Iodides via a Radical Mechanism. *Org. Lett.* **2019**, *21*, 6597–6602.

12. Shrestha, T. B.; Troyer, D. L.; Bossmann, S. H. Strategies for Large-Scale Synthesis of Coelenterazine for in Vivo Applications. *Synthesis*, **2014**, *46*, 646–652.

13. Moriya, T.; Yoneda, S.; Kawana, K.; Ikeda, R.; Konakahara, T.; Sakai, N. Indium-Catalyzed Reductive Bromination of Carboxylic Acids Leading to Alkyl Bromides. *Org. Lett.* **2012**, *14*, 4842–4845.

14. Zhang, B.; Wang, H.; Kang, Y.; Zhang, P.; Xu, H.; Lu, Y.; Sun, W. Rhodium-Catalyzed Direct Ortho C–H Arylation Using Ketone as Directing Group with Boron Reagent. *Org. Lett.* **2017**, *19*, 5940–5943.

15. Dalling, A. G.; Yamauchi, T.; McCreanor, N. G.; Cox, L.; Bower, J. F. Carbonylative C−C Bond Activation of Electron-Poor Cyclopropanes: Rhodium-Catalyzed (3+1+2) Cycloadditions of Cyclopropylamides. *Angew. Chem. Int. Ed.* **2019**, *58*, 221–225.

16. Frisch, M. J.; Trucks, G. W.; Schlegel, H. B.; Scuseria, G. E.; Robb, M. A.; Cheeseman, J. R.; Scalmani, G.; Barone, V.; Petersson, G. A.; Nakatsuji, H.; Li, X.; Caricato, M.; Marenich, A. V.; Bloino, J.; Janesko, B. G.; Gomperts, R.; Mennucci, B.; Hratchian, H. P.; Ortiz, J. V.; Izmaylov,

A. F.; Sonnenberg, J. L.; Williams-Young, D.; Ding, F.;Lipparini, F.; Egidi, F.;Goings, J.; Peng, B.; Petrone, A.; Henderson, T.; Ranasinghe, D.; Zakrzewski, V. G.; Gao, J.; Rega, N.; Zheng, G.; Liang, W.; Hada, M.; Ehara, M.; Toyota, K.; Fukuda, R.; Hasegawa, J.; Ishida, M.; Nakajima, T.; Honda, Y.; Kitao, O.; Nakai, H.; Vreven, T.; Throssell, K.; Montgomery Jr., J. A.; Peralta, J. E.; Ogliaro, F.; Bearpark, M. J.; Heyd, J. J.; Brothers, E. N.; Kudin, K. N.; Staroverov, V. N.; Keith, T. A.; Kobfayashi, R.; Normand, J.; Raghavachari, K.; Rendell, A. P.; Burant, J. C.; Iyengar, S. S.; Tomasi, J.; Cossi, M.; Millam, J. M.; Klene, M.; Adamo, C.; Cammi, R.; Ochterski, J. W.; Martin, R. L.; Morokuma, K.; Farkas, O.; Foresman, J. B.; Fox, D. J. *Gaussian 16*; Gaussian, Inc.: Wallingford, CT, **2016**.

17. Chai, J.-D.; Head-Gordon, M. Long-range corrected hybrid density functionals with damped atom–atom dispersion corrections. *Phys. Chem. Chem. Phys.* **2008**, *10*, 6615−6620.

18. Weigend, F.; Ahlrichs, R. Balanced basis sets of split valence, triple zeta valence and quadruple zeta valence quality for H to Rn: Design and assessment of accuracy. *Phys. Chem. Chem. Phys.* **2005**, *7*, 3297−3305.

19. Marenich, A. V.; Cramer, C. J.; Truhlar, D. G. Universal Solvation Model Based on Solute Electron Density and on a Continuum Model of the Solvent Defined by the Bulk Dielectric Constant and Atomic Surface Tensions. *J. Phys. Chem. B* **2009**, *113*, 6378–6396.

20. (a) Grimme, S.; Bannwarth, C.; Shushkov, P. A Robust and Accurate Tight-Binding Quantum Chemical Method for Structures, Vibrational Frequencies, and Noncovalent Interactions of Large Molecular Systems Parametrized for All spd-Block Elements (Z = 1–86). *J. Chem. Theory Comput.* **2017**, *13*, 1989–2009. (b) Bannwarth, C.; Ehlert, S.; Grimme, S. GFN2-xTB—An Accurate and Broadly Parametrized Self-Consistent Tight-Binding Quantum Chemical Method with Multipole Electrostatics and Density-Dependent Dispersion Contributions. *J. Chem. Theory Comput.* **2019**, *15*, 1652–1671. (c) Pracht, P.; Caldeweyher, E.; Ehlert, S.; Grimme, S. A Robust Non-Self-Consistent Tight-Binding Quantum Chemistry Method for large Molecules. *ChemRxiv* **2019,** https://doi.org/10.26434/chemrxiv.8326202.v1

21. Legault, C. Y. *CYLview*, 1.0b; Universite′ de Sherbrooke, **2009**; http://www.cylview.org.

22. Jones, C. G.; Martynowycz, M. W.; Hattne, J.; Fulton, T. J.; Stoltz, B. M.; Rodriguez, J. A.; Nelson, H. M.; Gonen, T. The CryoEM method MicroED as a powerful tool for small molecule structure determination. *ACS Cent. Sci.* **2018**, *4*, 1587-1592.

23. Kabsch, W. XDS. *Acta Cryst.* **2010**, *D66*, 125-132.

24. Kabsch, W. Integration, scaling, space-group assignment and post-refinement. *Acta Cryst.* **2010**, *D66*, 133-144.

25. Hattne, J.; Reyes, F. E.; Nannenga, B. L.; Shi, D.; de la Cruz, M. J.; Leslie, A. G.; Gonen, T. MicroED data collection and processing. *Acta Cryst.* **2015**, *A71*, 353-360.

26. Sheldrick, G. M. A short history of SHELX. *Acta Cryst.* **2008**, *A64*, 112-122.

27. Sheldrick, G. M. SHELXT - integrated space-group and crystal-structure determination. *Acta Cryst.* **2015**, *A71*, 3-8.

28. Sheldrick, G. M. SHELXT - integrated space-group and crystal-structure determination. *Acta Cryst.* **2015**, *C71*, 3-8.

29. Hübschle, C. B.; Sheldrick, G. M.; Dittrich, B. ShelXle: a Qt graphical user interface for SHELXL. *J. Appl. Crystallogr.* **2011**, *44*, 1281-1284.

9. ^{1}H NMR, ^{13}C NMR spectral data

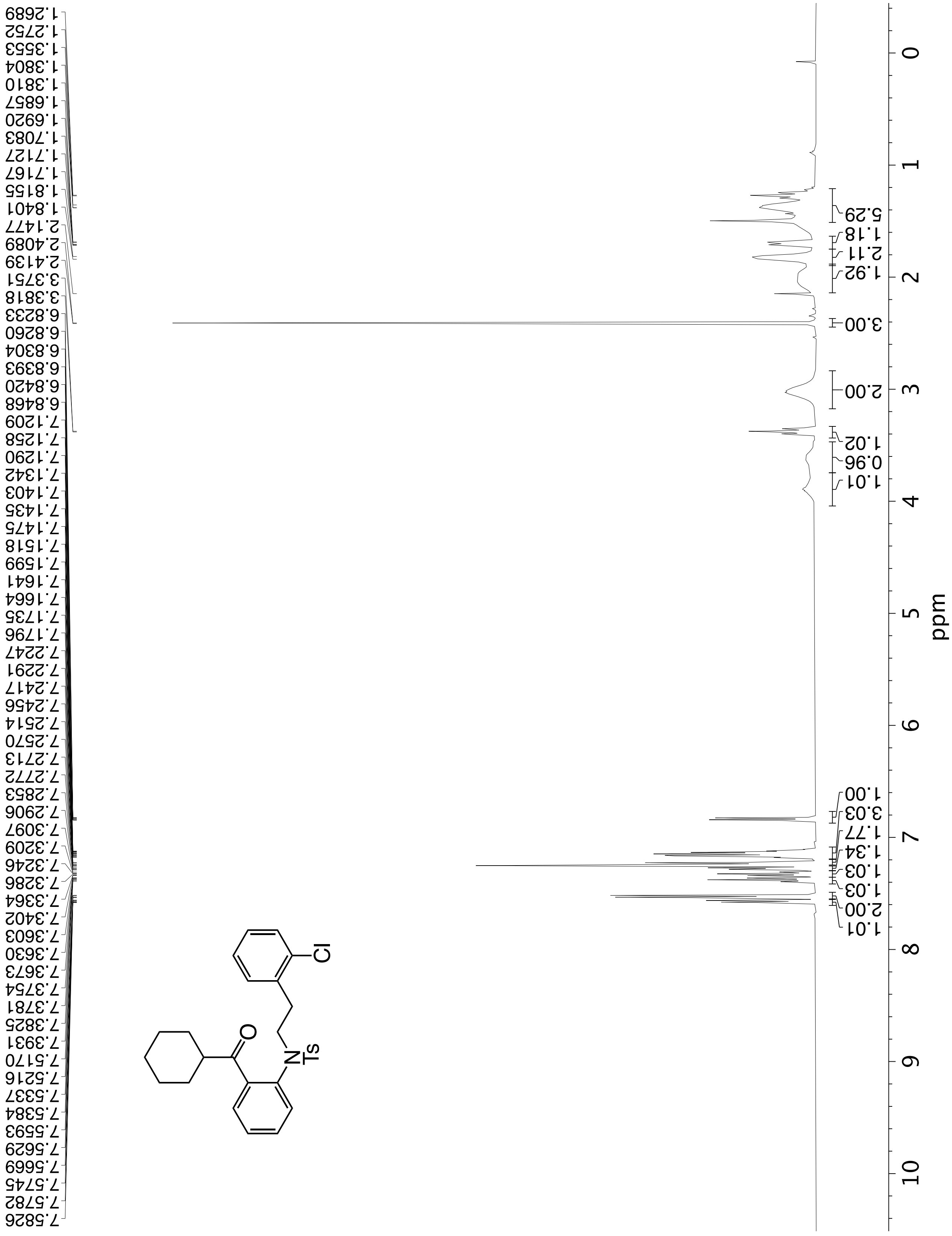

^{1}H NMR (400 MHz, $CDCl_3$) of compound **SI-8**.

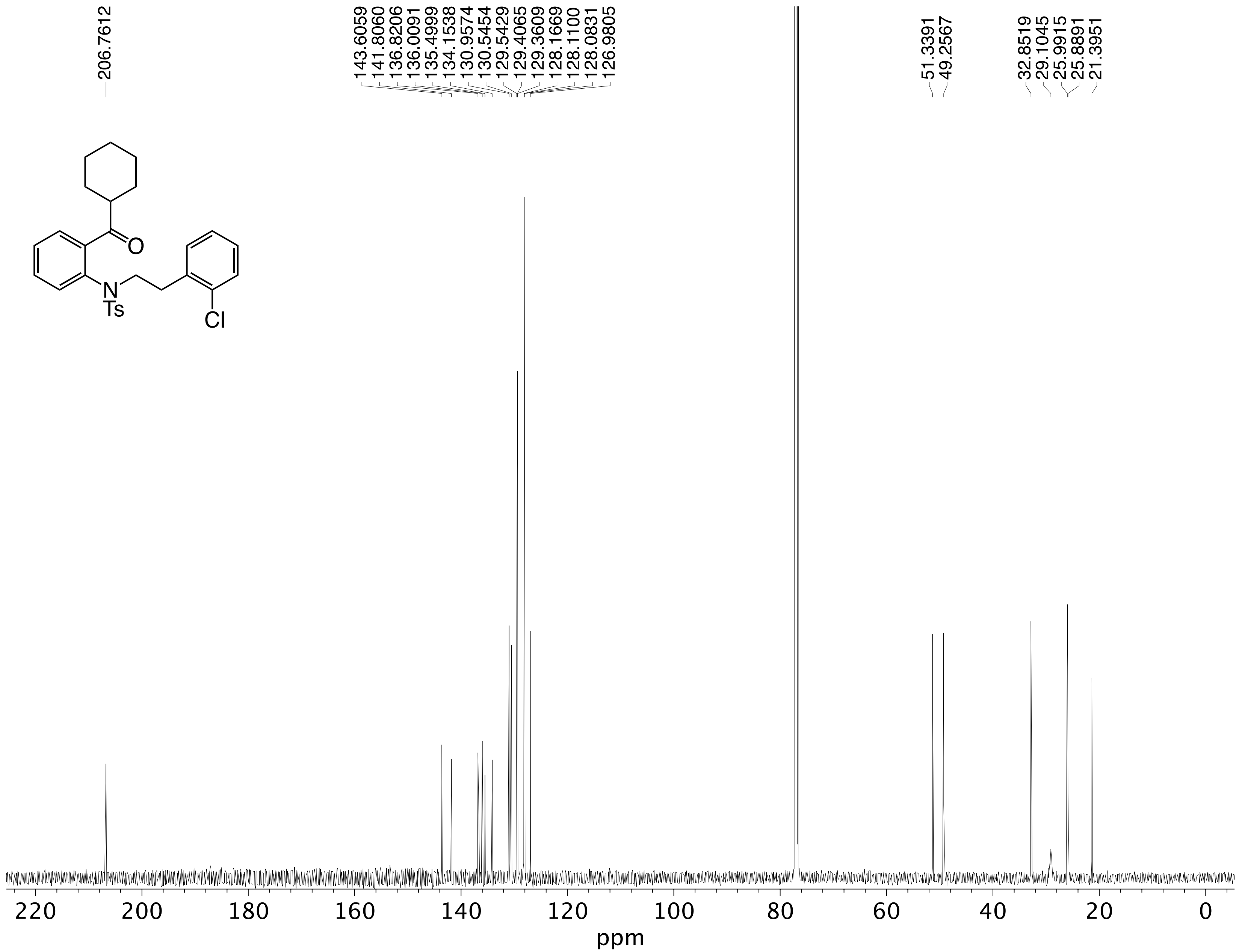

^{13}C NMR (101 MHz, $CDCl_3$) of compound **SI-8**.

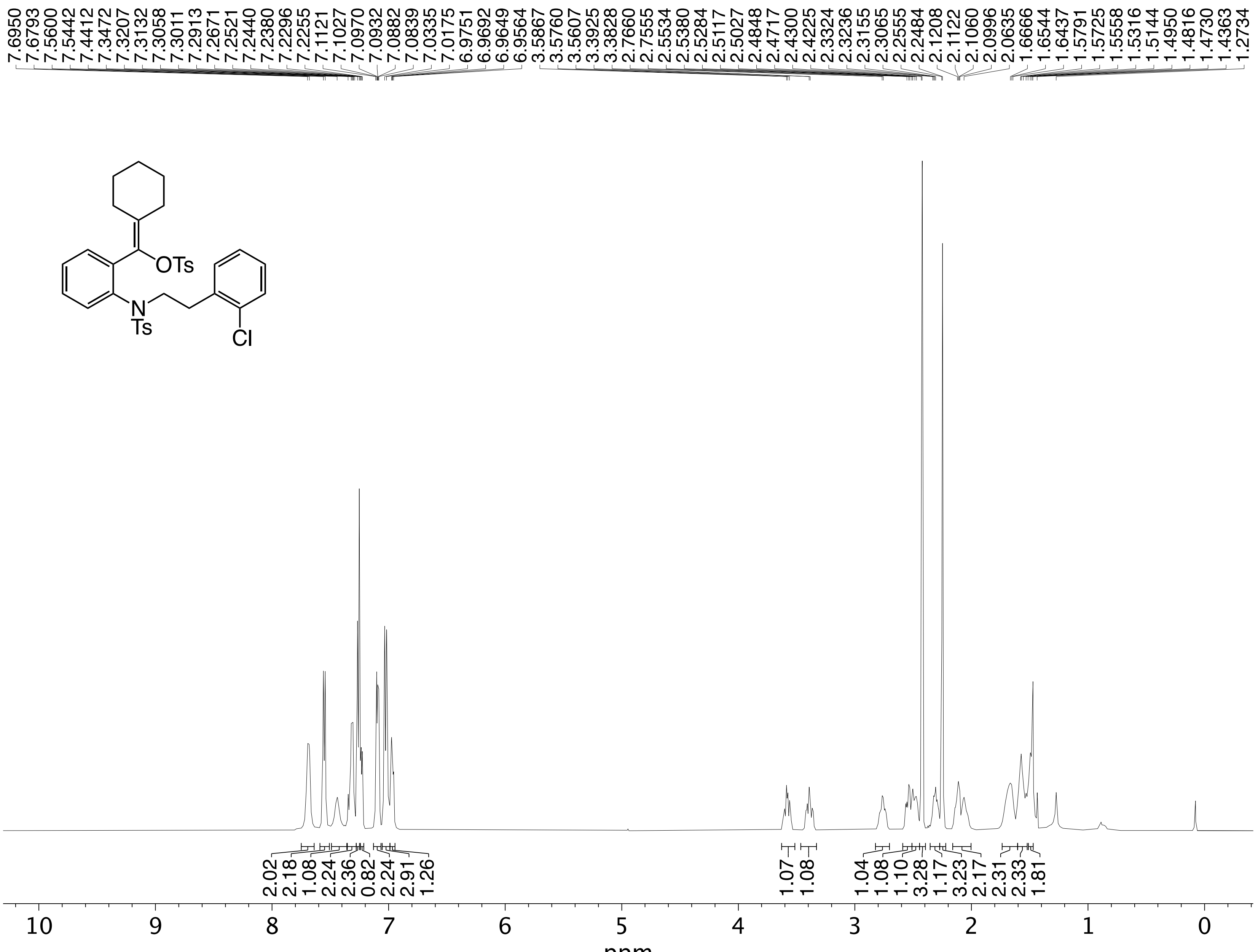

^{1}H NMR (500 MHz, $CDCl_3$, 70 °C) of compound **1**.

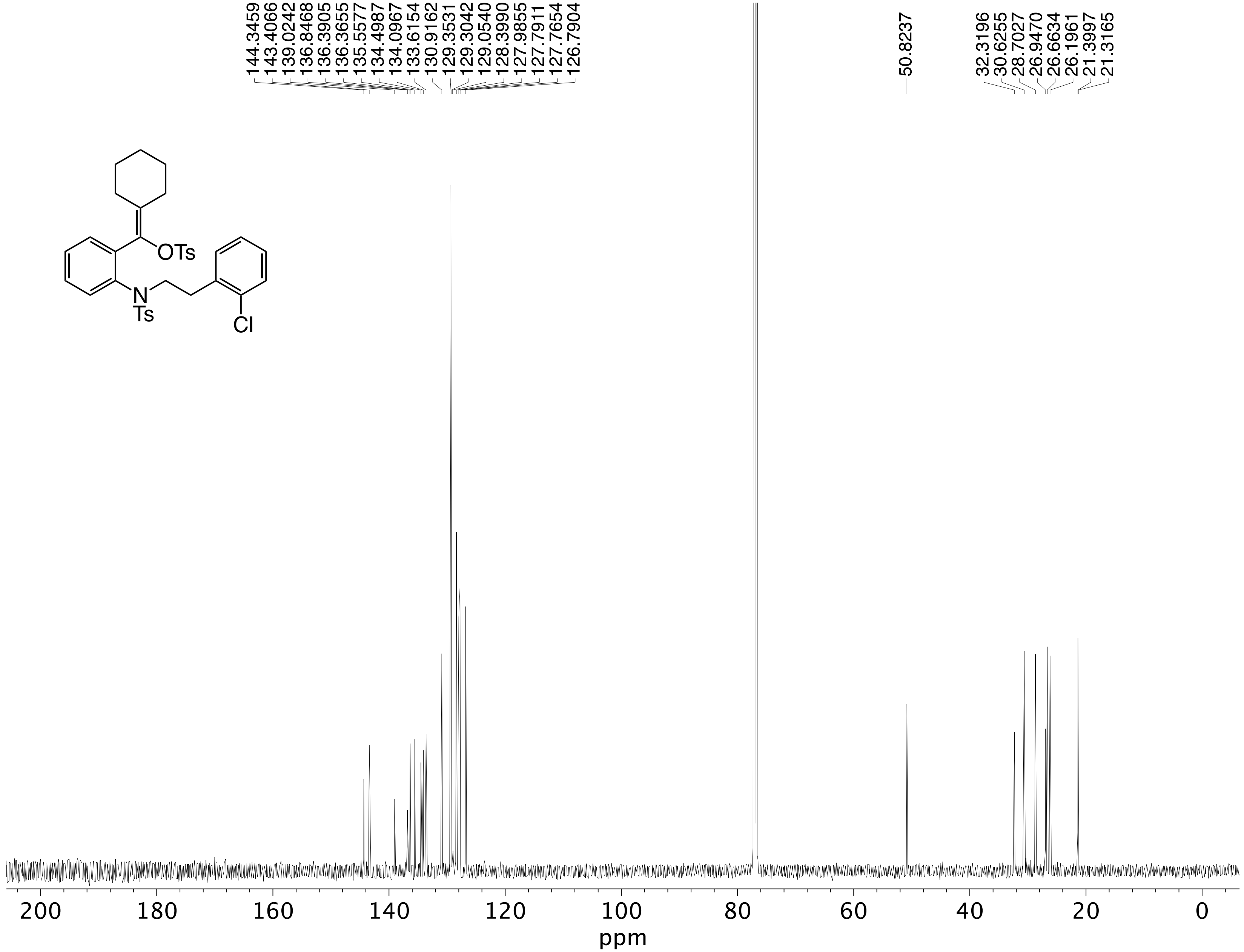

^{13}C NMR (126 MHz, $CDCl_3$) of compound **1**.

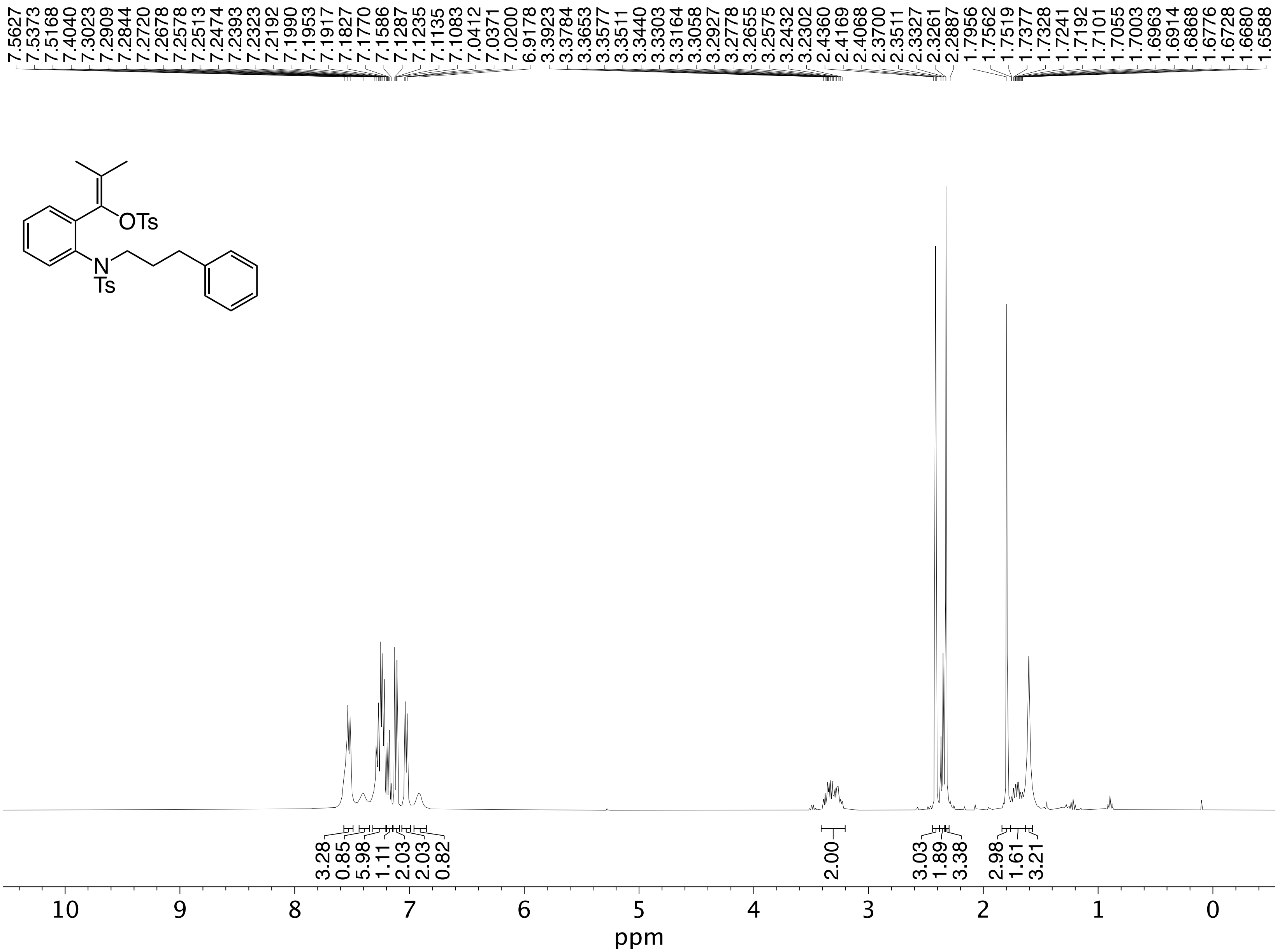

^{1}H NMR (400 MHz, $CDCl_3$) of compound **SI-11**.

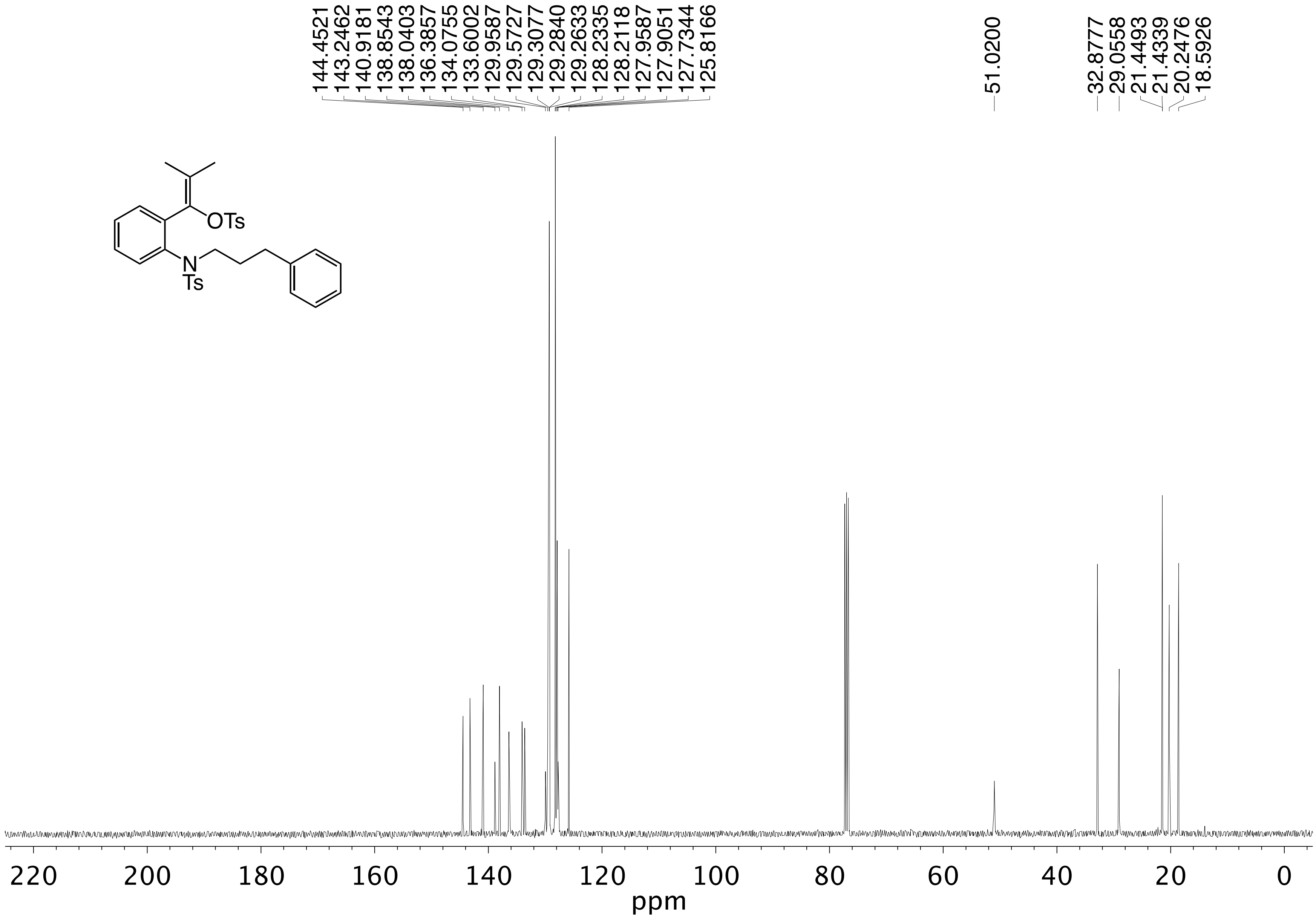

^{13}C NMR (101 MHz, $CDCl_3$) of compound **SI-11**.

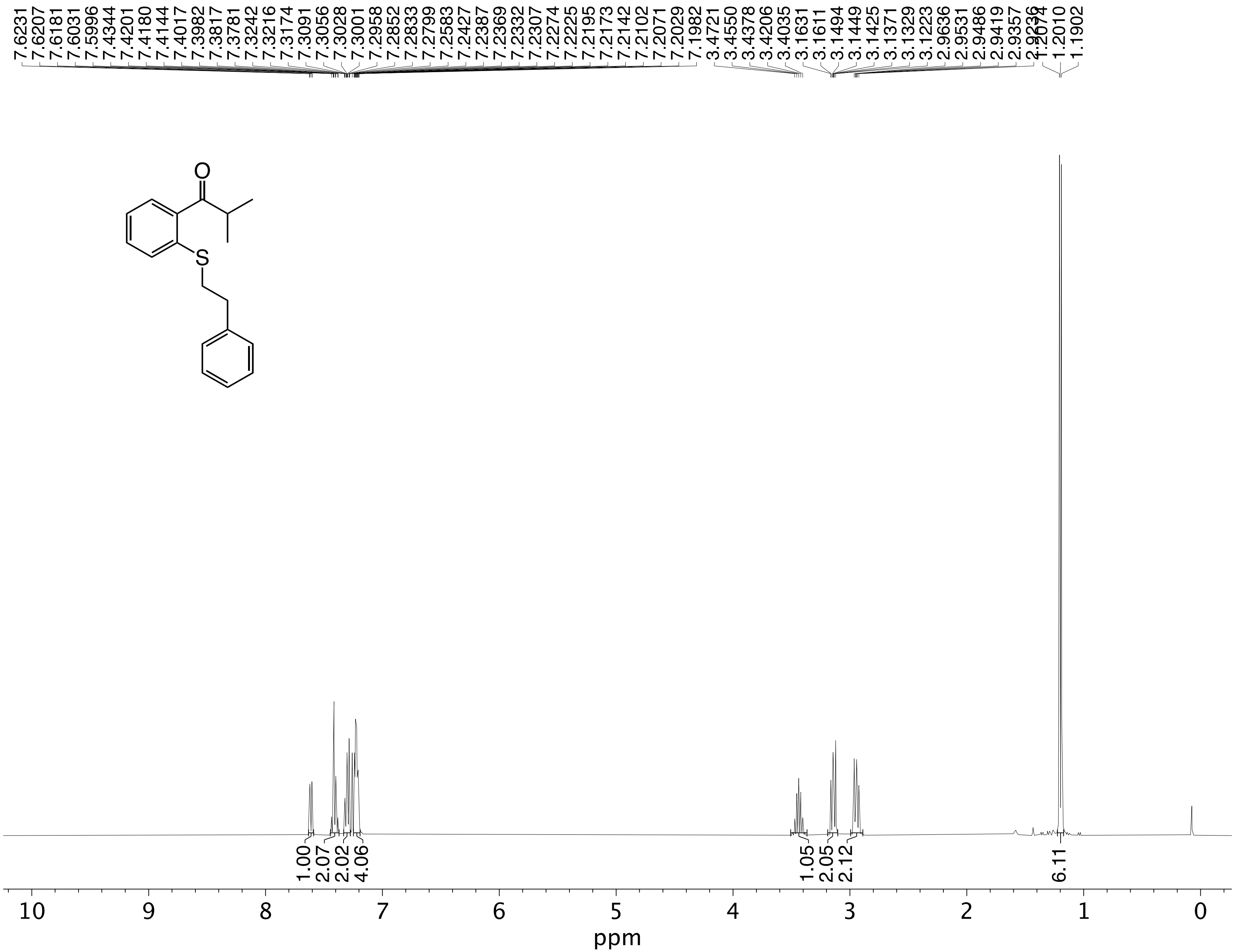

^{1}H NMR (400 MHz, $CDCl_3$) of compound **SI-13**.

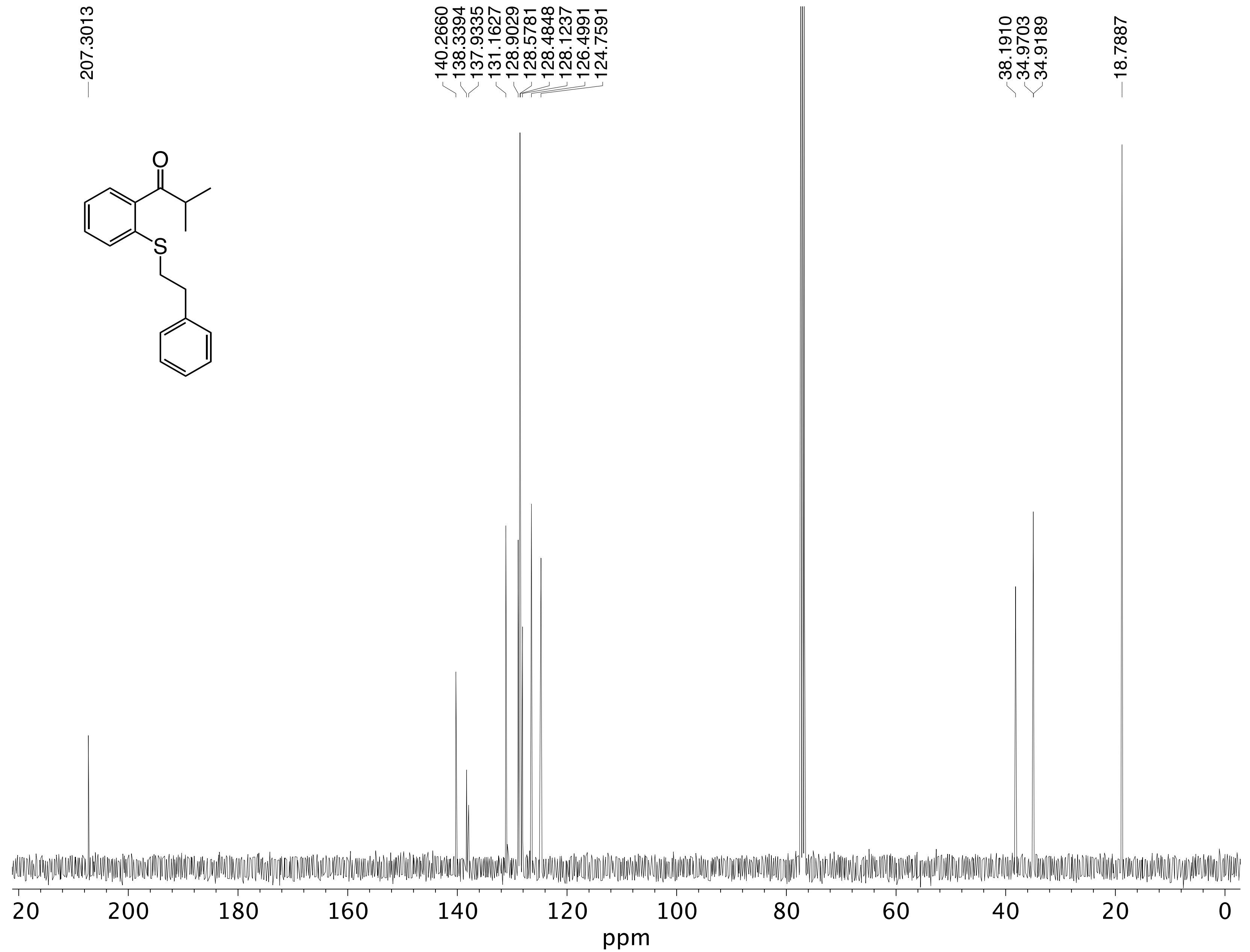

^{13}C NMR (101 MHz, $CDCl_3$) of compound **SI-13**.

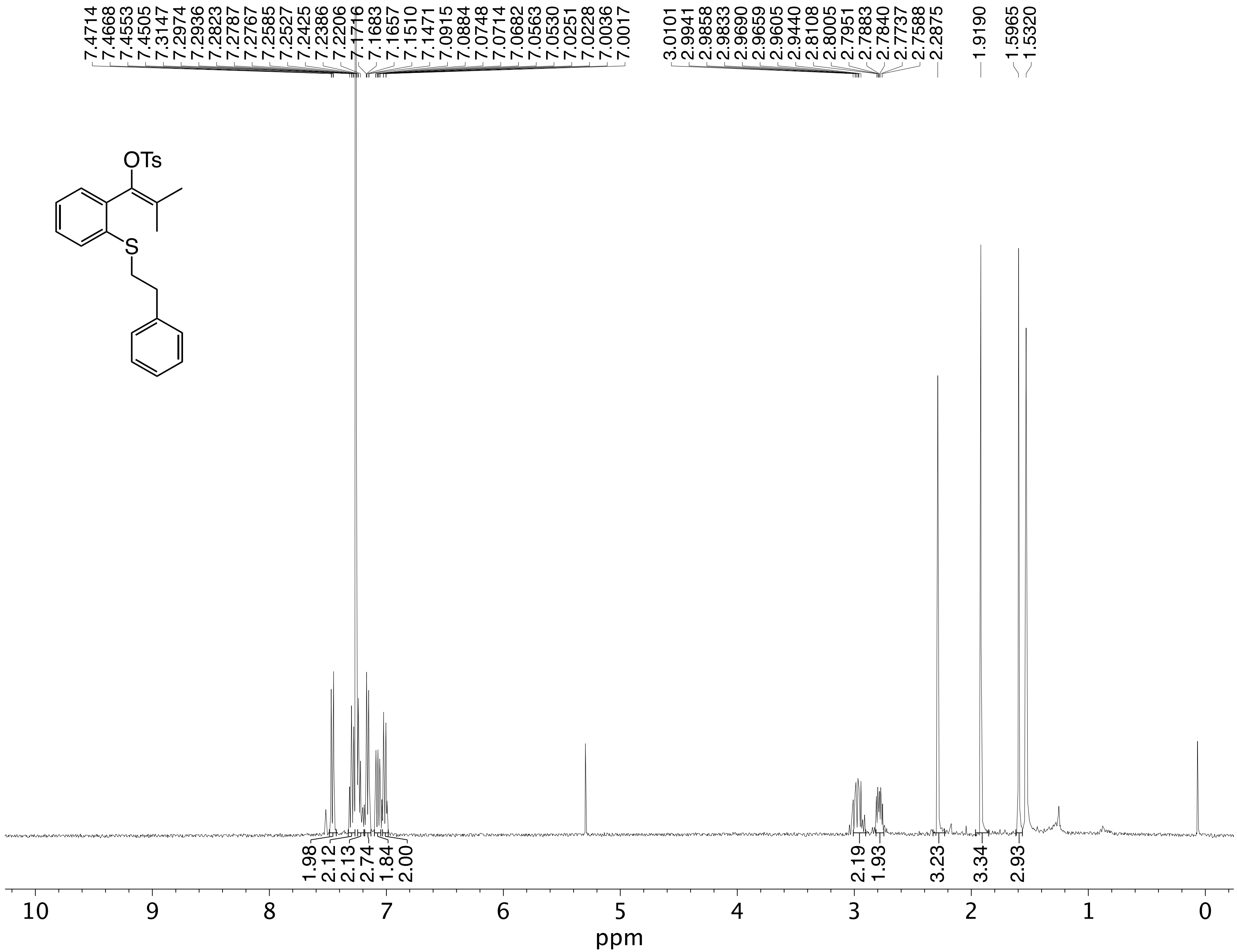

^{1}H NMR (400 MHz, $CDCl_3$) of compound **SI-14**.

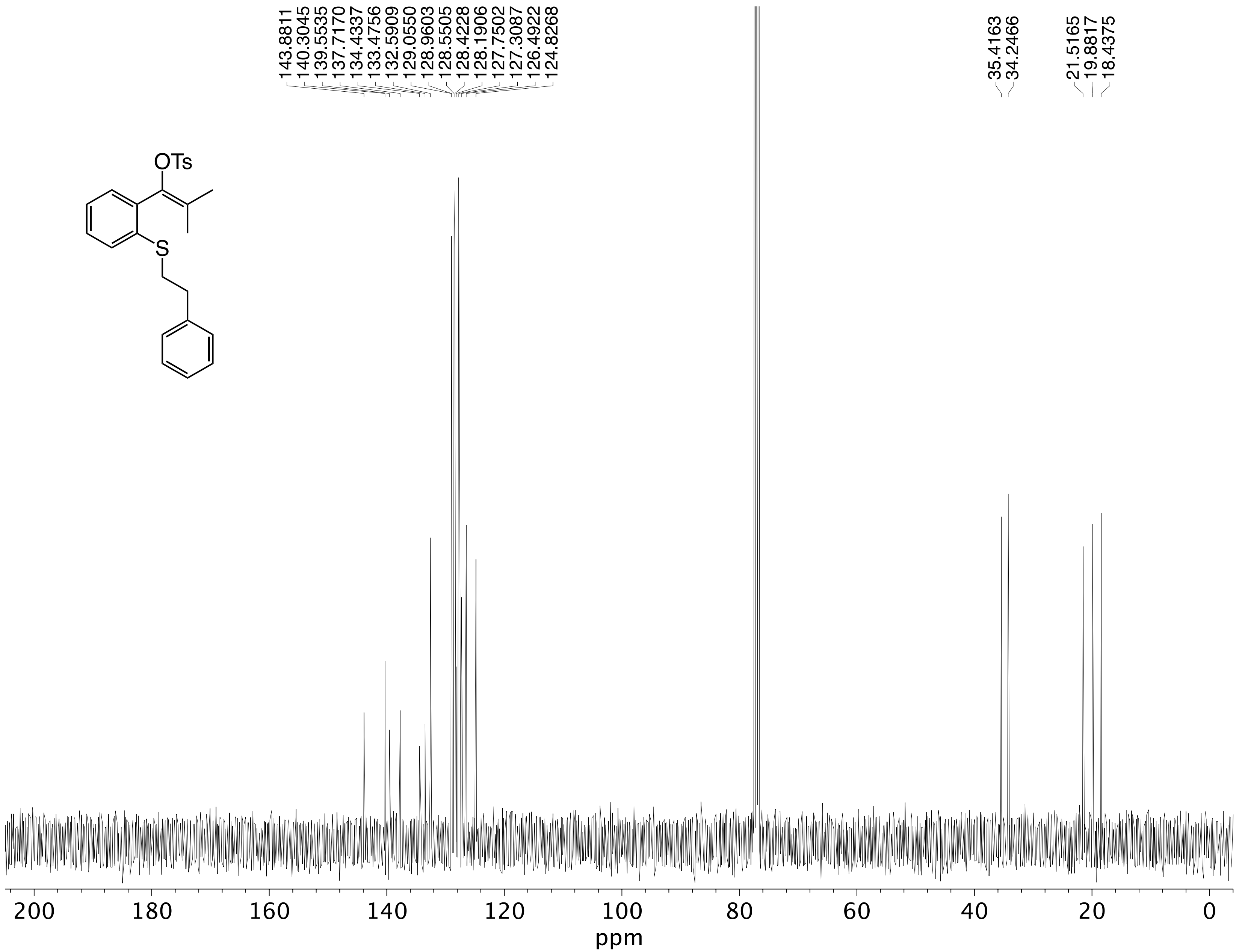

^{13}C NMR (101 MHz, $CDCl_3$) of compound **SI-14**.

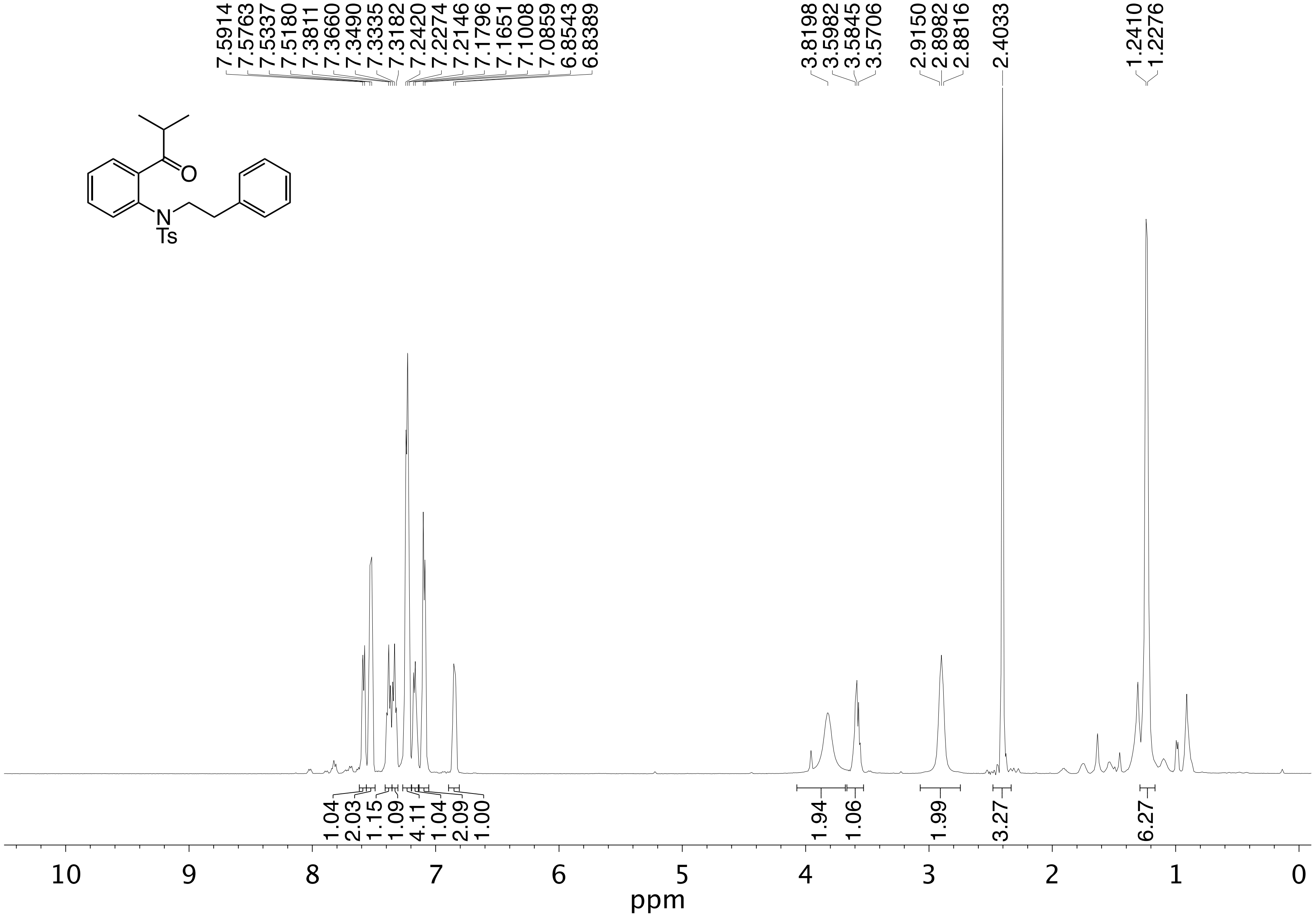

^{1}H NMR (500 MHz, $CDCl_3$, 70°C) of compound **SI-15**.

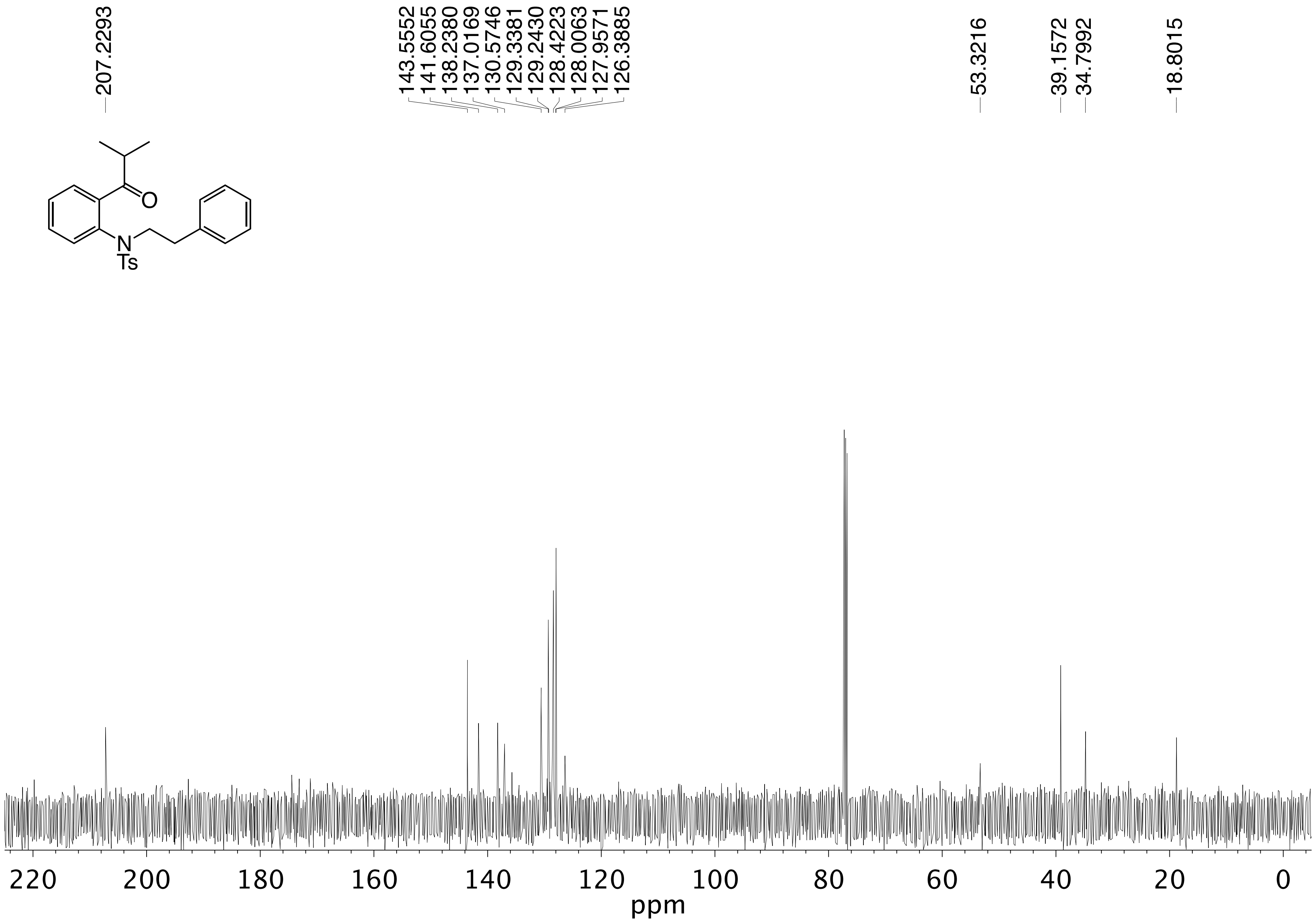

^{13}C NMR (126 MHz, $CDCl_3$, 70°C) of compound **SI-15**.

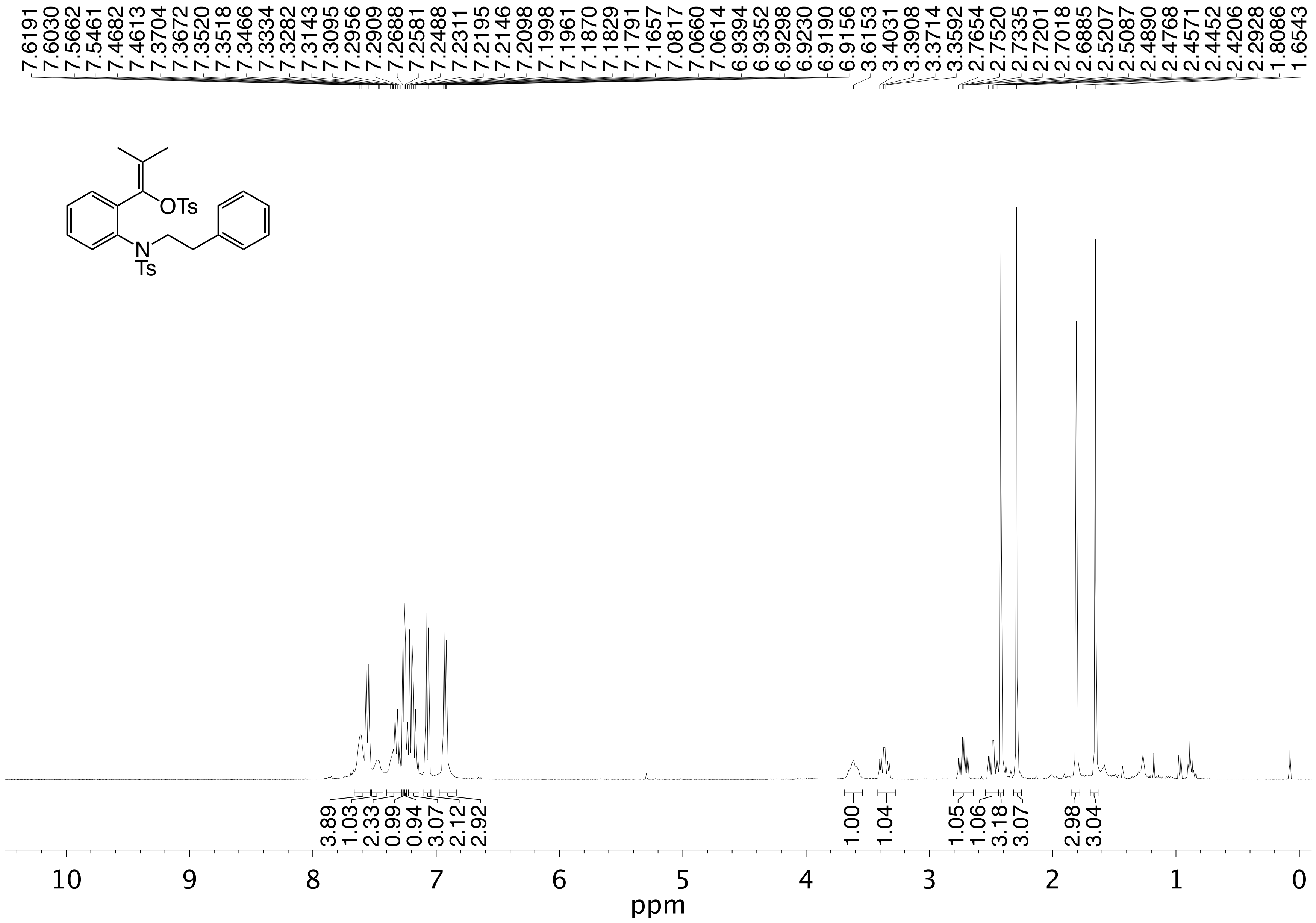

^{1}H NMR (400 MHz, $CDCl_3$) of compound **SI-16**.

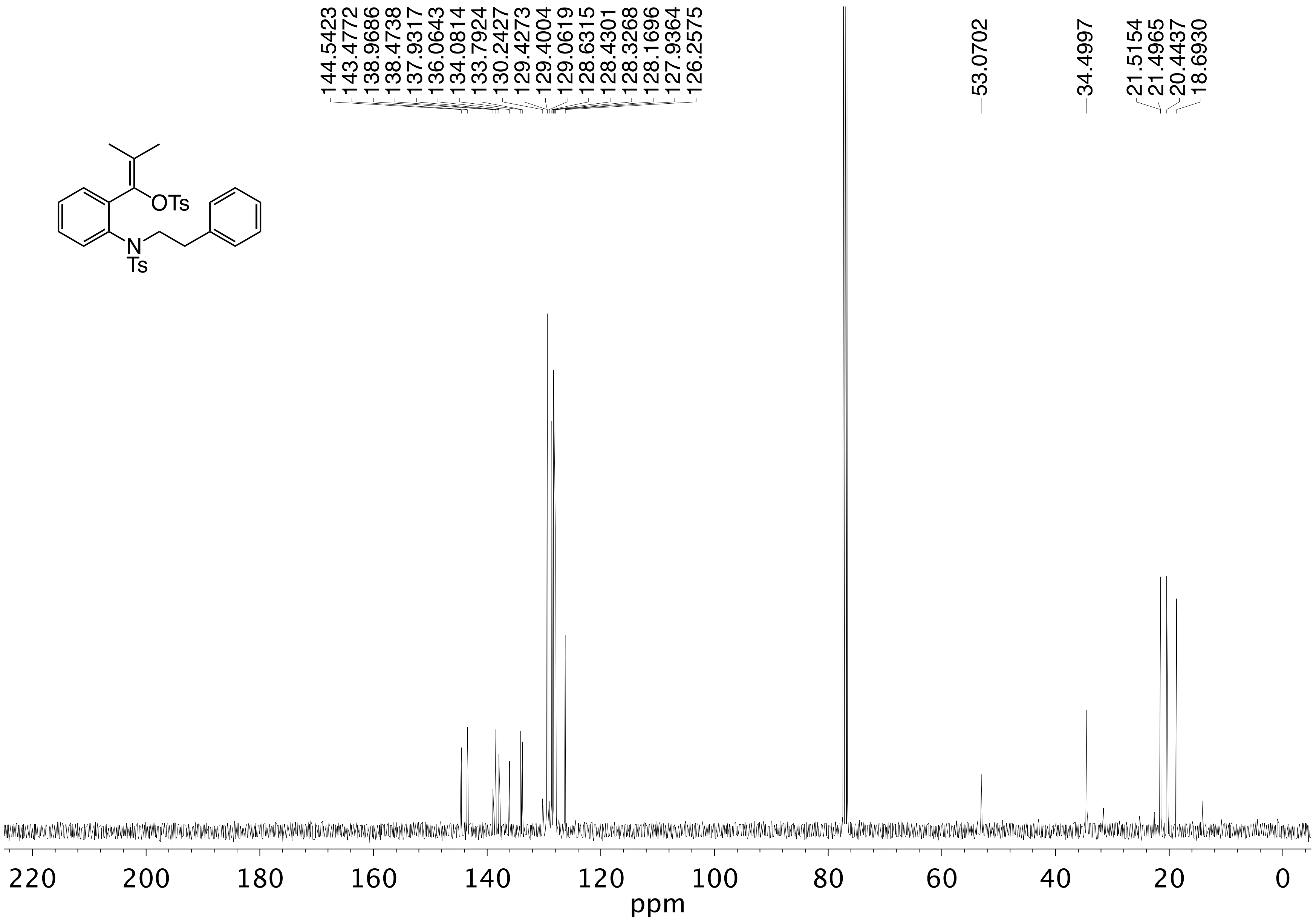

^{13}C NMR (101 MHz, $CDCl_3$) of compound **SI-16**.

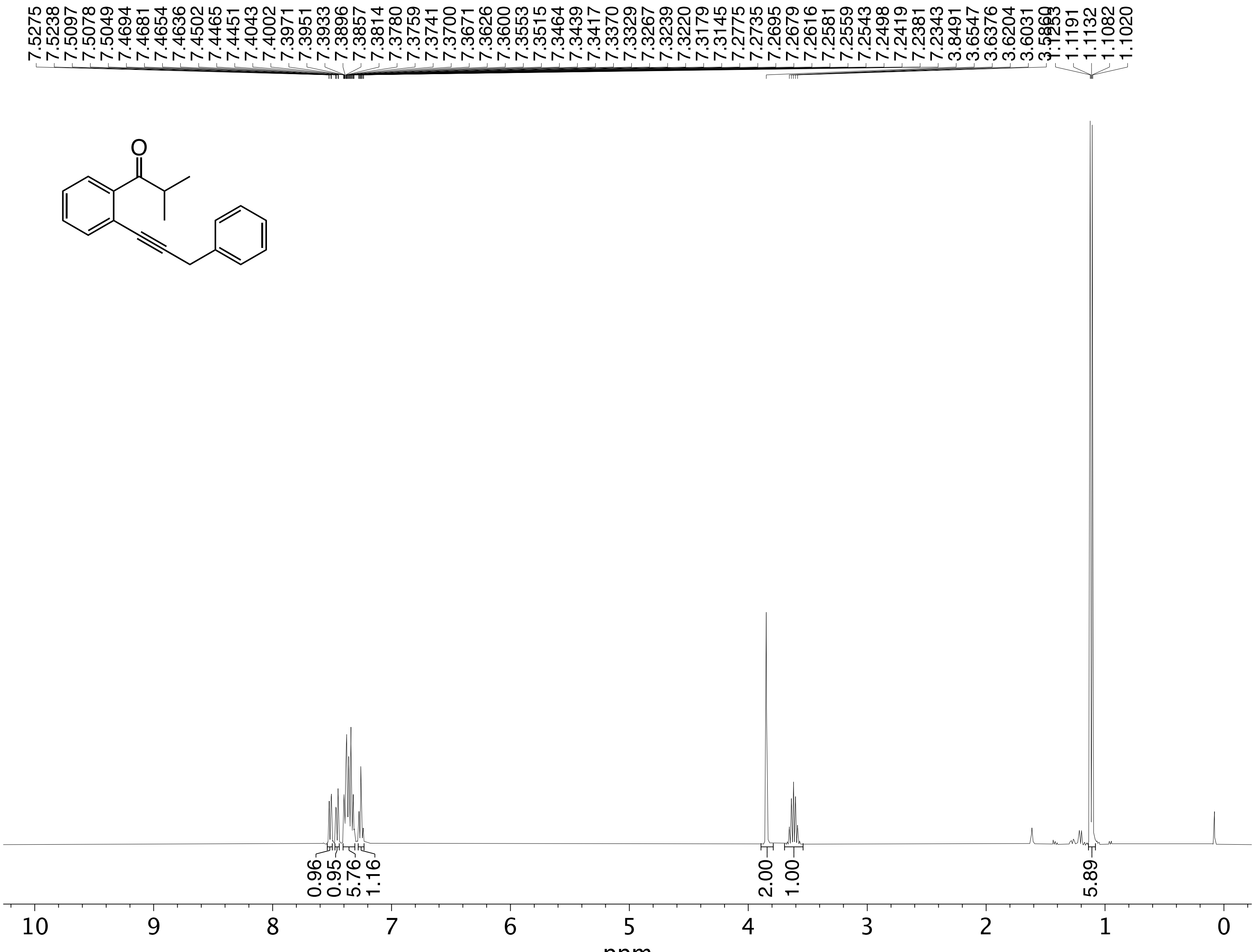

^{1}H NMR (400 MHz, $CDCl_3$) of compound **SI-18**.

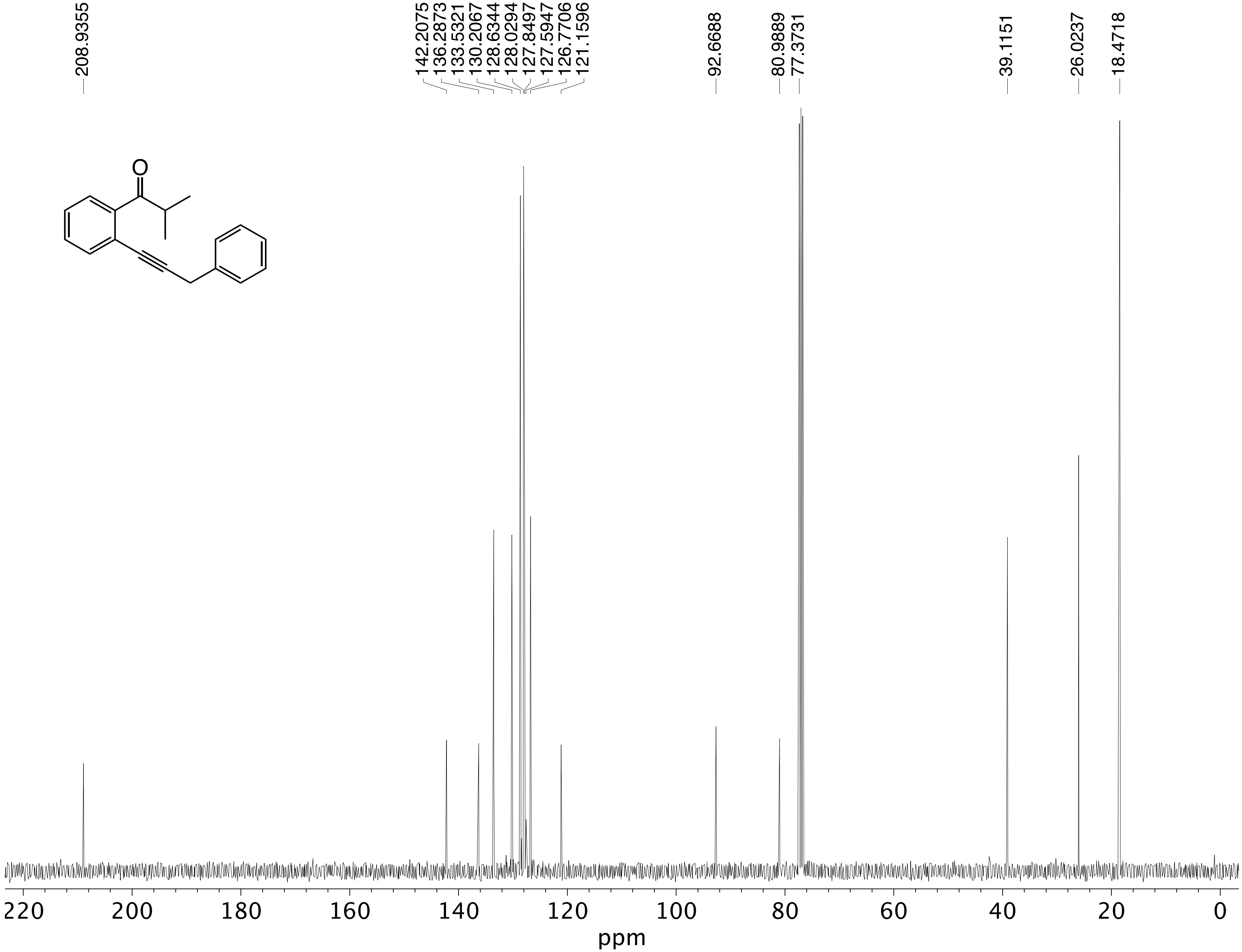

^{13}C NMR (101 MHz, $CDCl_3$) of compound **SI-18**.

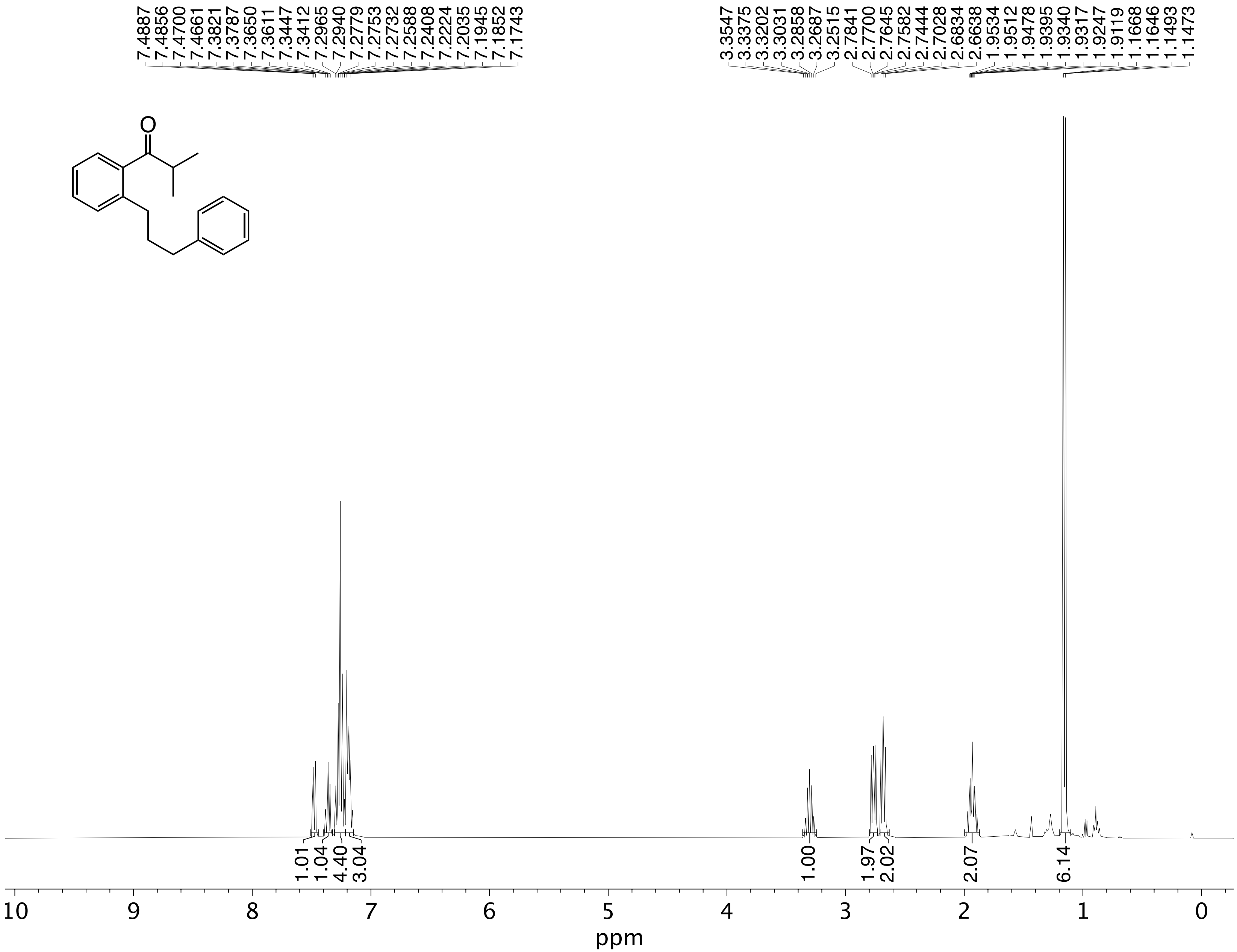

^{1}H NMR (400 MHz, $CDCl_3$) of compound **SI-19**.

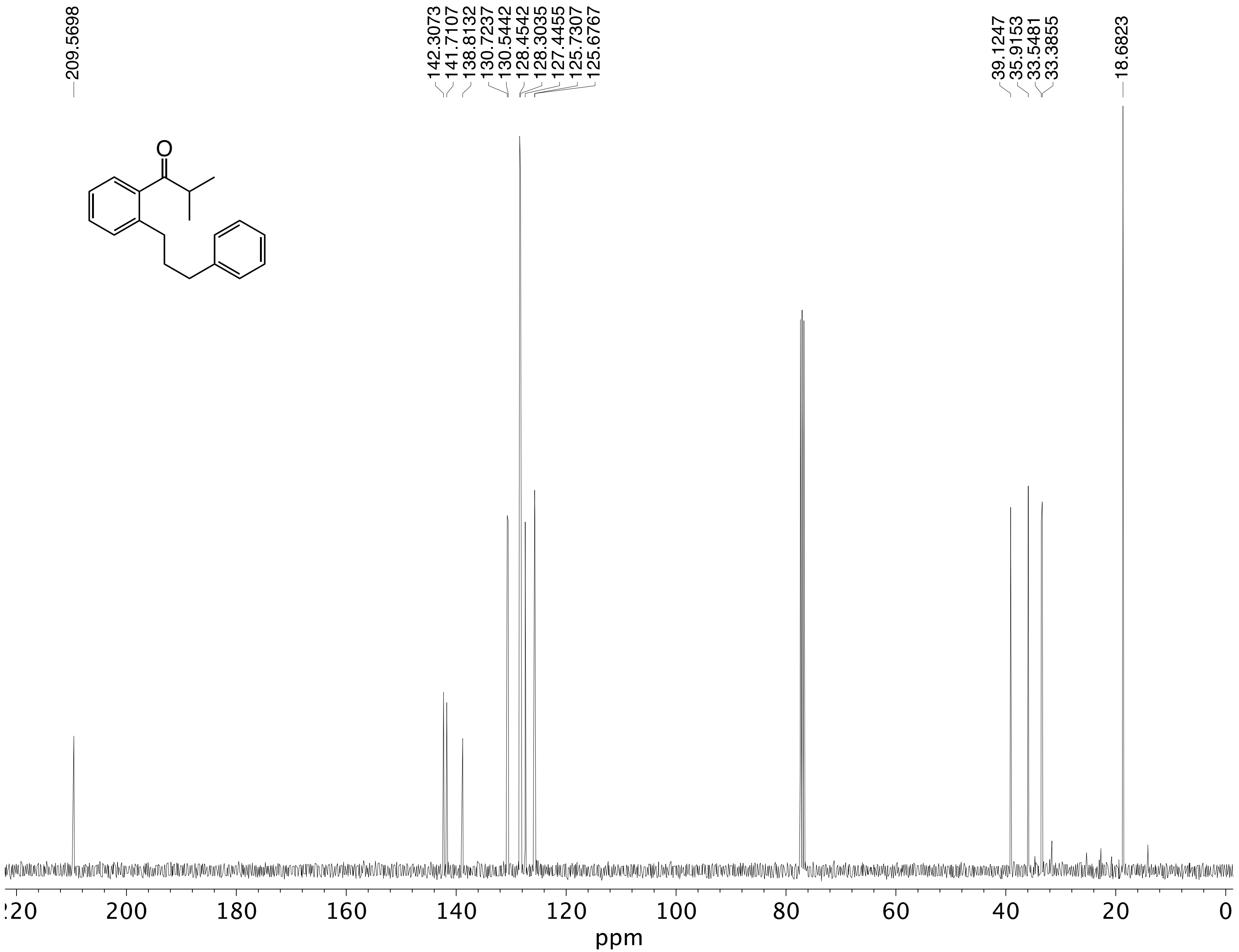

^{13}C NMR (101 MHz, $CDCl_3$) of compound **SI-19**.

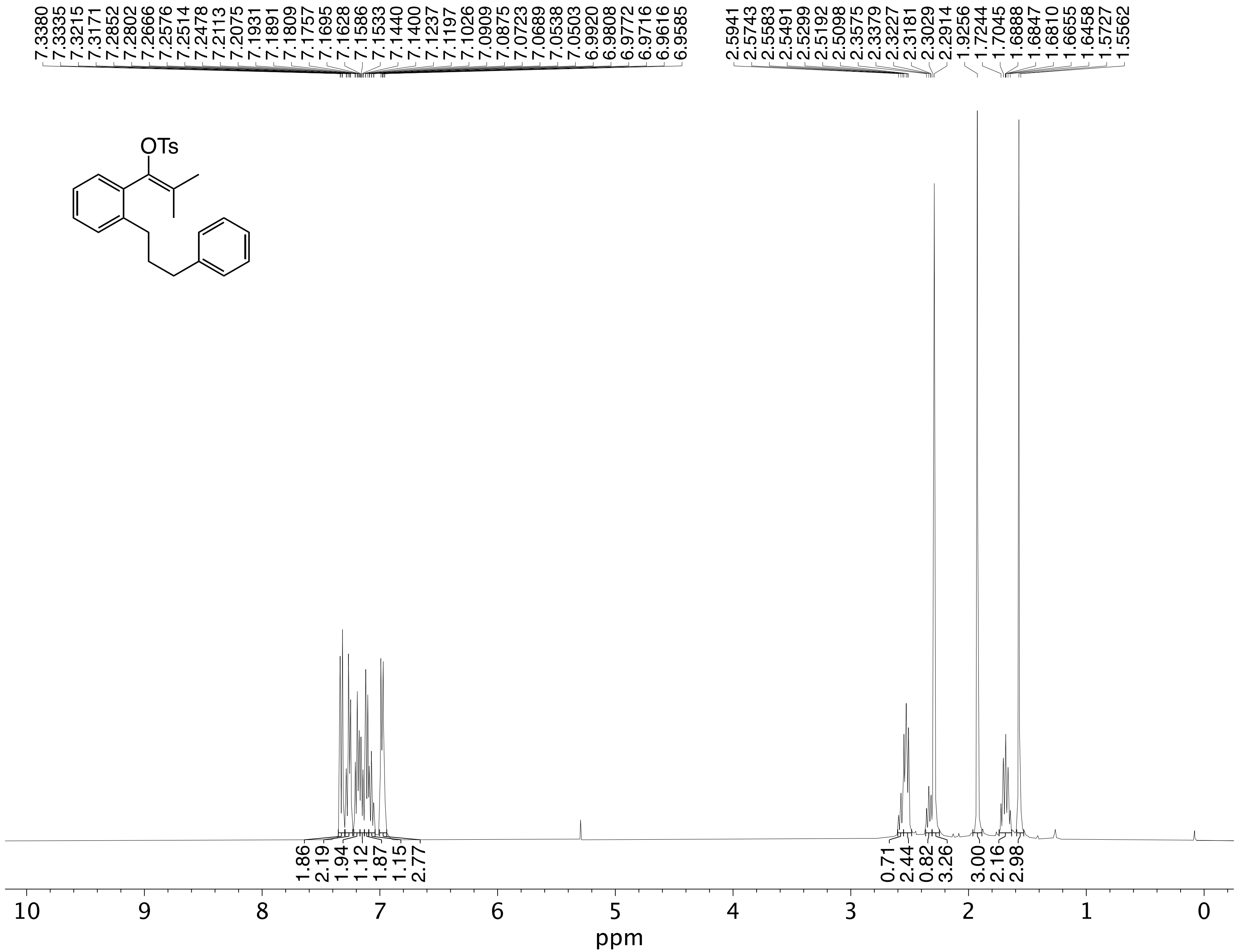

^1H NMR (400 MHz, $CDCl_3$) of compound **SI-20**.

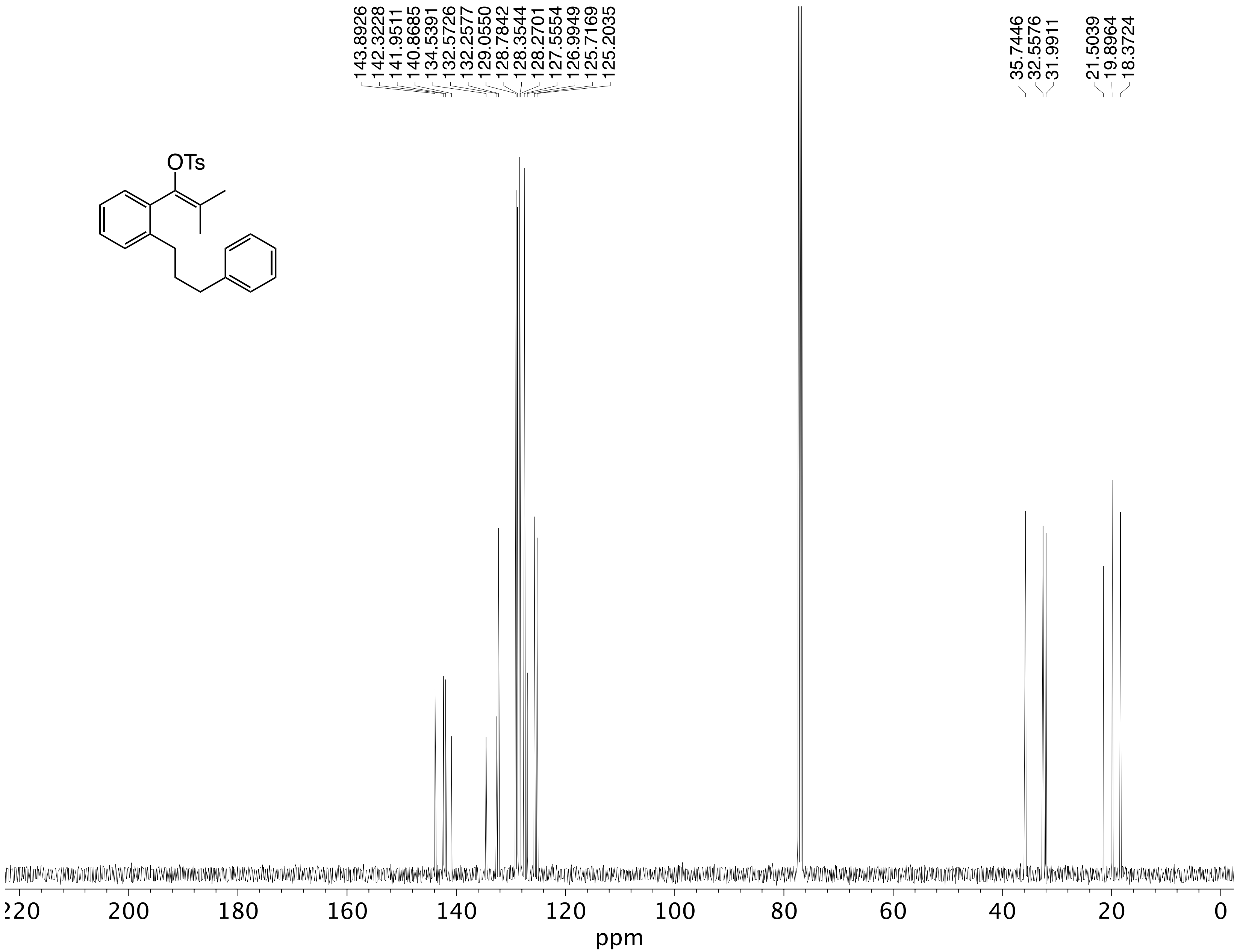

^{13}C NMR (101 MHz, $CDCl_3$) of compound **SI-20**.

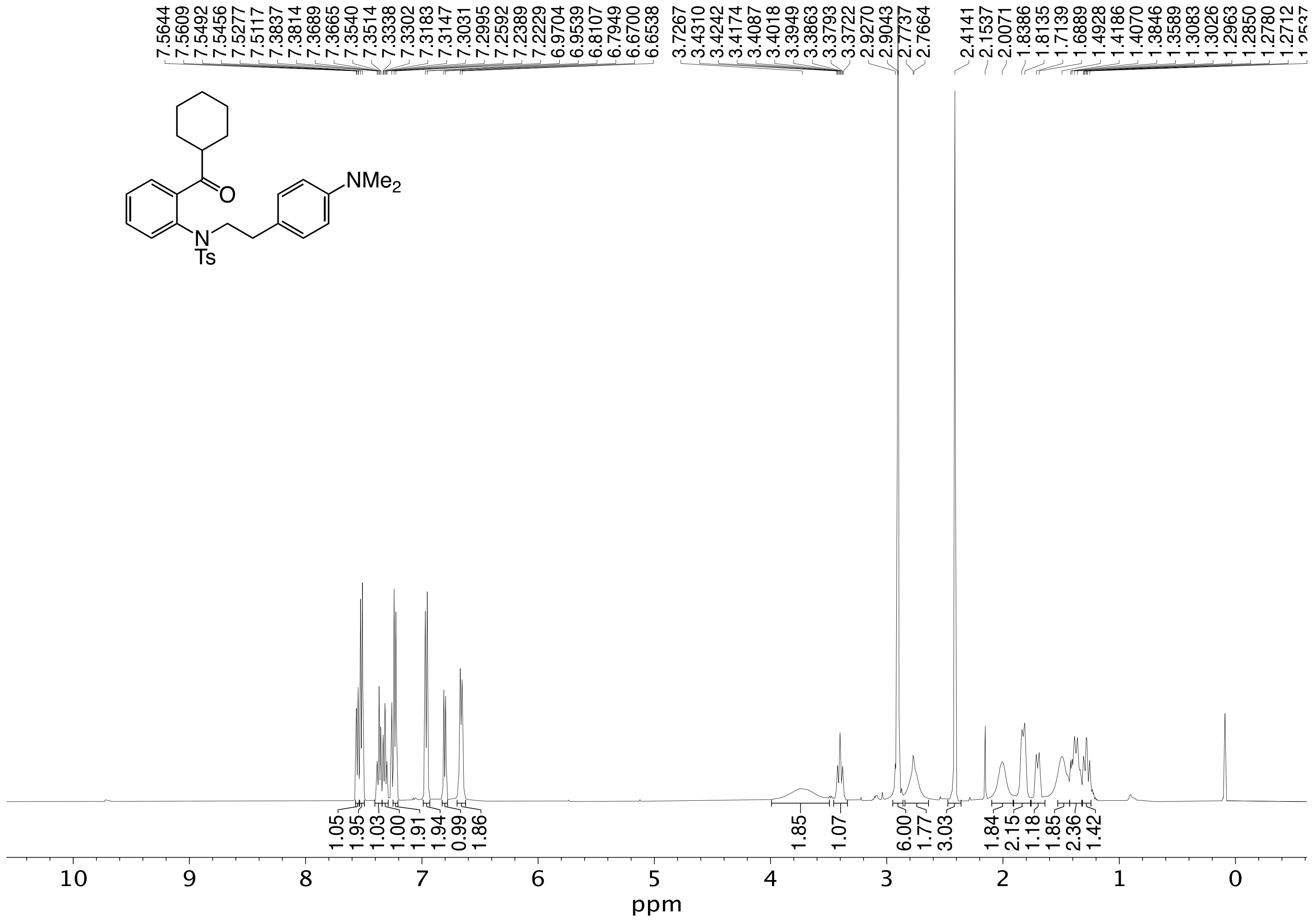

^{1}H NMR (500 MHz, $CDCl_3$, 60°C) of compound **SI-23**.

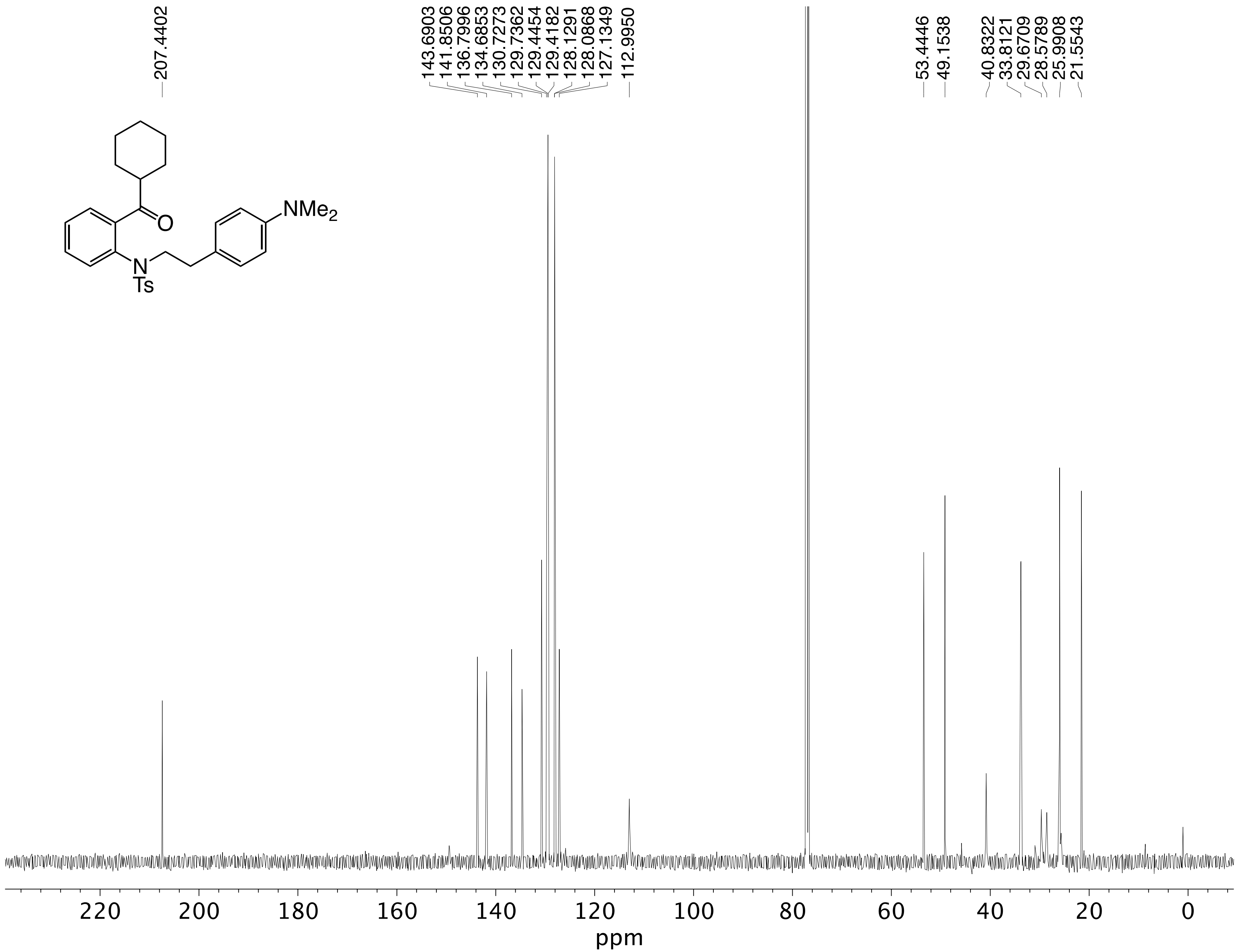

^{13}C NMR (126 MHz, $CDCl_3$) of compound **SI-23**.

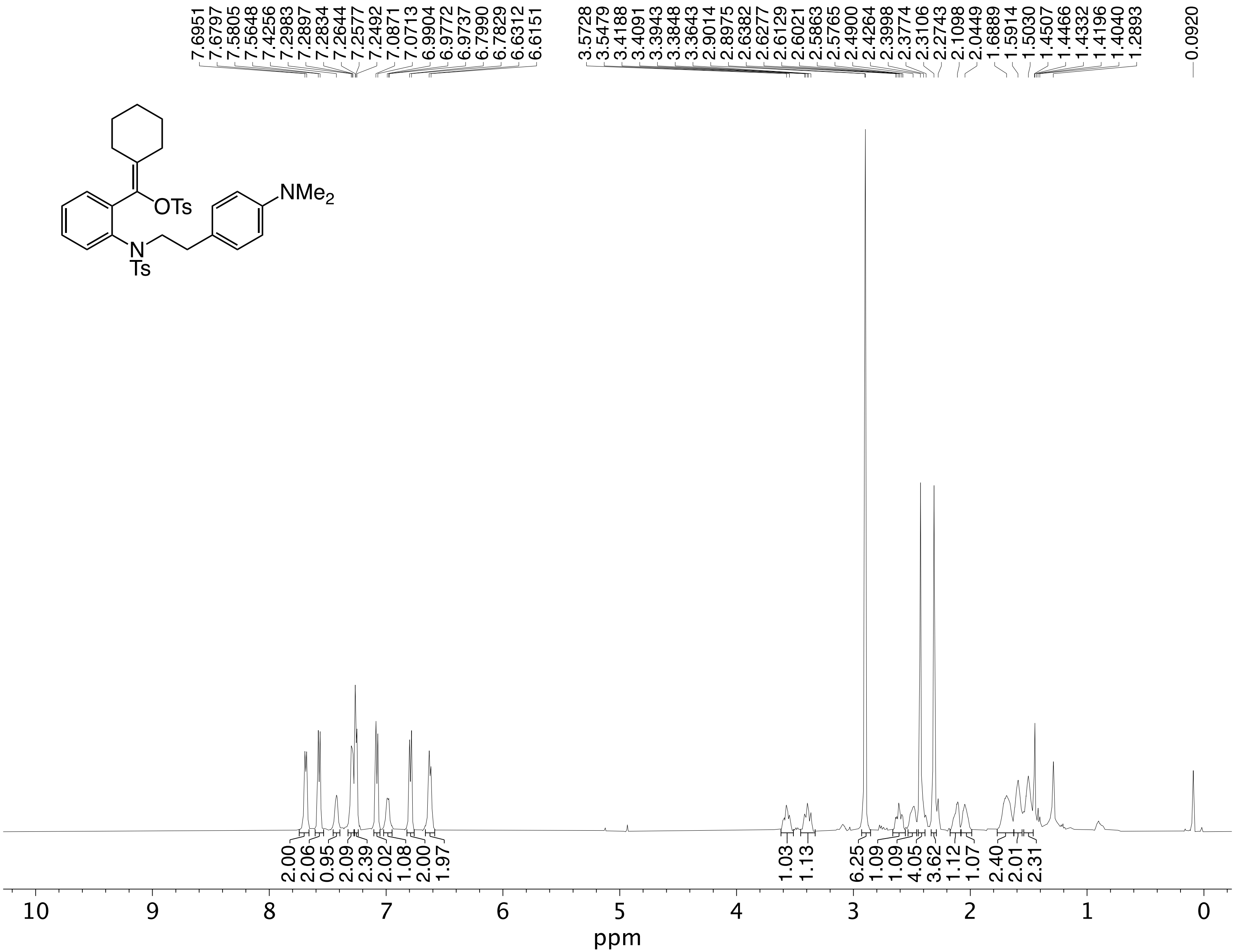

^{1}H NMR (500 MHz, $CDCl_3$, 70°C) of compound **SI-24**.

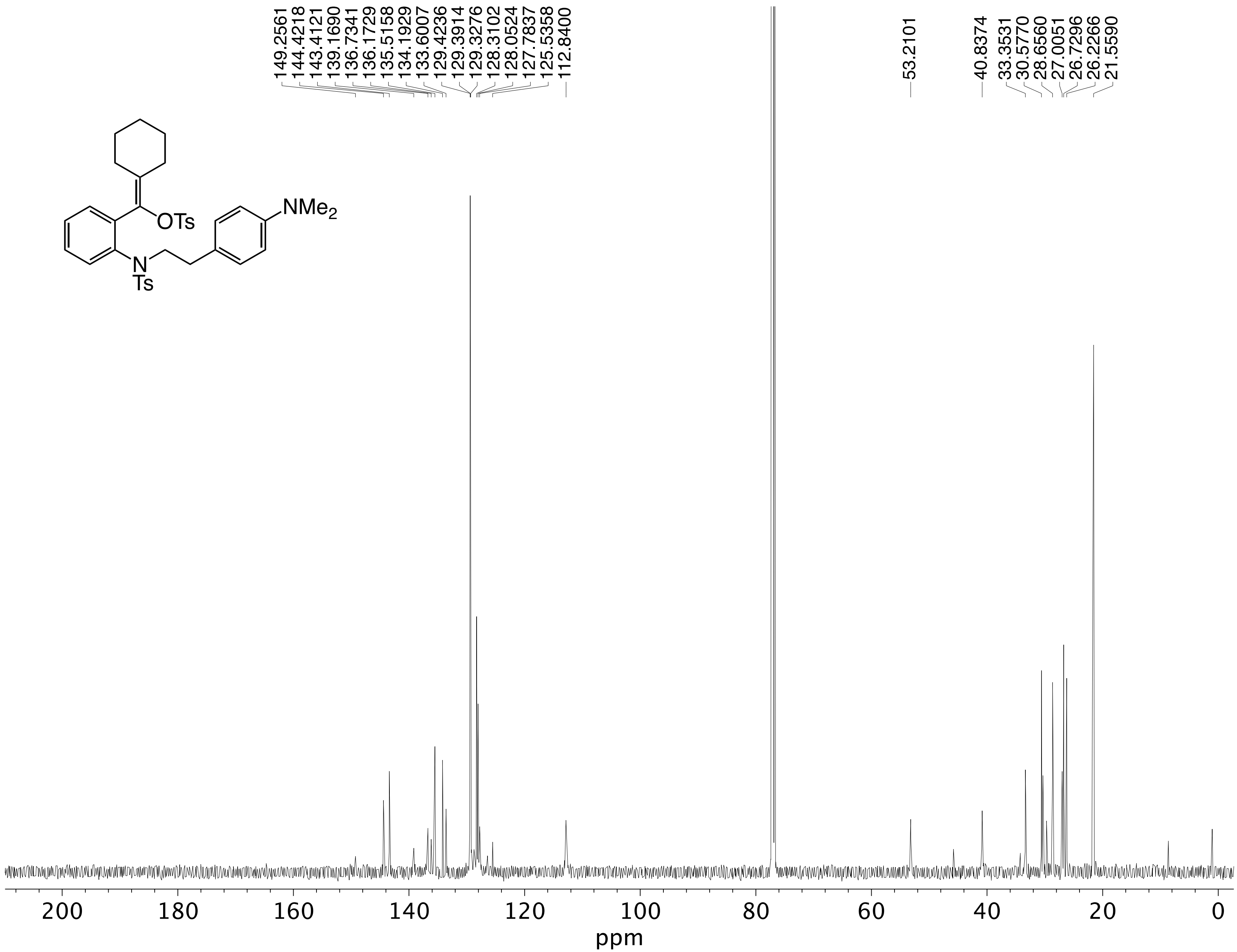

^{13}C NMR (126 MHz, $CDCl_3$) of compound **SI-24**.

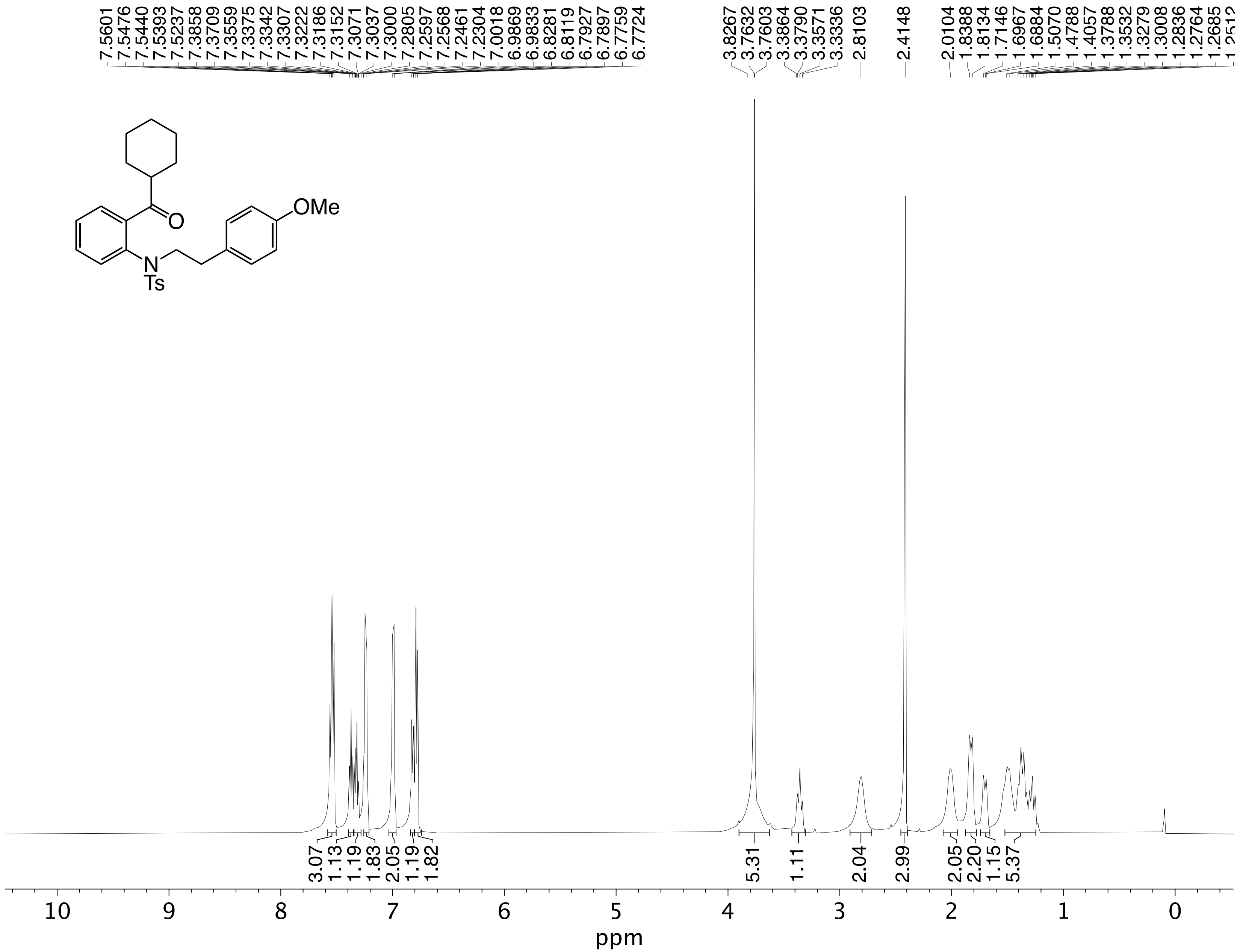

^{1}H NMR (500 MHz, $CDCl_3$, 70°C) of compound **SI-26**.

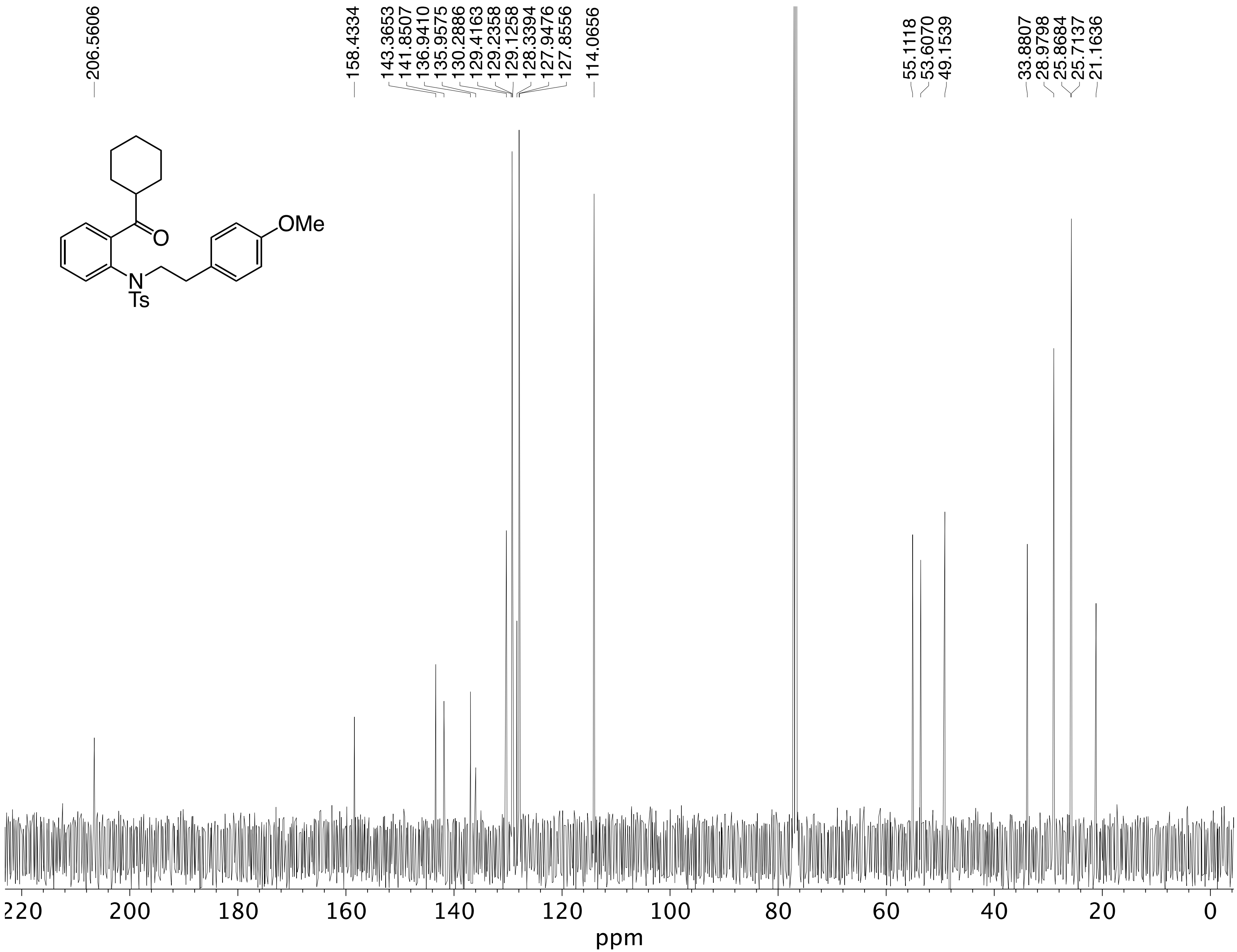

^{13}C NMR (126 MHz, $CDCl_3$, 70°C) of compound **SI-26**.

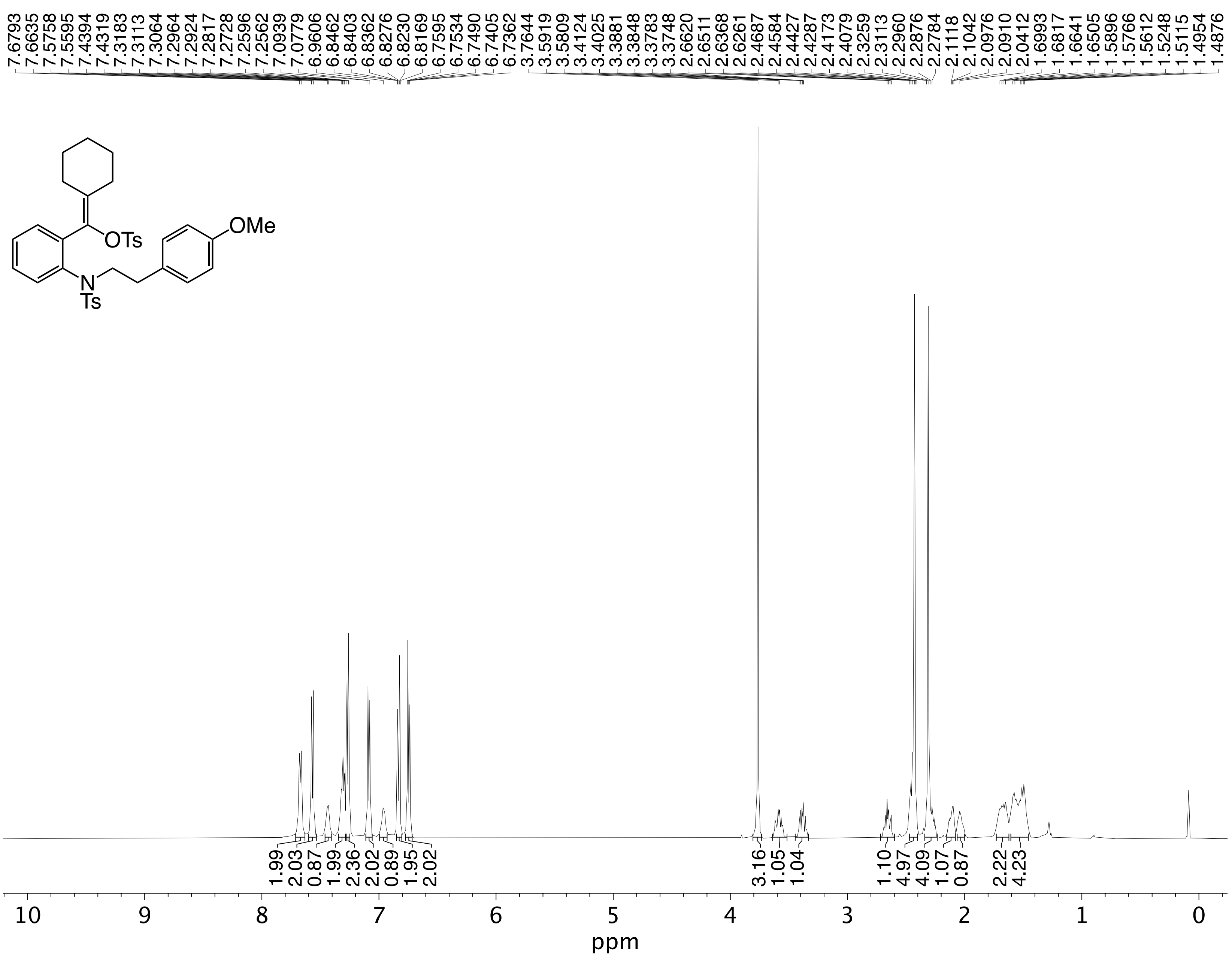

^1H NMR (500 MHz, $CDCl_3$, 50°C) of compound **SI-27**.

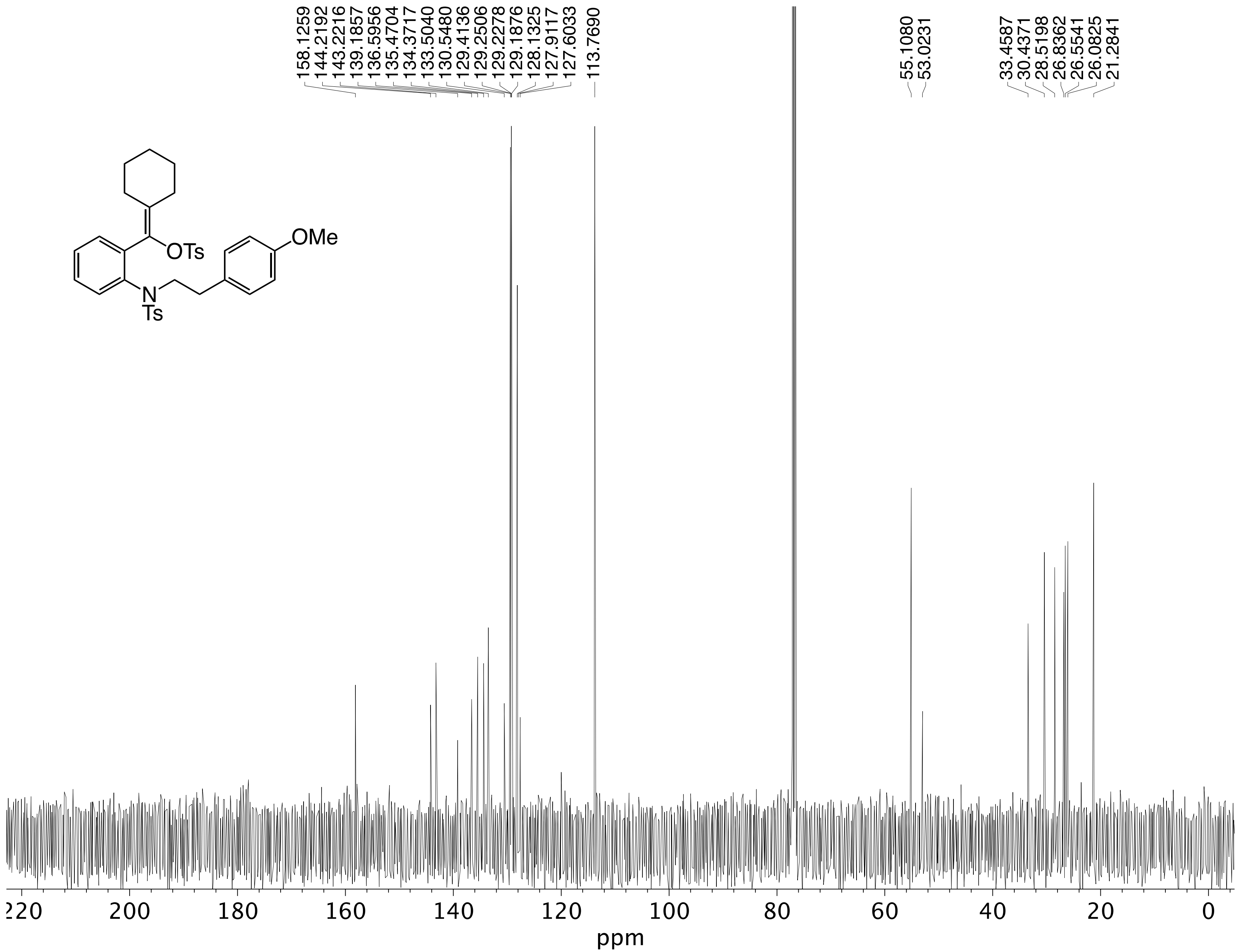

^{13}C NMR (126 MHz, $CDCl_3$, 50°C) of compound **SI-27**.

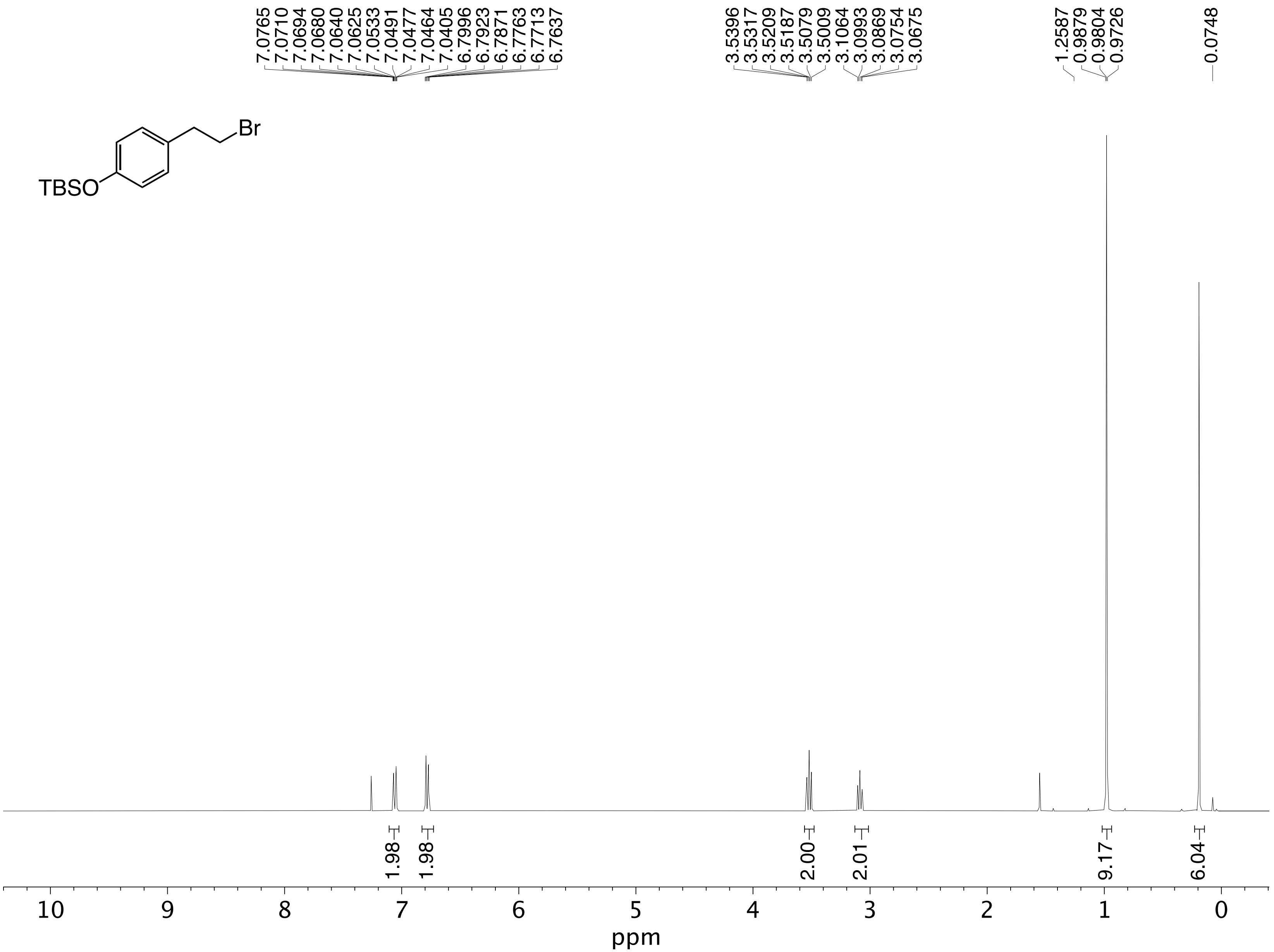

^{1}H NMR (400 MHz, $CDCl_3$) of compound **SI-30**.

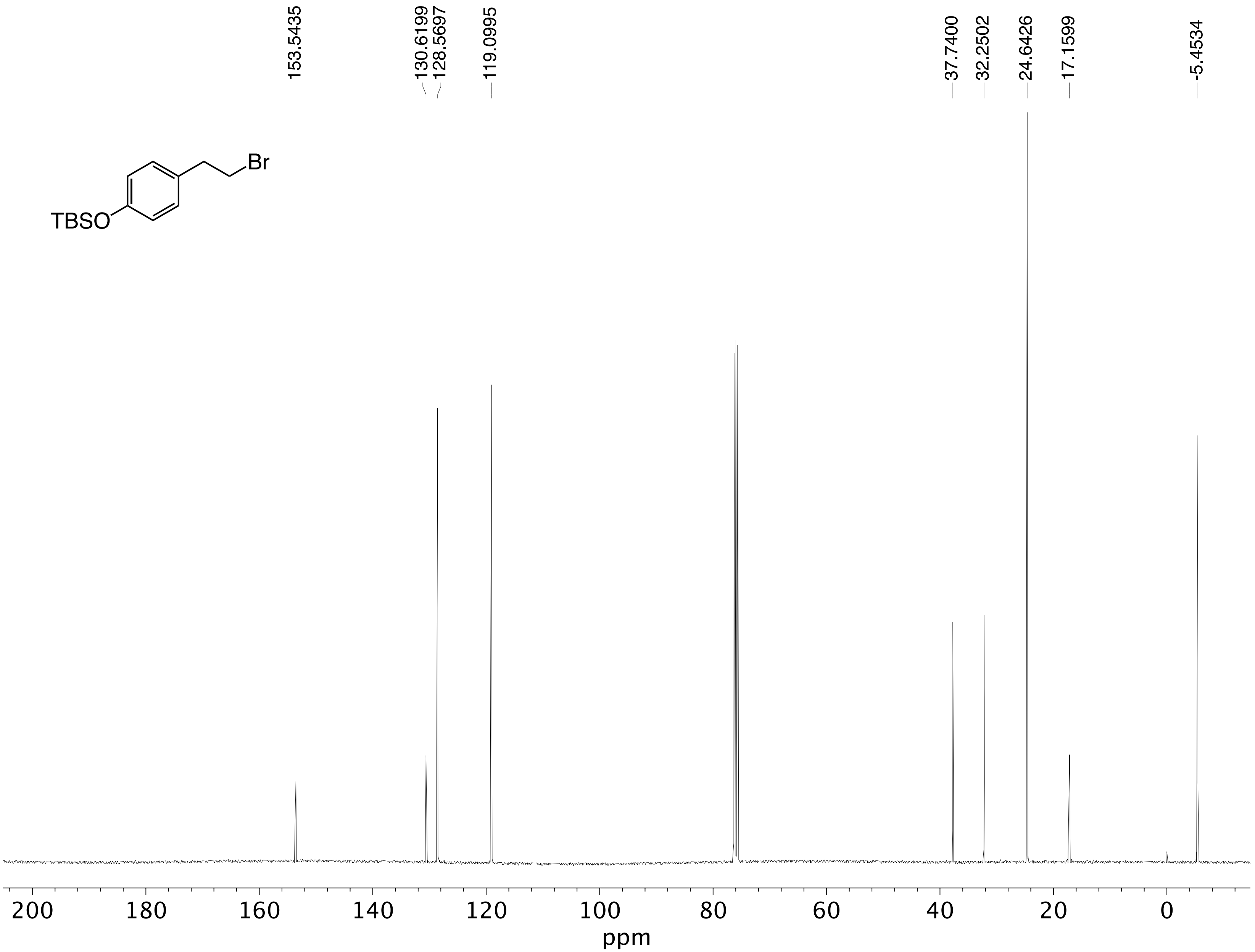

^{13}C NMR (101 MHz, $CDCl_3$) of compound **SI-30**.

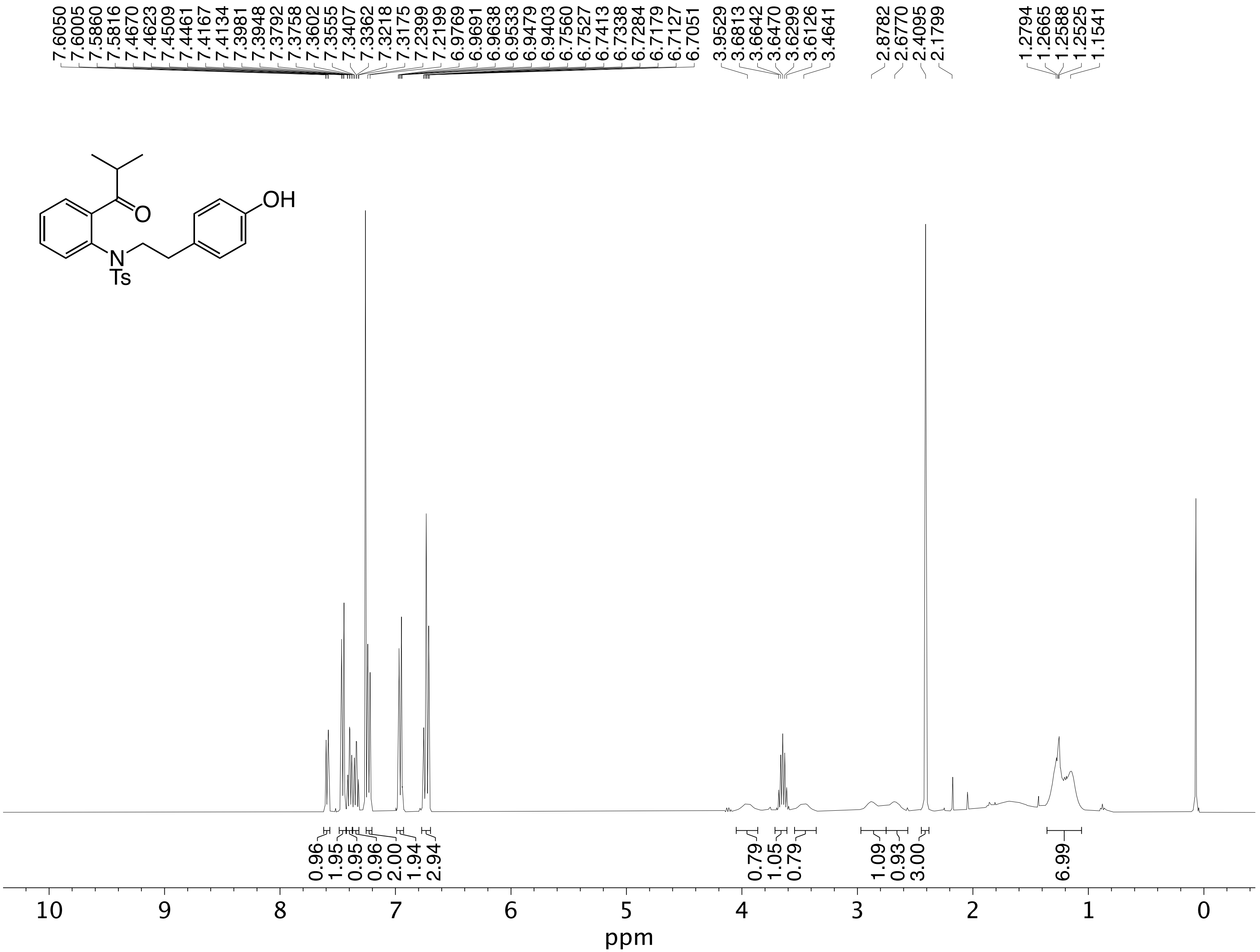

^{1}H NMR (400 MHz, $CDCl_3$) of compound **SI-31**.

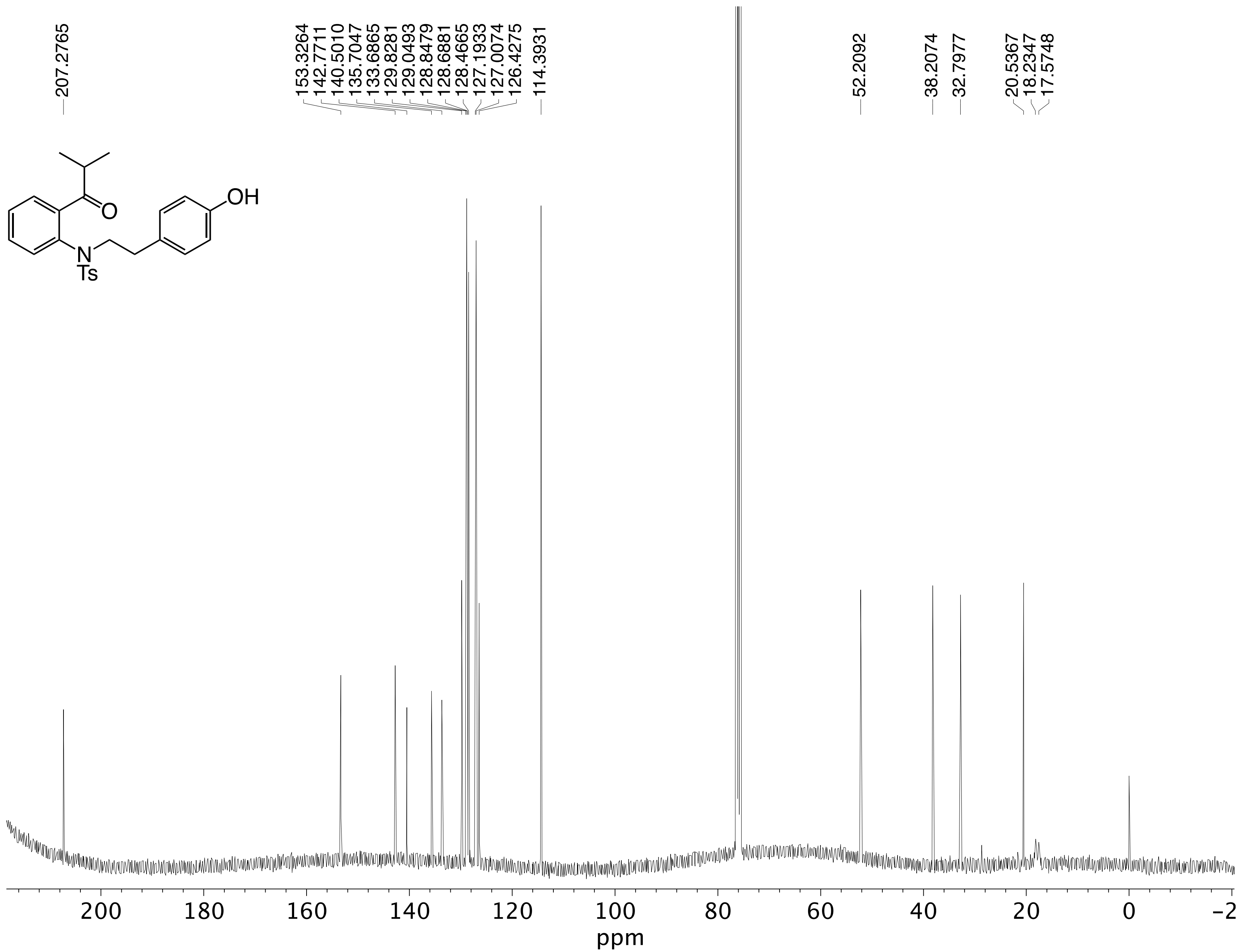

^{13}C NMR (101 MHz, $CDCl_3$) of compound **SI-31**.

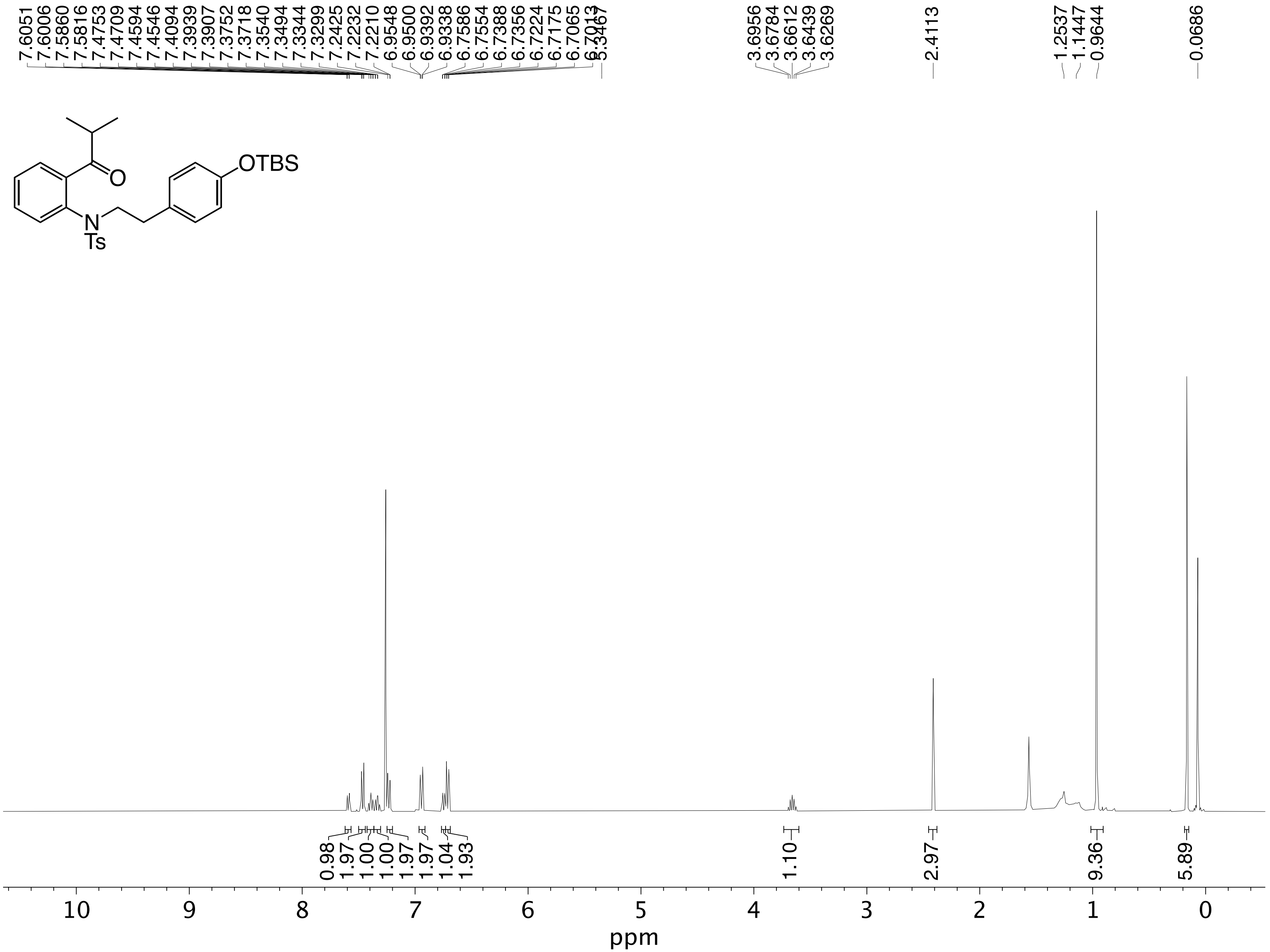

^{1}H NMR (400 MHz, $CDCl_3$) of compound **SI-32**.

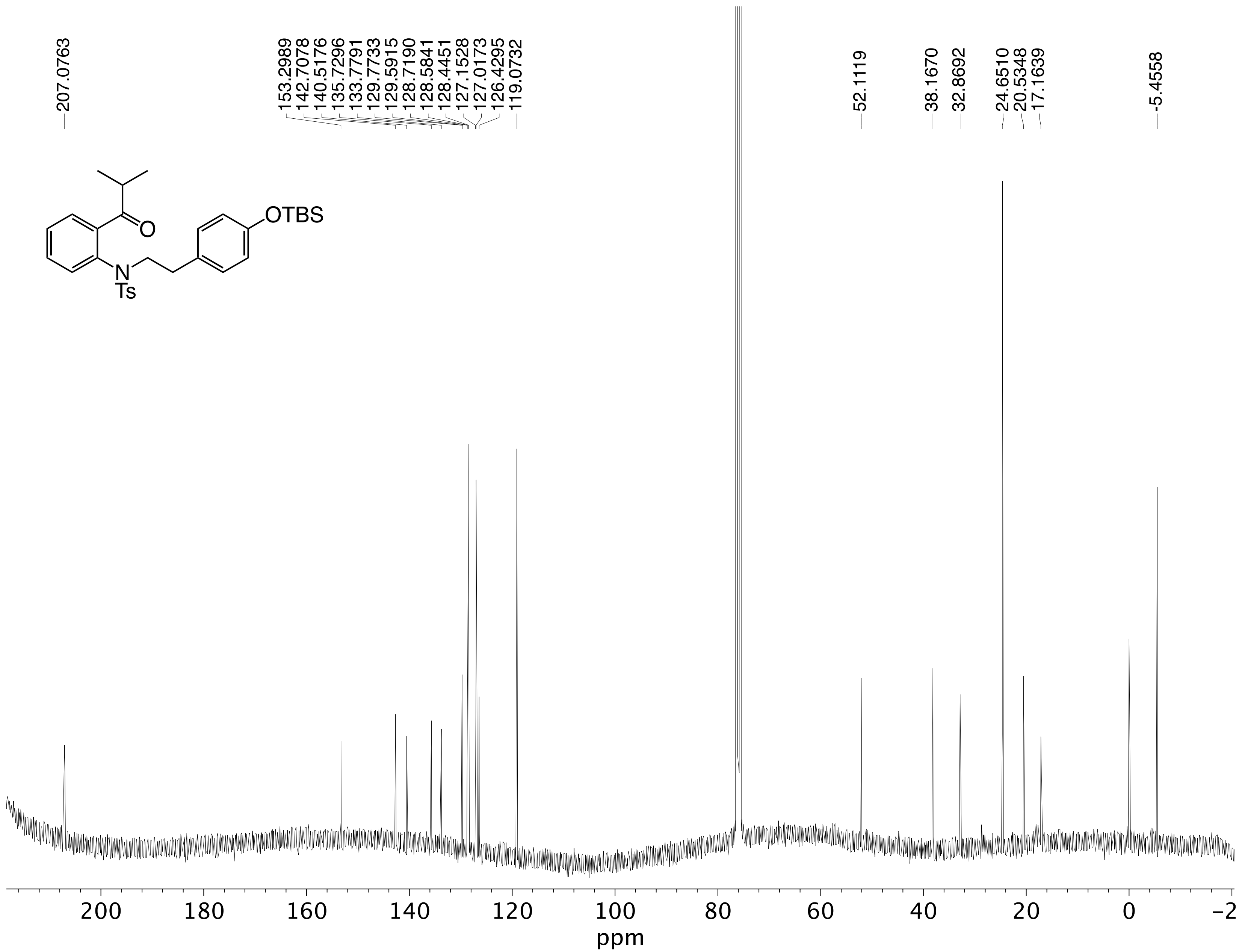

^{13}C NMR (101 MHz, $CDCl_3$) of compound **SI-32**.

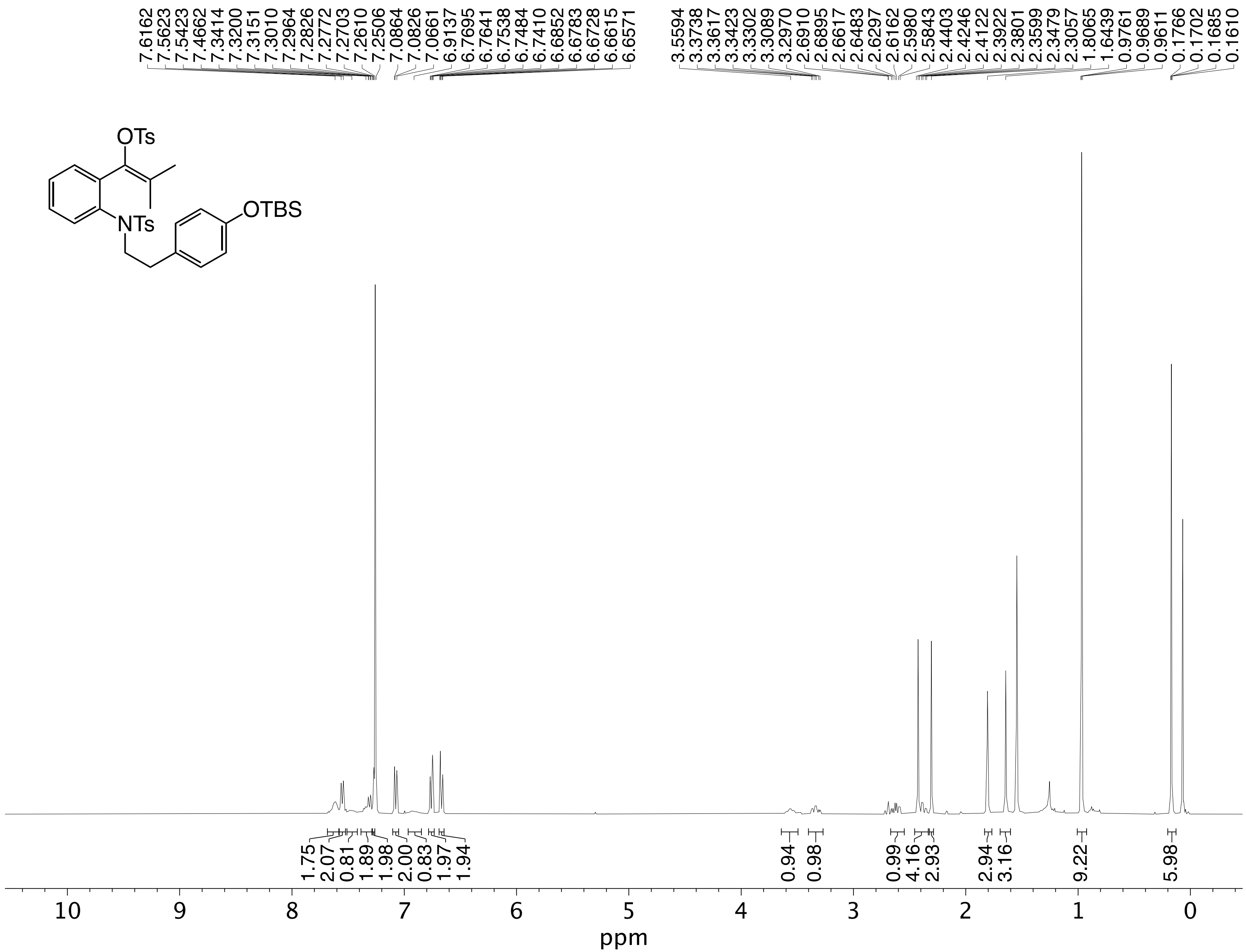

^{1}H NMR (400 MHz, $CDCl_3$) of compound **SI-33**.

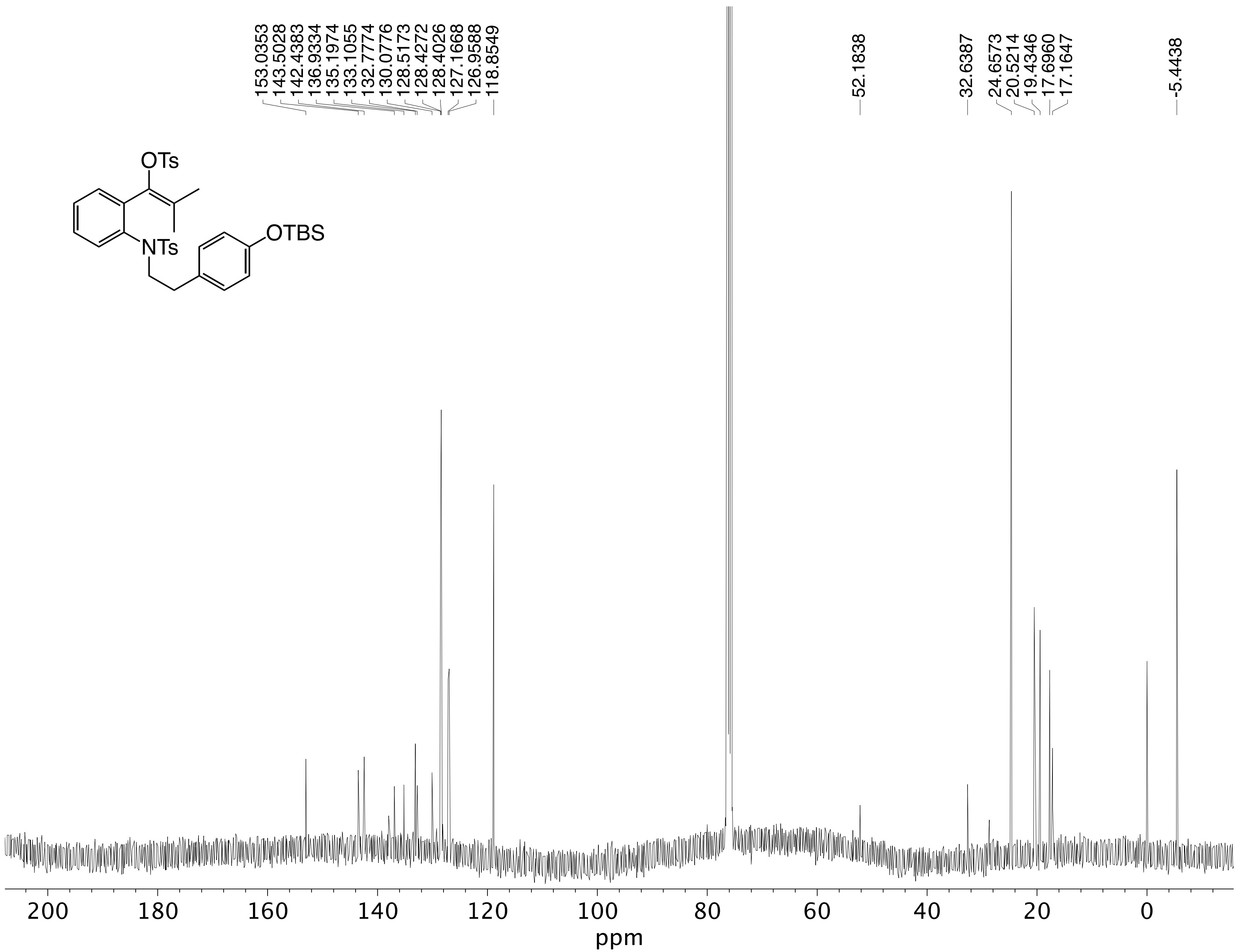

^{13}C NMR (101 MHz, $CDCl_3$) of compound **SI-33**.

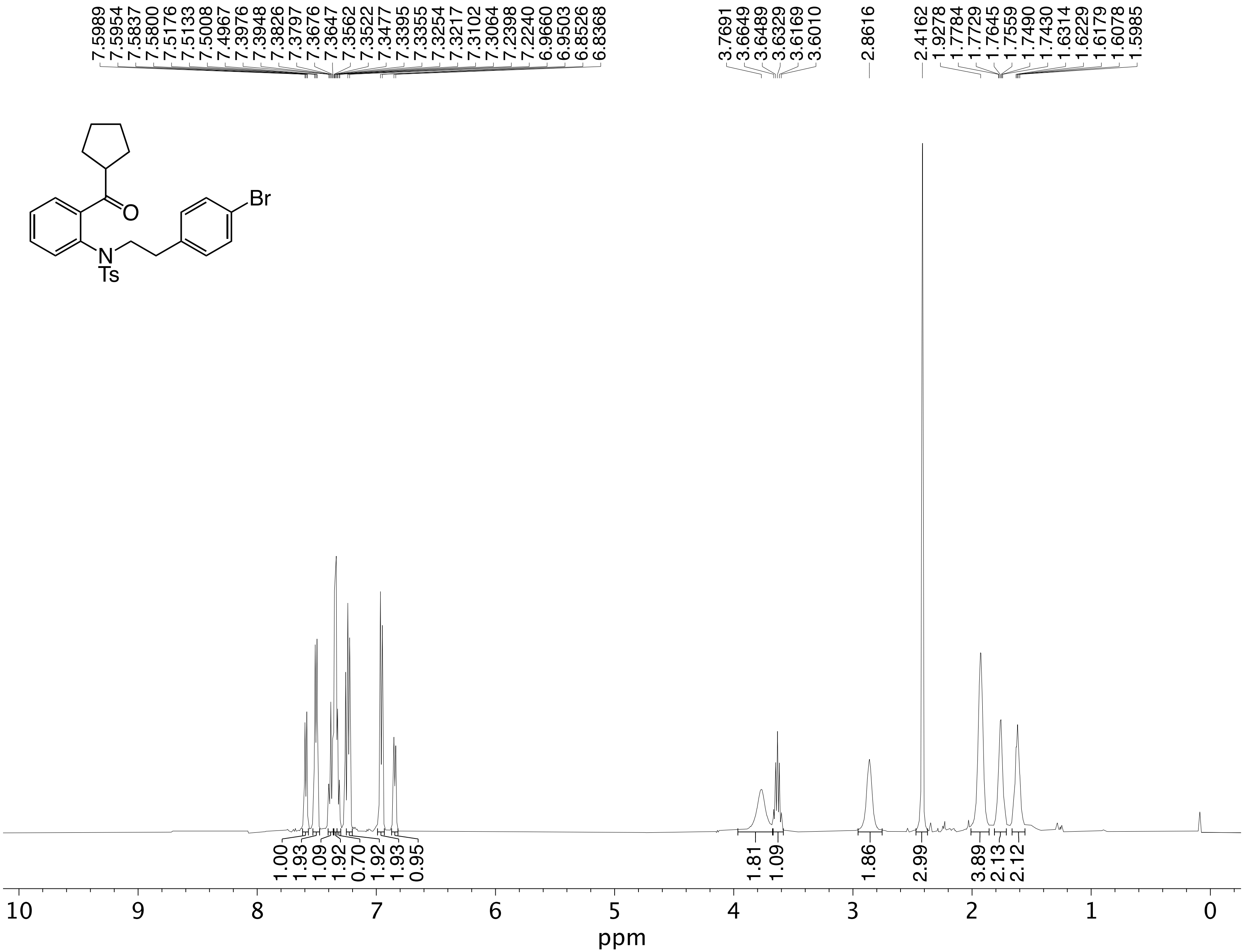

^{1}H NMR (500 MHz, $CDCl_3$, 70°C) of compound **SI-35**.

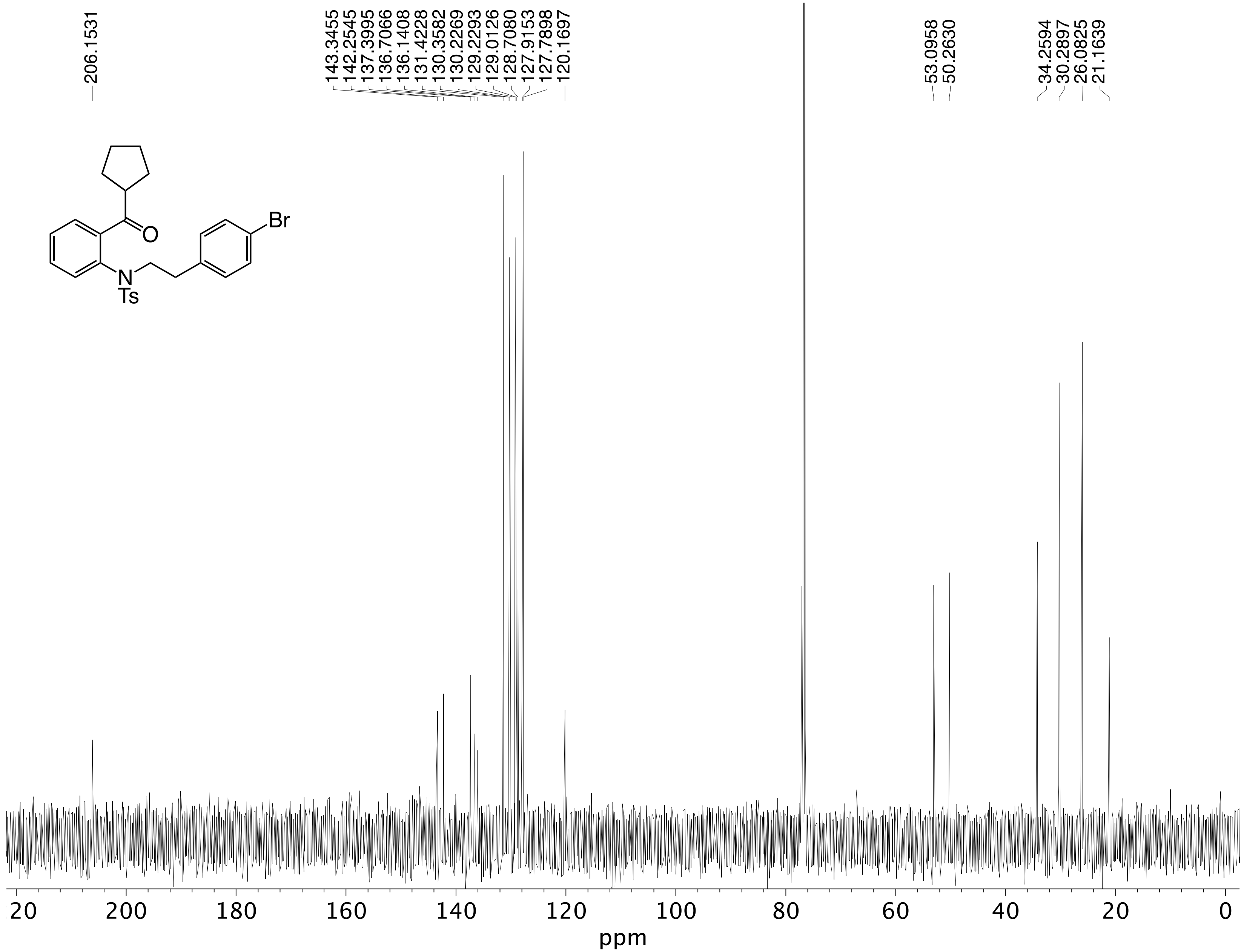

^{13}C NMR (126 MHz, $CDCl_3$, 70°C) of compound **SI-35**.

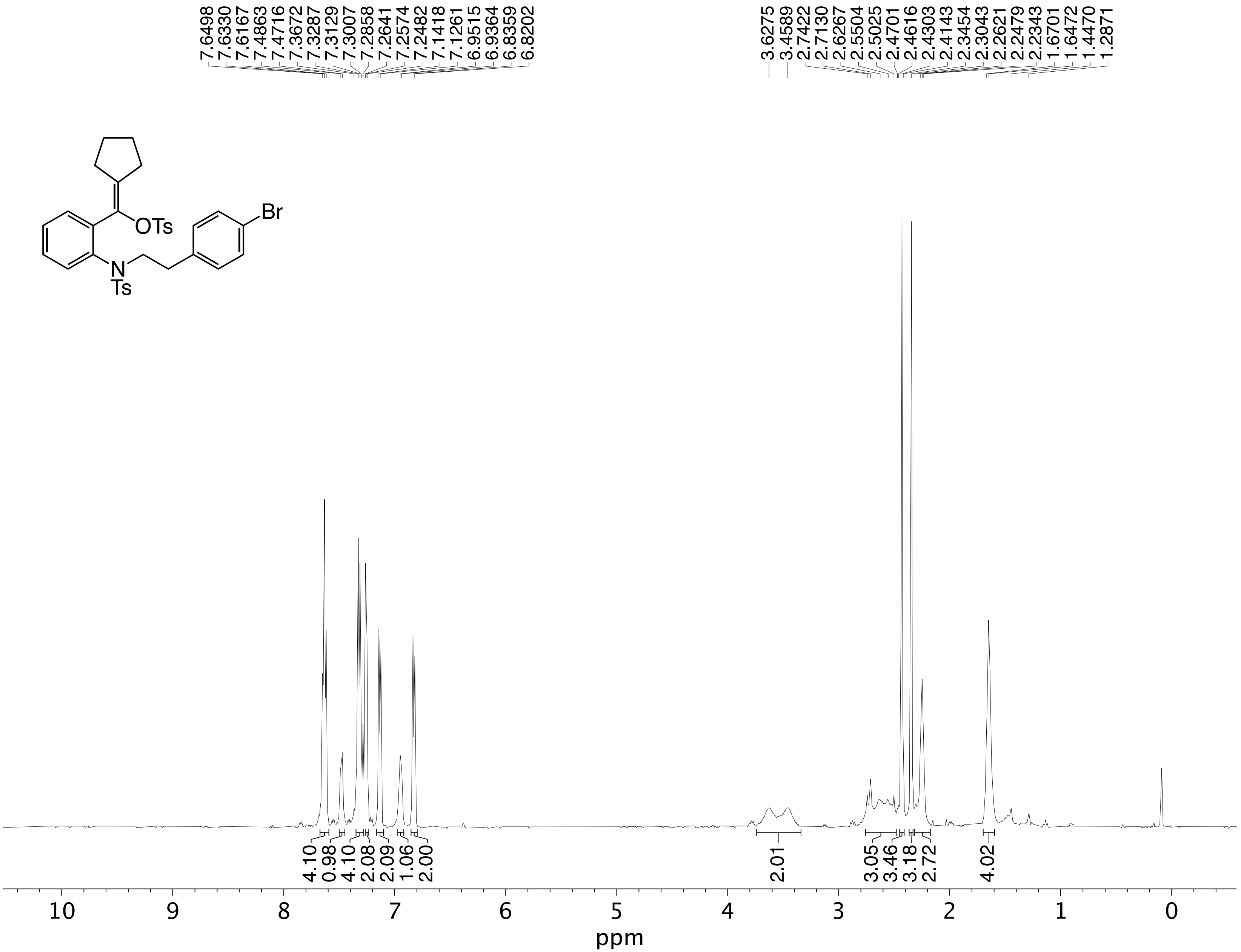

^{1}H NMR (500 MHz, $CDCl_3$, 70°C) of compound **SI-36**.

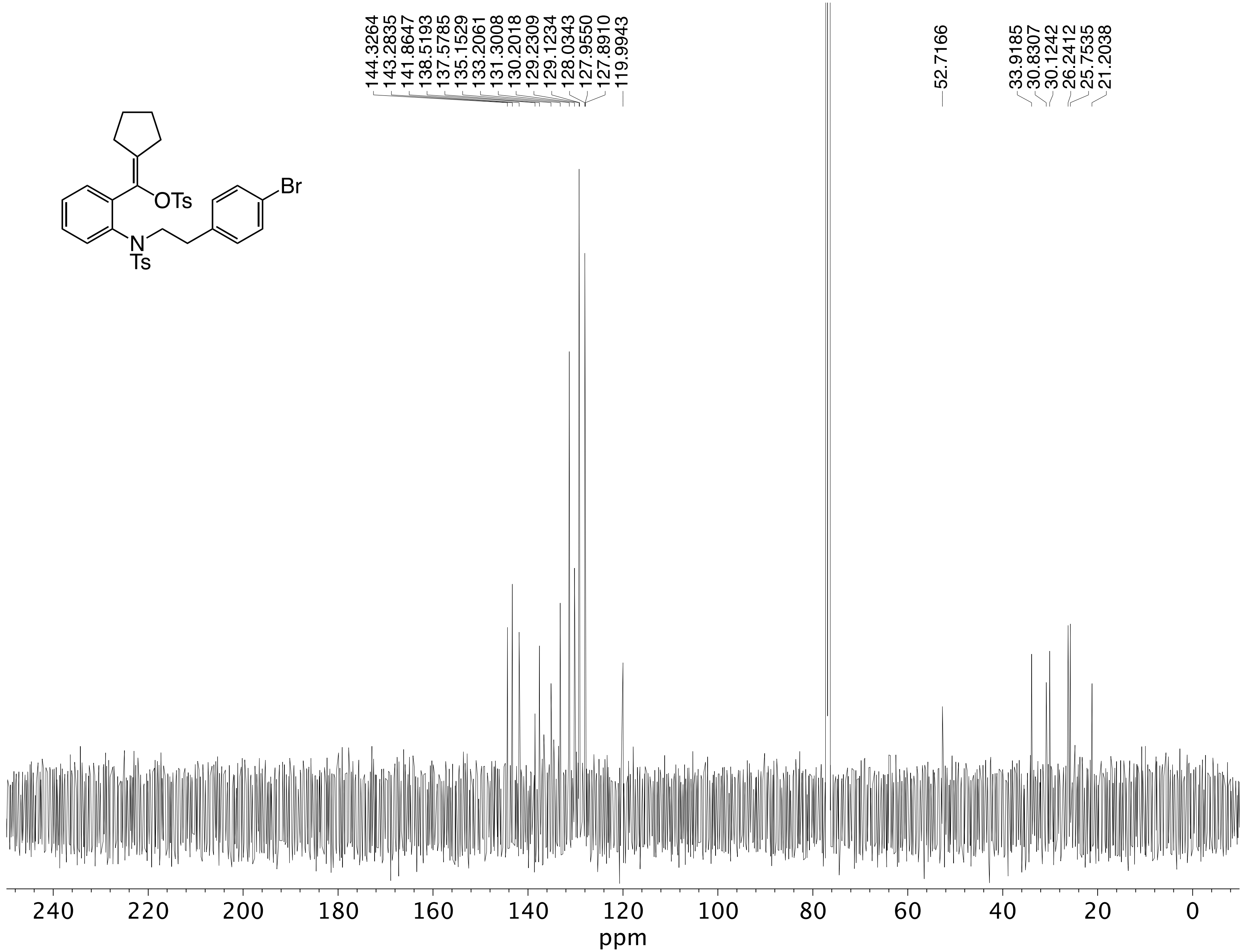

^{13}C NMR (126 MHz, $CDCl_3$, 70°C) of compound **SI-36**.

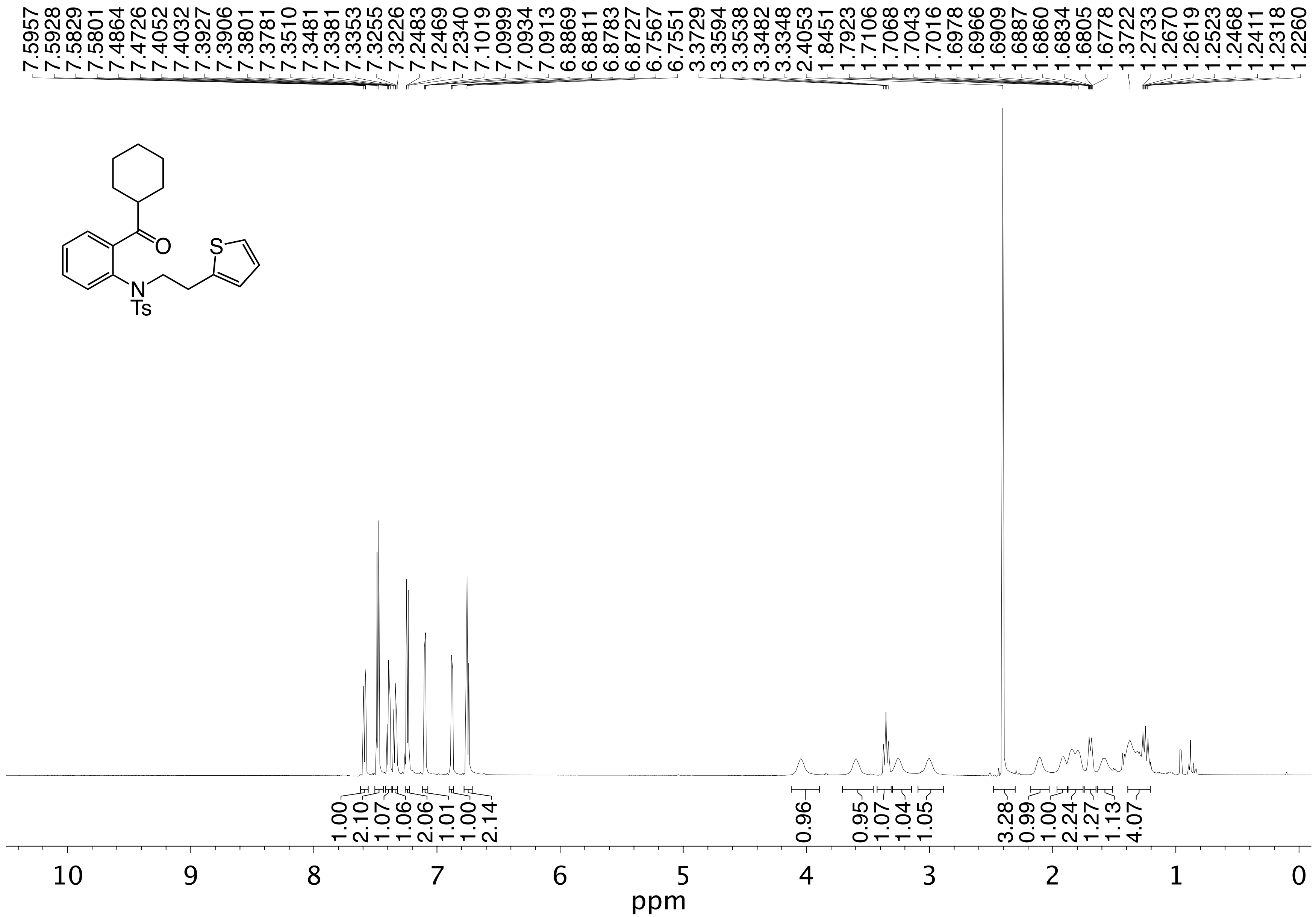

^{1}H NMR (500 MHz, $CDCl_3$) of compound **SI-37**.

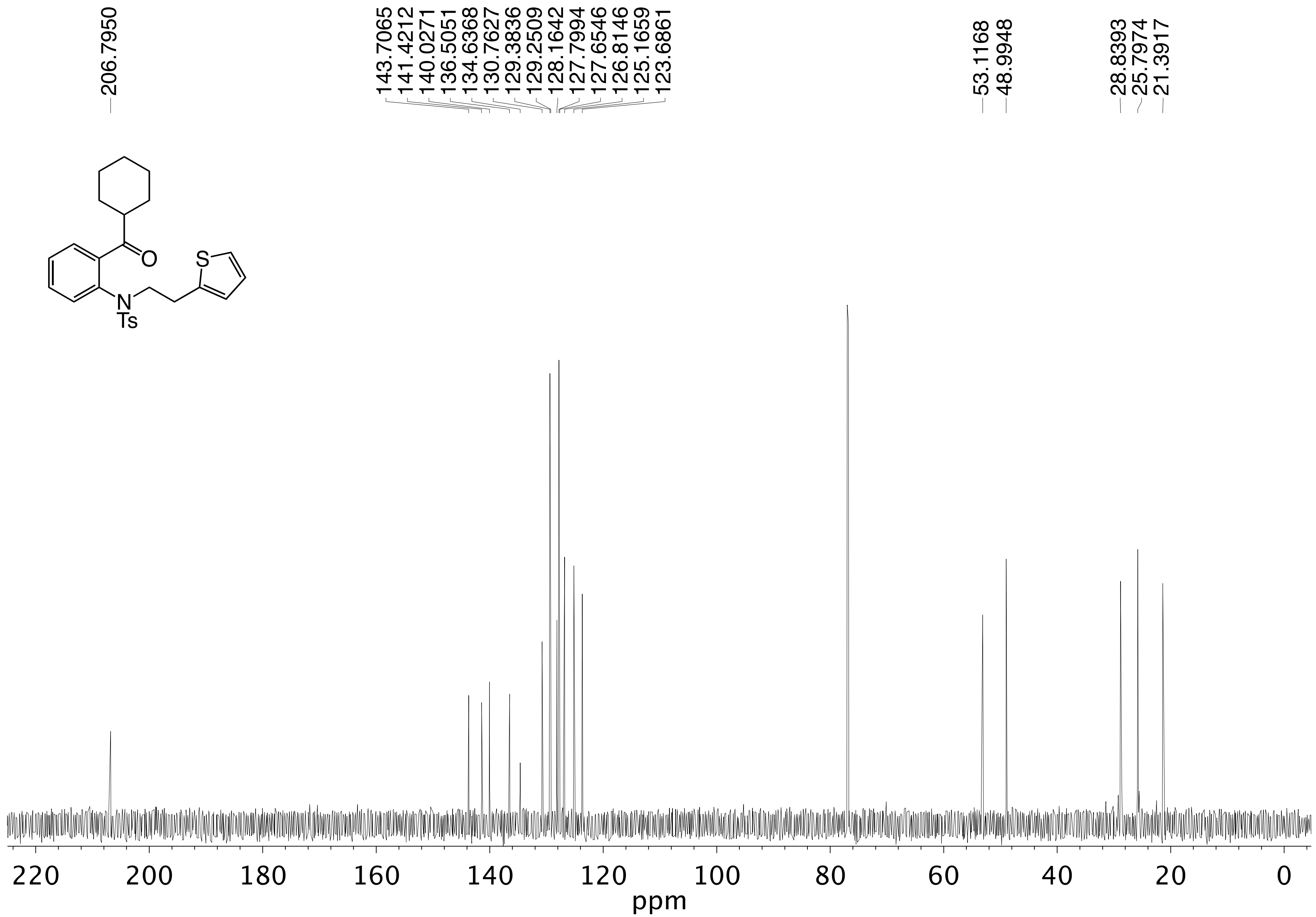

^{13}C NMR (126 MHz, $CDCl_3$) of compound **SI-37**.

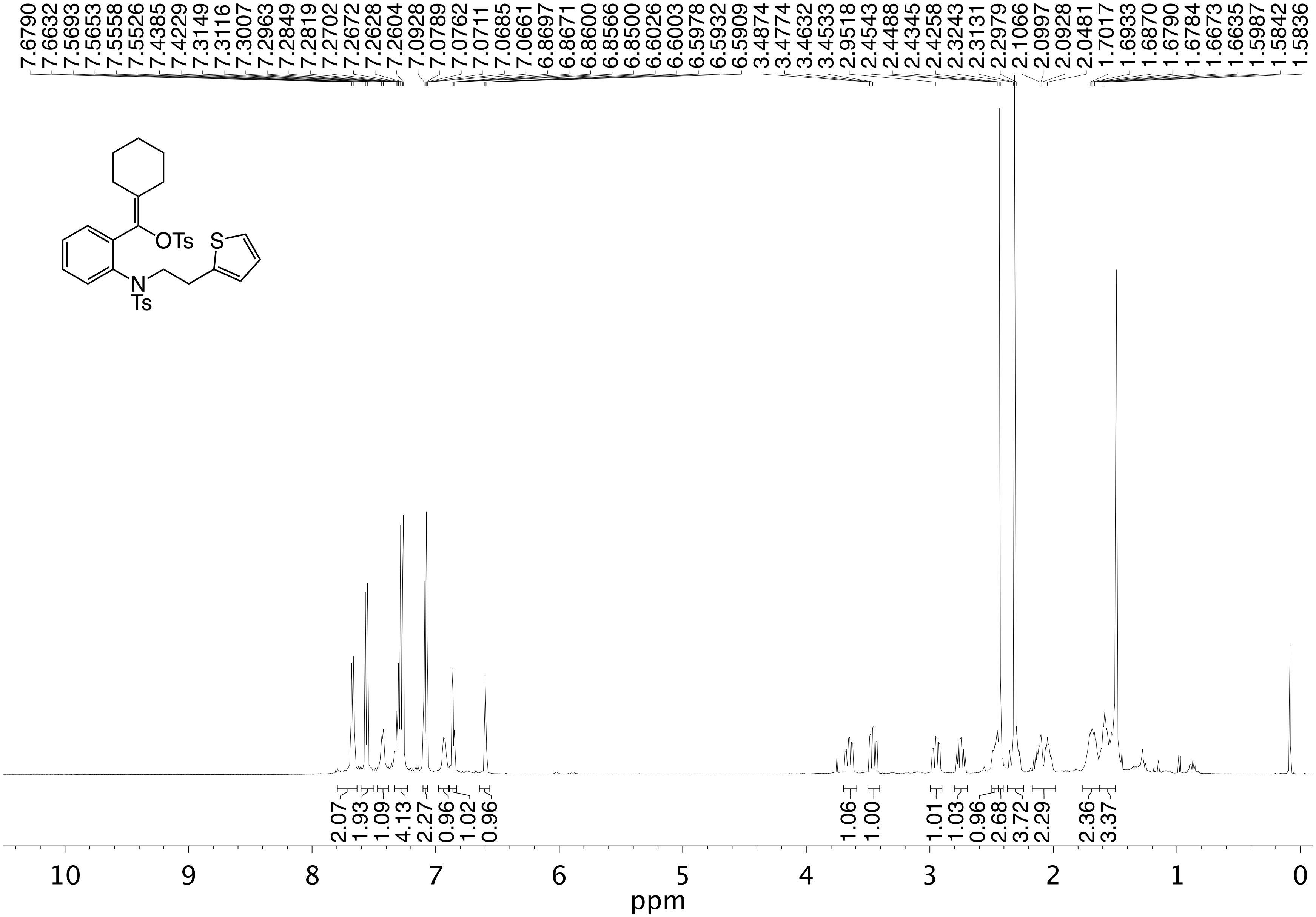

^{1}H NMR (500 MHz, $CDCl_3$, 55°C) of compound **SI-38**.

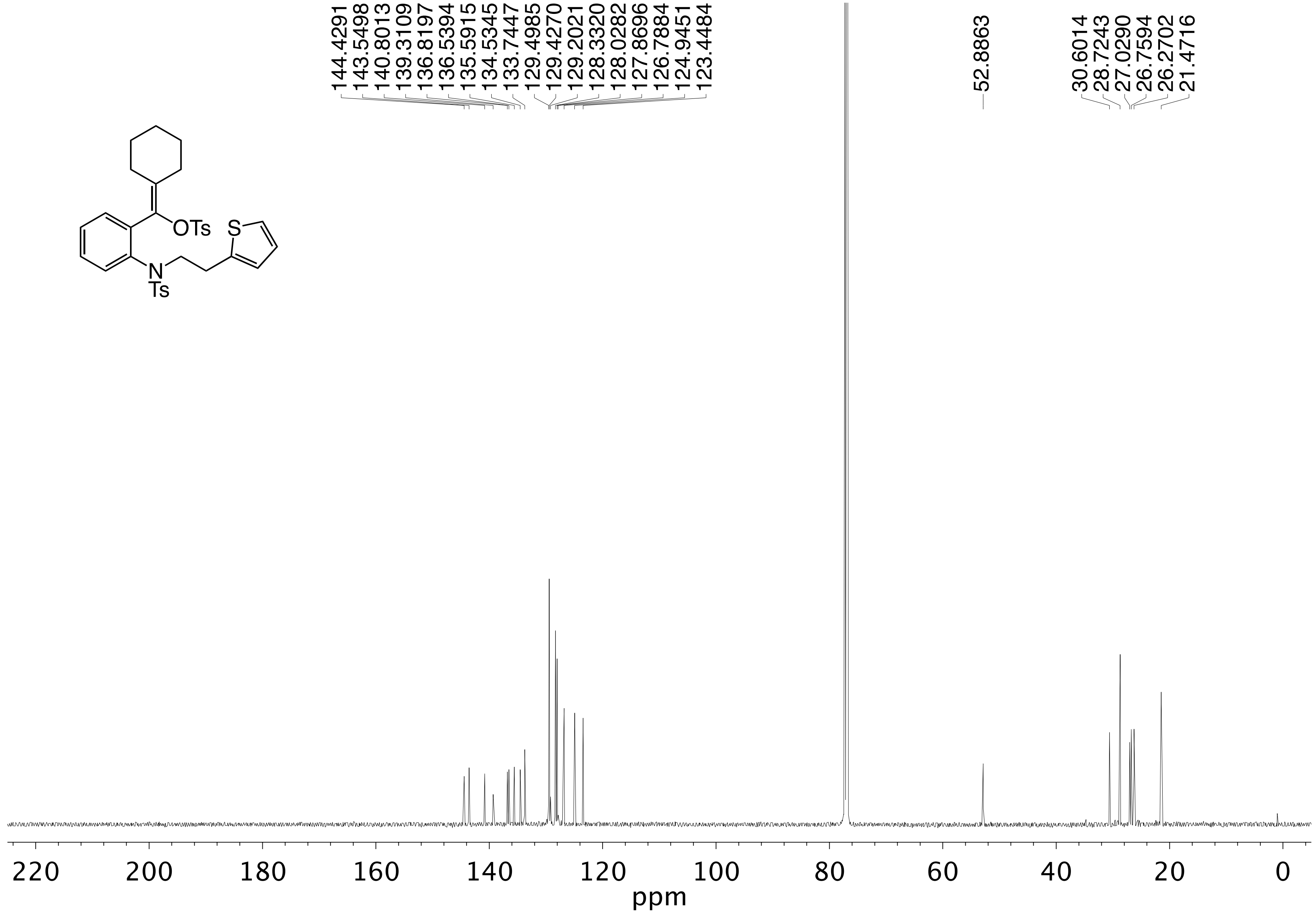

^{13}C NMR (126 MHz, $CDCl_3$, 55°C) of compound **SI-38**.

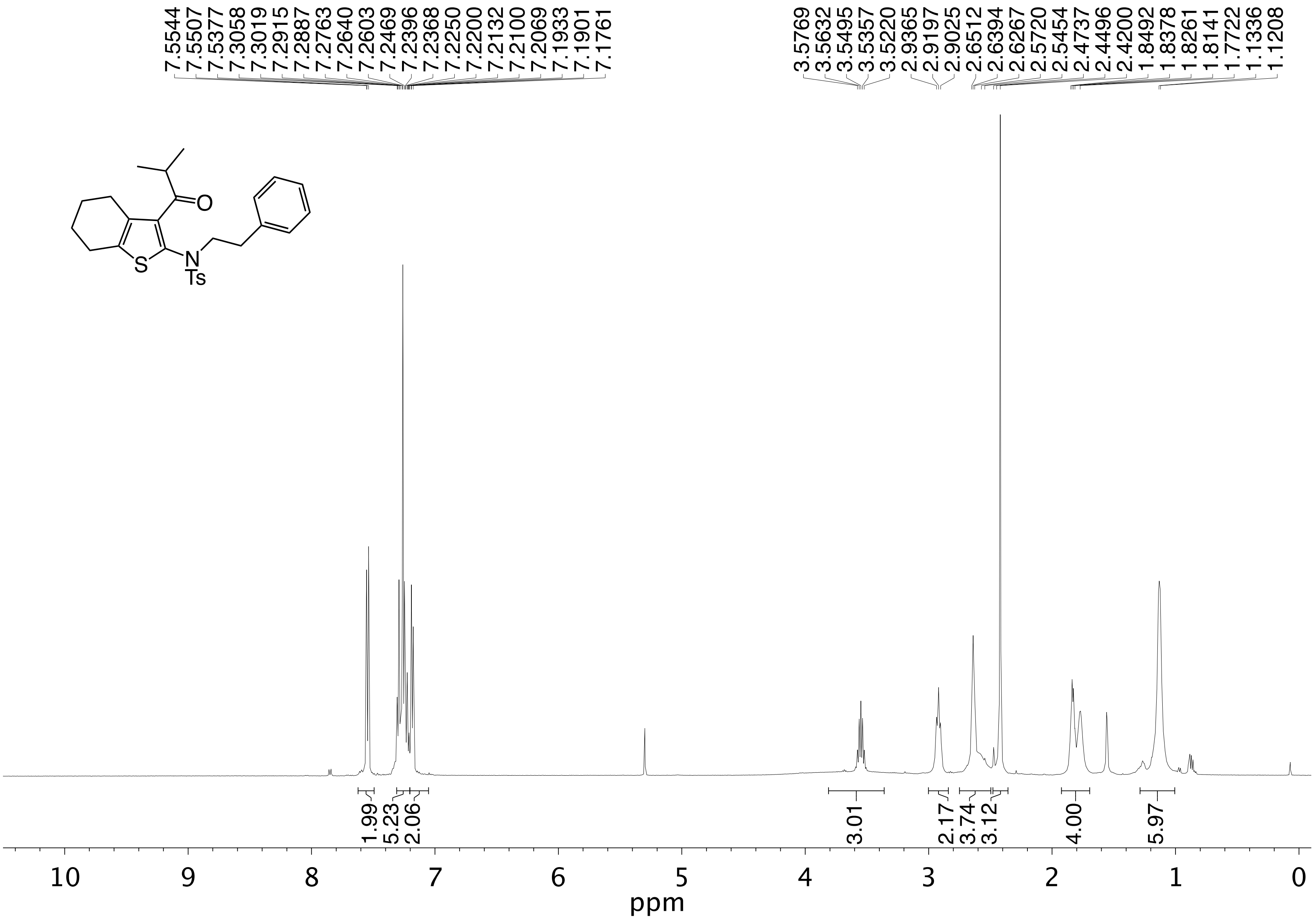

^{1}H NMR (500 MHz, $CDCl_3$, 70°C) of compound **SI-41.**

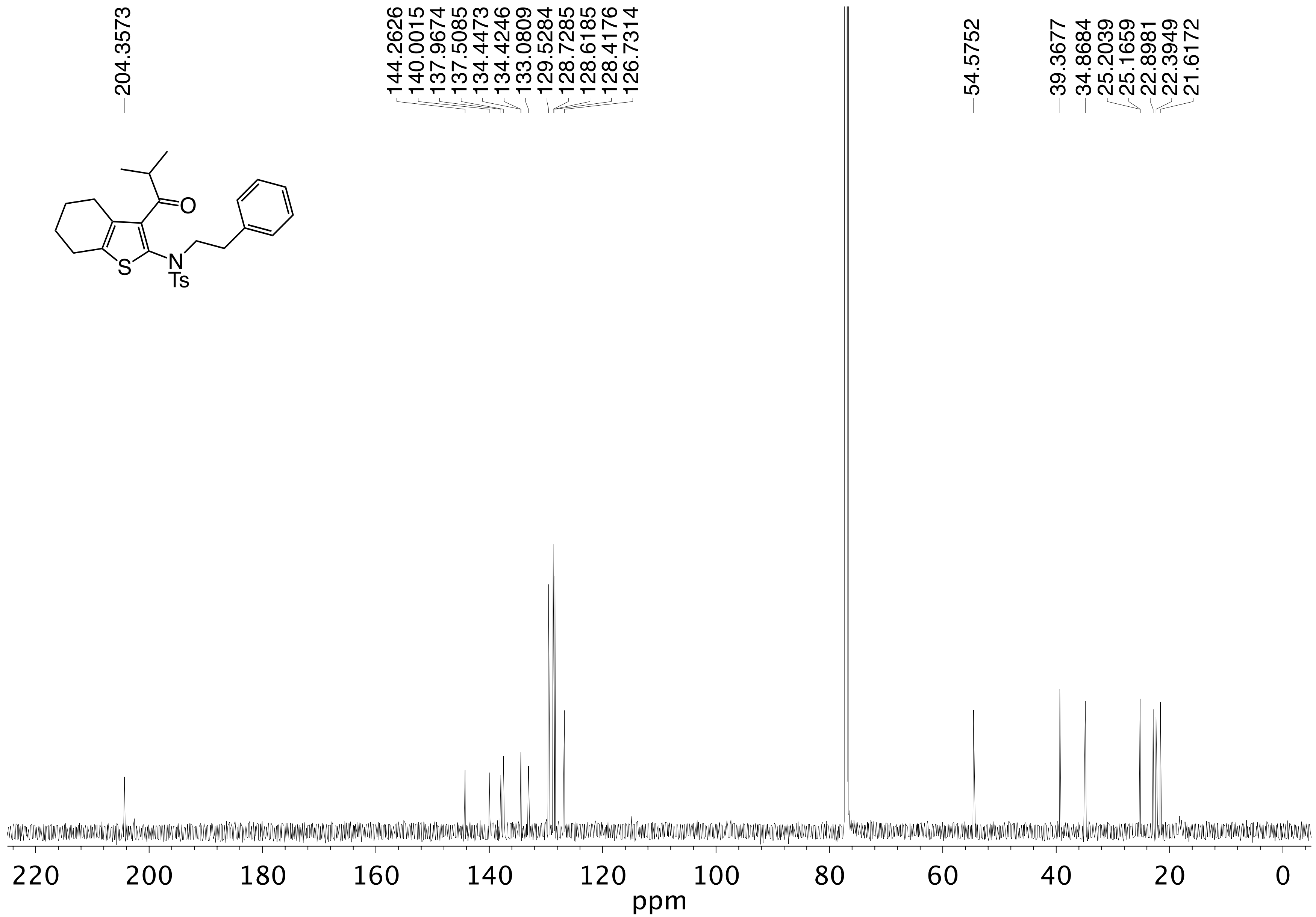

^{13}C NMR (126 MHz, $CDCl_3$, 70°C) of compound **SI-41.**

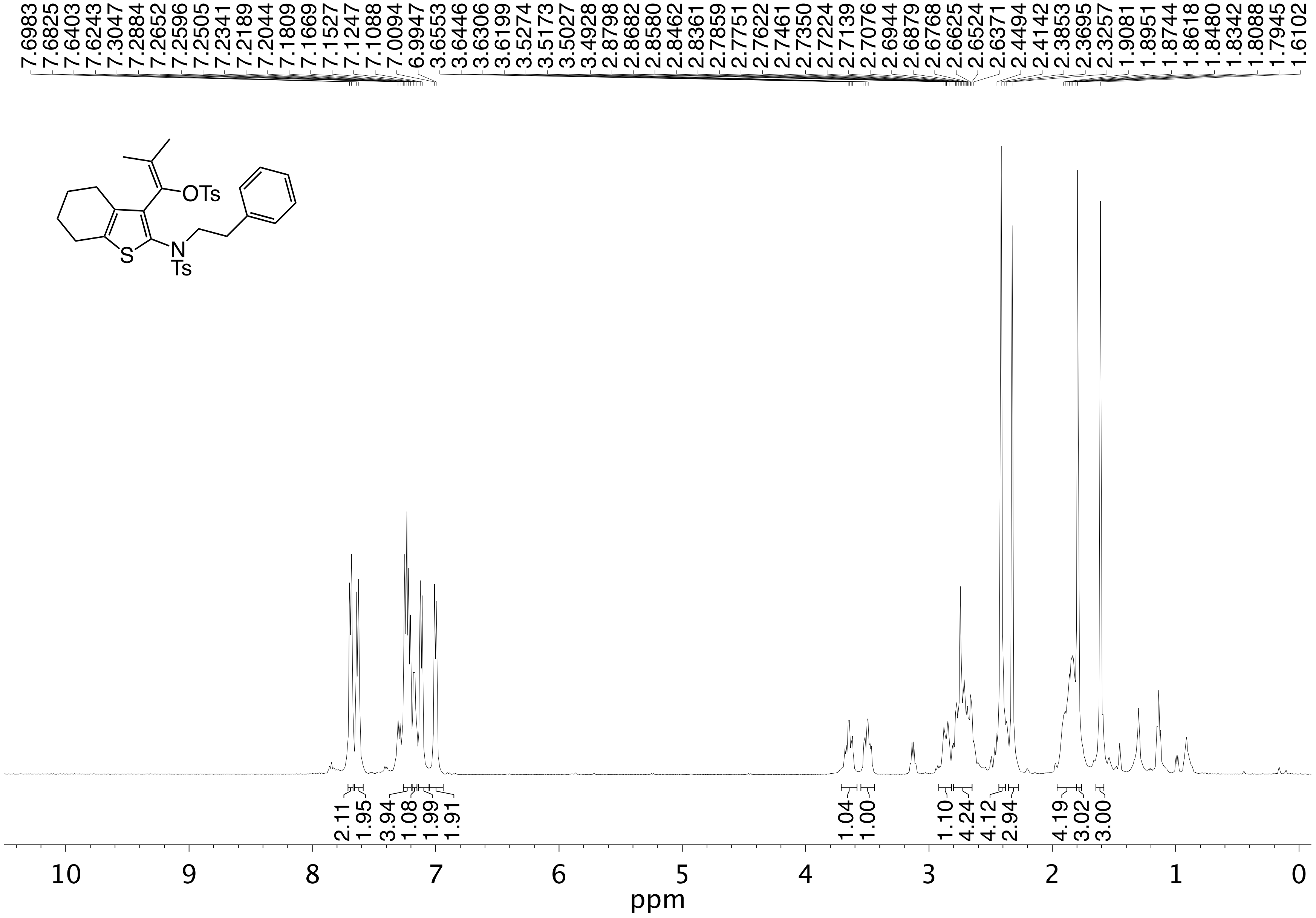

^{1}H NMR (500 MHz, $CDCl_3$, 70°C) of compound **SI-42.**

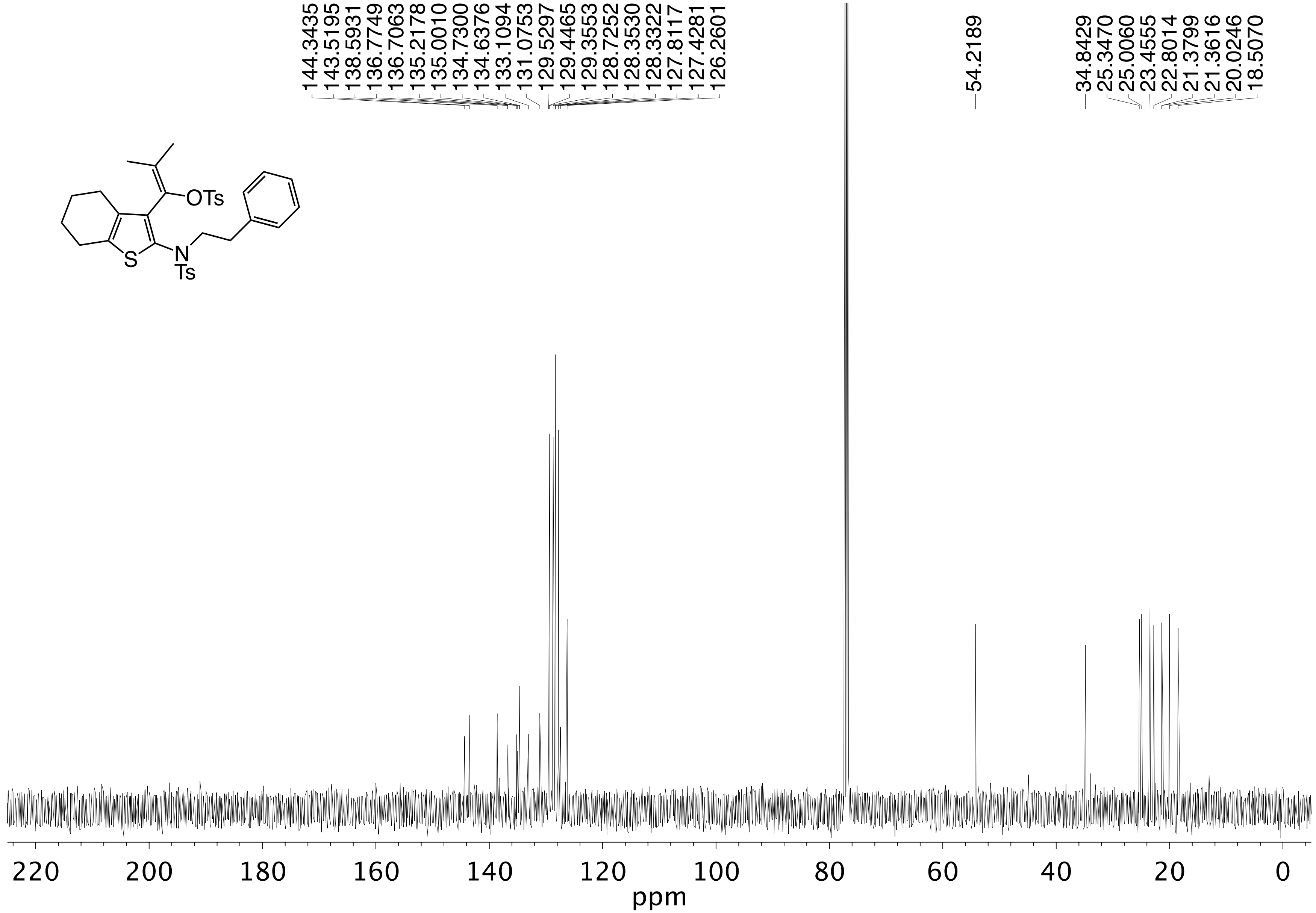

^{13}C NMR (126 MHz, $CDCl_3$, 70°C) of compound **SI-42.**

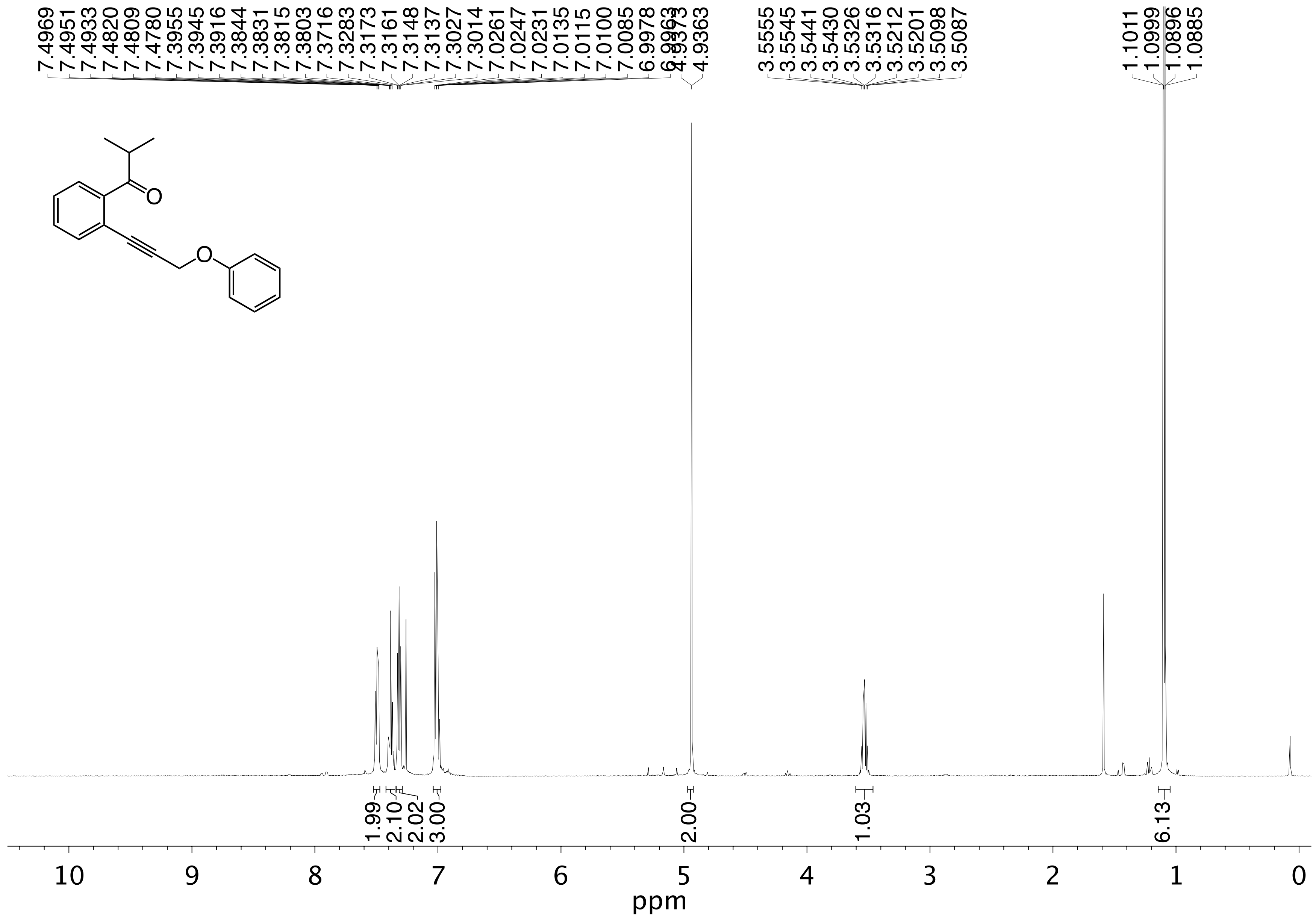

^{1}H NMR (600 MHz, $CDCl_3$) of compound **SI-43.**

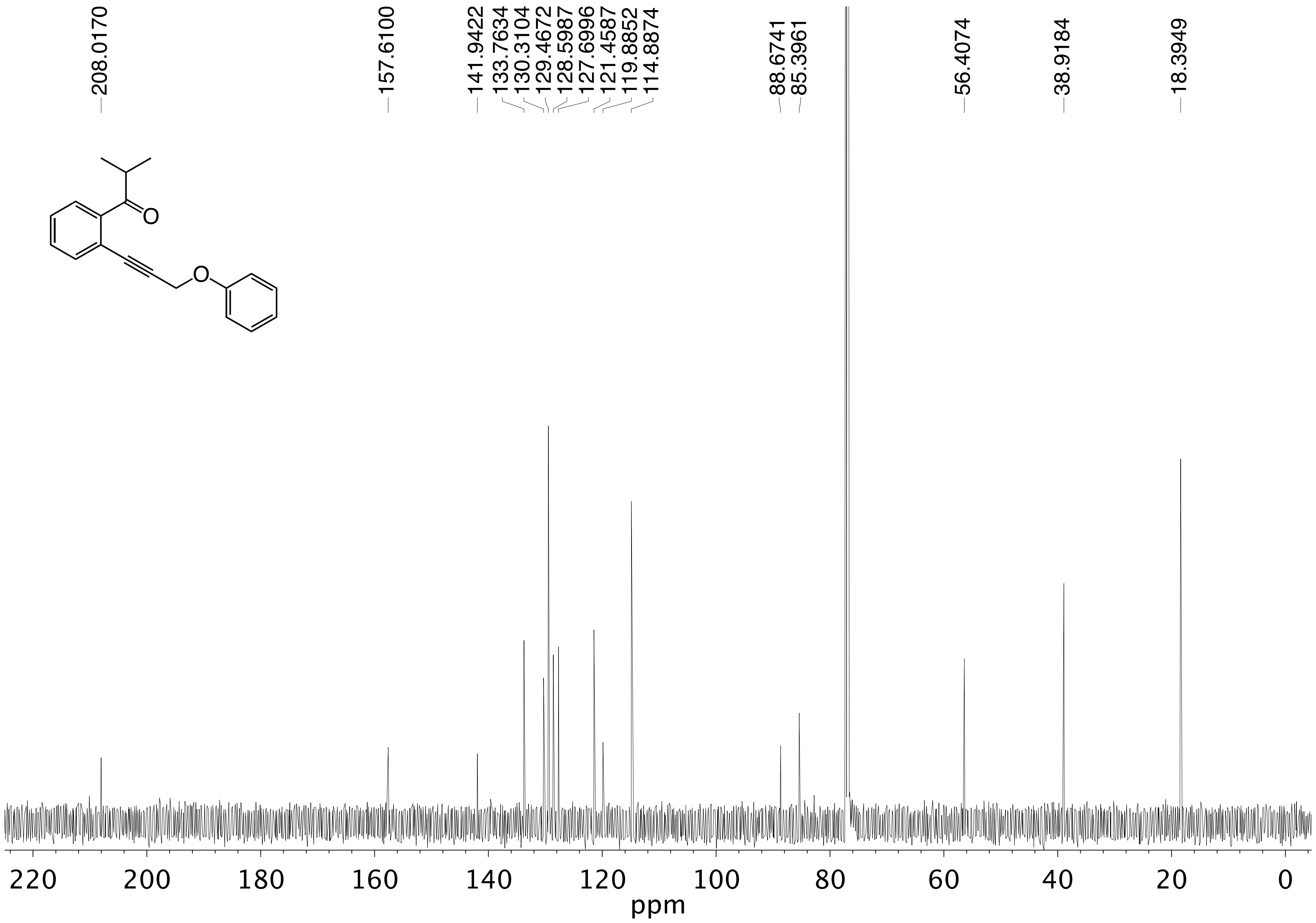

^{13}C NMR (151 MHz, $CDCl_3$) of compound **SI-43.**

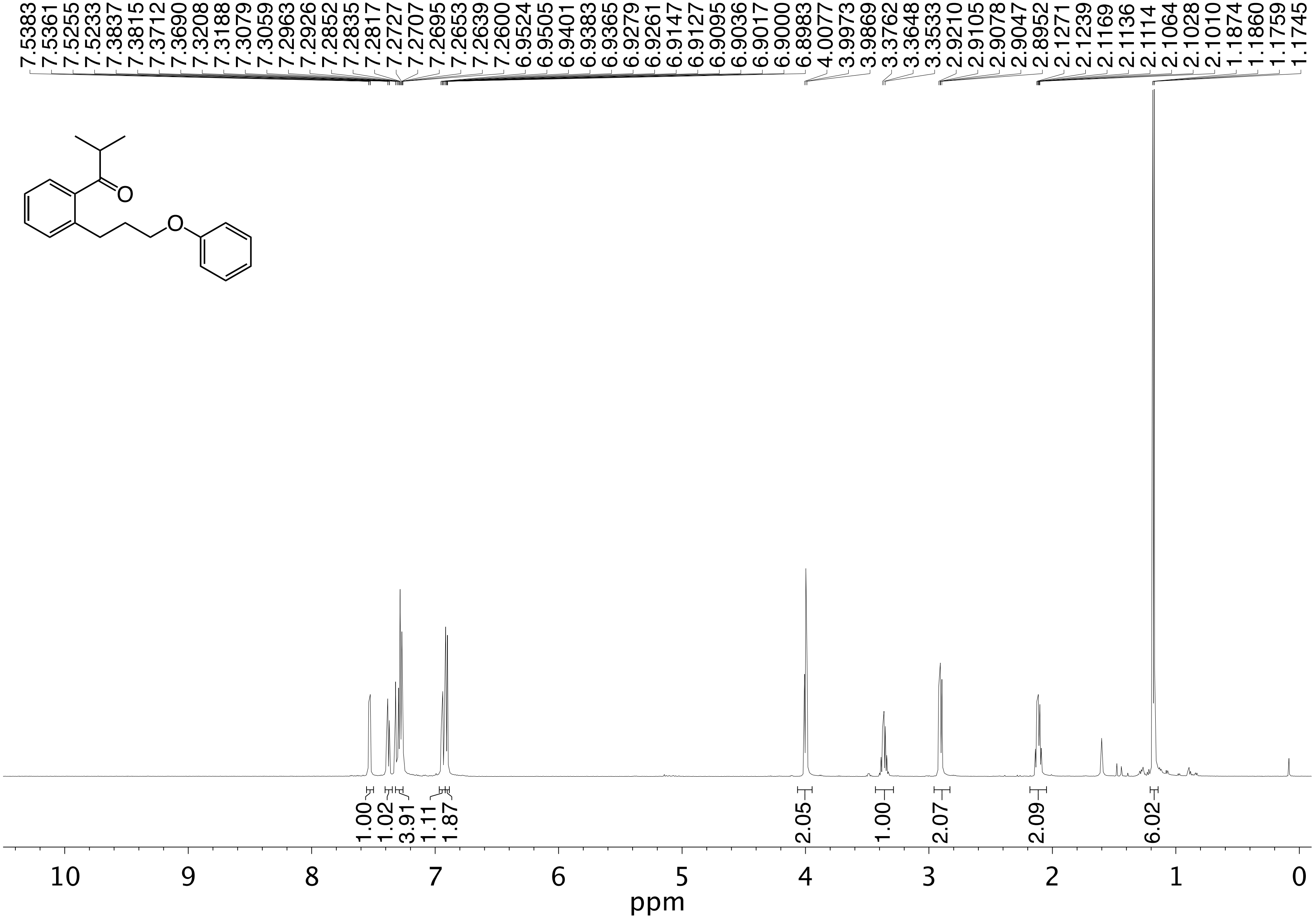

^{1}H NMR (600 MHz, $CDCl_3$) of compound **SI-44.**

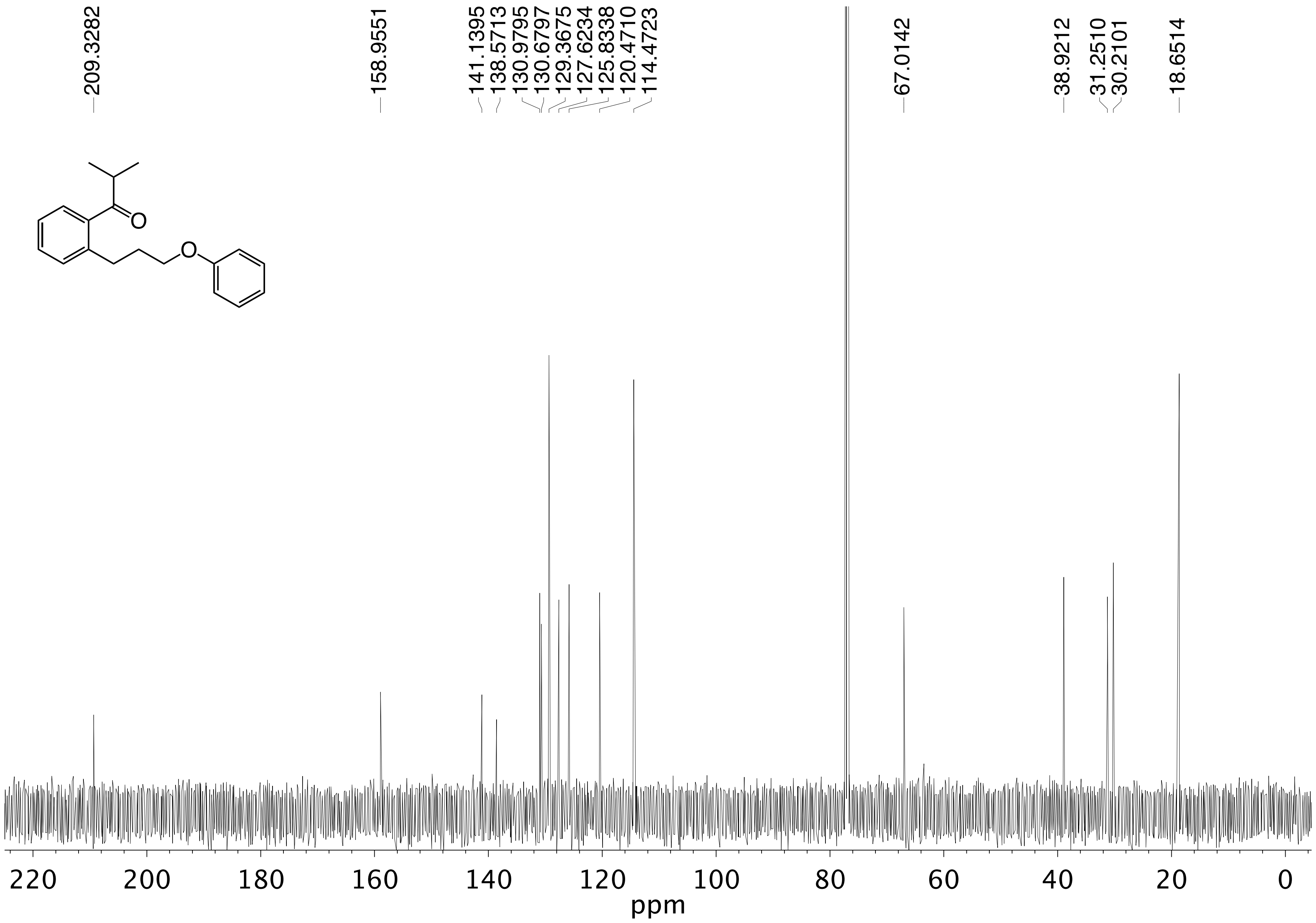

^{13}C NMR (151 MHz, $CDCl_3$) of compound **SI-44.**

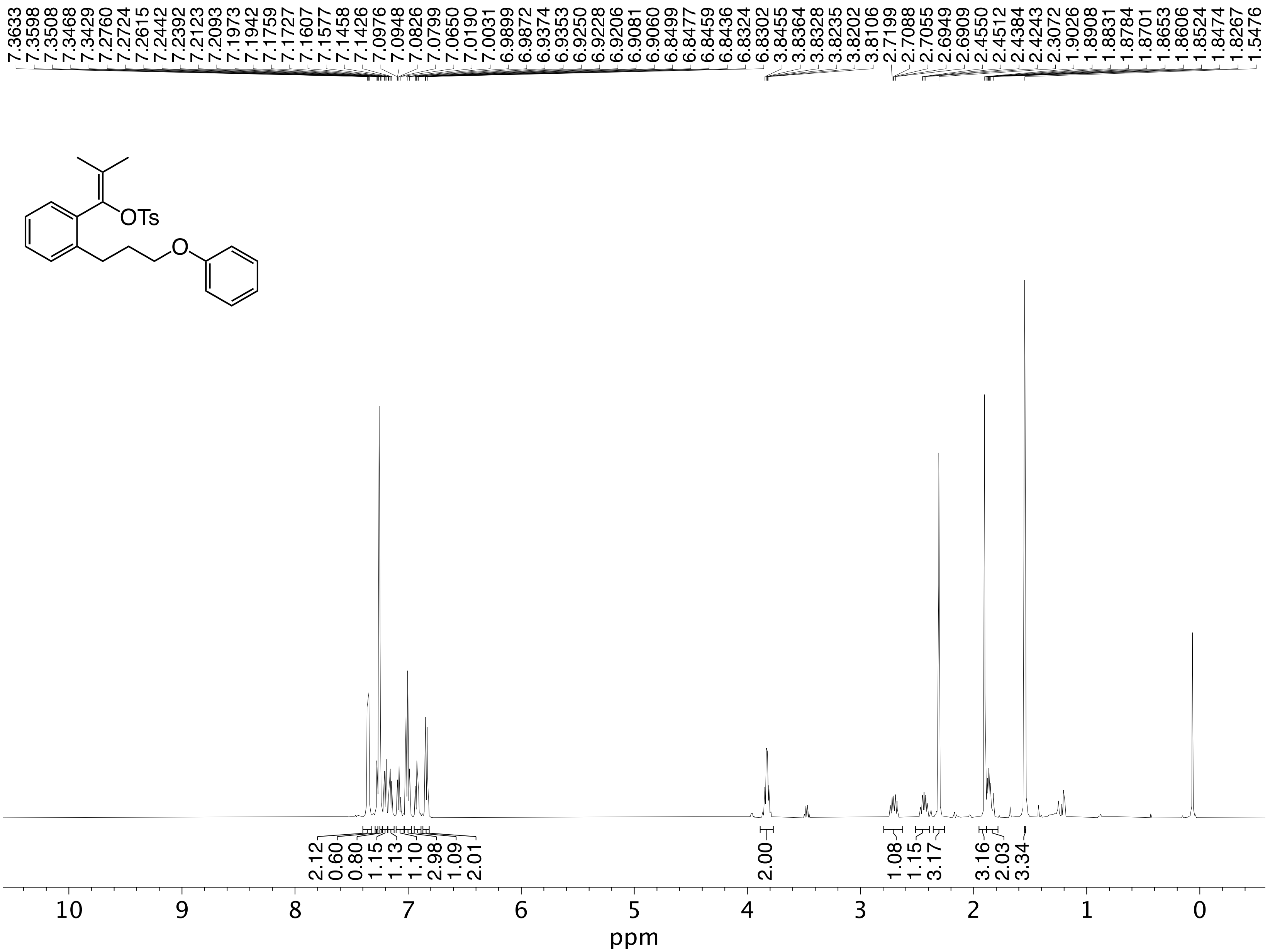

^{1}H NMR (500 MHz, $CDCl_3$) of compound **SI-45.**

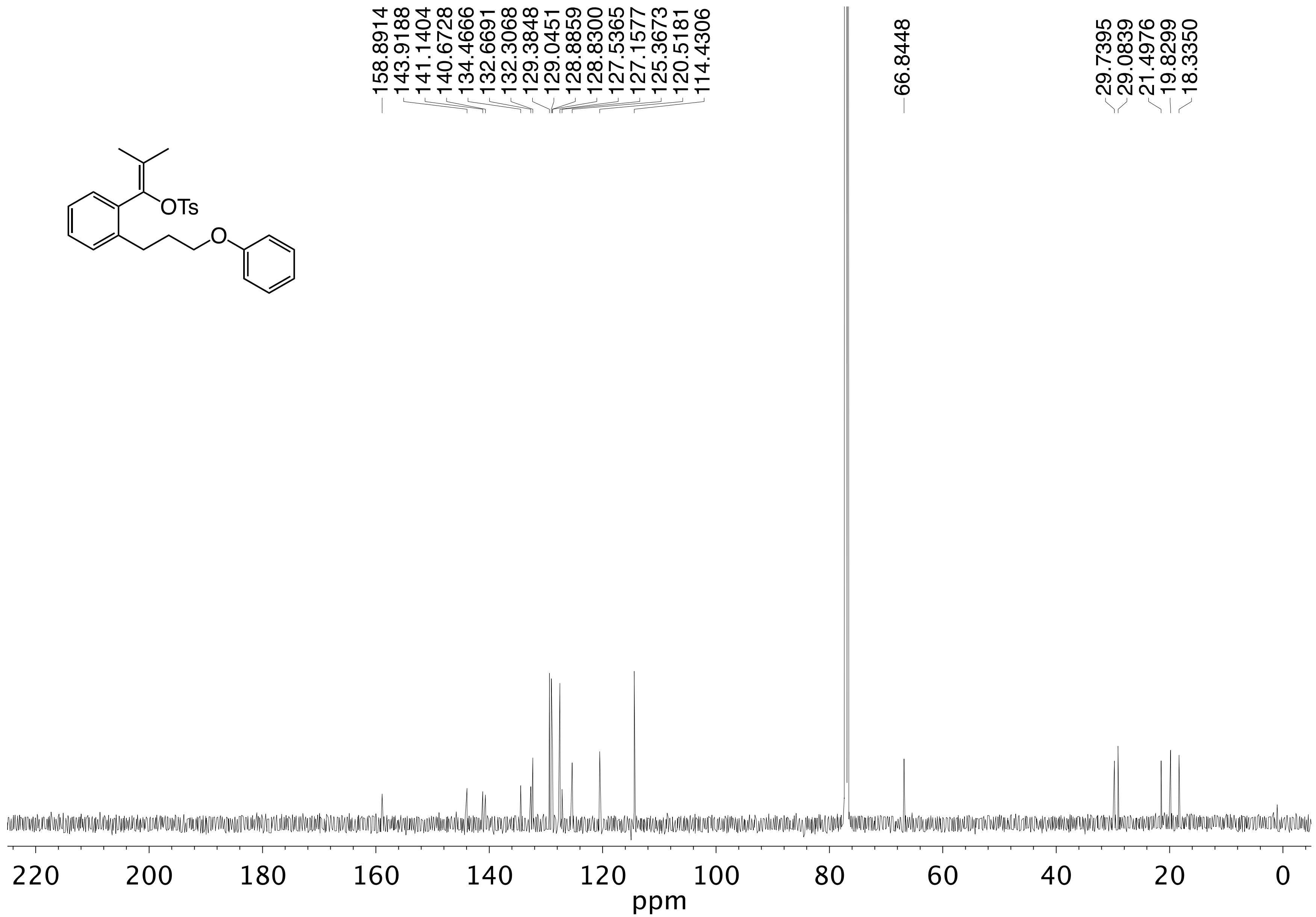

^{13}C NMR (126 MHz, $CDCl_3$) of compound **SI-45.**

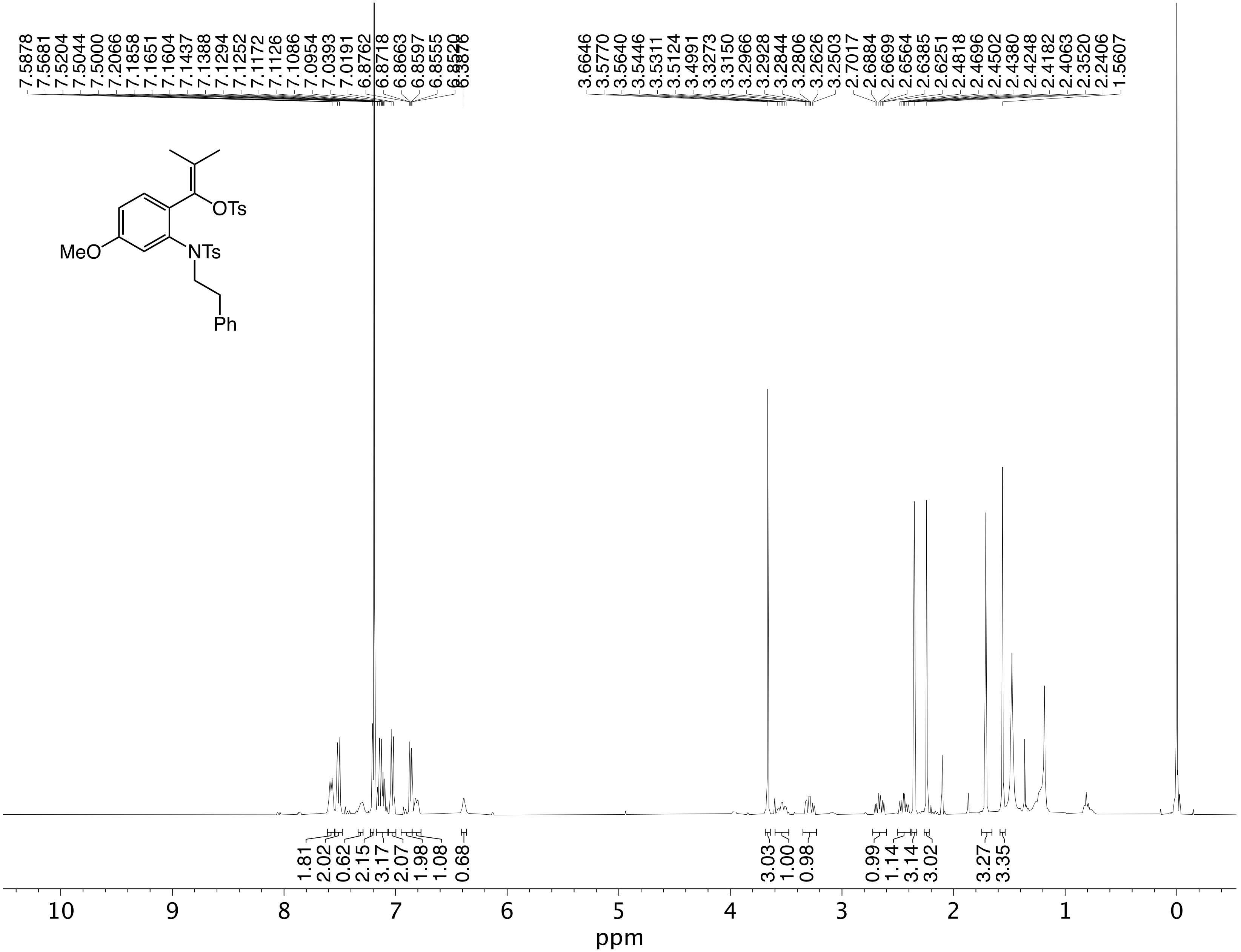

^{1}H NMR (400 MHz, $CDCl_3$) of compound **SI-46.**

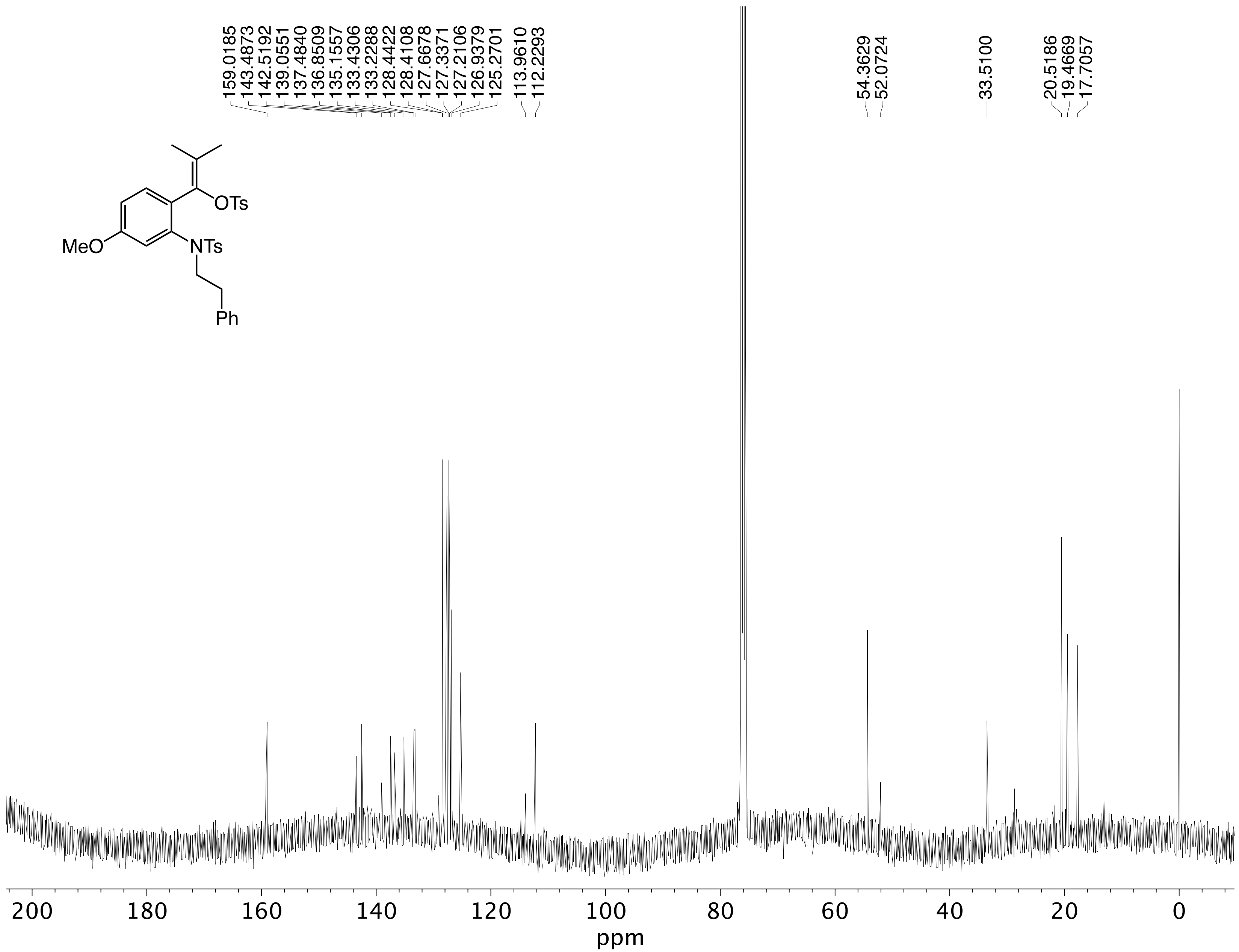

^{13}C NMR (101 MHz, $CDCl_3$) of compound **SI-46.**

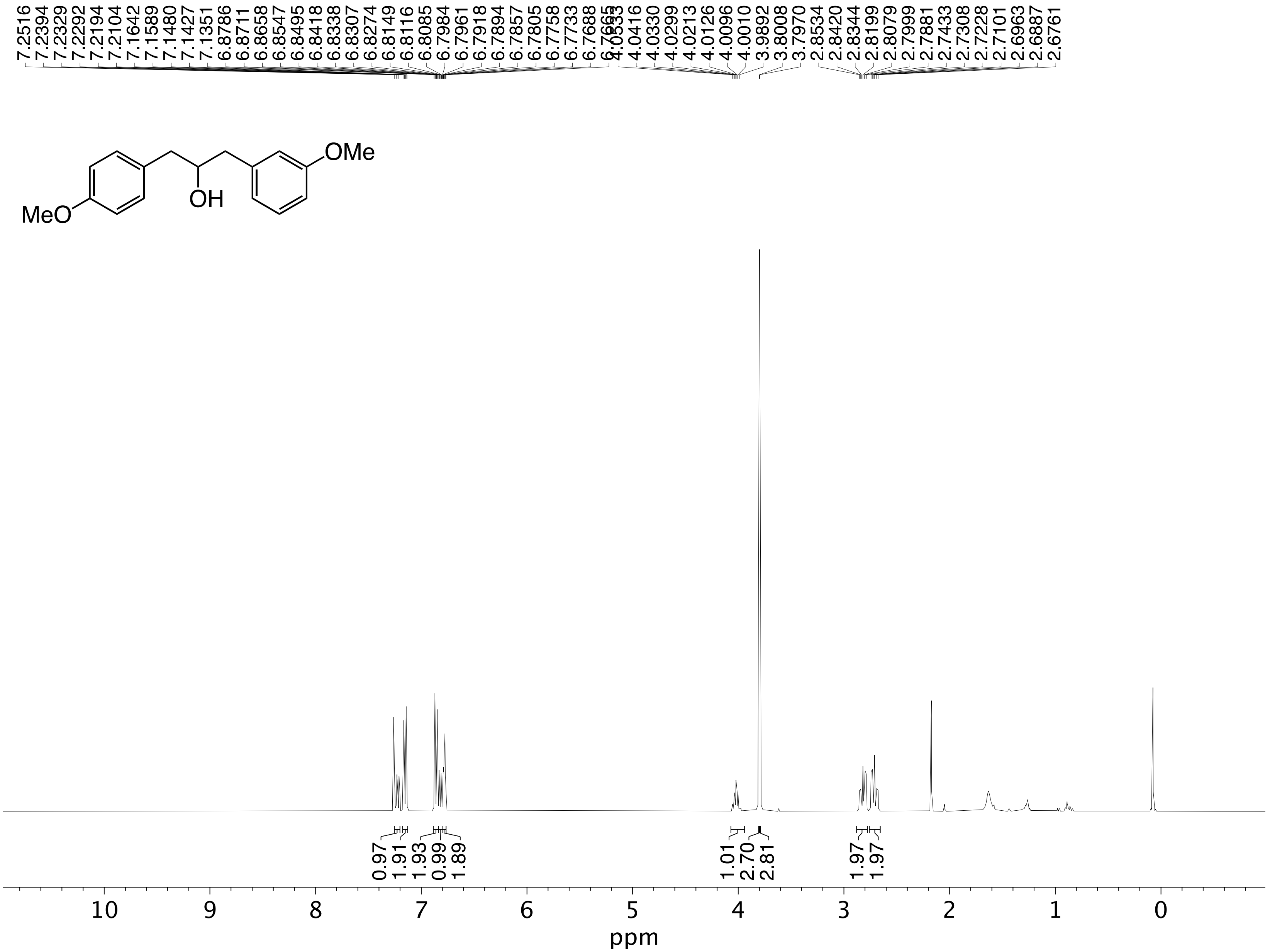

^{1}H NMR (400 MHz, $CDCl_3$) of compound **SI-48.**

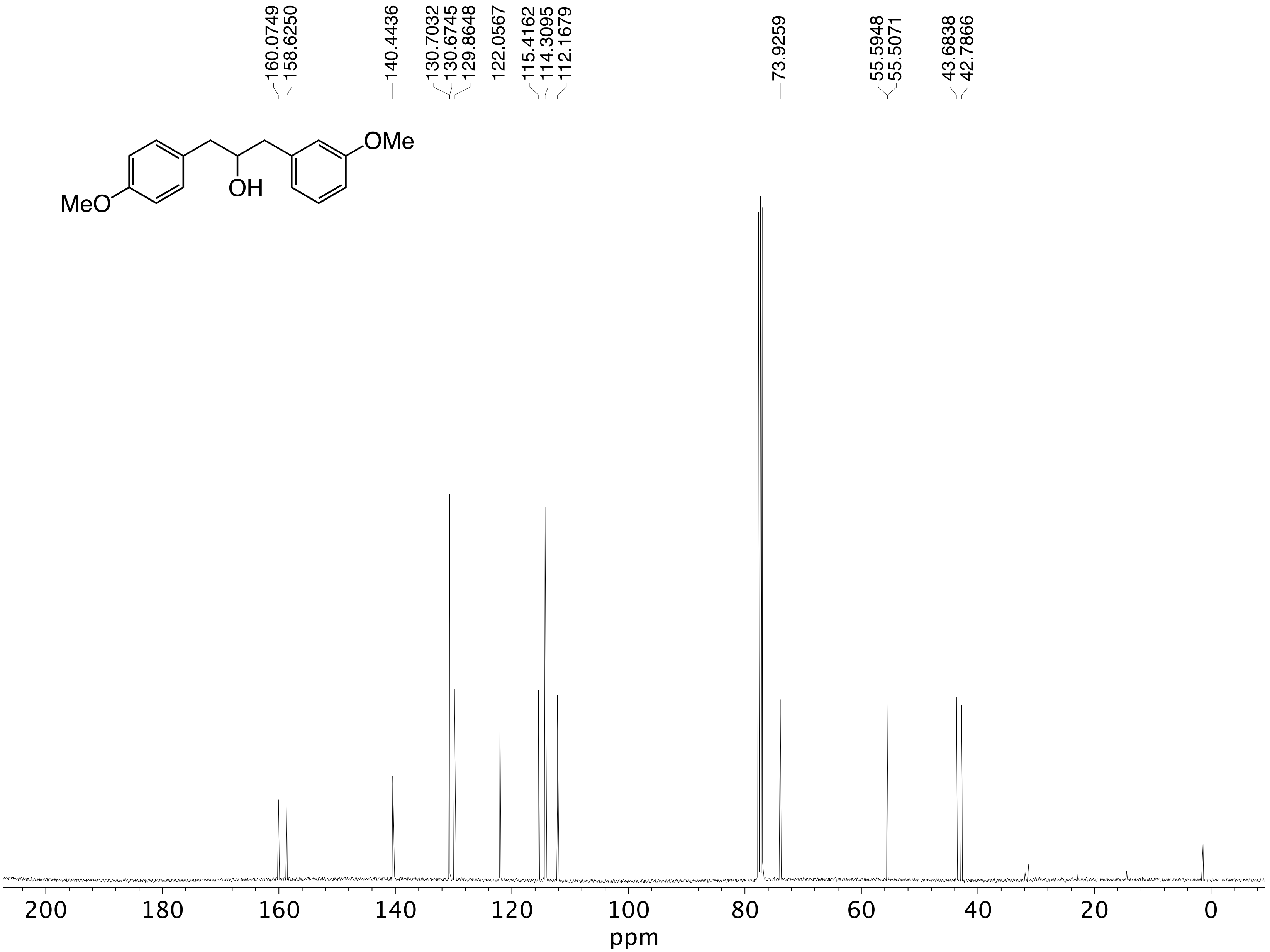

^{13}C NMR (101 MHz, $CDCl_3$) of compound **SI-48.**

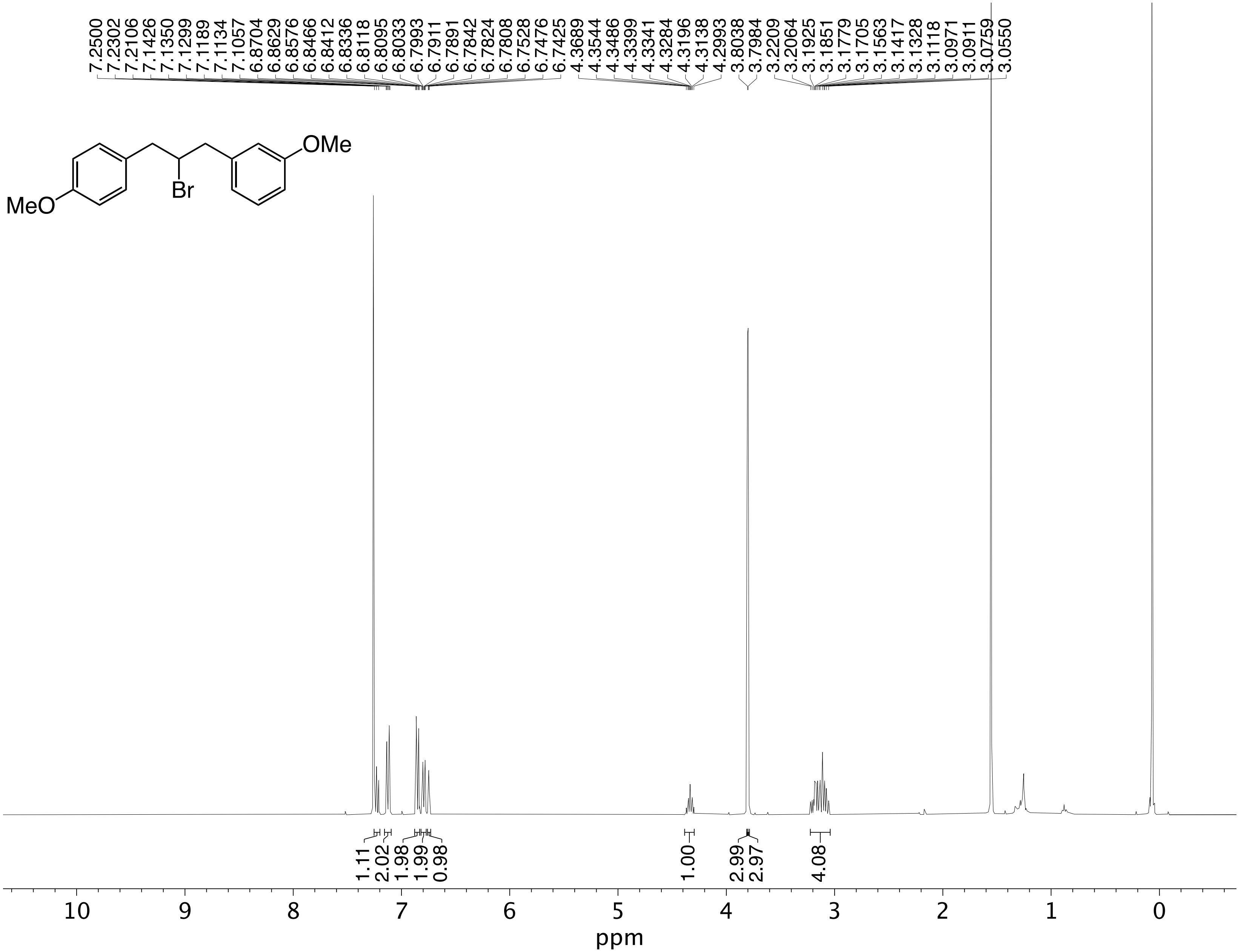

^{1}H NMR (400 MHz, $CDCl_3$) of compound **SI-49.**

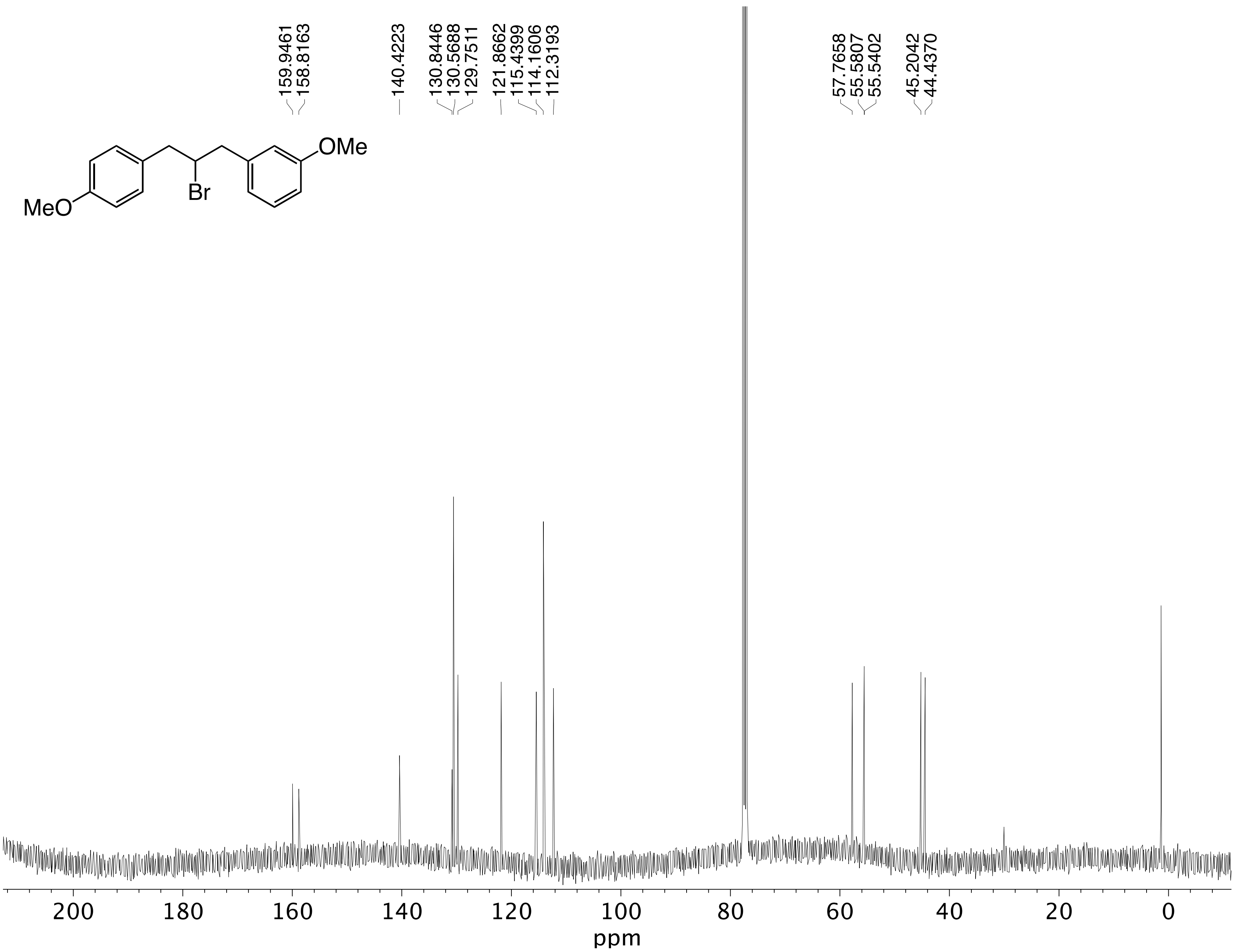

^{13}C NMR (101 MHz, $CDCl_3$) of compound **SI-49.**

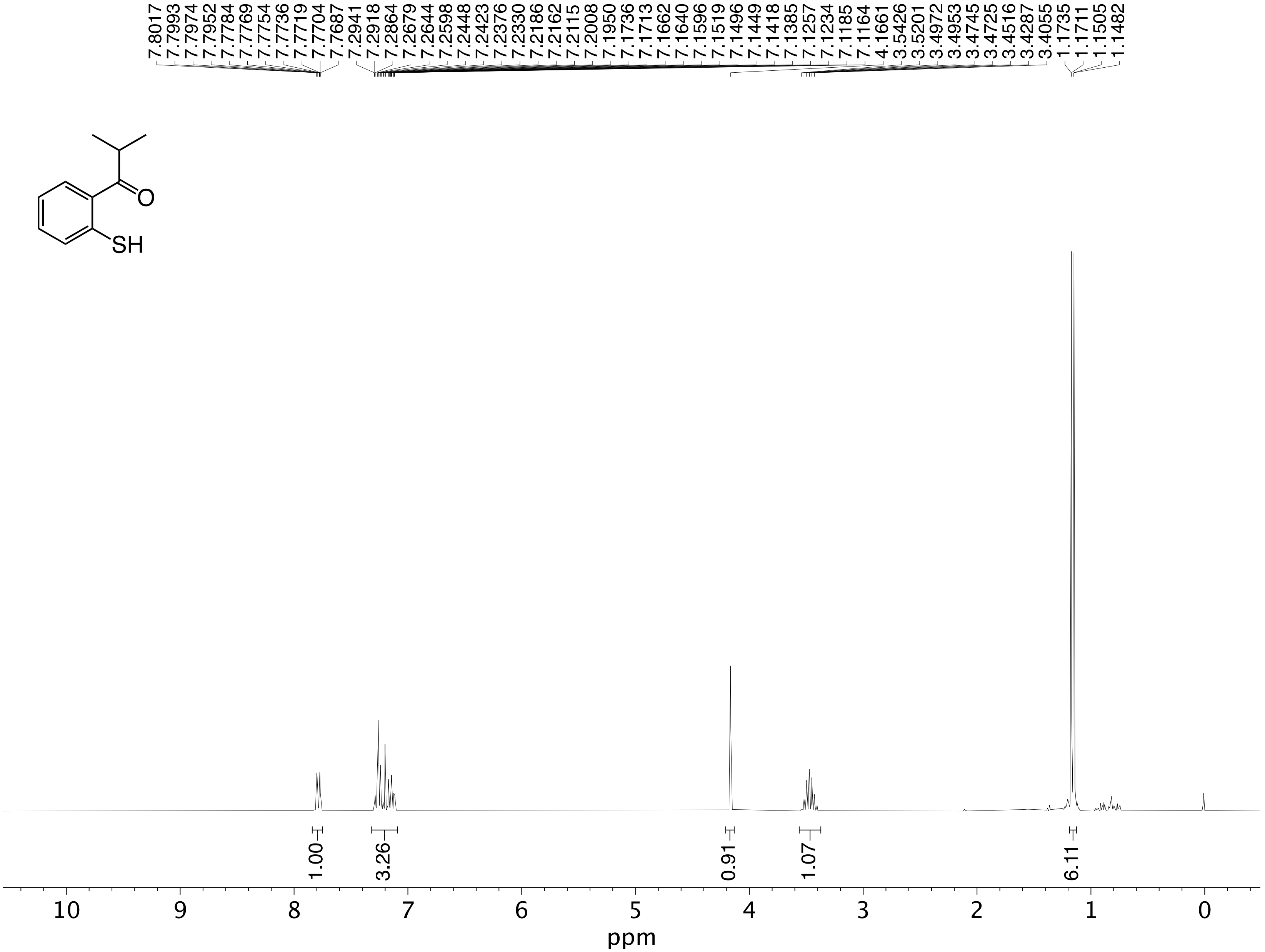

^{1}H NMR (300 MHz, $CDCl_3$) of compound **SI-50.**

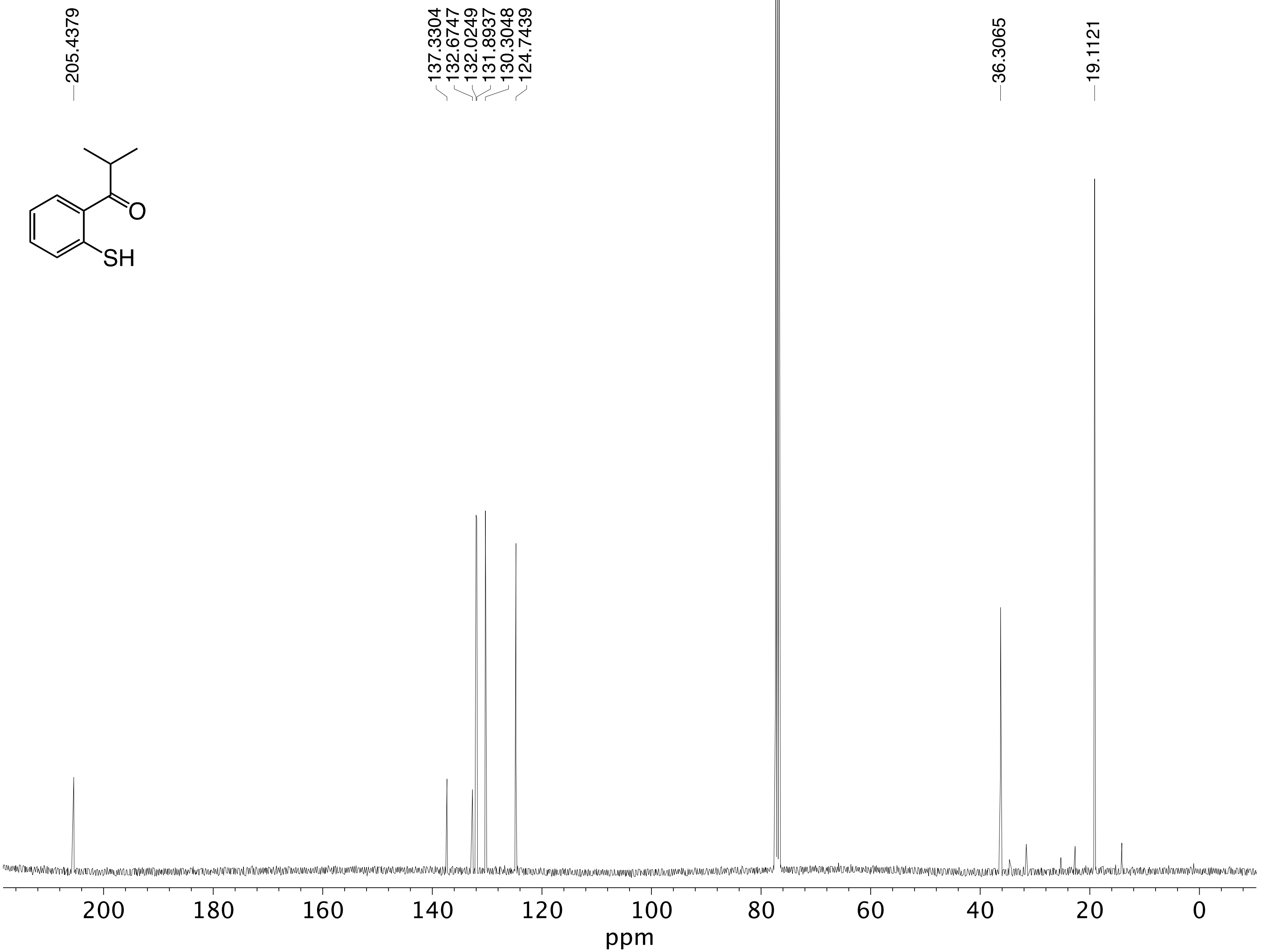

^{13}C NMR (101 MHz, $CDCl_3$) of compound **SI-50.**

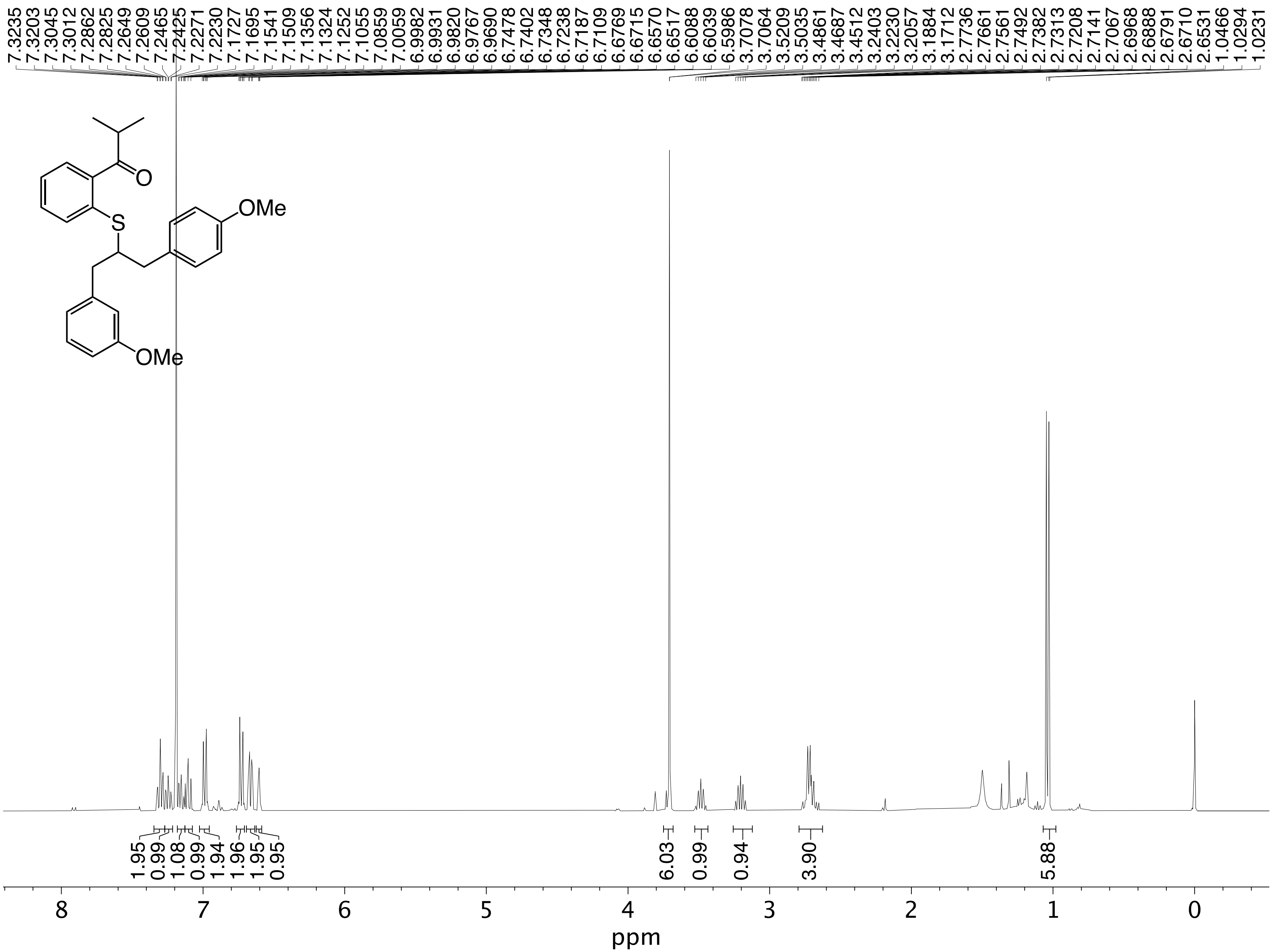

^{1}H NMR (400 MHz, $CDCl_3$) of compound **SI-51.**

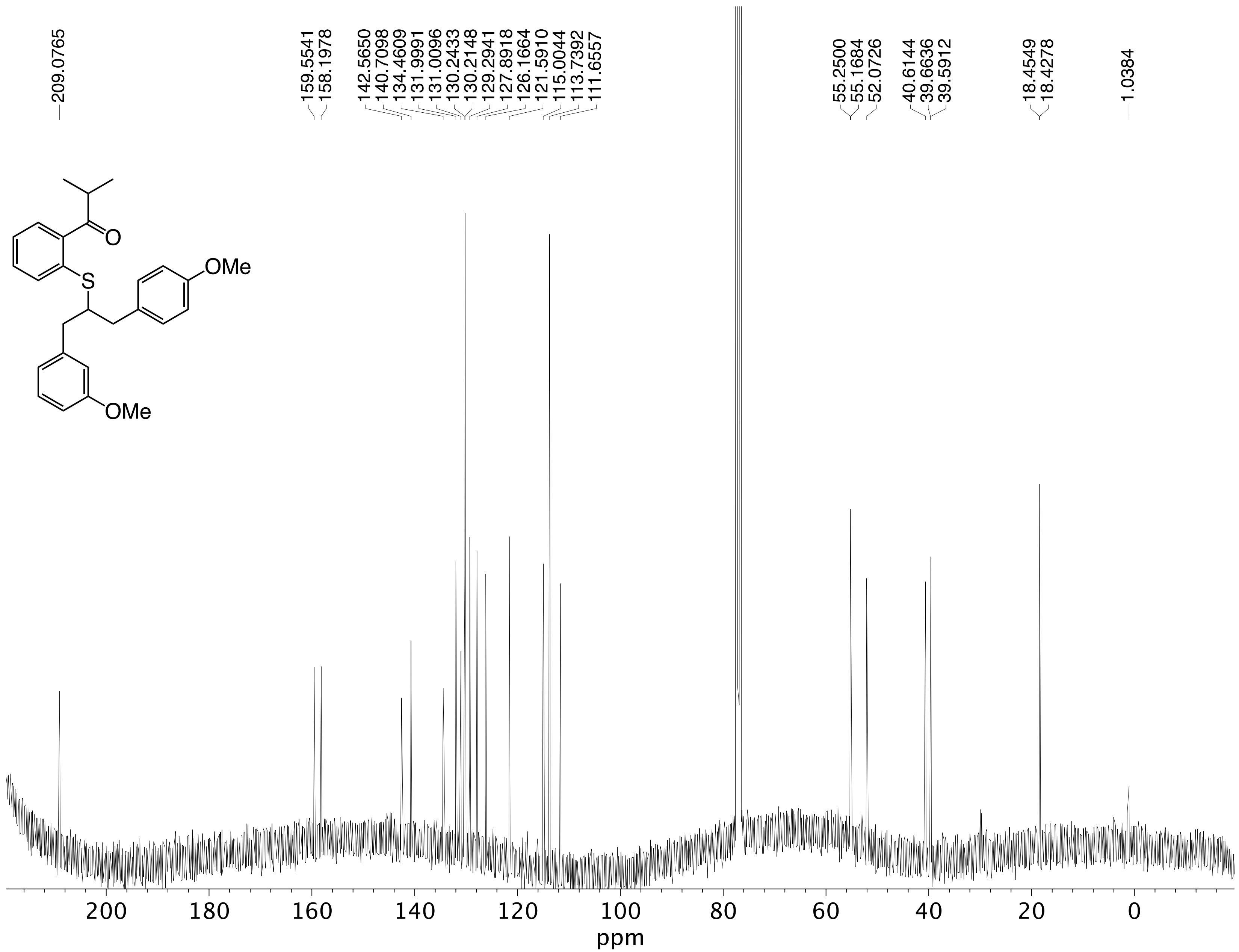

^{13}C NMR (101 MHz, $CDCl_3$) of compound **SI-51.**

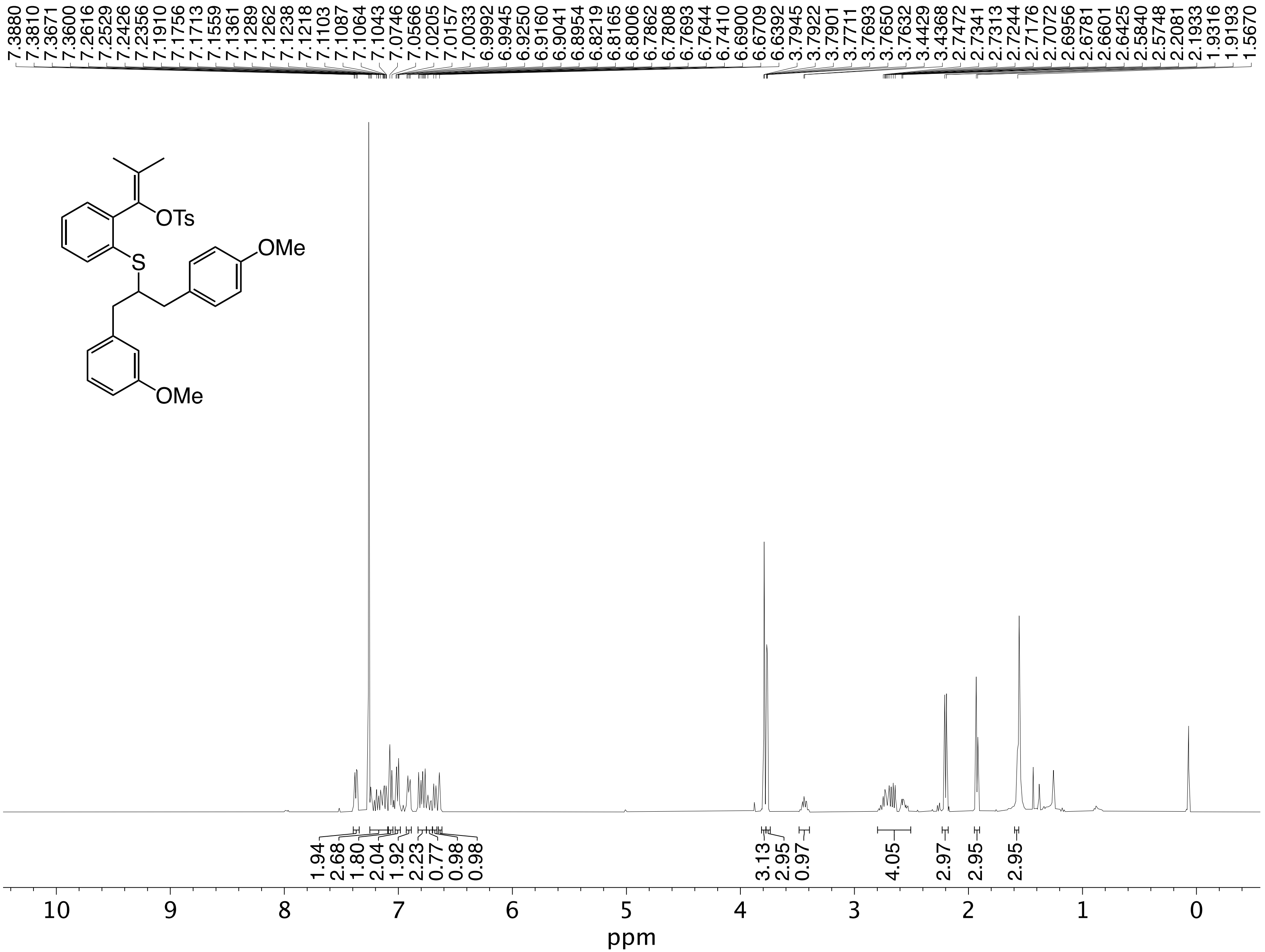

^{1}H NMR (400 MHz, $CDCl_3$) of compound **28**.

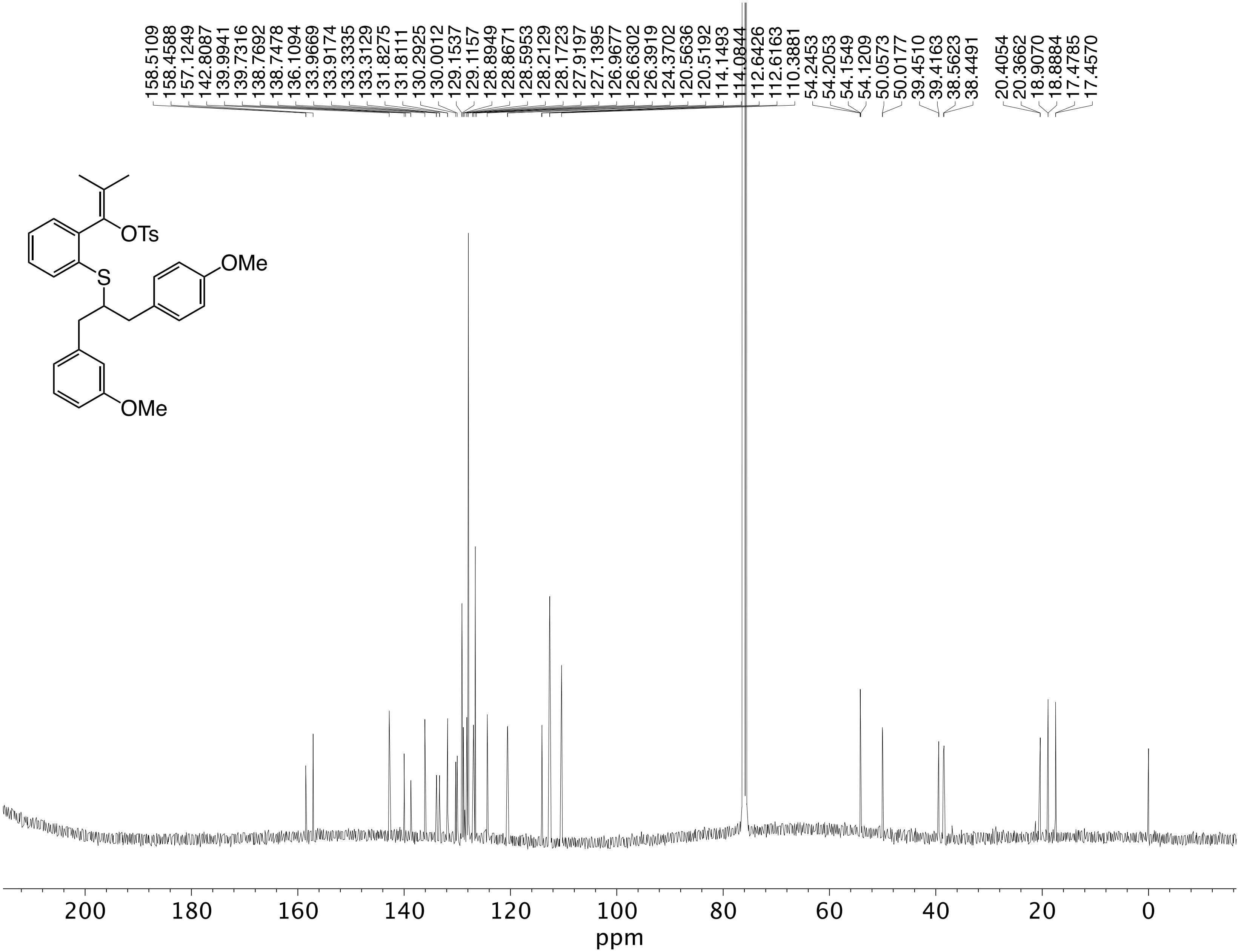

^{13}C NMR (101 MHz, $CDCl_3$) of compound **28**.

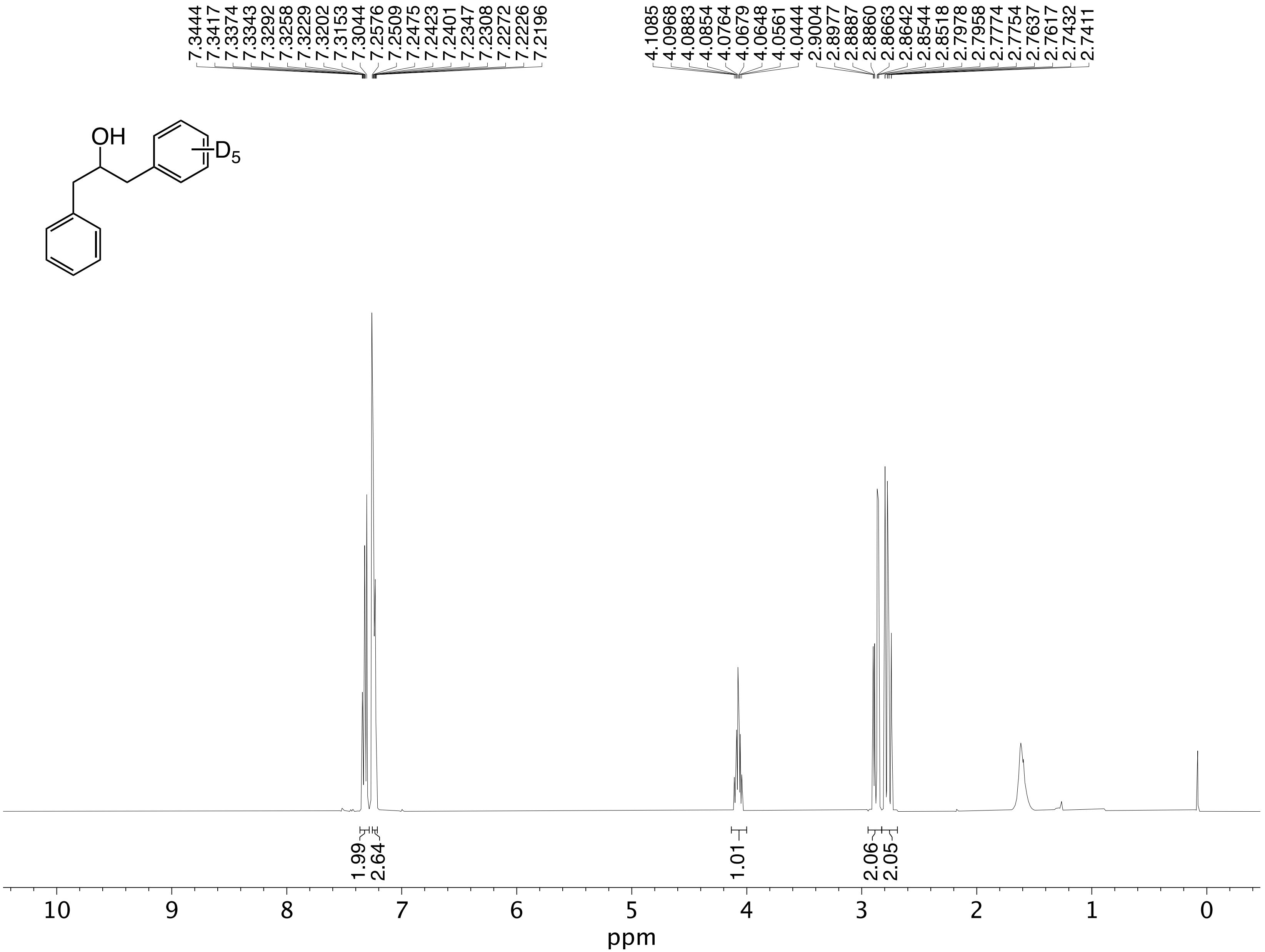

^{1}H NMR (400 MHz, $CDCl_3$) of compound **SI-54**.

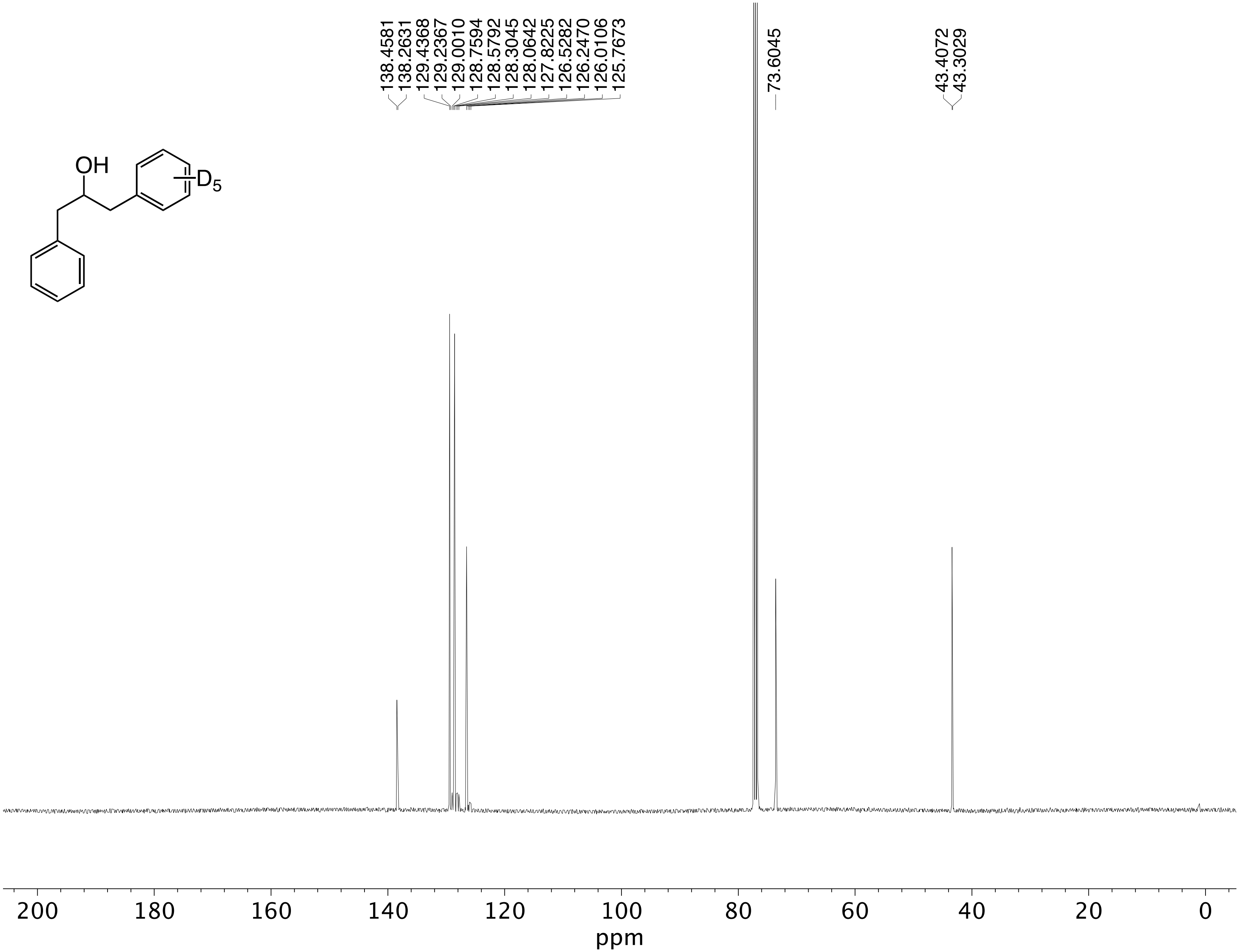

^{13}C NMR (101 MHz, $CDCl_3$) of compound **SI-54**.

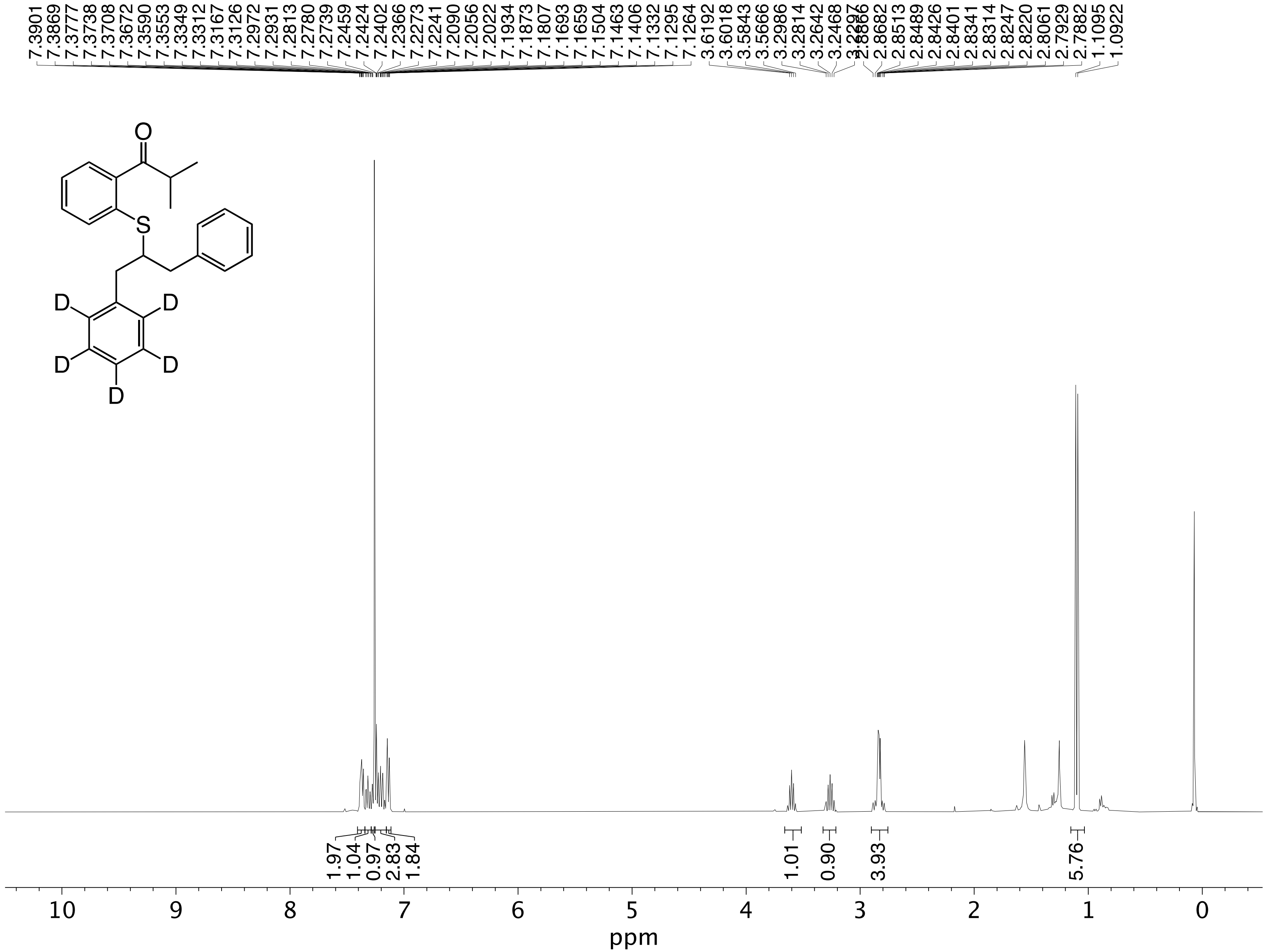

^{1}H NMR (400 MHz, $CDCl_3$) of compound **SI-56**.

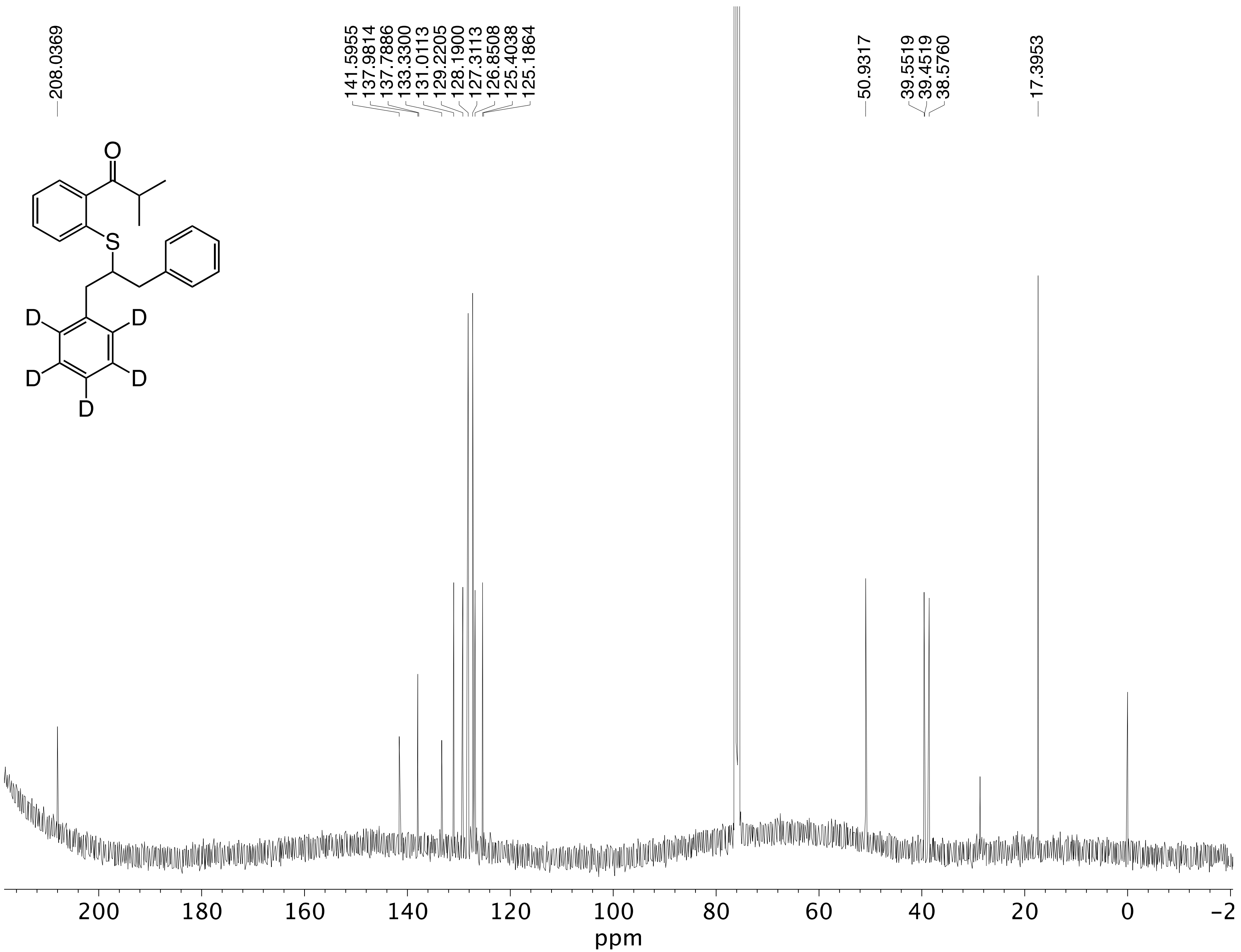

^{13}C NMR (101 MHz, $CDCl_3$) of compound **SI-56**.

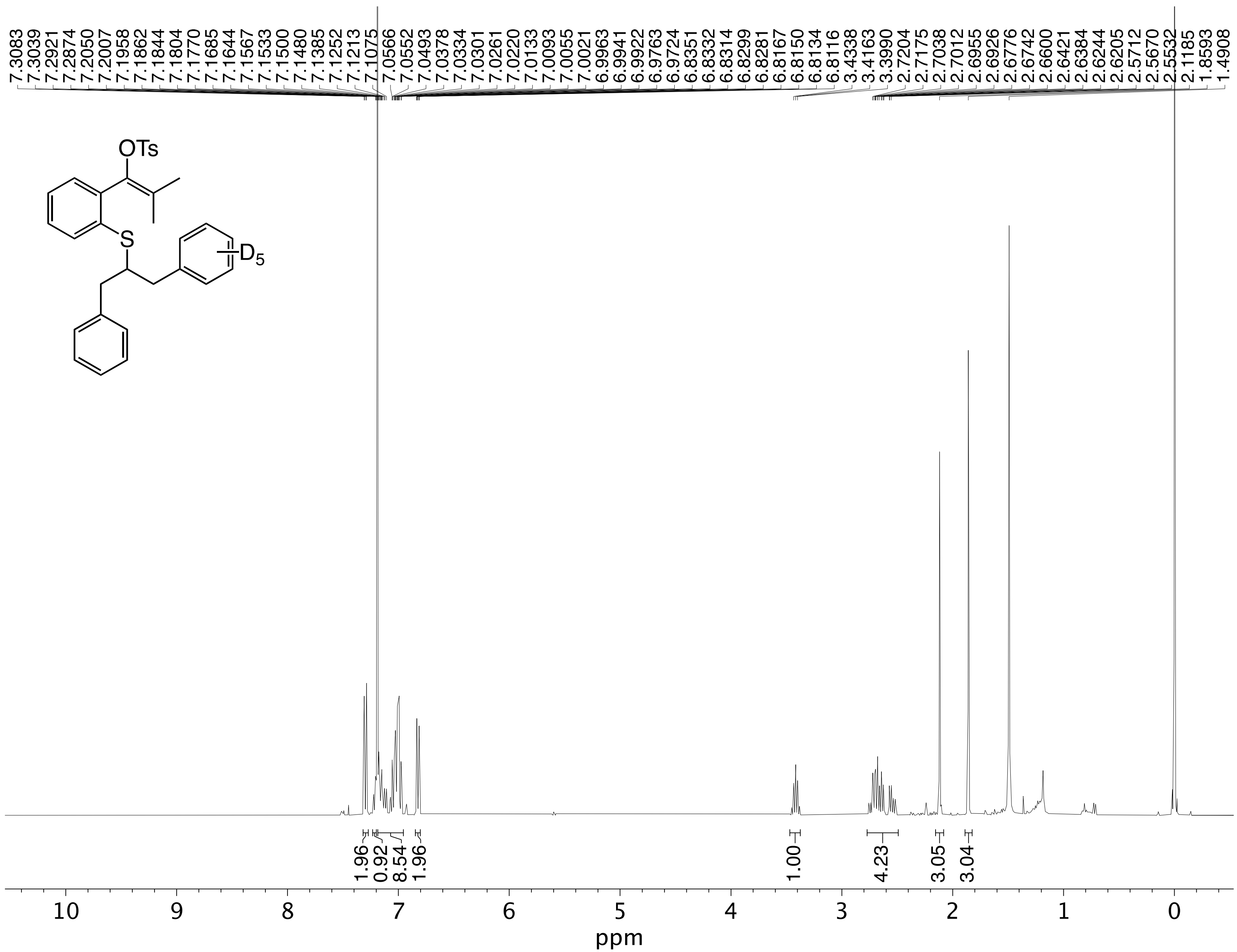

^{1}H NMR (400 MHz, $CDCl_3$) of compound **30**.

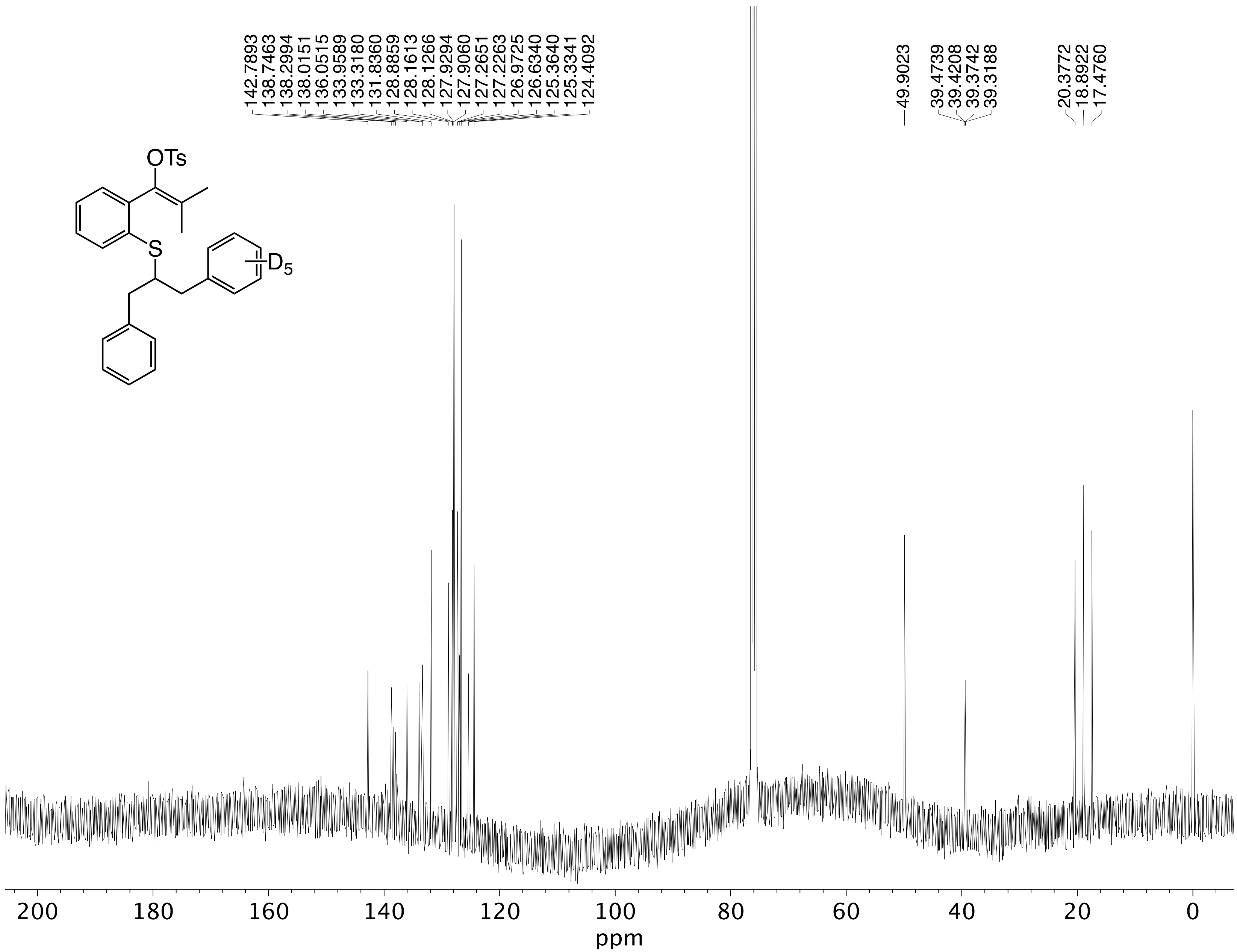

^{13}C NMR (101 MHz, $CDCl_3$) of compound **30**.

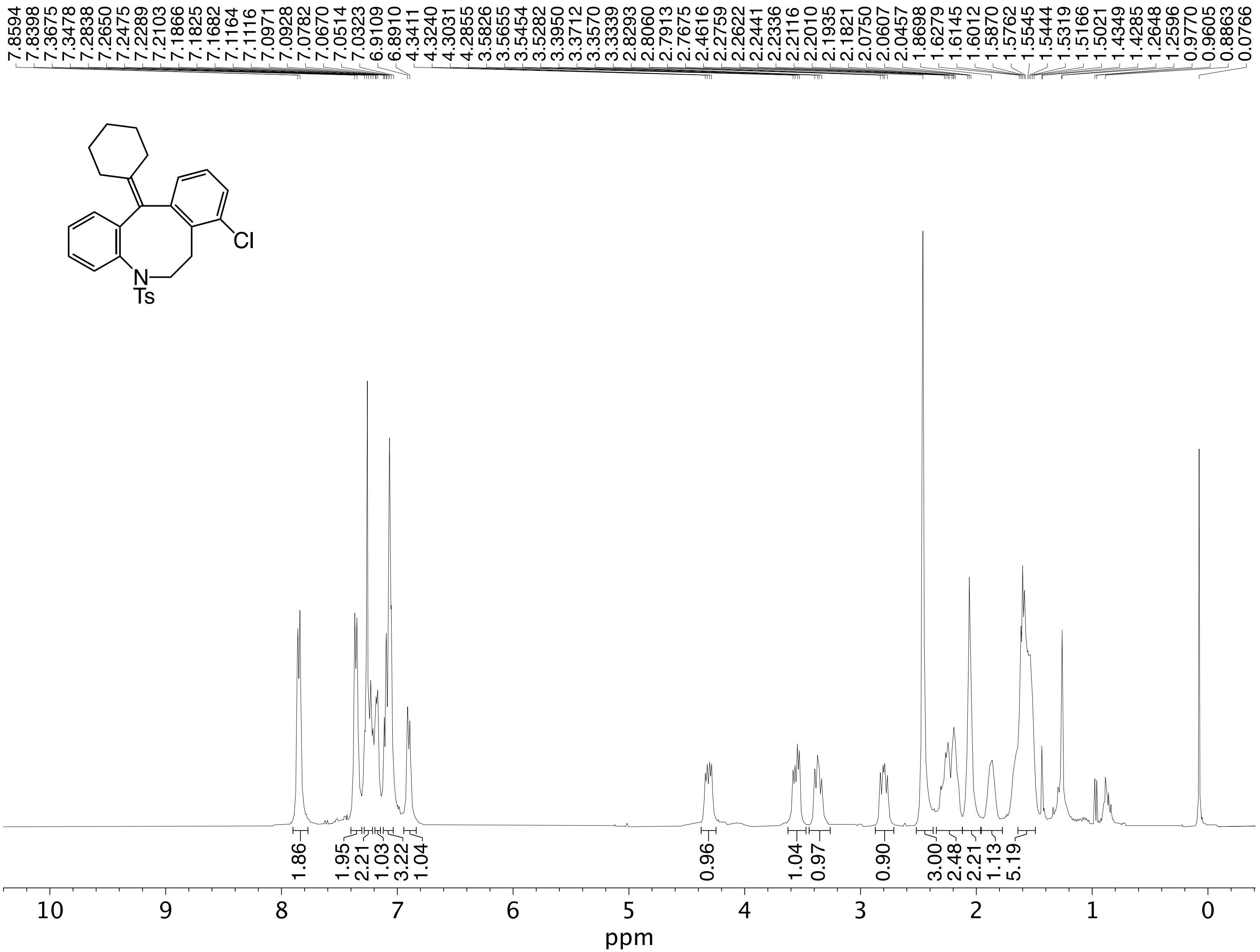

^{1}H NMR (400 MHz, $CDCl_3$) of compound **2**.

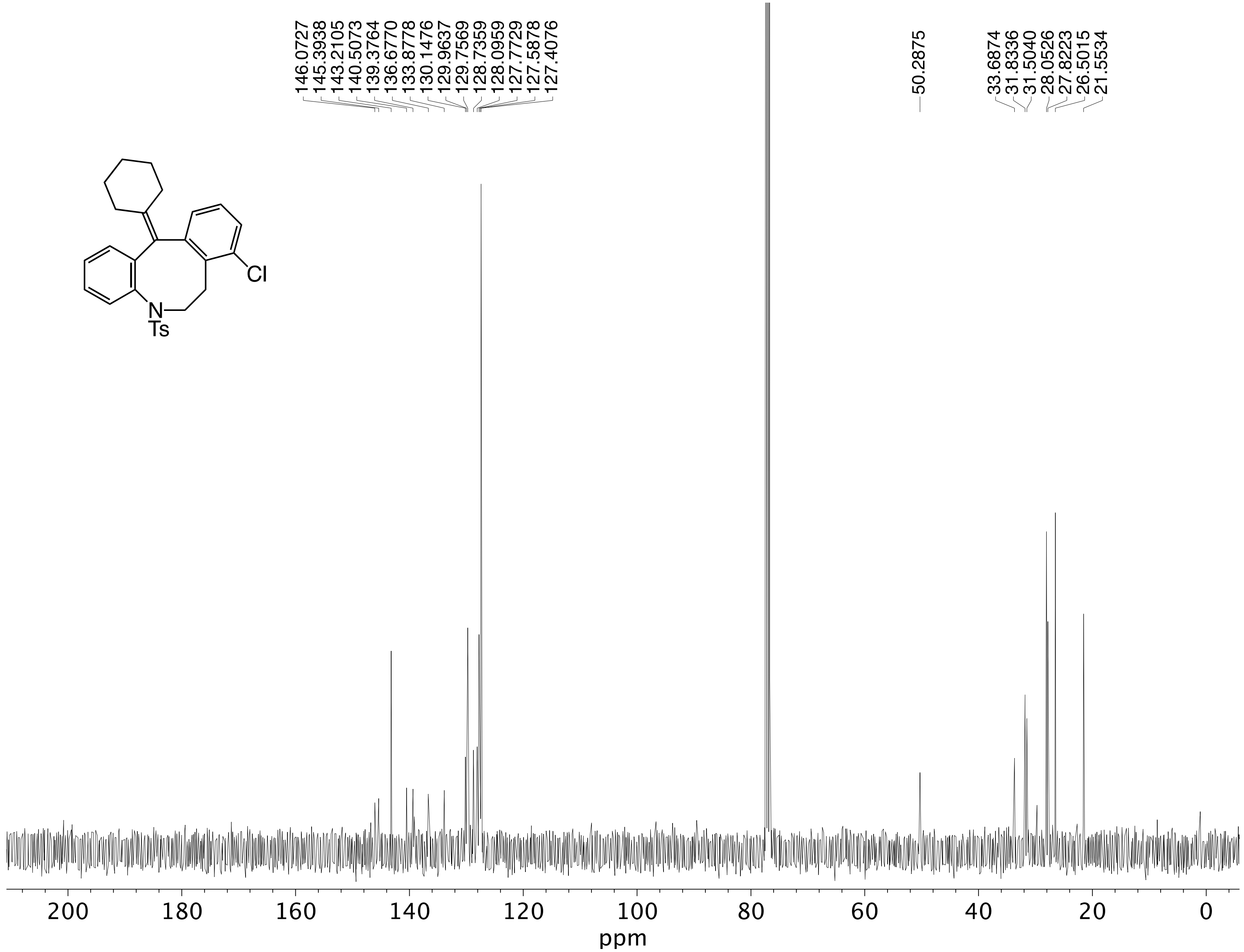

^{13}C NMR (101 MHz, $CDCl_3$) of compound **2**.

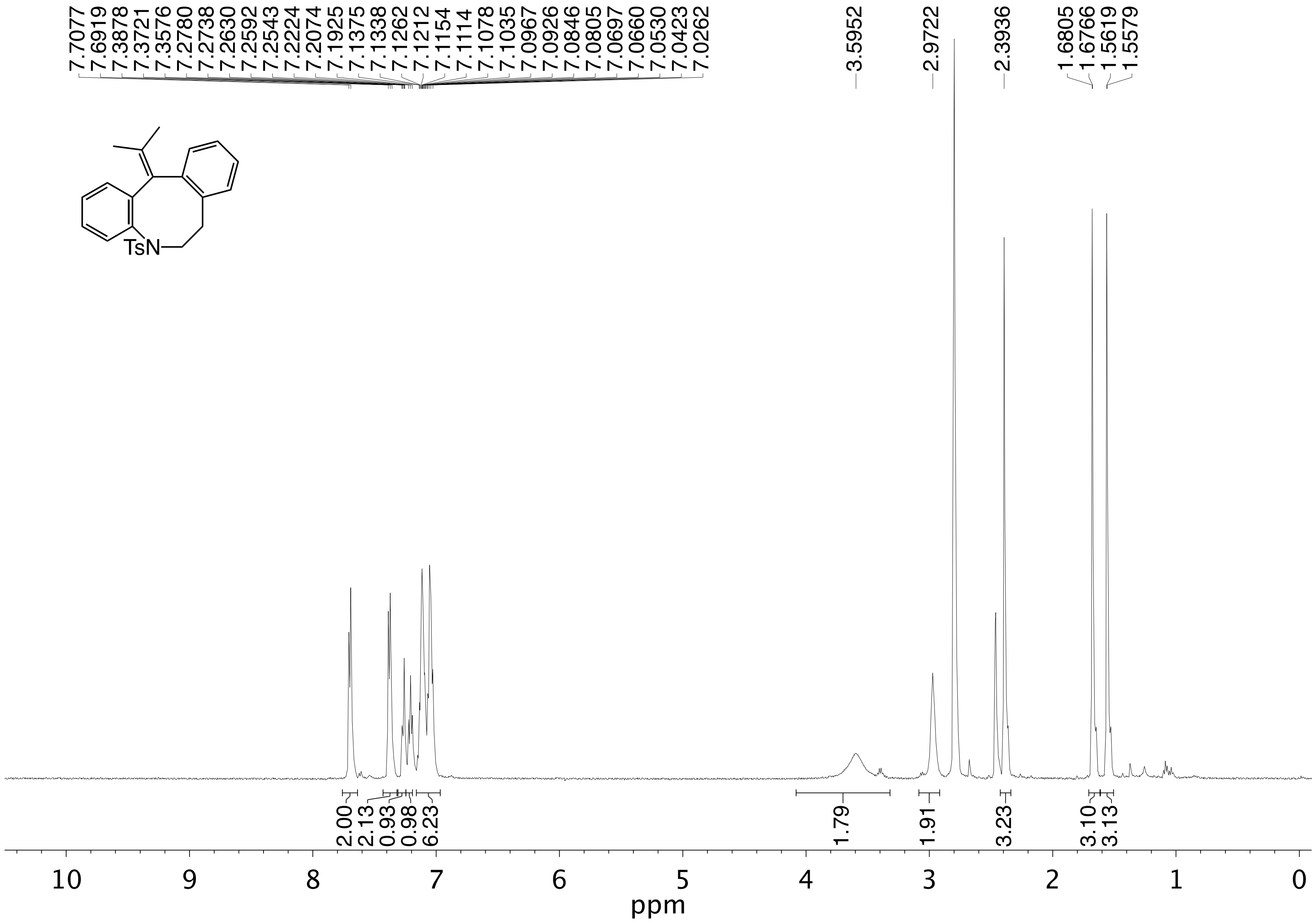

^{1}H NMR (500 MHz, DMSO-d_6, 125°C) of compound **5**.

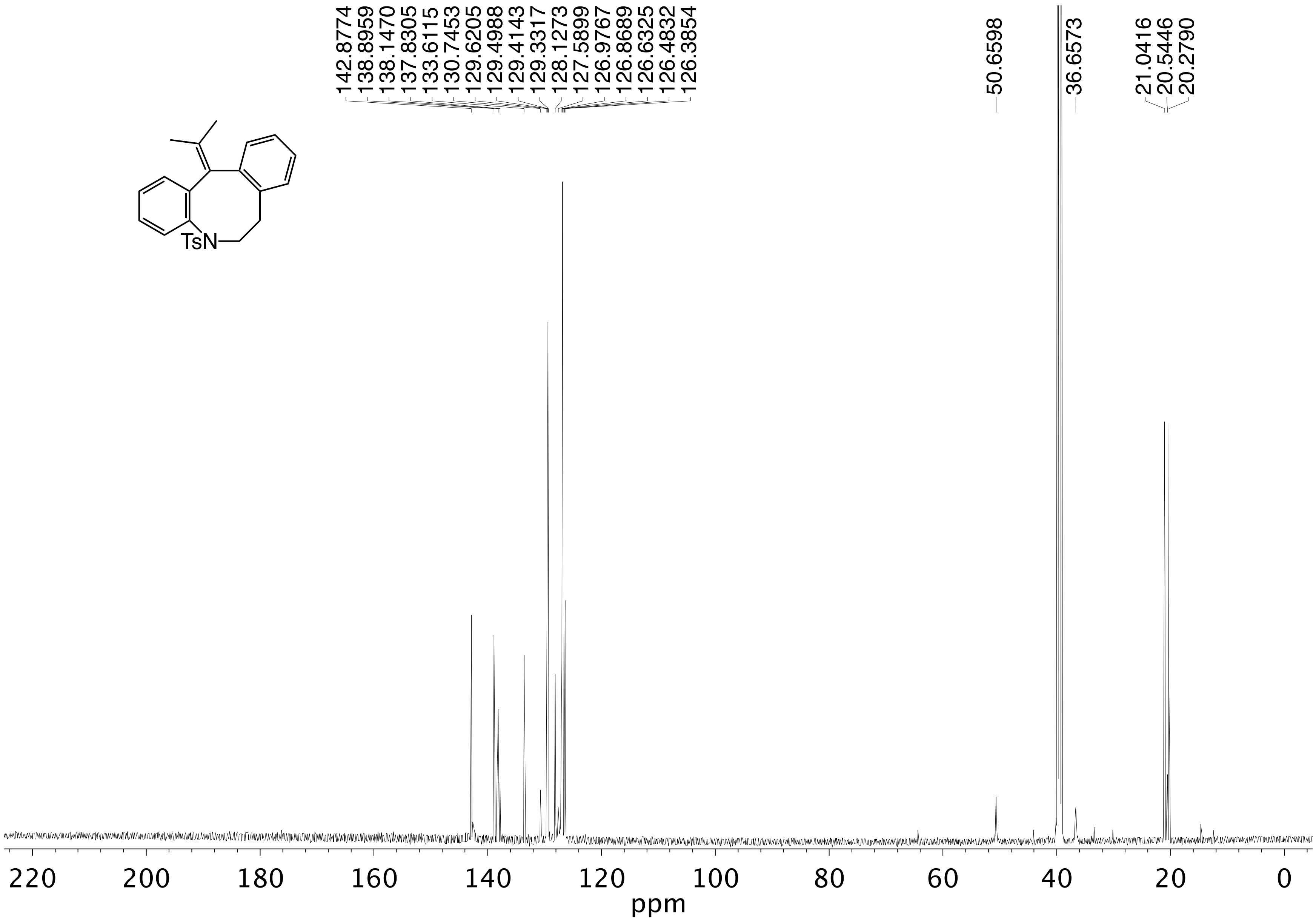

^{13}C NMR (126 MHz, DMSO-d_6, 75°C) of compound **5**.

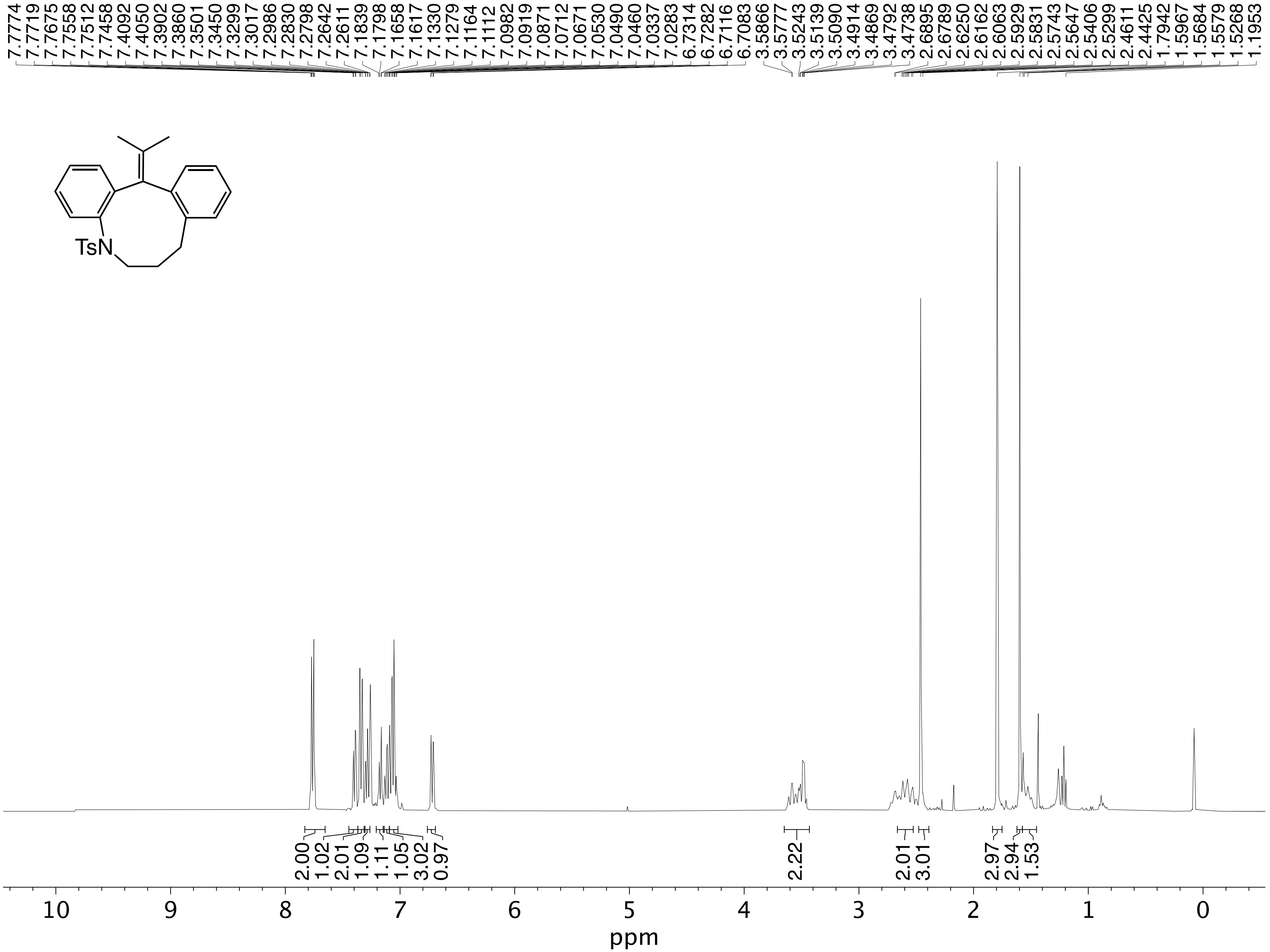

^{1}H NMR (500 MHz, $CDCl_3$) of compound **6**.

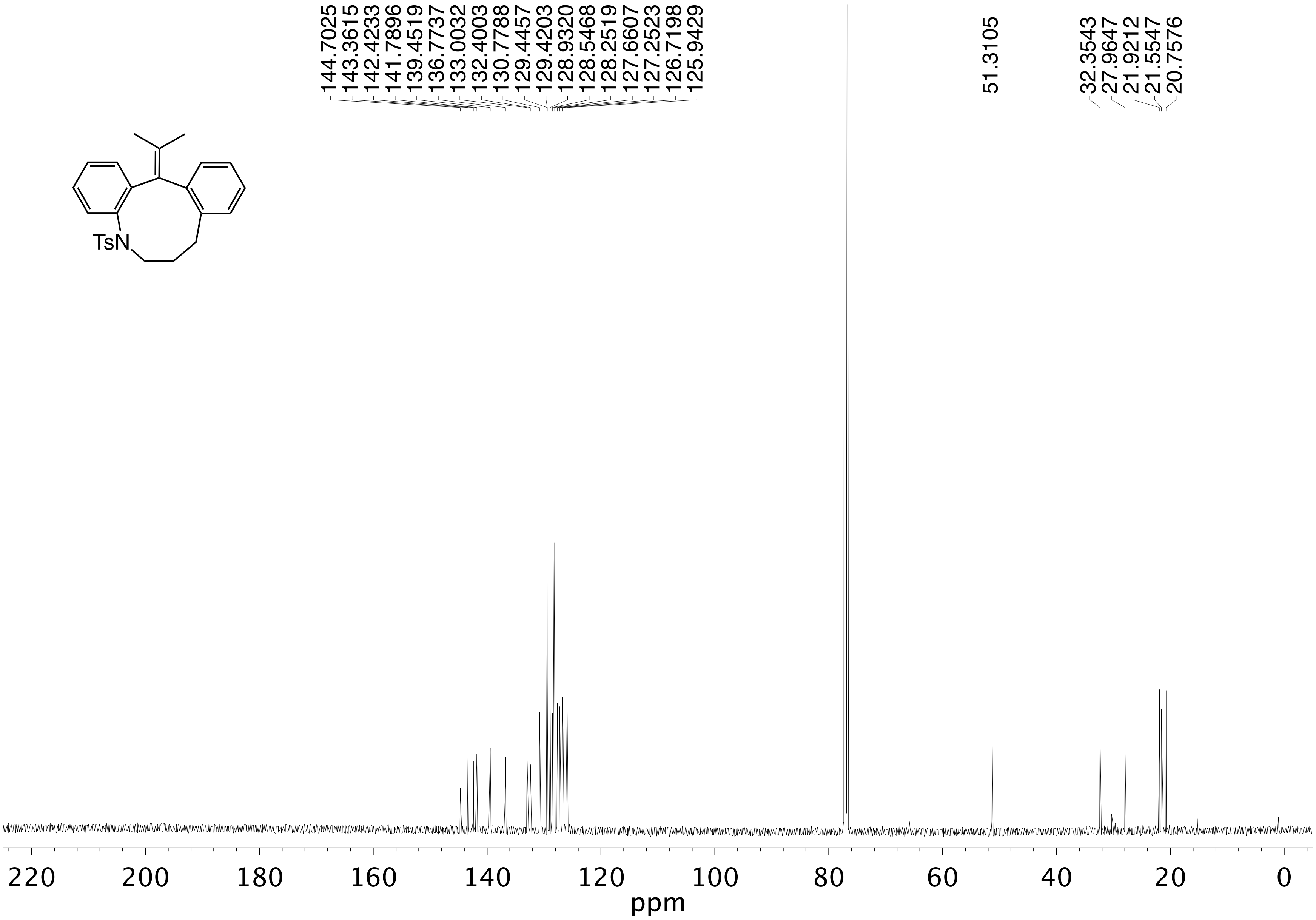

^{13}C NMR (126 MHz, $CDCl_3$) of compound **6**.

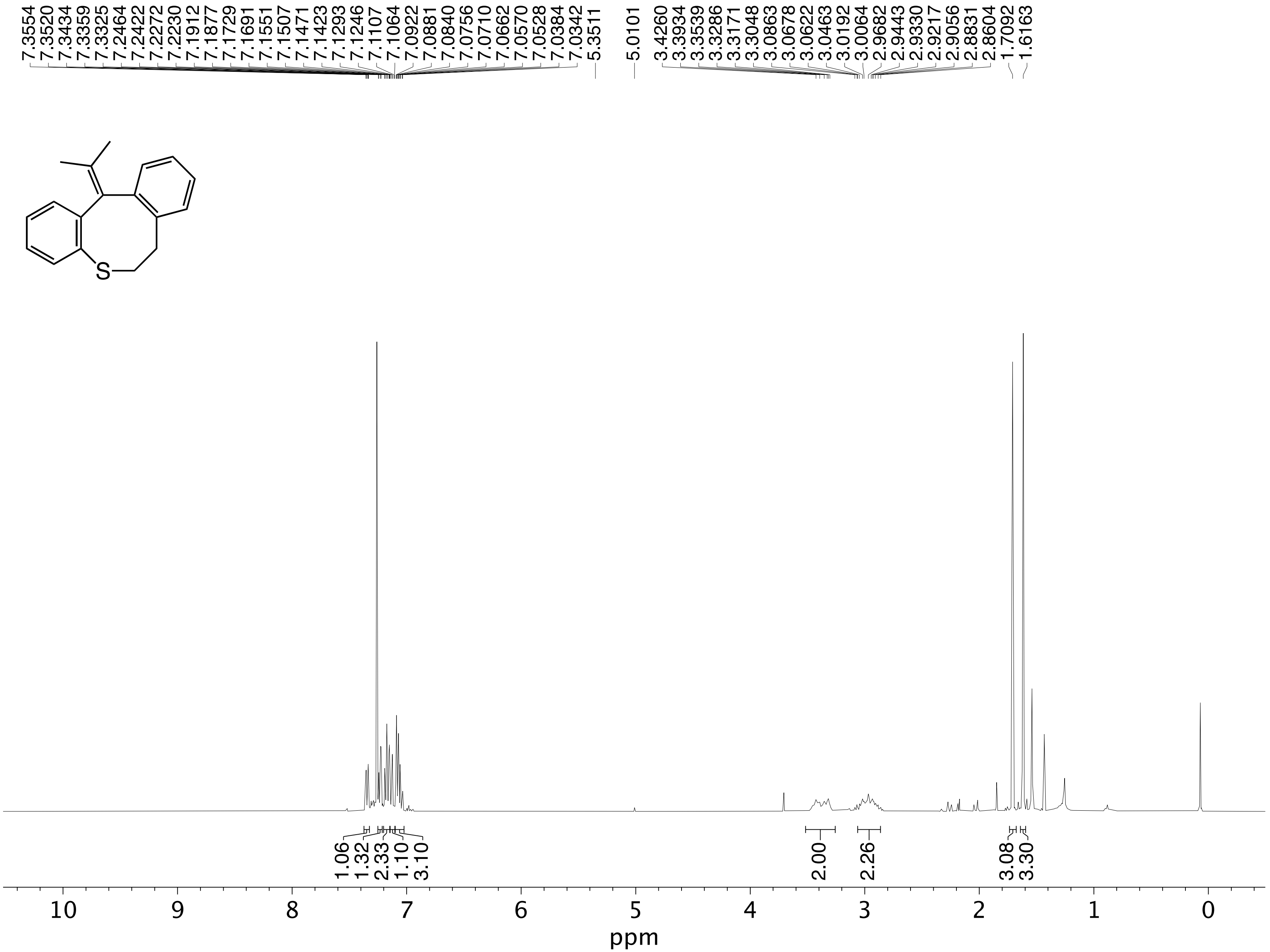

^{1}H NMR (400 MHz, $CDCl_3$) of compound **8**.

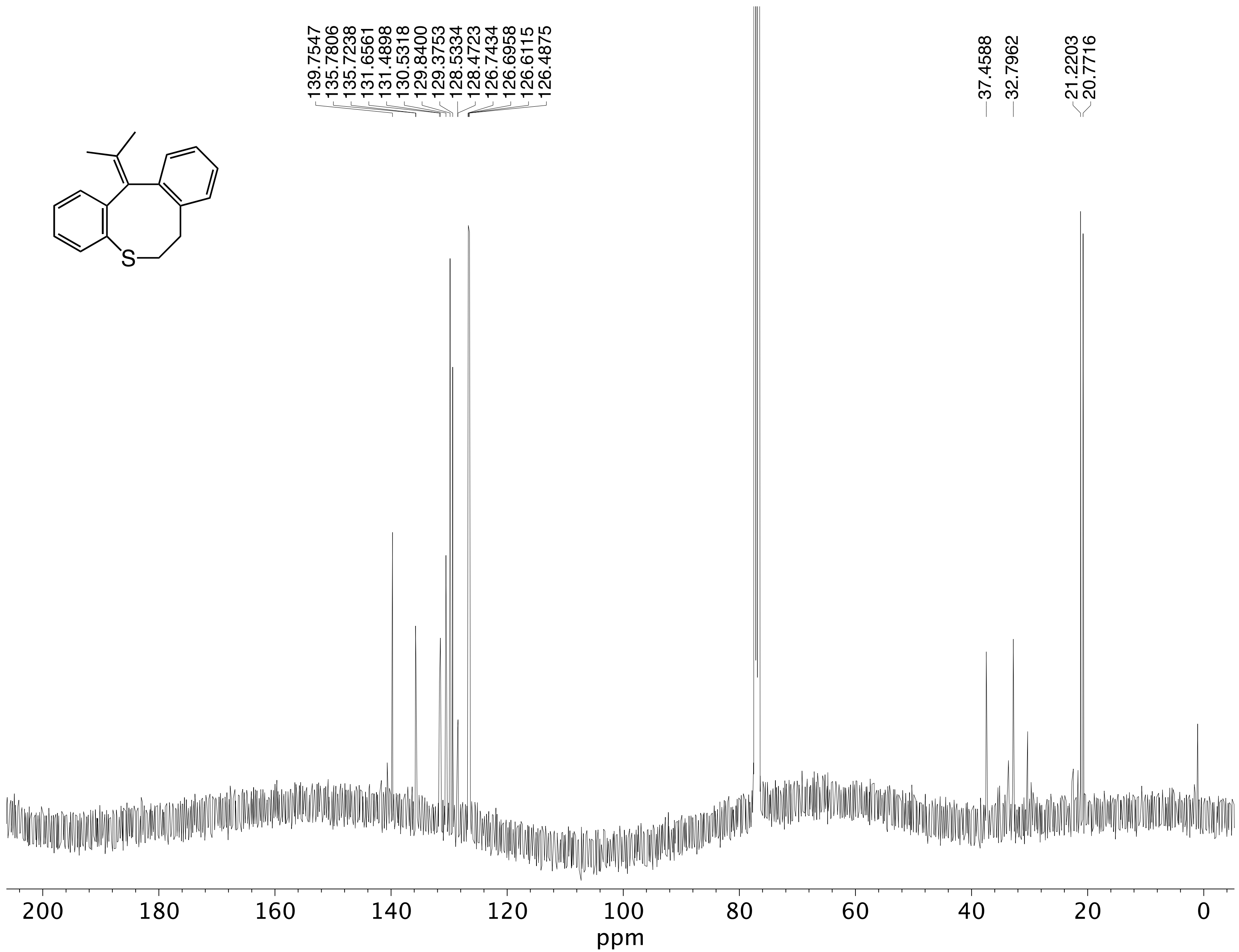

^{13}C NMR (101 MHz, $CDCl_3$) of compound **8**.

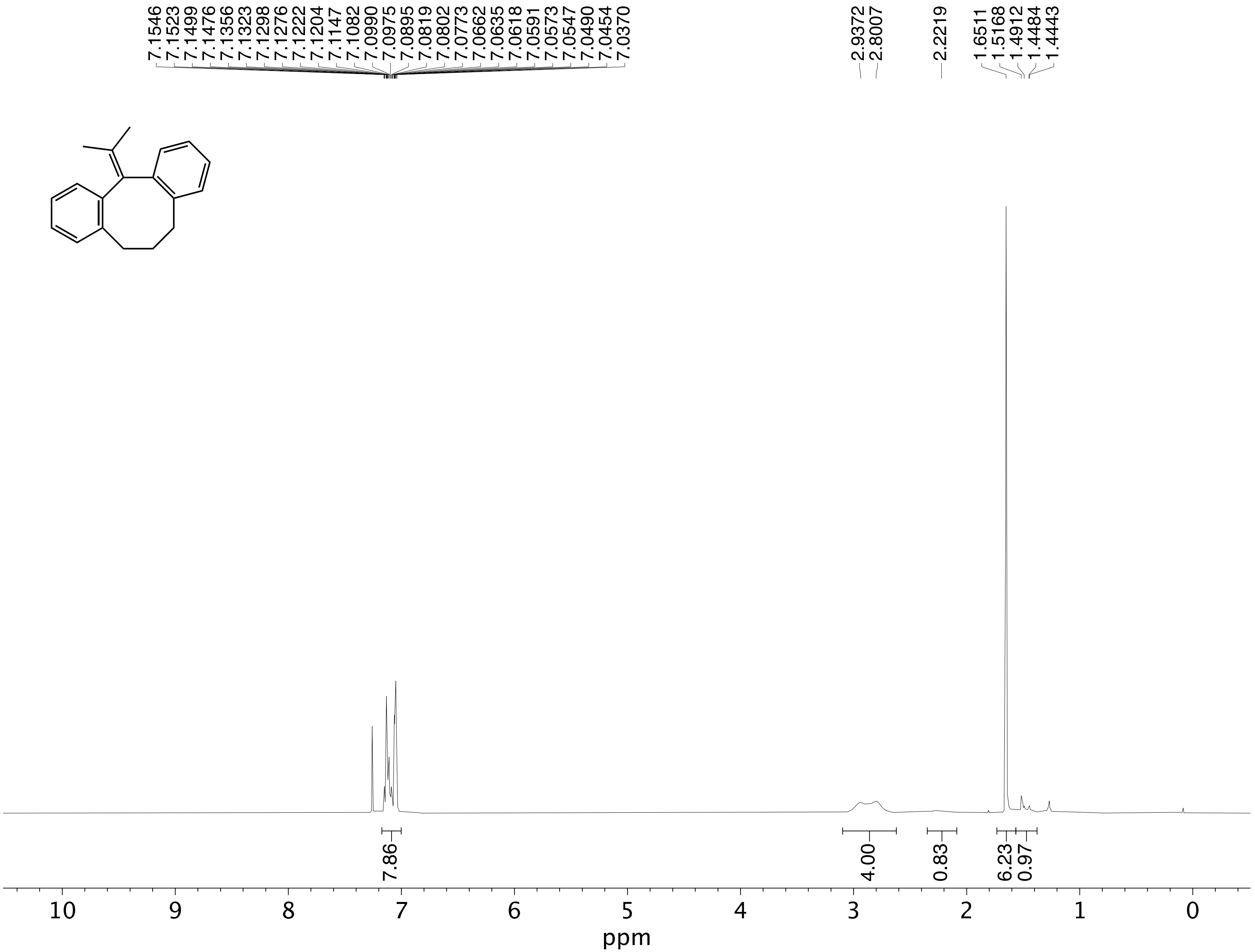

^{1}H NMR (400 MHz, $CDCl_3$) of compound **9**.

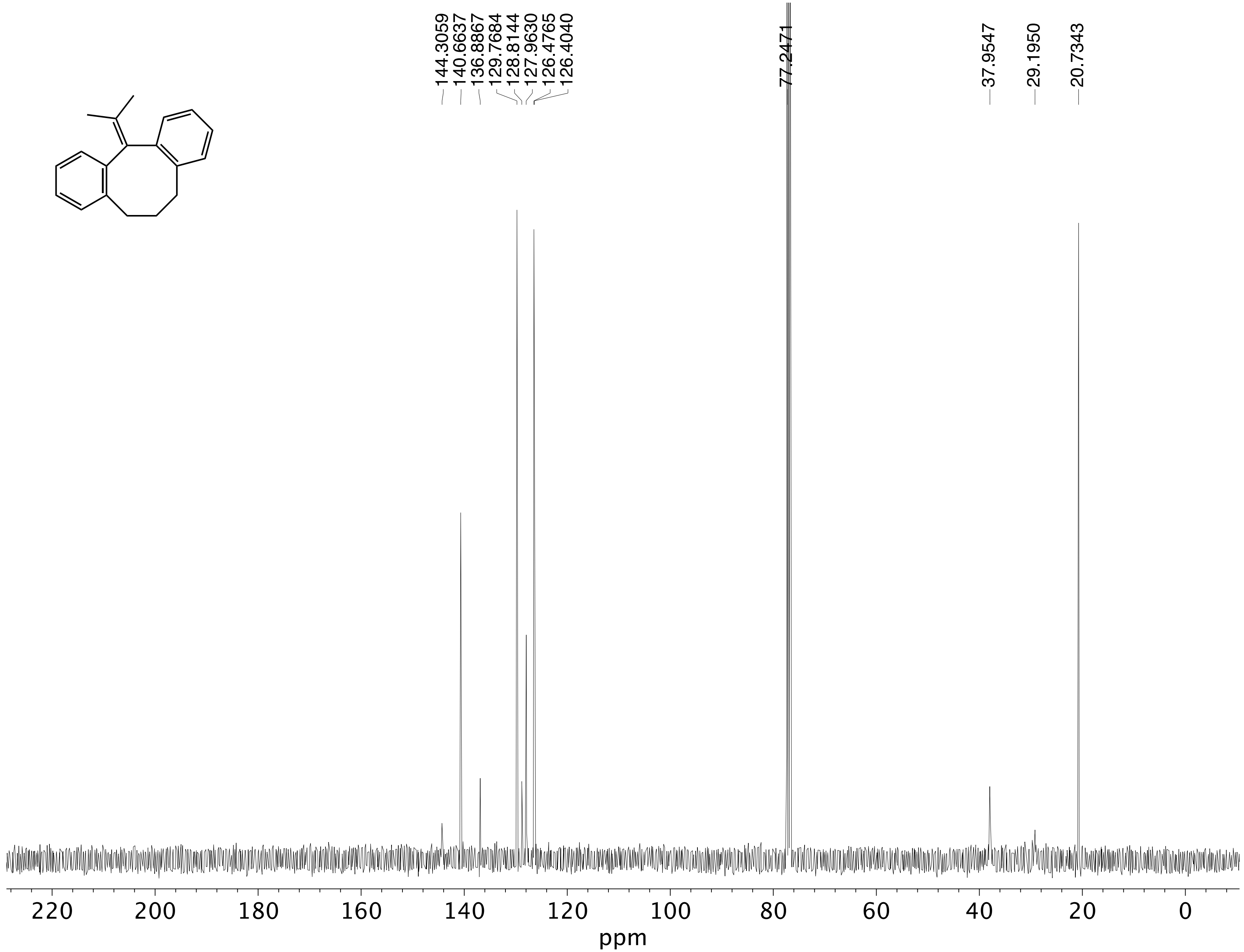

^{13}C NMR (101 MHz, CDCl3) of compound **9**.

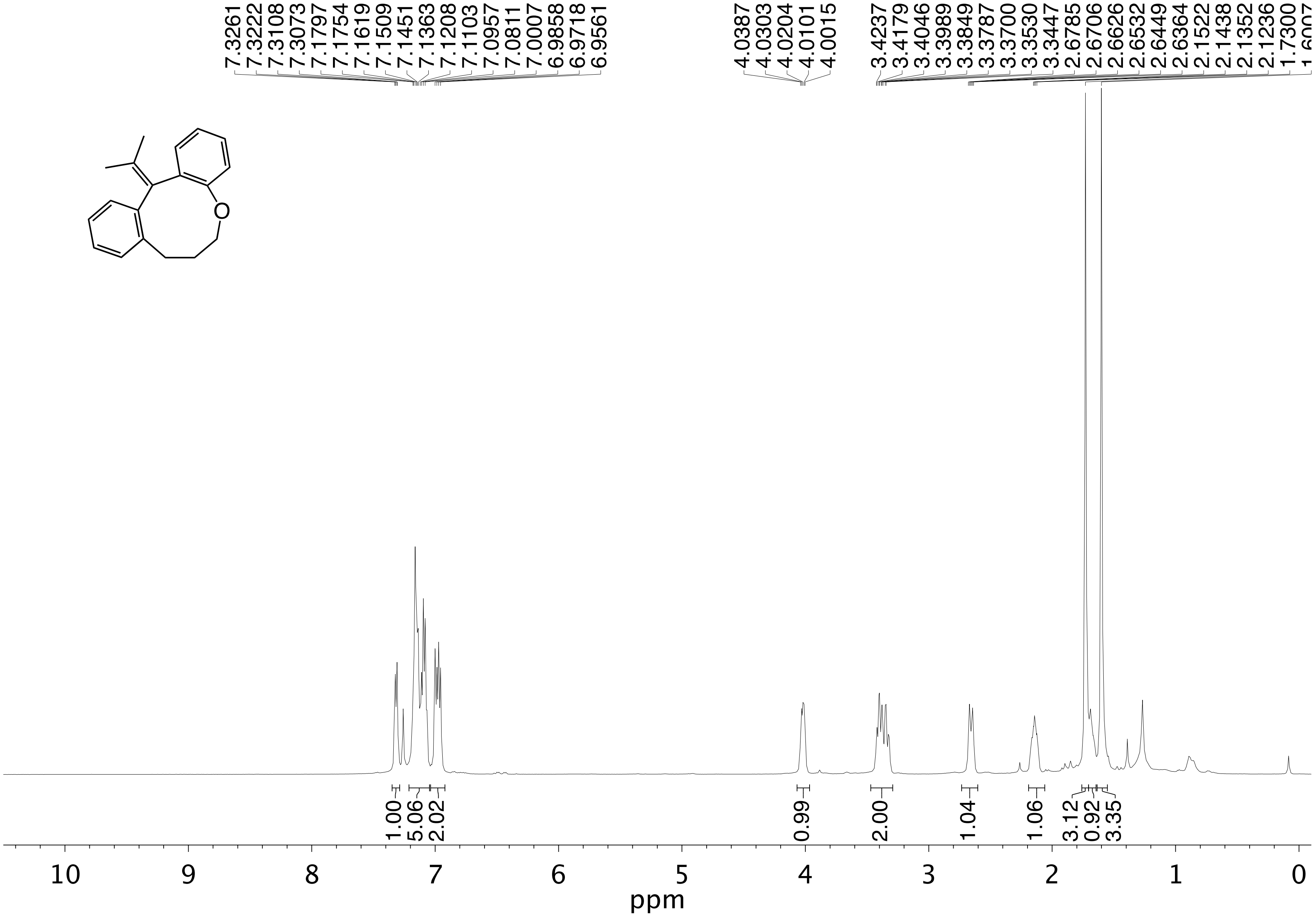

^1H NMR (500 MHz, $CDCl_3$) of compound **10.**

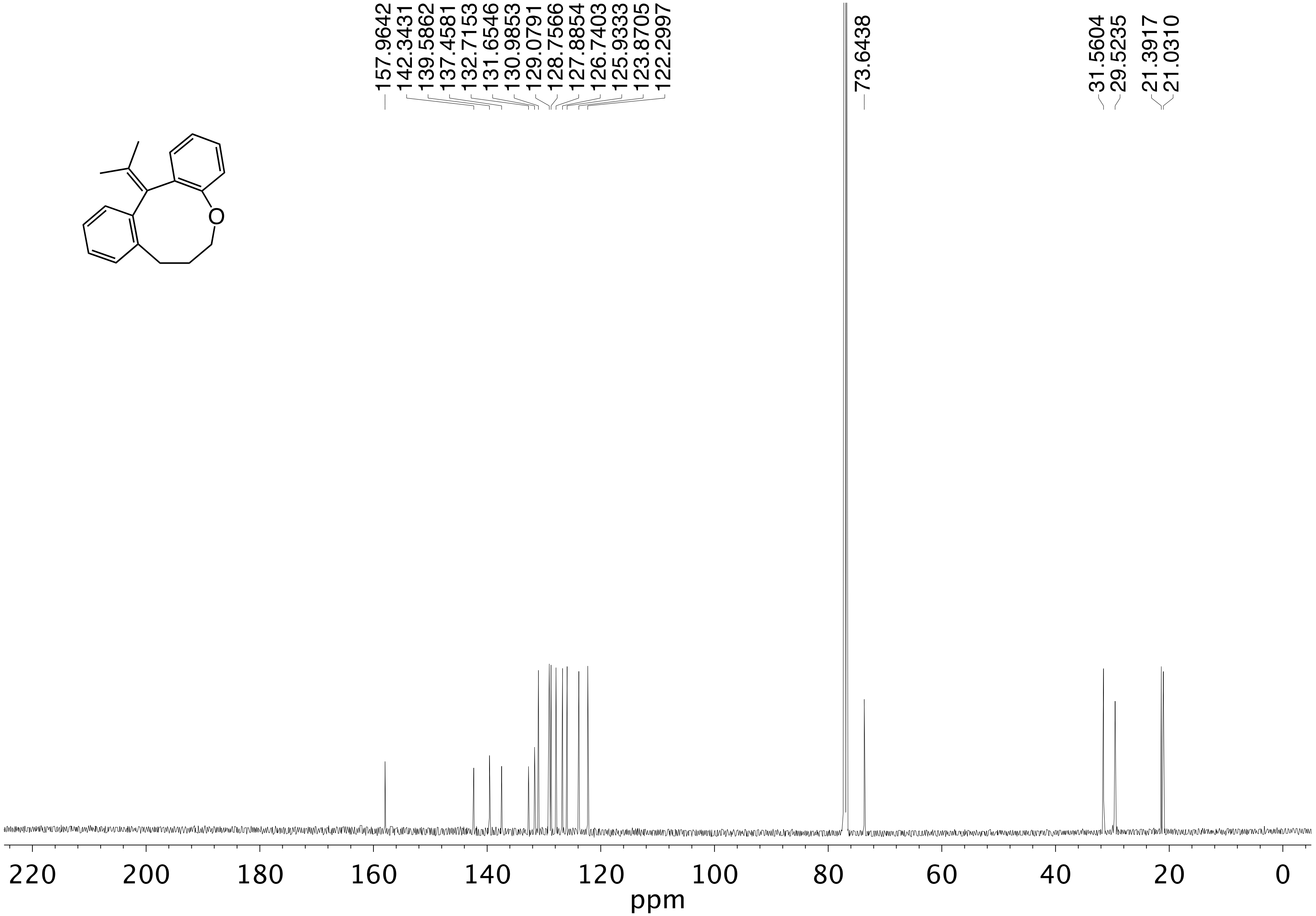

^{13}C NMR (126 MHz, $CDCl_3$) of compound **10.**

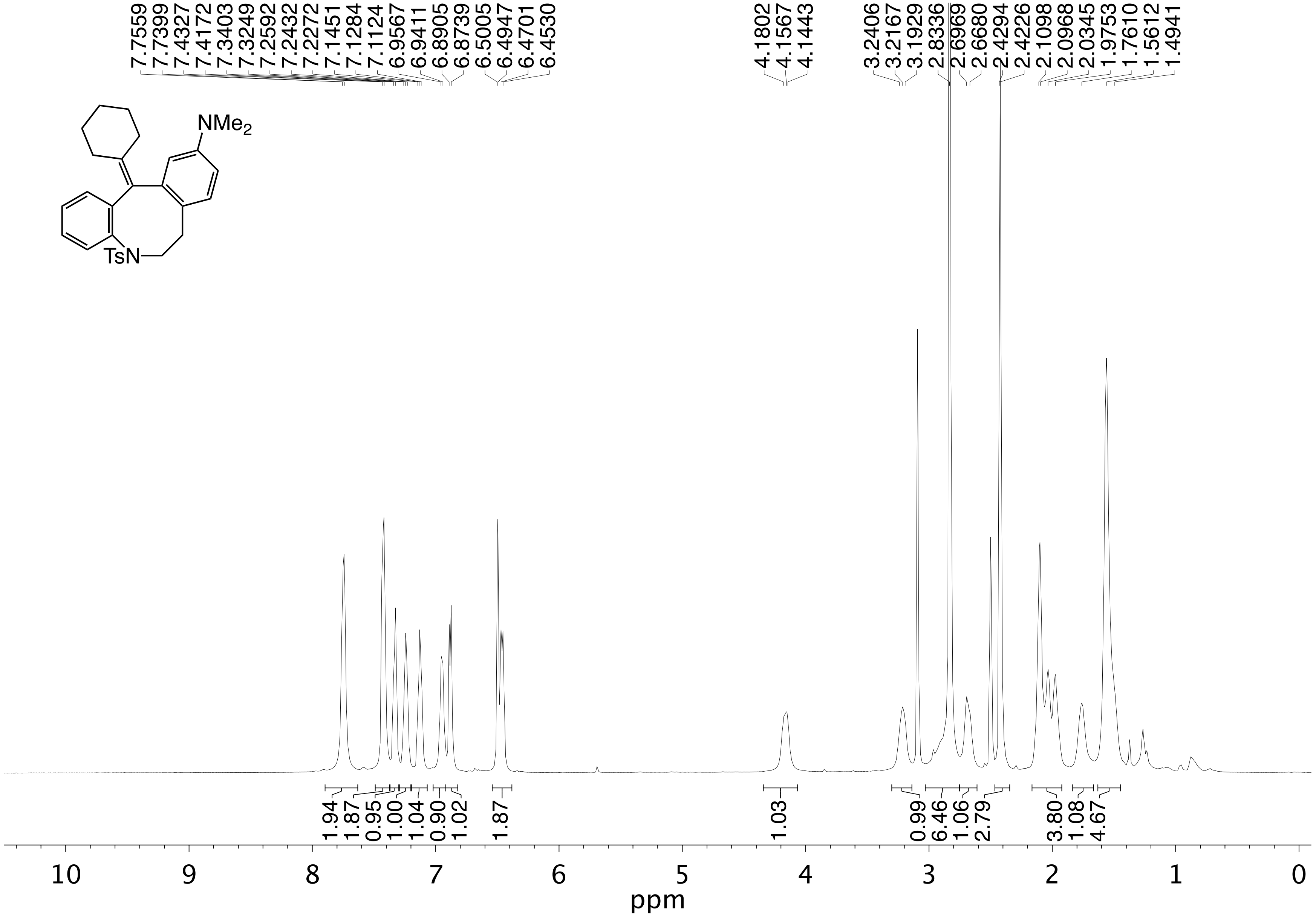

^{1}H NMR (500 MHz, DMSO-d_6, 75°C) of compound **11**.

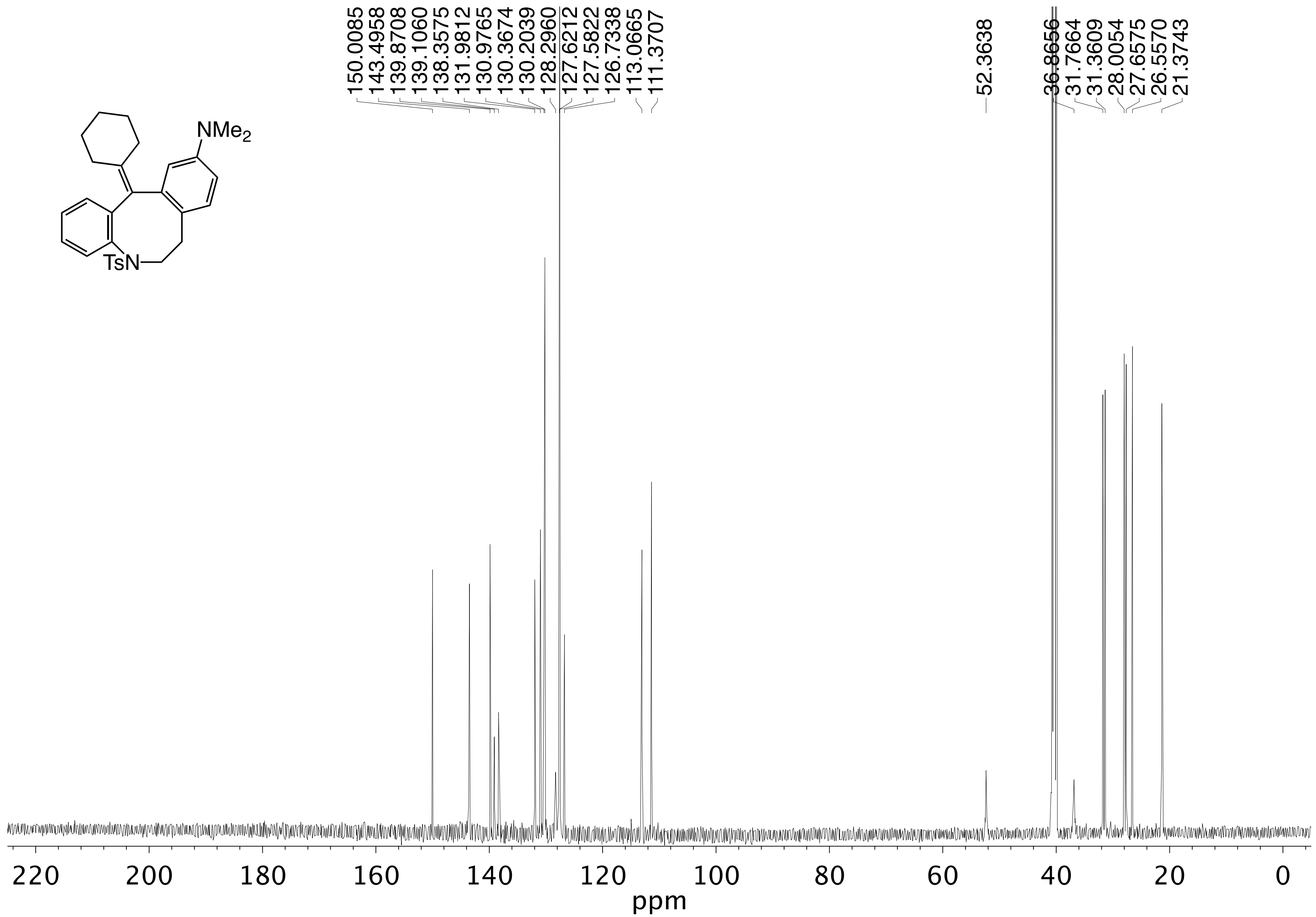

^{13}C NMR (126 MHz, DMSO-d_6, 70°C) of compound **11**.

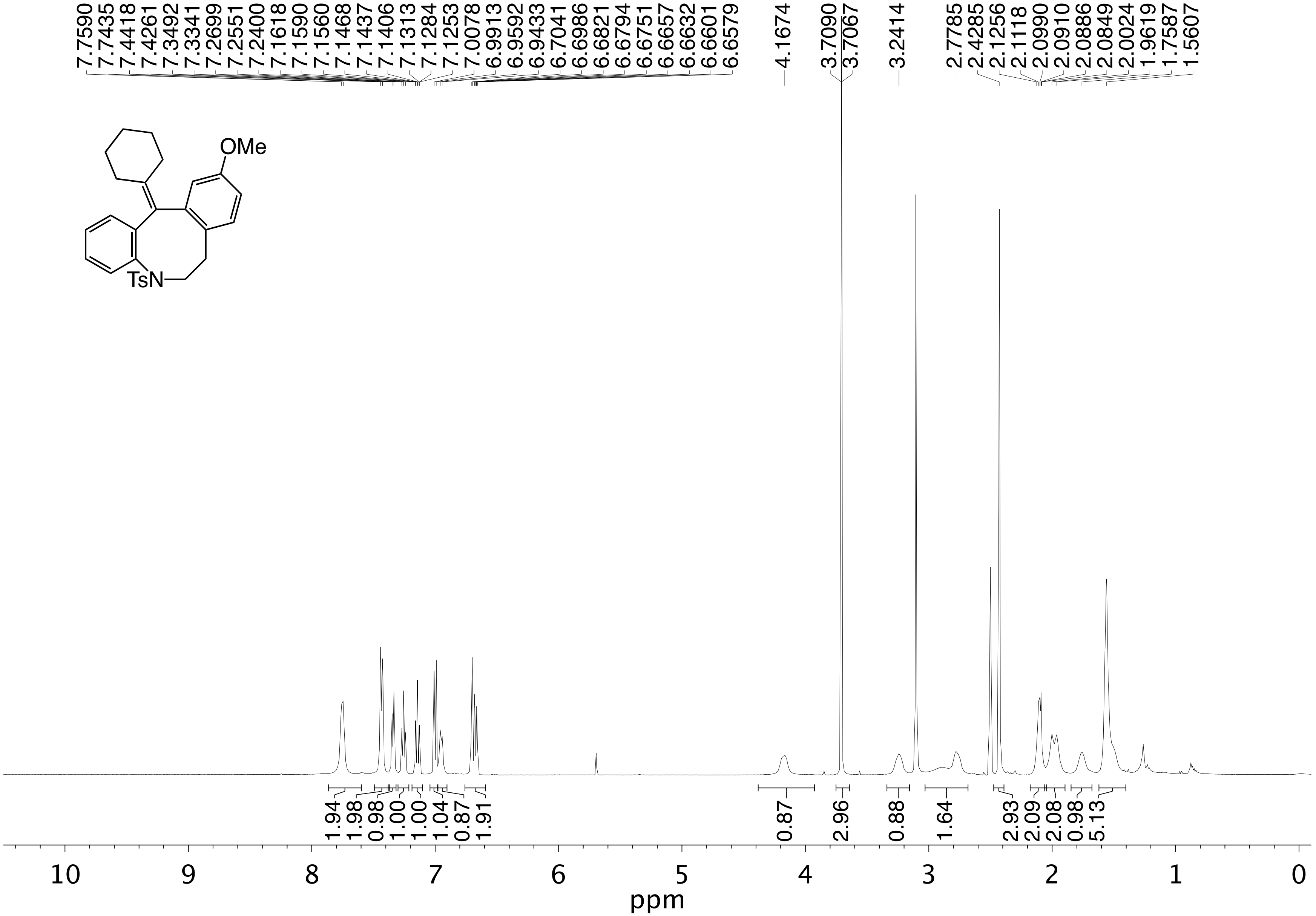

^{1}H NMR (500 MHz, DMSO-d_6, 75°C) of compound **12**.

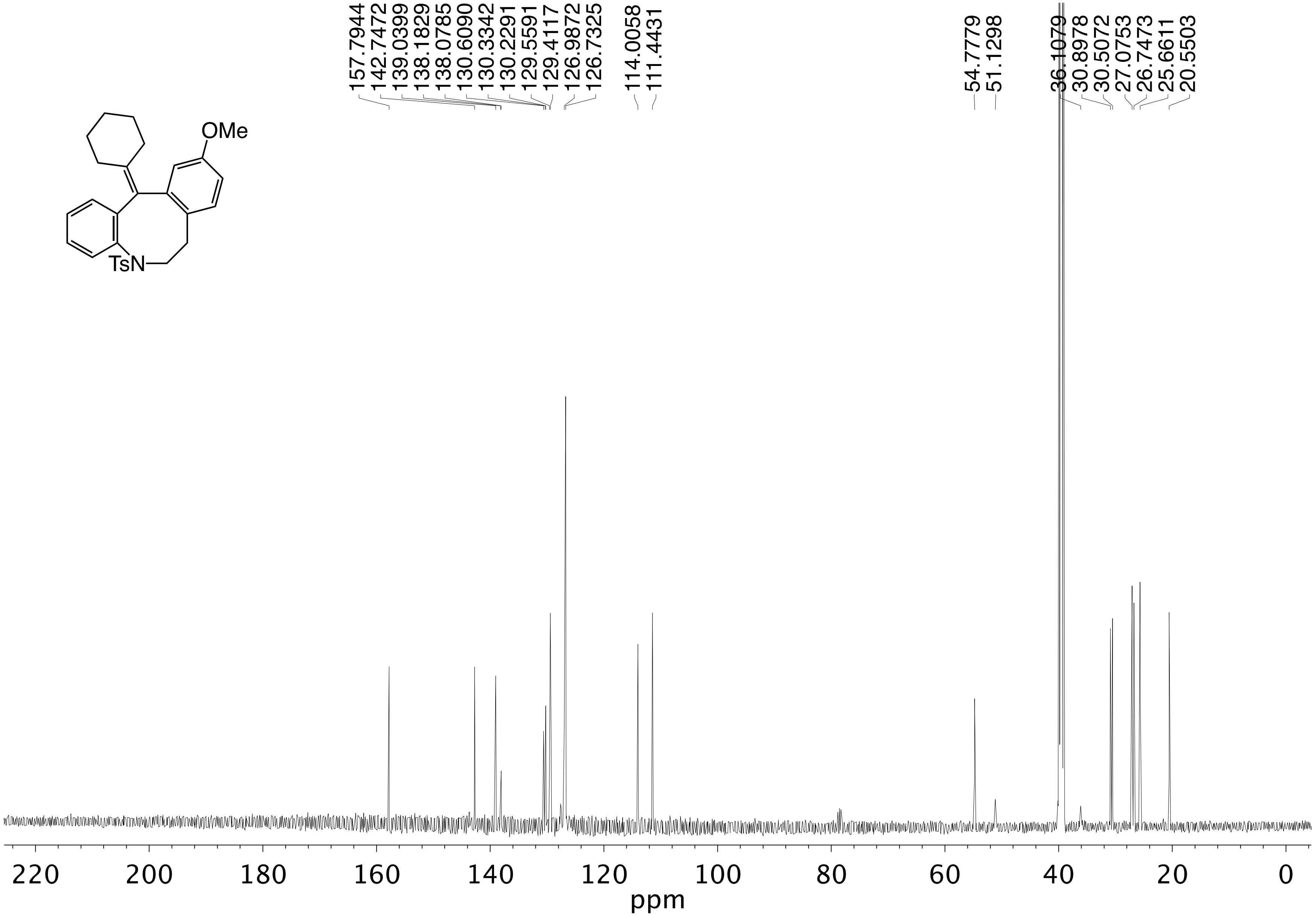

^{13}C NMR (126 MHz, DMSO-d_6, 75°C) of compound **12**.

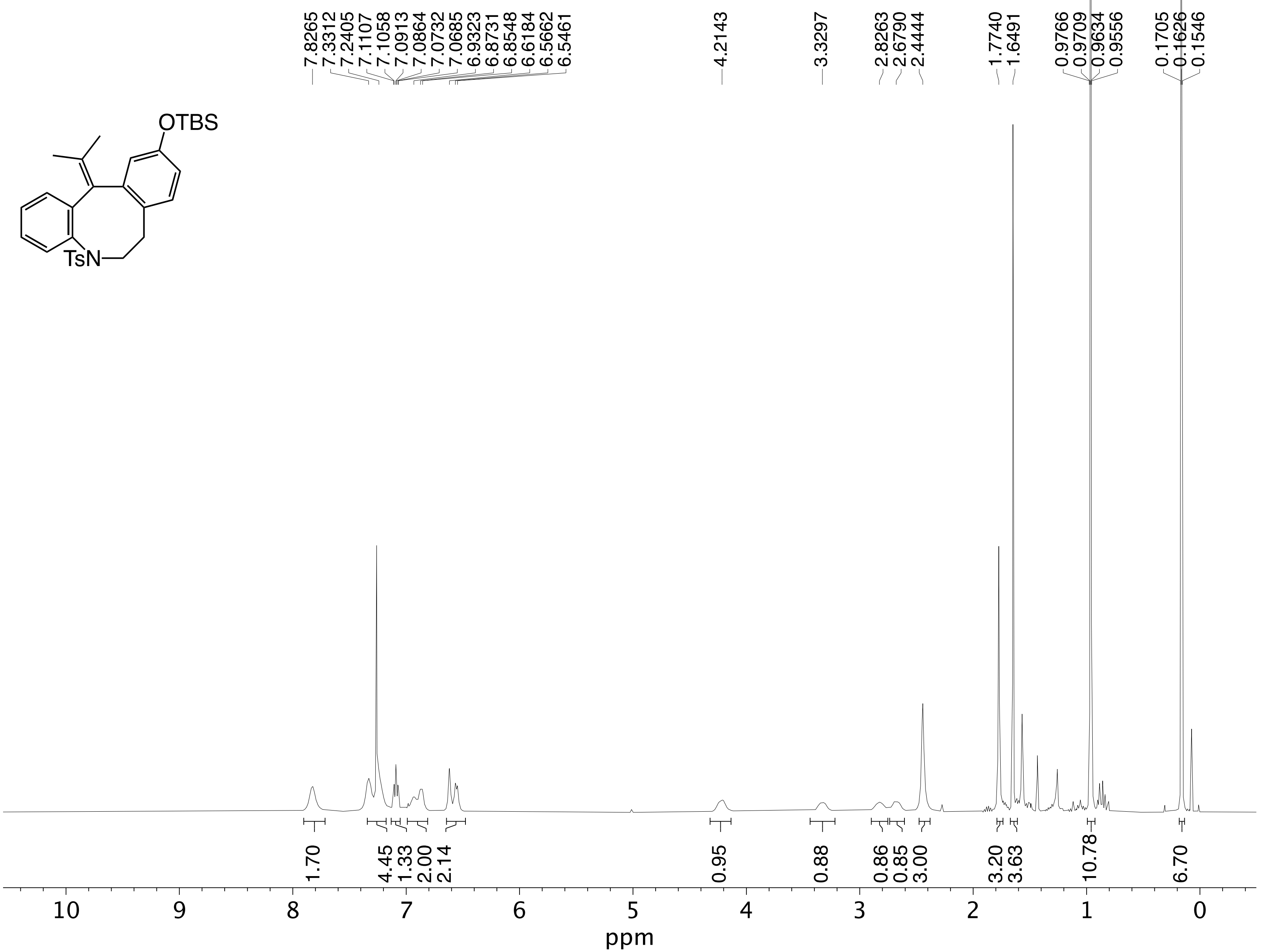

^{1}H NMR (400 MHz, $CDCl_3$) of compound **13**.

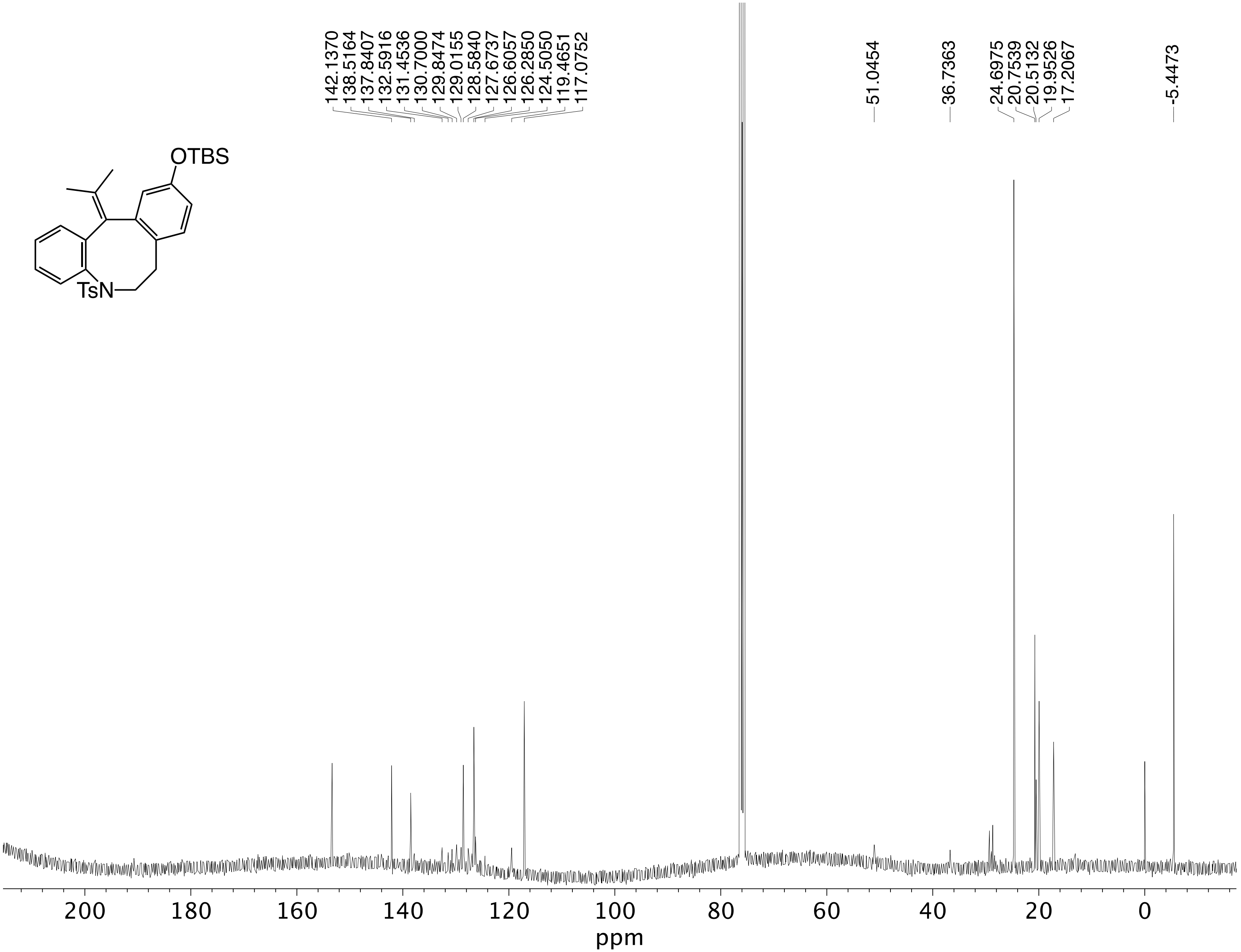

^{13}C NMR (101 MHz, $CDCl_3$) of compound **13**.

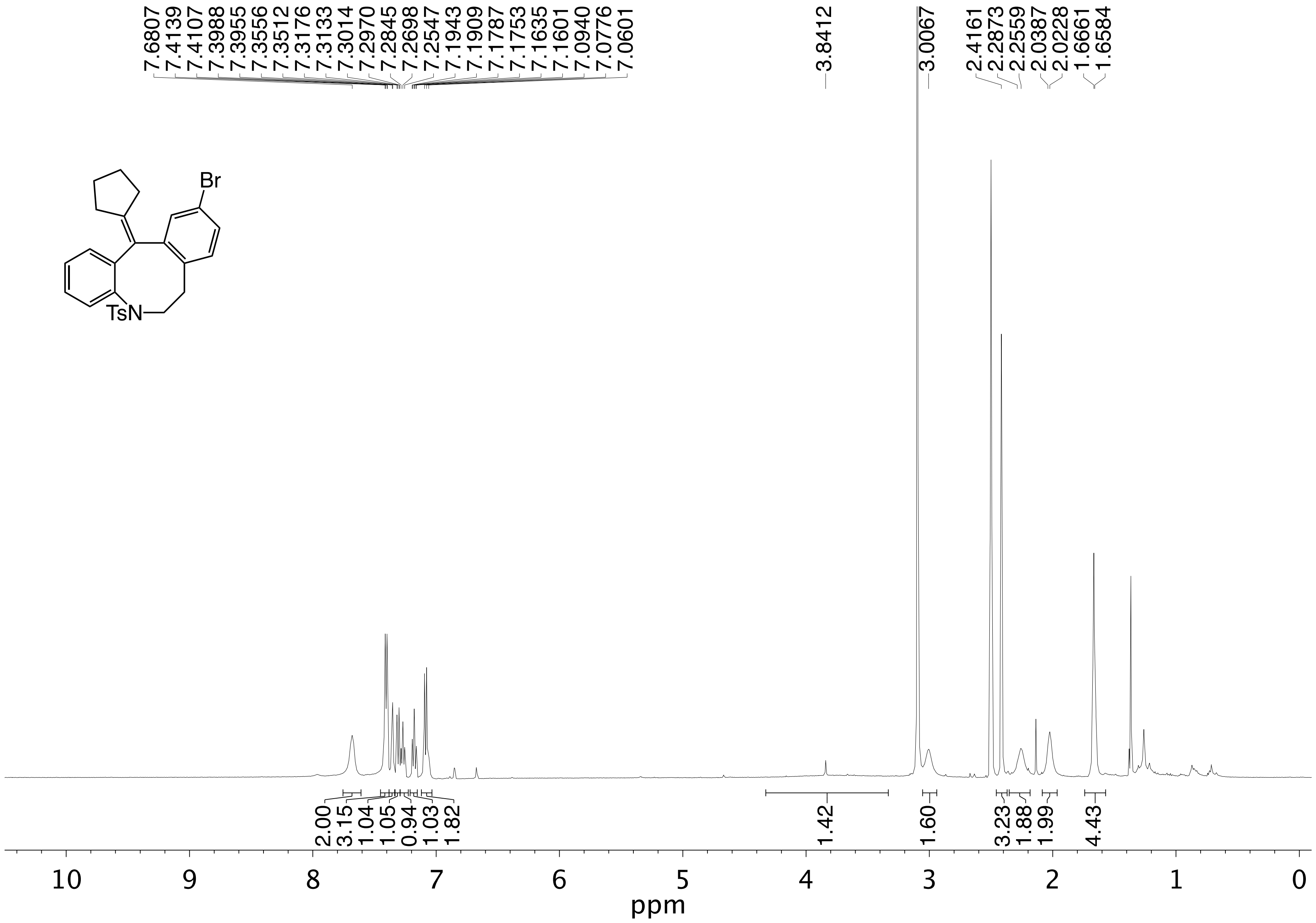

^1H NMR (500 MHz, DMSO-d_6, 75°C) of compound **15**.

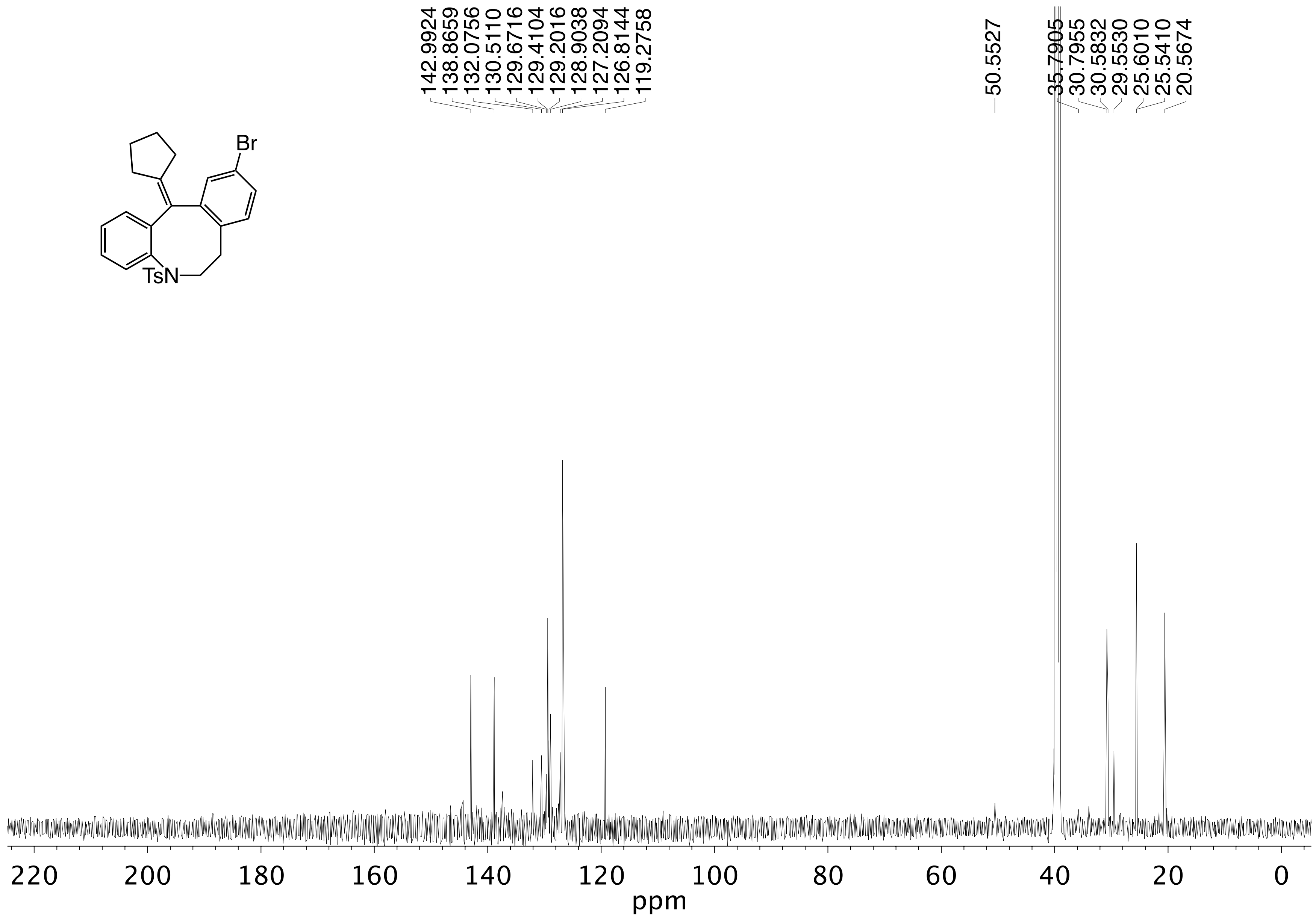

^{13}C NMR (126 MHz, DMSO-d_6, 75°C) of compound **15**.

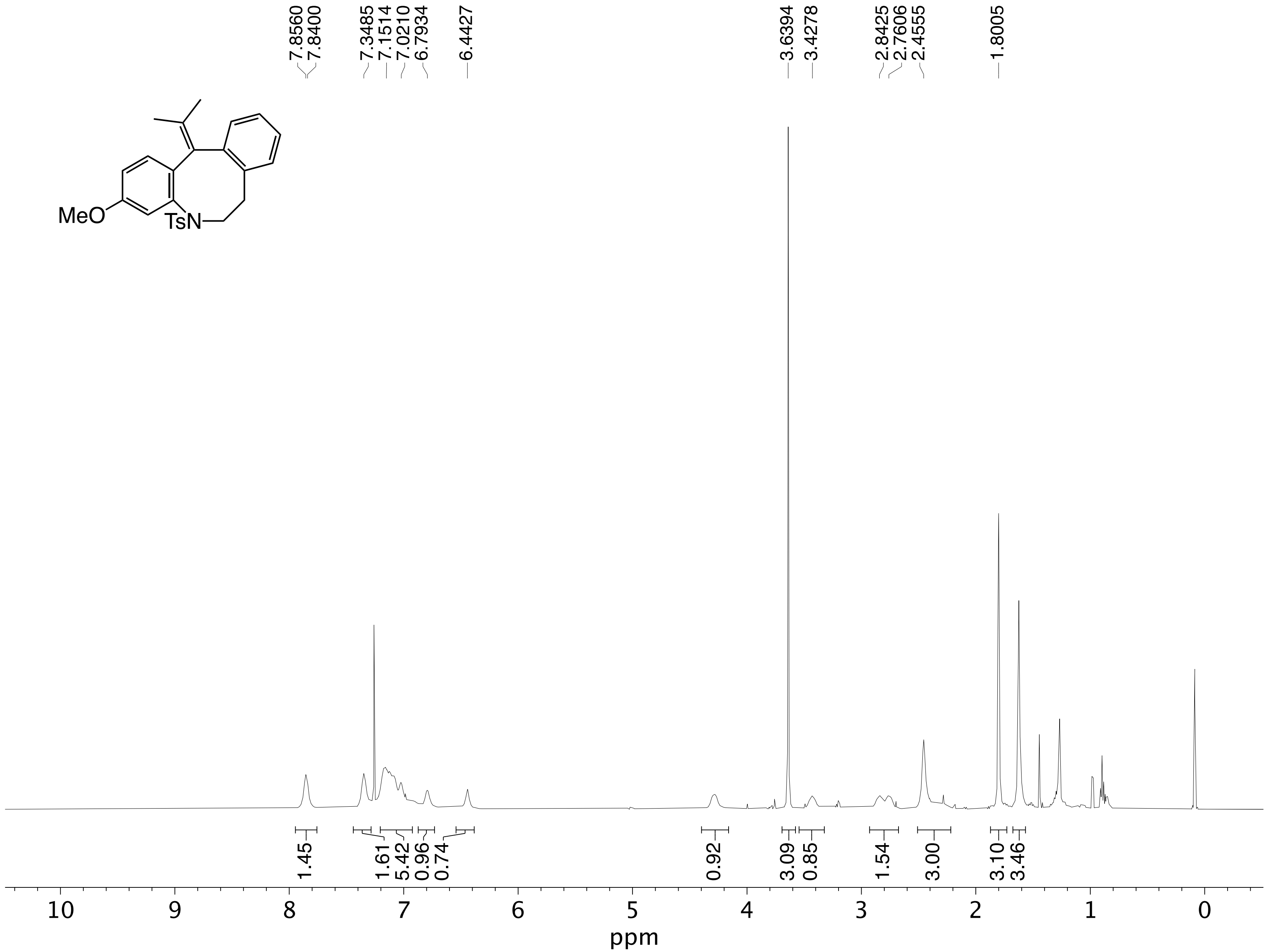

^{1}H NMR (500 MHz, $CDCl_3$) of compound **16.**

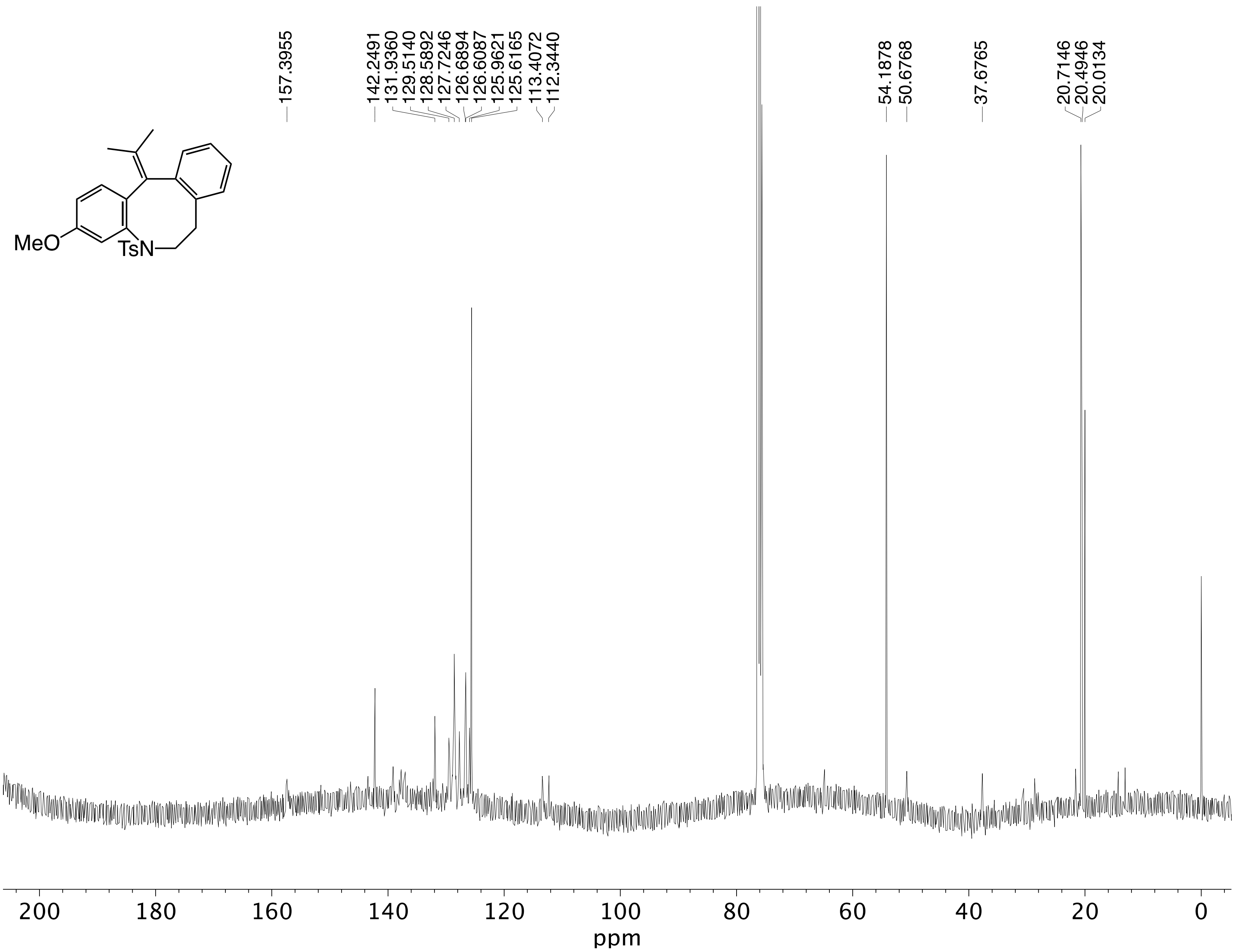

^{13}C NMR (101 MHz, $CDCl_3$) of compound **16.**

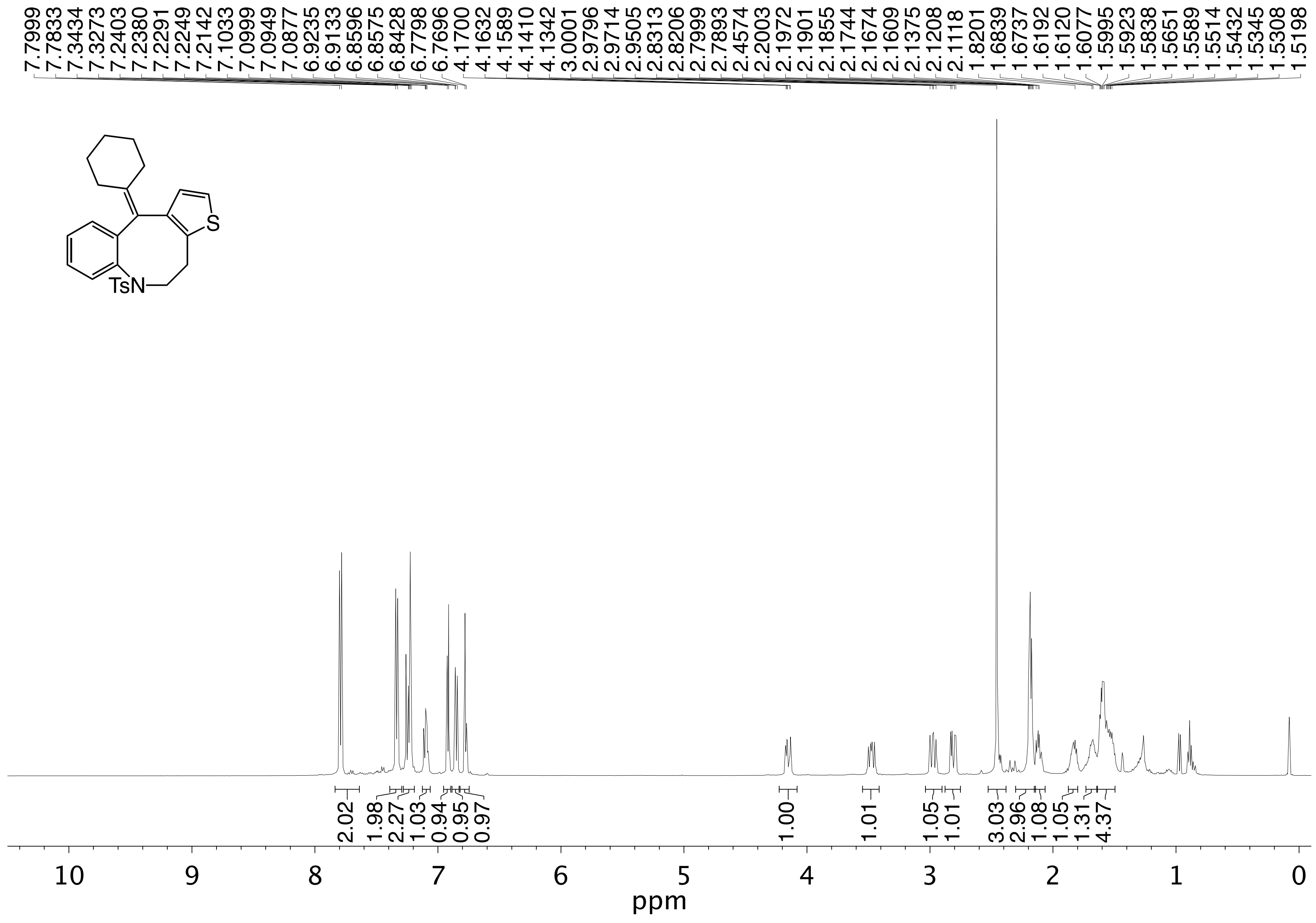

^{1}H NMR (500 MHz, $CDCl_3$) of compound **18**.

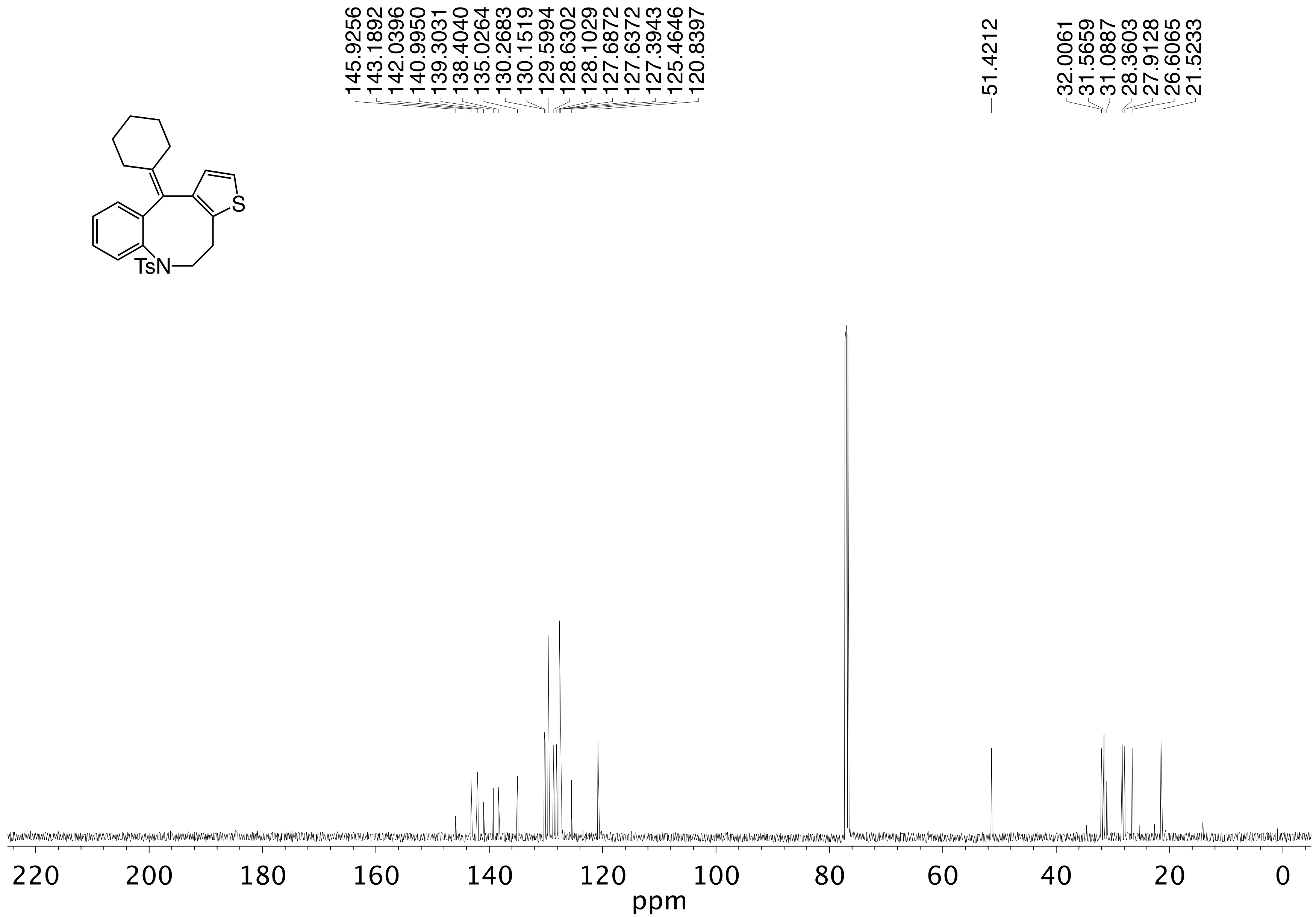

^{13}C NMR (126 MHz, $CDCl_3$) of compound **18**.

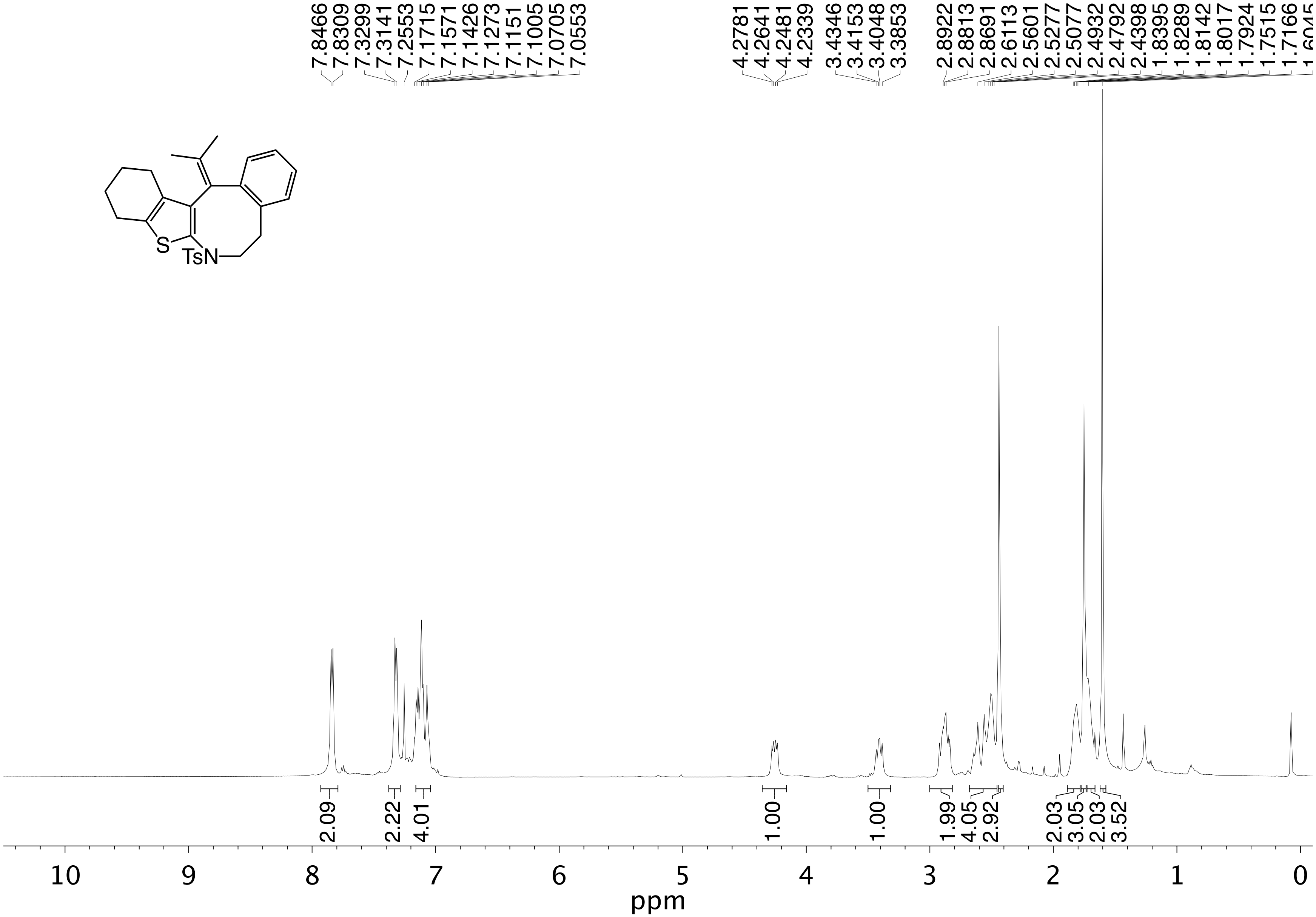

^{1}H NMR (500 MHz, $CDCl_3$) of compound **19.**

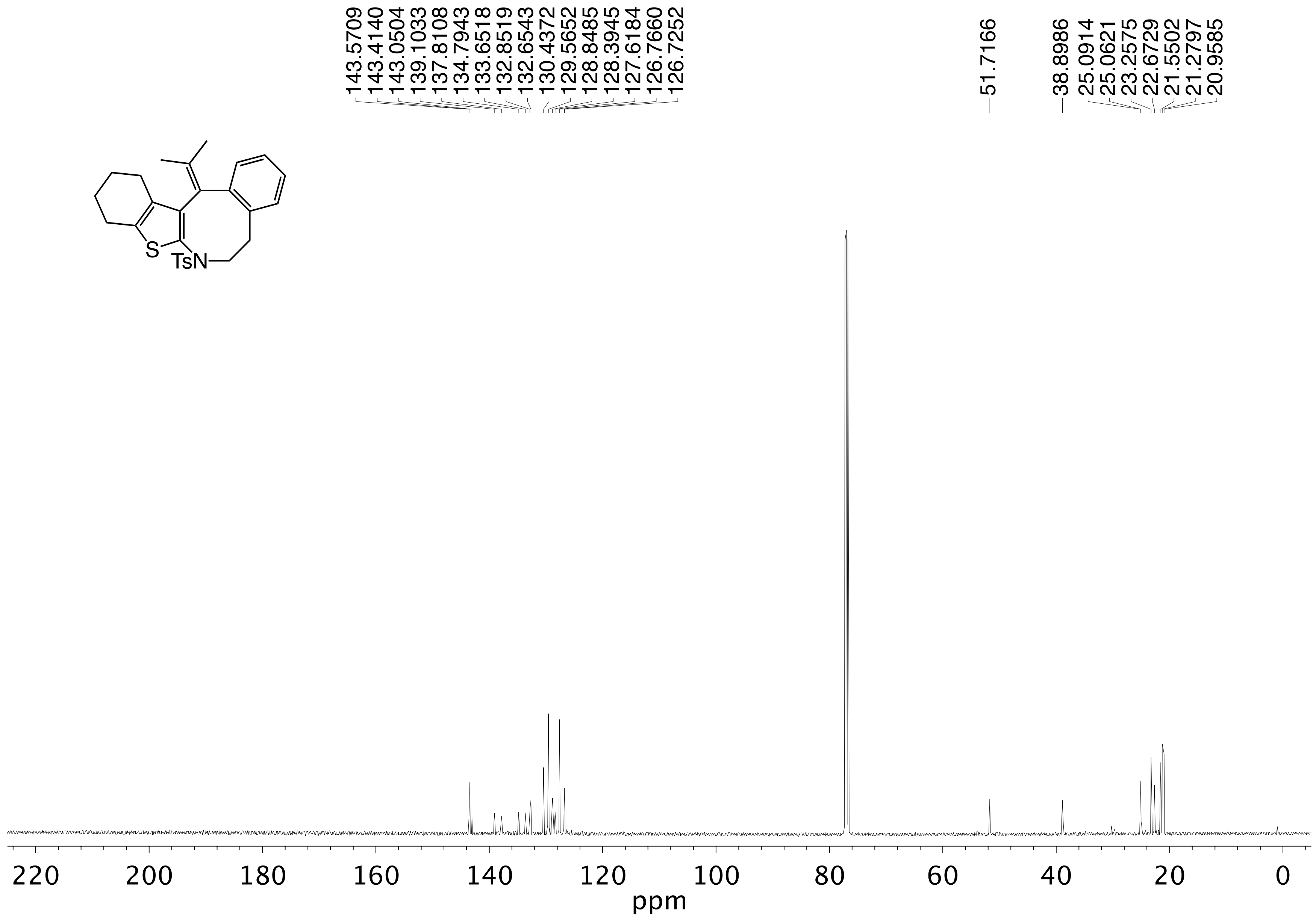

^{13}C NMR (126 MHz, $CDCl_3$) of compound **19.**
